# A global meta-analysis of ITS rDNA sequences from material belonging to the genus *Ganoderma* (Basidiomycota, Polyporales) including new data from selected taxa

**DOI:** 10.3897/mycokeys.75.59872

**Published:** 2020-12-01

**Authors:** Vassiliki Fryssouli, Georgios I. Zervakis, Elias Polemis, Milton A. Typas

**Affiliations:** 1 Agricultural University of Athens, Laboratory of General and Agricultural Microbiology, Iera Odos 75, 11855 Athens, Greece; 2 National and Kapodistrian University of Athens, Department of Genetics and Biotechnology, Faculty of Biology, Panepistemiopolis, Athens 15701, Greece

**Keywords:** Biogeography, fungal diversity, ITS, medicinal mushroom, phylogeny, taxonomy

## Abstract

*Ganoderma* P. Karst. is a cosmopolitan genus of white-rot fungi which comprises species with highly-prized pharmaceutical properties, valuable biotechnological applications and of significant phytopathological interest. However, the status of the taxonomy within the genus is still highly controversial and ambiguous despite the progress made through molecular approaches. A metadata analysis of 3908 nuclear ribosomal internal transcribed spacer (ITS) rDNA sequences obtained from GenBank/ENA/DDBJ and UNITE was performed by targeting sequences annotated as *Ganoderma*, but also sequences from environmental samples and from material examined for the first time. *Ganoderma* taxa segregated into five main lineages (Clades A to E). Clade A corresponds to the core of laccate species and includes *G. shanxiense* and three major well-supported clusters: Cluster A.1 (‘*G. lucidum* sensu lato’) consists of taxa from Eurasia and North America, Cluster A.2 of material with worldwide occurrence including *G. resinaceum* and Cluster A.3 is composed of species originating from all continents except Europe and comprises *G. lingzhi*. Clade B includes *G. applanatum* and allied species with a Holarctic distribution. Clade C comprises taxa from Asia and Africa only. Clade D consists of laccate taxa with tropical/subtropical occurrence, while clade E harbours the highest number of non-laccate species with a cosmopolitan distribution. The 92 *Ganoderma*-associated names, initially used for sequences labelling, correspond to at least 80 taxa. Amongst them, 21 constitute putatively new phylospecies after our application of criteria relevant to the robustness/support of the terminal clades, intra- and interspecific genetic divergence and available biogeographic data. Moreover, several other groups or individual sequences seem to represent distinct taxonomic entities and merit further investigation. A particularly large number of the public sequences was revealed to be insufficiently and/or incorrectly identified, for example, 87% and 78% of entries labelled as *G. australe* and *G. lucidum*, respectively. In general, ITS demonstrated high efficacy in resolving relationships amongst most of the *Ganoderma* taxa; however, it was not equally useful at elucidating species barriers across the entire genus and such cases are outlined. Furthermore, we draw conclusions on biogeography by evaluating species occurrence on a global scale in conjunction with phylogenetic structure/patterns. The sequence variability assessed in ITS spacers could be further exploited for diagnostic purposes.

## Introduction

The genus *Ganoderma* P. Karst. (Basidiomycota, Polyporales) is characterised by a cosmopolitan distribution and high species diversity, especially in the tropics and subtropics. It comprises white-rot fungi that possess an efficient ligninolytic mechanism which is exploited in various biotechnological applications (Ntougias et al. 2012; Zhou et al. 2013; Coelho-Moreira et al. 2018). Many species cause severe diseases (root and butt rots and basal stem rot, differing in terms of invasiveness and host specificity) in economically-important agricultural and forest crops (Elliott and Broschat 2001; Coetzee et al. 2015; Sahebi et al. 2017). *Ganoderma* basidiomes and mycelium biomass have been used for many centuries in traditional medicine mainly in Asia since they are sources of a wide spectrum of bioactive compounds, including polysaccharides, proteins and terpenoids (e.g. ganoderic and lucideric acids) with significant health-promoting and medicinal properties, for example, anti-aging, immunomodulating, anti-cancer, anti-inflammatory, antimicrobial and prebiotic activity (Paterson 2006; Boh et al. 2007; Gargano et al. 2017; Hapuarachchi et al. 2018a; Hsu and Cheng 2018; Khan et al. 2018; Koutrotsios et al. 2019). Nowadays, pertinent *Ganoderma*-derived products are popular worldwide and their development and trade include approximately 800 “ling-zhi products” with a global distribution which are associated with a multibillion-dollar industry (Lai et al. 2004; Li et al. 2016).

*Ganoderma* was erected to include only one species, *G. lucidum* (Curtis: Fr.) P. Karst. (Karsten 1881). Patouillard (1889) added several other laccate taxa with pigmented spores and adhering tubes and subsequent key studies by Murrill (1908), Imazeki (1939)Donk (1948), Steyaert (1972), Corner (1983) and Gilbertson and Ryvarden (1986) discussed the diversity of the genus extensively. The main discriminating character of *Ganoderma* (with respect to other polypores) is the production of double-walled ellipsoid to ovoid basidiospores with truncate or umbonate apex and a coloured endospore with columnar or crest ornamentations (Moncalvo and Ryvarden 1997). The appearance of crust in the upper surface of the basidiome was traditionally used for separating Ganoderma species at the subgenus rank, i.e. subgenus Ganoderma for taxa with laccate-shiny pilei possessing a palisade of inflated hyphae at their upper surface and subgenus Elfvingia for taxa with non-laccate (dull) pilei (Imazeki 1952). Species identification was, until recently, mainly based on the morphology of basidiomes, as well as on ecology and geographical distribution (Corner 1983; Gilbertson and Ryvarden 1986; Ryvarden 2000; Ryvarden 2004; Wasser et al. 2006). However, molecular phylogenetic analyses evidenced that several morphological and cultural characteristics widely used in *Ganoderma* taxonomy were polyphyletic (Moncalvo et al. 1995a; Gottlieb et al. 2000), while the delimitation of various *Ganoderma* species – initially circumscribed using morpho-anatomic and ecological features only – was refined by a range of recent molecular studies (Cao et al. 2012; Zhou et al. 2015; Hennicke et al. 2016; Loyd et al. 2018).

Still, the species concept in *Ganoderma* is not universally accepted and remains inadequately established (Corner 1983; Moncalvo et al. 1995a; Richter et al. 2015; Papp et al. 2017). For many species, phylogenetic and mating data are missing or are fragmentary and not properly documented. In addition, morphological criteria are largely affected by pleomorphic and environmentally-influenced characters and hence their use often leads to unclear and obsolete species descriptions (high phenetic plasticity of morpho-anatomic characters often hinders correct assessment of specimen identity) and subsequently to misidentifications. In addition, the loss or bad condition of type specimens and the failure of lecto-, neo- or epitypification for important taxa have resulted in inconsistencies in taxonomy. Therefore, it comes as no surprise that 456 *Ganoderma* names appear in Mycobank (http://www.mycobank.org/) (Richter et al. 2015), which are estimated to represent from 80 to 290 species worldwide (Ryvarden 2000; Kirk et al. 2008).

The internal transcribed spacer (ITS: ITS1-5.8S-ITS2) region of the nuclear ribosomal RNA was erected as the formal DNA barcode in Fungi since it demonstrates a clear barcoding gap for a wide range of lineages and is often in good agreement with morphological/biological species concepts and could, therefore, be exploited for identification purposes (Schoch et al. 2012; Badotti et al. 2017). Unfortunately, the quality of publicly available fungal ITS sequences varies signifantly and their reliability is often dubious due to mislabelling of the material collected, nomenclatural errors and technical issues, while a large number of submissions are not fully determined or annotated (Nilsson et al. 2006; Bidartondo et al. 2008; Nilsson et al. 2012; Schoch et al. 2012; Kõljalg et al. 2013; Nilsson et al. 2017; Hofstetter et al. 2019). As a consequence, the use of online search tools (e.g. BLAST) is often not helpful and can be misleading. Moreover, the adoption of a specific taxonomic threshold with respect to ITS sequence similarity values (e.g. 97%; Geml et al. 2014; Gweon et al. 2015) deriving from comparisons between species is of questionable usefulness in the case of genera like *Ganoderma*, consisting of numerous taxa with high variability and diverse evolutionary background.

Although the ITS region has been used in more *Ganoderma* studies than any other marker for proposing new taxa (Douanla-Meli and Langer 2009; Kinge and Mih 2011; Cao and Yuan 2013; Coetzee et al. 2015; Li et al. 2015; Xing et al. 2018; Hapuarachchi et al. 2019) and for determining relationships amongst species (Moncalvo 2000; Moncalvo and Buchanan 2008; Wang et al. 2012; Wang et al. 2014; Zhou et al. 2015), published *Ganoderma* phylogenies often show weak support and/or resolution in certain clades (Moncalvo et al. 1995a; Smith and Sivasithamparam 2000; Kinge and Mih 2011; Mohanty et al. 2011; Xing et al. 2016). In addition, limited phylogenetic information is available for many taxa (e.g. the *G. australe* and *G. gibbosum* complexes or as regards species occurring in the Neotropics), thus complicating attempts at resolving their dubious status and relationships. Recent multigene approaches (Zhou et al. 2015; Loyd et al. 2018; Cabarroi-Hernández et al. 2019; Tchotet Tchoumi et al. 2019) provided valuable data towards resolving phylogenetic patterns in *Ganoderma* and mitigated limitations of previous methodologies based mainly (or entirely) on morphological criteria and on the use of single-genetic marker approaches. However, such studies are still confined to a rather low number of species and/or specific geographic regions. In addition, improperly validated material and misidentifications in the records existing in public databases are surfacing and constitute major obstacles at drawing robust taxonomic/phylogenetic conclusions. As a consequence, the distribution patterns for many taxa of the genus remain undefined, species concepts are unclear and interspecific relationships are ambiguous.

On the basis of the discrepancies and shortcomings noted before, the objectives of this study were: (i) to perform a thorough metadata analysis on the basis of a global dataset of *Ganoderma*ITS sequences, (ii) to evaluate the accuracy of specimen identifications to species, (iii) to determine not fully assessed (i.e. “*Ganoderma* sp.”) or erroneously labelled sequences in GenBank (www.ncbi.nlm.nih.gov/genbank/) and other relevant databases, in order to associate taxonomic names with phylogenetic lineages, (iv) to expand the knowledge on distribution and biogeography of *Ganoderma* species, (v) to examine controversial boundaries amongst existing species and complexes and (vi) to contribute to the development of quick and efficient sequence-based tools suitable for identification of *Ganoderma* species through the large-scale assessment of molecular information existing in public databases.

## Methods

### Abbreviations

5.8S: ribosomal DNA 5.8S gene; ASV: amplicon sequence variant; BI: Bayesian Inference phylogenetic analysis; BPP: Bayesian Posterior Probability; BS: Bootstrap Support; DDBJ: DNA Data Bank of Japan; DOI: digital object identifier; DS: dataset; ENA: European Nucleotide Archive, ITS: ribosomal DNA internal transcribed spacer region; ML: Maximum Likelihood phylogenetic analysis; NCBI: National Center for Biotechnology Information.

### Biological material

Dried specimens were obtained on loan from the fungaria of the Bulgarian Academy of Sciences (**FBE**), the Catholic University of Louvain (**MUCL**), the University of Palermo (**PAL**) and the Agricultural University of Athens (**ACAM**). In addition, several specimens were collected from various areas of Greece and pure cultures established are maintained in the fungal cultures collection of the Laboratory of General and Agricultural Microbiology (Agricultural University of Athens), while dried material is preserved in the fungarium of the same institution (ACAM).

We studied 54 specimens in the form of either dried material or pure cultures. They represented well-established *Ganoderma* taxa with European distribution, i.e. four laccate taxa [*G. lucidum* (11 specimens), *G. carnosum* (5), *G. resinaceum* (18) and *G. pfeifferi* (3)] and two non-laccate/dull taxa [*G. adspersum* (8) and *G. applanatum* (5)]. In addition, material not fully identified (*Ganoderma* sp.; 2), together with commercial strains labelled as *G. lucidum* (1) and *G. tsugae* (1), was examined. Initial species labelling was in accordance with the identification made by the respective collector; however, at the end of our study, some of them were re-assessed. Details on their identity appear in Suppl. material 1: Table S1.

### Culture conditions and DNA extraction

Mycelia for DNA extraction were produced in static potato dextrose (Difco, USA) cultures. Following a 10–15 day incubation period at 25 °C, the mycelia were harvested by filtration and either directly processed for DNA extraction or stored at -20 °C. Mycelium or dried basidiome samples were pulverised by a micropestle in the presence of sterile sand and liquid nitrogen. Total genomic DNA was subsequently extracted through the silica Plant II DNA Extraction Miniprep System (Macherey and Nagel, Germany) by following the standard CTAB protocol provided by the manufacturer with minor modifications, i.e. the lysis step was extended to 1 h at 65 °C and the precipitation step to 1 h at room temperature, while the final elution step was performed at 65 °C for 1 h (Zervakis et al. 2014). DNA was quantified by using a Nanodrop ND-1000 spectrometer (Nanodrop Technologies, USA) after which it was adjusted to a final concentration of 50 ng μl^-1^ prior to PCR. DNA extracts were stored in aliquots at -20 °C.

### PCR amplification, sequencing and data assembly

Sequences of the ITS region were generated for phylogenetic analyses. DNA samples were subjected to PCR amplification of the ITS region by using the primer pairs ITS1/ITS4 (White et al. 1990) or ITS1F/ITS4b (Gardes and Bruns 1993). PCR reactions were prepared from genomic DNA in 50 μl PCR reagent containing 1.5 U Takara HiFi (High Fidelity PCR system, Takara Bio USA, Inc.) and 0.25 μM of each primer. The amplification reactions were conducted on a MiniAmp Plus Thermal Cycler (Applied Biosystems, CA, USA). The PCR conditions were as follows: initial denaturation at 95 °C for 3 min followed by 35 cycles of denaturation at 95 °C for 30 sec, annealing at 50 °C for ITS1/ITS4 and 55 °C for ITS1f/ITS4b for 30 sec and elongation/extension at 72 °C for 1 min. A final extension at 72 °C for 10 min was included to complete the reaction. The required controls (positive and negative) were included in all reactions.

Amplified fragments were examined by electrophoresis on 1% agarose gels. PCR products of the expected size were purified by microcentrifugation using the PureLink PCR purification kit (Invitrogen/Thermo Fisher Scientific, USA), according to manufacturer’s protocol. Purified amplicons were processed for bidirectional Sanger sequencing at CEMIA (University of Thessaly, Greece; https://cemia.eu/) using the forward ITS1 and reverse ITS4 primers. The resulting chromatograms were proofread and assembled using Unipro UGENE v.31 (Okonechnikov et al. 2012). Validated sequences were submitted to GenBank and the following accession numbers were obtained: MG706203 to MG706256 (Suppl. material 1: Tables S1, S2).

### Sequence alignment and phylogenetic analyses

**Figure 1.**
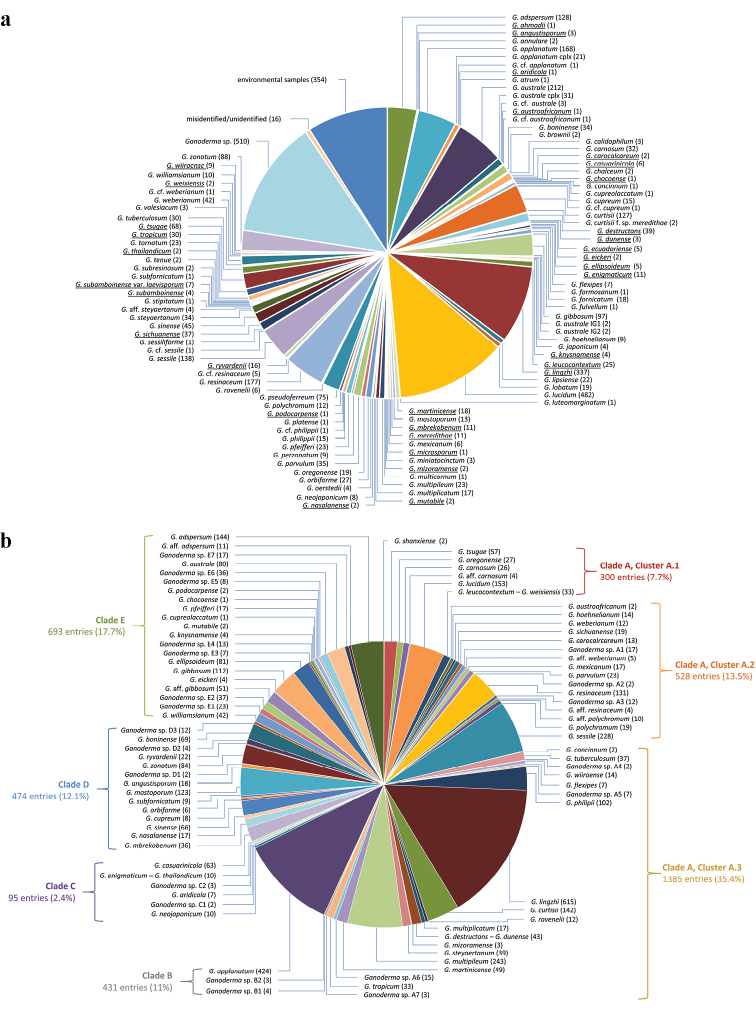
**a** Initial labelling of 3908 *Ganoderma* sequences analysed in the present study: numbers in parentheses correspond to sequences deposited under the particular name in GenBank/ENA/DDBJ and UNITE, while species names appear underlined when ITS sequences derive from type material **b** final assigment of 3908 *Ganoderma* sequences to 80 species and six distinct groups as a result of the phylogenetic analyses performed in this study: numbers in parentheses correspond to the number of sequences grouped within each taxon (data deriving from Table 1 and Suppl. material 1: Tables S2, S4).

An initial dataset was compiled by retrieving and examining all publicly available ITS sequences assigned to the genus *Ganoderma* (followed either by a species epithet or not fully identified and labelled as “*Ganoderma* sp.”), as well as associated environmental samples and misidentified entries, appearing in GenBank/ENA/DDBJ (The International Nucleotide Sequence Database Collaboration; Karsch-Mizrachi et al. 2012) and UNITE (http://unite.ut.ee; Nilsson et al. 2019) until 31 July 2020 (Suppl. material 1: Tables S2–S5). Data derived from 555 batch submissions including 3970 entries were examined by a preliminary BLAST analysis (Altschul et al. 1997) after excluding sequences of short length (< 350 bases). At an initial stage, 62 sequences were excluded since they were erroneously assigned to the genus *Ganoderma* or their identity could not be reliably resolved (Suppl. material 1: Table S3). The rest (3908 entries, including the newly-generated sequences obtained from this study) were compared pairwise for similarity by using the assembly algorithm in Geneious Prime version 11.1.4 (Kearse et al. 2012). Each set of identical sequences was termed as “amplicon sequence variant” (ASV) (Callahan et al. 2017); gaps and degenerated sites were considered as differences. Sequences not grouped in ASVs were those presenting unique sequence profiles (singletons or “singleton ASVs”). All relevant information appears in Table 1, Suppl. material 1: Tables S2, S4, and in Fig. 1a.

**Table 1.** Summarised information on the *Ganoderma*ITS sequences used in this study. Species marked in bold appear as they are presented in Clades and/or Clusters according to the outcome of the phylogenetic analysis (Fig. 3), followed by sequences original labelling in GenBank/ENA/DDBJ and UNITE, the number of sequences examined per taxon name (in parentheses), the geographic origin of the sequenced material, the type of associated host (when available) and the support values the respective terminal clades received (BS in ML and BPP in BI analyses; Figures 4–7). Names marked with asterisk (*) include sequences deriving from type material. A detailed presentation of the pertinent material is provided in Suppl. material 1: Tables S2, S4.

CLADES/Species (no. of sequences per taxon)	Sequences original labelling (no. of sequences per taxon)	Geographic origin of sequenced material	Host type	BS/BPP values
**CLADE A**				**69**%/-
***G. shanxiense* (2)**	*G. shanxiense* (2)	China	AD	100%/1.00
**CLADE A, Cluster A.1**				**100%/1.00**
***G. tsugae* (57)**	*G. tsugae* (46)*, *G. lucidum* (3), uncultured *Ganoderma* (3), *G. ahmadii* (1)*, *G. carnosum* (1), *G. valesiacum* (1), *Ganoderma* sp. (1), *Polyporus tsugae* (1)	Canada, Germany, India, Pakistan, UK, USA	AD, GS	
***G. oregonense* (27)**	*G. oregonense* (15), *G. carnosum* (5), *G. tsugae* (4), *G. lucidum* (2), uncultured soil fungus (1)	Canada, Estonia, USA	GS	
***G. carnosum* (26)**	*G. carnosum* (22), *G. lucidum* (4)	Belgium, Czech Republic, France, Greece, Poland, Slovakia, Slovenia	GS	
**G. aff. carnosum (4)**	uncultured soil fungus (2), *G. carnosum* (1), *G. lucidum* (1)	Estonia, UK		93%/1.00
***G. lucidum* (153)**	*G. lucidum* (107), uncultured soil fungus (28), *G. tsugae* (12), *G. oerstedii* (3), *G. oregonense* (1), *G. valesiacum* (1), *Ganoderma* sp. (1)	Algeria, Argentina, Armenia, Belgium, Bulgaria, China, Czech Republic, Finland, France, Greece, India, Iran, Italy, Norway, Russia, Slovakia, Spain, South Korea, Sweden, Thailand, UK, USA, commercial	AD, GS	
***G. leucocontextum – G. weixiensis* (33)**	*G. leucocontextum* (24)*, *G. lucidum* (3), *Ganoderma* sp. (3), *G. weixiensis* (2)*, *G. carnosum* (1)	China (Tibet), Nepal, Pakistan	AD, GS	81%/1.00
**CLADE A, Cluster A.2**				**94%/1.00**
***G. austroafricanum* (2)**	*G. austroafricanum* (1)*, G. aff. austroafricanum (1)	South Africa	AD	89%/-
***G. hoehnelianum* (14)**	*G. hoehnelianum* (9), *Ganoderma* sp. (5)	China, Gabon, Myanmar		99%/1.00
***G. weberianum* (12)**	*G. weberianum* (9), *G. microsporum* (1)*, *G. sichuanense* (1), *Ganoderma* sp. (1)	Philippines, Taiwan	AD	91%/0.99
***G. sichuanense* (19)**	*G. sichuanense* (9)*, *G. weberianum* (4), *G. lucidum* (2), *G. tenue* (2), *Ganoderma* sp. (1), uncultured soil fungus (1)	Australia, China, India	AD	
***G. carocalcareum* (13)**	*Ganoderma* sp. (8), *G. weberianum* (3), *G. carocalcareum* (2)*	Gabon, Cameroon	AD	-/0.98
***Ganoderma* sp. A1 (17)**	*G. weberianum* (17)	India	AD	100%/1.00
**G. aff. weberianum (5)**	*G. weberianum* (2), G. cf. weberianum (1), *G. subamboinense* (1), *Ganoderma* sp. (1)	Brazil, China, India	AD	
***G. mexicanum* (17)**	*G. mexicanum* (6), G. subamboinense var. laevisporum (5)*, *G. subamboinense* (2), *G. weberianum* (2), *G. sessiliforme* (1), *G. tuberculosum* (1)	Argentina, Brazil, Martinique, Mexico, USA	AD	
***G. parvulum* (23)**	*G. parvulum* (10), *G. weberianum* (5), *Ganoderma* sp. (4), G. subamboinense var. laevisporum (2), *G. stipitatum* (1), *G. subamboinense* (1)*	Brazil, Colombia, Costa Rica, Cuba, French Guiana, Mexico, USA	AD	77%/-
***Ganoderma* sp. A2 (2)**	*G. resinaceum* (1), *Ganoderma* sp. (1)	China		100%/1.00
***G. resinaceum* (131)**	*G. resinaceum* (105), *G. lucidum* (8), *Ganoderma* sp. (8), *G. pfeifferi* (6), G. cf. resinaceum (2), Polyporales sp. (2)	Belgium, Bulgaria, China, Czech Republic, Egypt, France, Greece, India, Iran, Iraq, Italy, Netherlands, Poland, Slovakia, South Africa, South Korea, Tunisia, Turkey, UK	AD	
***Ganoderma* sp. A3 (12)**	*G. resinaceum* (5), G. cf. resinaceum (3), *Ganoderma* sp. (2), *G. lucidum* (1), uncultured *Ganoderma* (1)	Malaysia, Taiwan, commercial	AD	99%/1.00
**G. aff. sessile (4)**	*G. lucidum* (4)	India, Turkey	AD	68%/-
**G. aff. polychromum (10)**	*G. resinaceum* (5), *G. sessile* (2), G. cf. sessile (1), *G. platense* (1), *G. zonatum* (1)	Argentina, USA	AD	98%/1.00
***G. polychromum* (19)**	*G. polychromum* (11), *G. lucidum* (6), *G. sessile* (2)	China, India, USA	AD	93%/1.00
***G. sessile* (228)**	*G. sessile* (134), *G. resinaceum* (60), *Ganoderma* sp. (15), *G. lucidum* (10), *G. oregonense* (2), *G. boninense* (1), *G. lobatum* (1), *G. neojaponicum* (1), *G. polychromum* (1), *G. valesiacum* (1), *Hericium erinaceum* (1), uncultured root-associated fungus (1)	Argentina, China, India, Japan, Russia, South Korea, Taiwan, USA, commercial	AD, AM	78%/1.00
**CLADE A, Cluster A.3**				**76%/1.00**
***G. concinnum* (2)**	*G. chalceum* (1), *G. concinnum* (1)	Brazil		
***G. tuberculosum* (37)**	*G. tuberculosum* (28), *Ganoderma* sp. (6), *Coriolopsis caperata* (1), *G. parvulum* (1), *G. resinaceum* (1)	Brazil, Colombia, Cuba, Martinique, Mexico, Panama, USA	AD	100%/1.00
***Ganoderma* sp. A4 (2)**	*G. lucidum* (2)	Argentina		99%/1.00
***G. wiiroense* (14)**	*G. wiiroense* (9)*, *Ganoderma* sp. (3), *G. lucidum* (2)	Ghana, India, Senegal	AD	100%/1.00
***G. flexipes* (7**)	*G. flexipes* (7)	China, Laos, Vietnam	AD, GS	100%/1.00
***Ganoderma* sp. A5 (7)**	*G. multiplicatum* (7)	China, Myanmar	AD	100%/1.00
***G. philippii* (102)**	*G. pseudoferreum* (75), *G. philippii* (15), *Ganoderma* sp. (9), *G. australe* (2), uncultured soil fungus (1)	China, Indonesia, Malaysia, Thailand	AD	97%/1.00
***G. lingzhi* (615)**	*G. lingzhi* (333)*, *G. lucidum* (206), *Ganoderma* sp. (37), *G. sichuanense* (27)*, *G. tsugae* (5), *Amauroderma rugosum* (1), *G. boninense* (1), *G. calidophilum* (1), *G. cupreum* (1), *G. luteomarginatum* (1), *Haddowia longipes* (1), *Laccaria bicolor* (1)	Bangladesh, China, India, Iran, Iraq, Japan, Laos, Malaysia, Myanmar, Nepal, South Korea, Thailand, commercial	AD, AM	100%/1.00
***G. curtisii* (142)**	*G. curtisii* (124), *G. meredithae* (11)*, *G. lucidum* (3), G. curtisii f. sp. meredithae (2), *Ganoderma* sp. (2)	Mexico, USA, commercial	AD	
***G. ravenelii* (12**)	*G. ravenelii* (6), *G. curtisii* (3), *G. lucidum* (2), uncultured fungus (1)	India, USA	AD, GS	78%/1.00
***G. multiplicatum* (17)**	*G. multiplicatum* (10), *G. perzonatum* (7)	Brazil, Colombia, Mexico	AD	99%/1.00
***G. destructans – G. dunense* (43)**	*G. destructans* (39)*, *G. dunense* (3)*, uncultured soil fungus (1)	Cameroon, South Africa	AD	
***G. mizoramense* (3)**	*G. mizoramense* (2)*, *G. lucidum* (1)	India	AD	
***G. steyaertanum* (39)**	*G. steyaertanum* (34), G. aff. steyaertanum (3), *Ganoderma* sp. (2)	Australia, Indonesia	AD	89%/0.99
***G. martinicense* (49)**	*G. parvulum* (24), *G. martinicense* (18)*, *G. perzonatum* (2), *G. lucidum* (1), *G. oerstedii* (1), *G. tornatum* (1), *G. tuberculosum* (1), *Ganoderma* sp. (1)	Argentina, Brazil, Colombia, Cuba, Martinique, Mexico, USA	AD	93%/1.00
***G. multipileum* (243)**	*Ganoderma* sp. (112), *G. lucidum* (105), *G. multipileum* (22), Agaricales sp. (1), *G. leucocontextum* (1), *G. lingzhi* (1), Polyporaceae sp. (1)	China, India, Nepal, Pakistan, Philippines, Taiwan, Thailand	AD, AM, GS	74%/0.97
***Ganoderma* sp. A6 (15)**	*G. tropicum* (15)	India	AD	100%/1.00
***G. tropicum* (33)**	*G. tropicum* (15)*, *G. fornicatum* (12), *G. williamsianum* (2), *Vanderbylia fraxinea* (2), *Ganoderma* sp. (1), uncultured soil fungus (1)	China, India, Laos, Taiwan, Thailand	AD	
***Ganoderma* sp. A7 (3)**	*G. fornicatum* (3)	Malaysia		100%/1.00
**CLADE B**				**96%/1.00**
***Ganoderma* sp. B1 (4)**	*Ganoderma* sp. (4)	China, USA		100%/1.00
***Ganoderma* sp. B2 (3)**	*G. applanatum* (1), *G. lingzhi* (1), *G. multipileum* (1)	Nepal		100%/1.00
***G. applanatum* (424)**	uncultured soil fungus (230), *G. applanatum* (119), *G. lipsiense* (21), Fungi (plant leaf) (15), uncultured *Ganoderma* (15), uncultured fungus (8), *G. adspersum* (5), *G. applanatum* cplx (3), *Ganoderma* sp. (2), fungal sp. (1), *G. australe* (1), G. cf. applanatum (1), *G. lobatum* (1), *G. oregonense* (1), *Trametes* sp. (1)	Antarctica, Armenia, Austria, Bulgaria, Canada, China, Czech Republic, Estonia, France, Germany, Greece, Hungary, India, Japan, Kyrgyzstan, Latvia, Lithuania, Netherlands, Poland, Russia, Slovakia, South Korea, Thailand, UK, USA, commercial	AD, AM, GS	99%/1.00
**CLADE C**				**100%/1.00**
**CLADE C, Cluster C.1**				**99%/1.00**
***G. neojaponicum* (10)**	*G. neojaponicum* (7), *G. calidophilum* (2), *Ganoderma* sp. (1)	China, Laos, Myanmar, Taiwan	AD	99%/1.00
**CLADE C, Cluster C.2**				**100%/1.00**
***Ganoderma* sp. C1 (2)**	*Ganoderma* sp. (2)	Cameroon	AM	82%/1.00
***G. aridicola* (7)**	*Ganoderma* sp. (6), *G. aridicola* (1)*	Cameroon, South Africa	AD, AM, GS	
***Ganoderma* sp. C2 (3)**	*Ganoderma* sp. (3)	Cameroon	AD	100%/1.00
***G. enigmaticum – G. thailandicum* (10)**	*G. enigmaticum* (7)*, *G. thailandicum* (2)*, uncultured soil fungus (1)	Ghana, Ivory Coast, South Africa, Thailand	AD, GS	
***G. casuarinicola* (63)**	*Ganoderma* sp. (47), *G. casuarinicola* (6)*, *G. enigmaticum* (4), uncultured fungus (2), *G. applanatum* (1), *G. carnosum* (1), *G. lucidum* (1), uncultured *Ganoderma* (1)	China, India, Sri Lanka	AD, AM, GS	71%/-
**CLADE D**				
**CLADE D, Cluster D.1**				**98%/1.00**
***G. mbrekobenum* (36)**	*Ganoderma* sp. (21), *G. mbrekobenum* (11)*, *G. applanatum* (1), *G. carnosum* (1), *G. lucidum* (1), *G. tsugae* (1)	Ghana, India, Senegal, Sri Lanka	AD, AM, GS	98%/1.00
**CLADE D, Cluster D.2**				
***G. nasalanense* (17)**	*G. australe* (9), *Ganoderma* sp. (4), *G. nasalanense* (2)*, uncultured soil fungus (2)	India, Laos, Malaysia, Vietnam	AD	98%/1.00
***G. sinense* (66)**	*G. sinense* (45), *Ganoderma* sp. (8), *G. lucidum* (5), *G. japonicum* (4), *G. subresinosum* (2), *G. atrum* (1), *G. formosanum* (1)	China, Taiwan, Thailand	AD	100%/1.00
**CLADE D, Cluster D.3**				**75%/1.00**
***G. cupreum* (8)**	*G. cupreum* (4), *G. australe* (1), G. cf. cupreum (1), *G. chalceum* (1), uncultured fungus (1)	Cameroon, Gabon, Malaysia, South Africa, Tanzania	AD	99%/1.00
***G. orbiforme* (6)**	*G. orbiforme* (6)	Brazil		89%/0.96
***G. subfornicatum* (9)**	*G. ecuadoriense* (5)*, *Ganoderma* sp. (2), *G. subfornicatum* (1)*, uncultured fungus (1)	Brazil, Ecuador, French Guiana, India, Peru	AD	100%/0.96
***G. mastoporum* (123)**	*G. australe* (60), *G. orbiforme* (19), *G. mastoporum* (13), *Ganoderma* sp. (11), *G. cupreum* (10), uncultured soil fungus (6), *G. fornicatum* (3), *G. multicornum* (1)	Australia, China, India, Indonesia, Laos, Malaysia, Myanmar, Taiwan, Thailand, Vietnam	AD, GS	80%/1.00
**CLADE D, Cluster D.4**				**93%/1.00**
**Group D.4.1**				**87%/1.00**
***G. angustisporum* (16)**	*G. australe* (8), *Ganoderma* sp. (5), *G. angustisporum* (3)*	Australia, China, India, Indonesia, Malaysia	AD, GS	
***Ganoderma* sp. D1 (2)**	*G. applanatum* (2)	Gabon	AD	100%/1.00
**Group D.4.2**				**91%/1.00**
***G. zonatum* (84)**	*G. zonatum* (84)	USA	AD, AM	100%/1.00
***Ganoderma* sp. D2 (4)**	*Ganoderma* sp. (4)	Colombia	AM	100%/1.00
***G. ryvardenii* (22)**	*G. ryvardenii* (15)*, *Ganoderma* sp. (6), *G. wiiroense* (1)	Cameroon, India	AD, AM	100%/1.00
***G. boninense* (69)**	*G. boninense* (32), *Ganoderma* sp. (29), *G. miniatocinctum* (3), *G. zonatum* (3), *G. orbiforme* (2)	China, Indonesia, Japan, Malaysia, Taiwan, Thailand, Vietnam	AM	
***Ganoderma* sp. D3 (12)**	*Ganoderma* sp. (12)	Indonesia	AM	98%/1.00
**CLADE E**				**81%/1.00**
**CLADE E, Cluster E.1**				**99%/1.00**
***G. williamsianum* (42)**	*G. australe* (29), *G. williamsianum* (7), G. cf. australe (2), *G. australe* cplx (2), *Ganoderma* sp. (1), uncultured fungus (1)	China, Malaysia, Myanmar, Thailand	AD	99%/1.00
**CLADE E, Cluster E.2**				**84%/0.99**
***Ganoderma* sp. E1 (23)**	*G. applanatum* cplx (8), *G. tornatum* (7), *Ganoderma* sp. (4), *G. lobatum* (3), *G. gibbosum* (1)	Brazil, Colombia, Costa Rica, Ecuador, French Guyana, Mexico, Peru, USA	AD	87%/0.99
***Ganoderma* sp. E2 (37)**	*G. gibbosum* (12), *G. tornatum* (8), *G. lobatum* (7), *Ganoderma* sp. (6), *G. applanatum* cplx (2), *G. australe* (2)	Argentina, Brazil, Colombia, Cuba, Puerto Rico, USA	AD, AM	96%/1.00
**G. aff. gibbosum (51)**	*Ganoderma* sp. (46), *G. australe* (3), *G. gibbosum* (1), *G. ryvardenii* (1)	India	AD, AM, GS	69%/1.00
***G. eickeri* (4)**	*G. eickeri* (2)*, *Ganoderma* sp. (2)	South Africa	AD	92%/1.00
***G. gibbosum* (113)**	*G. gibbosum* (61), *G. applanatum* (28), *G. australe* (10), *Ganoderma* sp. (3), *G. australe* cplx (2), *G. australe* IG1 (2), *G. lingzhi* (2), Agaricales sp. (1), *G. fulvellum* (1), *G. lucidum* (1), *Fuscoporia viticola* (1), uncultured *Ganoderma* (1)	China, Japan, Laos, Pakistan, South Korea, Taiwan, Thailand	AD, AM	
***G. ellipsoideum* (80)**	*G. gibbosum* (22), *G. australe* (10), *G. australe* cplx (10), *Ganoderma* sp. (10), *G. adspersum* (5), *G. applanatum* (5), uncultured soil fungus (5), *G. ellipsoideum* (5)*, *G. applanatum* cplx (3), *G. tornatum* (3), G. aff. steyaertanum (1), *Tomophagus* sp. (1)	Australia, Cambodia, China, India, Indonesia, Laos, Malaysia, Myanmar, Papua New Guinea, Sri Lanka, Thailand, USA, Vietnam	AD, AM, GS	-/0.99
***Ganoderma* sp. E3 (7)**	*G. australe* (6), uncultured soil fungus (1)	Australia, Indonesia		96%/1.00
***Ganoderma* sp. E4 (13)**	*G. australe* (12), *G. tornatum* (1)	Indonesia, Malaysia	AD, AM	100%/1.00
**CLADE E, Cluster E.3**				**71%/0.97**
***G. knysnamense* (4)**	*G. knysnamense* (4)*	South Africa	AD	100%/1.00
***G. mutabile* (2)**	*G. mutabile* (2)*	China		100%/1.00
***G. cupreolaccatum* (1)**	*G. cupreolaccatum* (1)			
***G. pfeifferi* (17)**	*G. pfeifferi* (17)	Czech Republic, Greece, Slovakia, UK	AD	93%/-
**CLADE E, Cluster E.4**				-/**0.99**
***G. chocoense* (1)**	*G. chocoense* (1)*	Ecuador		
***G. podocarpense* (2)**	*G. podocarpense* (1)*, uncultured soil fungus (1)	Ecuador, Panama		100%/1.00
***Ganoderma* sp. E5 (8)**	*Ganoderma* sp. (4), *G. lobatum* (2), *G. tornatum* (1), uncultured soil fungus (1)	Argentina	AD	100%/1.00
***Ganoderma* sp. E6 (35)**	*G. australe* (19), *Ganoderma* sp. (8), *G. australe* IG2 (2), *G. applanatum* (2), *G. australe* cplx (1), G. cf. australe (1), G. cf. philippii (1), uncultured *Ganoderma* (1), uncultured soil fungus (1)	China, India, Laos, New Zealand, Papua New Guinea, Taiwan, Thailand, Vietnam	AD	95%/1.00
***G. australe* (80)**	*G. australe* (27), *G. australe* cplx (14), *Ganoderma* sp. (13), uncultured soil fungus (10), *G. annulare* (2), *G. applanatum* cplx (2), *G. brownii* (2), *G. lobatum* (2), *G. tornatum* (2), fungal sp. (1), *G. adspersum* (1), *G. applanatum* (1), *G. lipsiense* (1), *G. lucidum* (1), uncultured *Ganoderma* (1)	Argentina, Australia, Brazil, Chile, Costa Rica, India, New Zealand, South Africa, UK, USA	AD, AM	
**CLADE E, Cluster E.5**				**97%/1.00**
***Ganoderma* sp. E7 (17)**	*Ganoderma* sp. (10), *G. applanatum* cplx (3), *G. lobatum* (3), uncultured soil fungus (1)	USA		100%/1.00
**G. aff. adspersum (11)**	*G. adspersum* (4), *G. applanatum* (4), *G. australe* cplx (2), uncultured soil fungus (1)	China, Japan, Korea		72%/-
***G. adspersum* (144)**	*G. adspersum* (113), *G. australe* (13), *Ganoderma* sp. (10), *G. applanatum* (4), basidiomycetes sp. (2), uncultured *Ganoderma* (1), uncultured fungus (1)	Armenia, Belgium, Croatia, France, Georgia, Germany, Greece, India, Iran, Italy, Slovakia, Spain, Tunisia, UK, USA	AD, GS	89%/1.00

Abbreviations used for associated hosts: AD, angiosperm eudicot; AM, angiosperm monocot; GS, gymnosperm.

The principal phylogenetic analysis of ITS sequences, spanning the entire genus, was based on the construction of the main dataset (‘DS’), which was prepared by preferably using ASVs since they were considered more reliable. Selected singletons were also included in the DS when they corresponded to type material, when representing material of diverse origin or under various taxonomic names and in the absence of adequate number of ASVs (four was set as minimum and ten as maximum) for a particular clade. On the basis of the outcome of the phylogenetic analysis performed on the DS, six additional partial datasets (designated as ‘pDS’) were constructed in order to examine in more detail relationships within each of the main phylogenetic groups of the genus as they derived from the analyses performed by including all additional entries available (Table 2). The outgroup taxa *Pycnoporus cinnabarinus* and *Trametes versicolor* were included, as well as 13 additional sequences of seven species representing several closely-related clades (Table 2) selected on the basis of the outcome of a recent study (Costa-Rezende et al. 2017), i.e. *Tomophagus cattienensis* and *T. colossus*, *G. subresinosum*, *G. tsunodae*, *G. ramosissimum* and *G. shandongense*, *Humphreya coffeata* and *G. sandunense*.

Hence, multiple alignments of seven different matrices (Table 2) were performed with the aid of the online version of MAFFT v. 7 (Katoh and Standley 2013; https://mafft.cbrc.jp/alignment/software/) by using the progressive method G-INS-1 and were finally inspected and manually optimised in MEGA X (Kumar et al. 2018). The 5’ end and the 3’ end of ITS1 and ITS2 were determined, based on hidden Markov models (HMMs), localisation deriving from the ITSoneDB (Santamaria et al. 2018) and ITS2 Database (Ankenbrand et al. 2015). Oligonucleotides marking the start and end of ITS regions are: TATCGA to ATATAC for ITS1, AACTTT to TCATGA for 5.8S and AATCTT to TTATGA for ITS2. The ITS alignments were partitioned into ITS1, 5.8S and ITS2 and a substitution model for each partition was selected with jModeltest v. 2.0 (Darriba et al. 2012) by using the corrected Akaike Information Criterion (cAIC; Hurvich and Tsai 1989). The best-fit models of evolution are included in Table 2. The number of variable and parsimony informative characters of each dataset was obtained using PAUP* v4.0b10 (Swofford 2003) (Table 2).

Phylogenetic analyses were based on Maximum Likelihood (ML) and Bayesian Inference (BI). The ML analyses were conducted with RaxML HPC BlackBox running on XSEDE (Stamatakis et al. 2014) under the general time-reversible (GTR) model of nucleotide substitution with gamma distributed rate heterogeneity (GTRGAMMA) for branch confidence with non-parametric bootstrap support (BS) according to MRE-based bootstrapping criteria assessed through the CIPRES Science Gateway-web portal/platform (Miller et al. 2010; http://www.phylo.org/) (Table 2). The BI analyses were conducted in MrBayes v. 3.2.6 (Ronquist et al. 2012). Two analyses using four independent chains (one cold and three heated) were run from a random starting tree and sampled every 1000 generations. Potential scale reduction factors (PSRF) were set to 1.0 for all parameters; each dataset was run for a (total) number of generations which permitted us to obtain values for standard deviation of split frequencies below 0.005 (Table 2). Subsequently, the sampled trees were summarised after omitting the first 25% as burn-in. Bayesian Posterior Probabilities (BPP) of each node were obtained with the majority rule and all compatible partitions were calculated from the remaining trees through a 50% majority rule consensus tree. Resulting trees were visualied using iTOL v. 5.5 (Letunic and Bork 2019). Alignments and phylogenetic trees were deposited in TreeBASE (http://treebase.org) under the submission ID 25723 (http://purl.org/phylo/treebase/phylows/study/TB2:S25723).

**Table 2.** Datasets of *Ganoderma* sequences constructed and details of the phylogenetic analyses conducted in the frame of this study.

Datasets constructed and analysed	No. of sequences used/total	Represented entries/total entries	Alignment length	Constant characters*	Parsimony Informative characters*	No. of Rapid Bootstraps	ML Optimisation Likelihood	Model substitution AICc TS1/5.8S/ITS2	No. of generations	Split frequency	50% credible trees	ML trees presented
DS: entire genus and individual Clades/Clusters	440/2119	2027/3908	713	328	307	504	-10429.843785	GTR+G/GTR/TVM+G	37885000	0.004398	28415	Figs 3, 4–7
pDS1a: Cluster A.1 (expanded)	120/124	297/301	571	500	25	600	-1701.266373	JC/JC/JC	13710000	0.004400	10285	Suppl. material 3: Fig. S2a
pDS1b: Cluster A.2 (expanded)	263/274	517/528	608	436	86	600	-2929.177334	TIM2ef+G/GTR+G/TIM3+G	63120000	0.004399	47341	Suppl. material 4: Fig. S2b
pDS1c: Cluster A.3 (expanded)	341/641	694/1385	648	376	156	552	-4501.116292	GTR+G/TVM/TVM+G	49780000	0.004400	37337	Suppl. material 5: Fig. S2c
pDS2/pDS3: Clades B & C (expanded)	26/292 67/74	224/431 88/95	607	560/459	38/89	600	-2652.570067	K80/JC/JC	7390000	0.004365	5545	Suppl. material 6: Fig. S2d
pDS4: Clade D (expanded)	292/316	449/474	631	391	149	504	-4424.884308	TPM3uf+I+G/TPM2/TPM1uf+I+G	40320000	0.004398	30241	Suppl. material 7: Fig. S2e
pDS5: Clade E (expanded)	367/396	664/693	656	386	162	552	-5123.351039	TIM3+G/GTR/TPM3uf+G	65585000	0.004398	49190	Suppl. material 8: Fig. S2f

### ITS sequence variation, diagnostic regions and phylogenetic species in *Ganoderma*

Elaboration of ITS metadata made it possible to assess the phylogeny of *Ganoderma* species under study and the support it received; values of bootstrap support (BS) in ML and Bayesian Posterior Probability (BPP) in BI analyses were considered significant and retained when equal or higher than 65% and 0.95 in ML and BI analyses, respectively. Moreover, inter- and intra-specific pairwise genetic distances (on the basis of uncorrected p-values) within and between allied *Ganoderma* taxa were calculated in MEGA X as the proportion (p) of nucleotide sites at which two sequences was different and was obtained by dividing the number of nucleotide differences by the total number of nucleotides. In addition, ITS sequence similarities were computed in MAFFT through the EMBL-EBI portal. Indicative cases are illustrated by boxplot graphs depicting genetic distances and sequence similarities in the main clades, as well as within and amongst selected species of the genus *Ganoderma*.

Widely-adopted thresholds for separating amongst species in Basidiomycota are < 97% to 98% for ITS sequence similarity and > 0.010 to 0.020 for genetic distance uncorrected p-values (Smith et al. 2007; Hughes et al. 2009; Matheny et al. 2009; Schoch et al. 2012; Kondo et al. 2018; Vu et al. 2018; Zervakis et al. 2019). Although such values were generally taken into account in this study, they were not found suitable for universal application in the genus *Ganoderma*. Therefore, phylogenetic species were accepted and/or discussed after examining each case individually through the evaluation of available information (including the number and origin of sequences analysed). Apart from the thresholds quoted above, of importance was whether the terminal subclade was statistically supported and if no overlap (i.e. presence of barcoding gap) were noted between the intraspecific divergence within each taxon and the interspecific variability amongst related taxa. Especially as regards the new phylogenetic species proposed hereby, they had to fulfil all of the following criteria: (a) form a terminal clade with strong support, (b) present mean values of sequence similarity < 98% and genetic distance > 0.015 vs. the closest species terminal clade and (c) no overlap exists between intraspecific values of genetic distance and sequence similarity vs. the respective interspecific values from comparisons to the closest-related taxon. In addition, these phylospecies were linked with the corresponding DOIs of UNITE (Suppl. material 1: Table S2).

Moreover, in order to provide additional information about the variation existing in the ITS spacers for the entire genus, as well as for each major clade/cluster derived from the phylogenetic analyses, the length and GC content of ITS1 and ITS2 were calculated in Geneious Prime version 11.1.4 (https://www.geneious.com) by examining all sequences used for the construction of the main phylogenetic tree (Fig. 3). Finally, two highly-polymorphic regions, one in each spacer, are flanked by conserved oligonucleotides (from TGCAC to GAATG in ITS1 and from AATCT to TAGCT in ITS2) serving as anchor points for sequence search; consequently, these species-specific oligonucleotides could be potentially exploited for diagnostic purposes, especially in taxa/groups represented by an adequate number of entries (e.g. > 10).

## Results and discussion

### Analysis of *Ganoderma*ITS rDNA sequences

In total, 3970 ITS entries were retrieved from the GenBank/ENA/DDBJ and UNITE databases; 62 sequences were removed from further analysis since they were either erroneously annotated as *Ganoderma* (58) or they could not be reliably identified (Suppl. material 1: Table S3). In the meta-analysis performed, 3908 entries were employed (Fig. 1a) and these were separated into 1735 unique sequences (singletons) and 384 ASVs representing 2173 entries (Table 1, Suppl. material 1: Tables S2, S4). Amongst them, 354 (9%) corresponded to environmental samples (e.g. entries labelled as “uncultured *Ganoderma*”, “uncultured fungus” or “uncultured soil fungus”), 16 were either misidentified (e.g. *Coriolopsis caperata*, *Hericium erinaceum* and *Laccaria bicolor*) or not fully identified (e.g. “Agaricales sp.” and “basidiomycetes sp.”), 510 (13%) were deposited as “*Ganoderma* sp.”, while the rest (3028) were labelled with 91 *Ganoderma*-associated taxon names (Table 1, Fig. 1a); this number does not include material with “aff.”, “cf.”, “cplx”, f. sp.”, “IG1” and “IG2” in their labelling.

**Figure 2.**
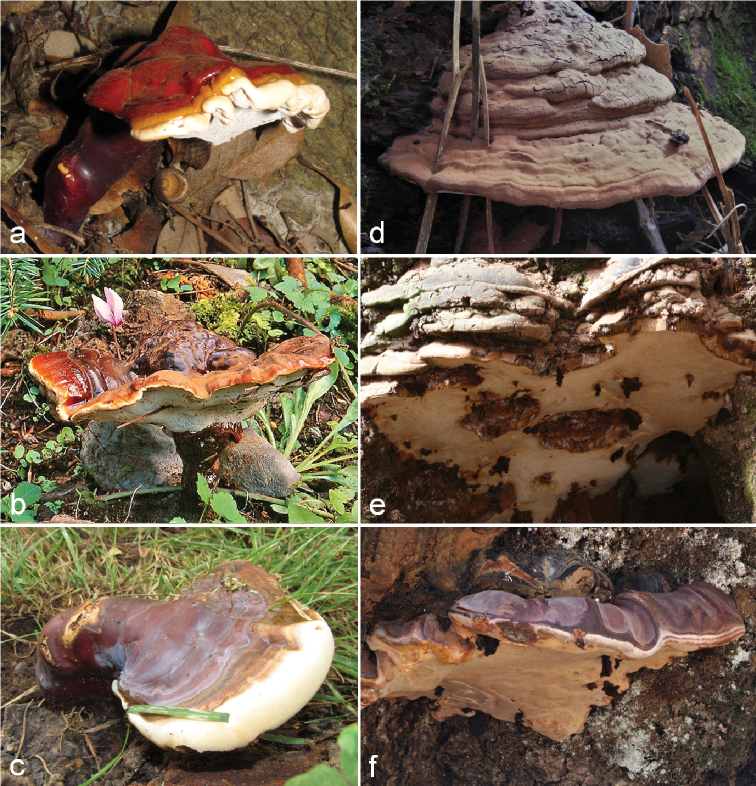
Basidiomes of *Ganoderma* spp. amongst those collected and analysed in this study (specimens codes appear in parantheses; Suppl. material 1: Table S1) **a***G. lucidum* (A1180) **b***G. carnosum* (DD1243) **c***G. resinaceum* (2012-0077) **d***G. adspersum* (2010-0015) **e***G. applanatum* (DD2119) **f***G. pfeifferi* (DD2118).

Almost half (45.3%) of all *Ganoderma* sequences deposited in GenBank/ENA/DDBJ and UNITE correspond to only eight species names, i.e. *G. lucidum* (12.3%), *G. lingzhi* (8.6%), *G. australe* (5.4%), *G. resinaceum* (4.5%), *G. applanatum* (4.3%), *G. sessile* (3.5%), *G. adspersum* (3.3%) and *G. curtisii* (3.3%) (Fig. 1a). On the other hand, 17 species names are represented by only one sequence each (seven of them derive from the type material), i.e. *G. ahmadii* (type), *G. aridicola* (type), *G. atrum*, *G. austroafricanum* (type), *G. chocoense* (type), *G. concinnum*, *G. cupreolaccatum*, *G. formosanum*, *G. fulvellum*, *G. luteomarginatum*, *G. microsporum* (type), *G. multicornum*, *G. platense*, *G. podocarpense* (type), *G. sessiliforme*, *G. stipitatum* and *G. subfornicatum* (type). Sequences from type material were available for a somewhat modest 33 taxa (Table 1, Fig. 1a). Moreover, 54 sequences from commercial strains (originally labelled as *G. lucidum* and *G. tsugae*, but turned out to be *G. lingzhi*) and European collections (*G. adspersum*, *G. applanatum*, *G. carnosum*, *G. lucidum*, *G. pfeifferi* and *G. resinaceum*) were generated for the first time and their details are given in Suppl. material 1: Table S1, while indicative photos of the collected basidiomes appear in Fig. 2.

For inferring the phylogeny of the entire genus, the main dataset (DS) included 2027 entries (i.e. ca. 52% of the total number of *Ganoderma* entries analysed) corresponding to 161 singletons and 279 ASVs (Table 1 and Suppl. material 1: Table S2). The molecular data matrix consisted of 713 aligned characters, of which 328 were constant, 78 were variable, but not parsimony informative and 307 were parsimony informative (Table 2); ITS1 aligned in 289, 5.8S in 162 and ITS2 in 262 positions. Expanded datasets (i.e. pDS1a, pDS1b, pDS1c, pDS2/ pDS3, pDS4 and pDS5) were also used for examining in greater detail phylogenetic relationships/affinities and to elucidate the identity of material within particular clades/clusters and detailed/expanded trees were then constructed (Cluster A.1, Cluster A.2, Cluster A.3, Clades B and C, Clade D and Clade E, respectively; Table 2). However, 642 singletons, which were initially included in the analysis, do not appear in the trees constructed due to over-representation of certain species (i.e. *G. lingzhi*, *G. multipileum* and *G. applanatum*) or to their particularly high heterogeneity causing destabilisation of the phylogenetic estimate via long-branch formation (Suppl. material 1: Table S4).

A comprehensive evaluation of all ITS sequences available permitted us to determine variation in the ITS1 and ITS2 spacers by examining their length and GC content; relevant data are presented below. In addition, the comparative assessment of ITS1 and ITS2 heterogeneity amongst *Ganoderma* species could subsequently contribute to species determination since they contain information of potential diagnostic value. The multiple sequence alignment revealed two polymorphic segments which could be potentially used for the identification of *Ganoderma* specimens at species level (Table 3; Suppl. material 2: Figure S1), while they could also be exploited for the development of species-specific primers. Pertinent results are discussed for each case separately in the respective parts of the following section.

**Table 3.** Summary of polymorphic regions in ITS1 and ITS2 spacers assessed in *Ganoderma* species/groups represented by ≥ 10 entries in the GenBank/ENA/DDBJ and UNITE databases. For each region, the length and position between conserved oligonucleotides (i.e. TGCAC to GAATG in ITS1 and AATCT to TAGCT in ITS2) are hereby provided. Additional pertinent information is included in Suppl. material 2: Fig. S1. Grouping in Clades/Clusters, as well as names and number of sequences per name, are in accordance with Table 1 and Fig. 3.

Species/Groups	ITS1 sequence of potential diagnostic value	Length (nt)	Position in the alignment of Suppl. material 2: Fig. S1 (without gaps)	ITS2 sequence of potential diagnostic value	Length (nt)	Position in the alignment of Suppl. material 2: Fig. S1 (without gaps)
**CLADE A**						
**Cluster A.1**						
*G. oregonense*		13	25–37	GCCTTTGCGGGTW	26	17–42
*G. tsugae*	TGTGAAGCGTGCT	13	25/26–37/38	TGYRGGCTTGGAC	26	17–42
*G. carnosum*		13	25–37	AGCCTTGC	8	16–23
*G. lucidum*	TGAAGCGYNCCYY	13	27–39	nd		
*G. leucocontextum* – *G. weixiensis*	CGAAGCGTGC	10	27–36	nd		
**Cluster A.2**						
*G. hoehnelianum*	CTTCAGTC	8	16–23	CTTGTGGGTT	10	20–29
*G. weberianum*	nd			nd		
*G. sichuanense*	nd			nd		
*G. carocalcareum*	AACGTCGTKAAGCGGGC	17	21–37	nd		
*Ganoderma* sp. A1	GGGTCTTTT	9	34–42	CGTCTTTC	8	60–67
*G. mexicanum*	GCTCTTTACTGAGCC	15	36–50	CGGCCGGCTCCTCT	21	65/67–85/87
*G. parvulum*		15	36–50	TAAATGC^1^	21	65/67–85/87
*G. resinaceum*	AAGCGGCG	8	55/56–62/63	nd		
*Ganoderma* sp. A3	GGATCGGCGT	10	55–64	ACAGATCT	8	13–20
*G. polychromum*	ACACCTAT	8	84–91	nd		
*G. sessile*	CCACAAACTCTR	12	78–89	CTTACAAA	8	10–17
**Cluster A.3**						
*G. tuberculosum*	GATTGTCG	8	21–28	CCATGCCC	8	58/59–65/66
*G. wiiroense*	GGCATTAT	8	21–28	TTCTCTTA	8	71/72–78/79
*G. philippii*	TTGCTGGG	8	39–46	CTTTTGTGGYTTT	13	18–30
*G. lingzhi*	CAGATTGC	8	19–26		10	54–63
*G. curtisii*	TGCGGAGCGCAT	12	49–60	CGGCCGTTAT	10	54–63
*G. ravenelii*	GAGTGCAT	8	53–60		10	54–63
*G. multiplicatum*	CCCTTTAT	8	35–42	nd		
*G. destructans* – *G. dunense*		9	22–30	nd		
*G. steyaertanum*	ATCVTAAAA^2^	9	22–30	CTCTTGGCC	9	61–69
*G. martinicense*		9	22–30	CATTCTTG	8	59–66
*G. multipileum*		9	22–30	G(C)AAGCTTTTG	10–11	13–22/23
*Ganoderma* sp. A6	TCCCAGGA	8	50–56	CTCCTCTCTT	10	72–81
*G. tropicum*	ACCGGGCTTTGCA	13	42–54	nd		
**CLADE B**						
*G. applanatum*	GTGCTYTT	8	32–39	TAAGCTTKTGT	11	14–24
**CLADE C**						
*G. neojaponicum*	ATGGATCGCG	10	18–27	AGGTGTTTG	9	47–55
*G. enigmaticum* – *G. thailandicum*	CTTCTTGTC	9	35–43	TTGCAACC	8	11–18
*G. casuarinicola*	GCTCTTGT	8	34–41		8	11–18
**CLADE D**						
*G. mbrekobenum*	TTWCAGASSGT	11	16–26	AGGCTATT	8	48–55
*G. nasalanense*	CGTTTTCA	8	70–77	TCTTTAATA	9	60/62–68/70
*G. sinense*	GGAGCTSGT	9	41–49	GTAAAGGC	8	24–31
*G. mastoporum*	nd			TTTTTARYGRKTTTGTAGG	19	19–37
*G. angustisporum*	GTGTAAAA	8	27–34	ATGGCTWGT	8	24/28/29–32/36/37
*G. zonatum*	TCGCTCGC	8	34–41	TCTCTTCA	8	3–10
*G. ryvardenii*	TCGTGCGG	8	23–30	CTTTAACT	8	61–68
*G. boninense*	GTTTGACRAGTT	12	40/44–51/55	ATCTCTTTGY	10	16–25
*Ganoderma* sp. D3	GGCGTGGT	8	24–31		10	16–25
**CLADE E**						
*G. williamsianum*	CTTCAGGTC	9	16–24	CTTAATYGA	9	21–29
*Ganoderma* sp. E1	GTTTTACG	8	15–22	ATRAGCTTCT	10	13–22
*Ganoderma* sp. E2		8	15–22	TATGKGAG	8	23–30
G. aff. gibbosum		13	27–39		10	60–69
*G. gibbosum*	TGARRSGGGCTYG^3^	13	27–39	TCCYTTTACR^3^	10	60–69
*G. ellipsoideum*		13	27–39		10	60–69
*Ganoderma* sp. E4	RTTAAACG	8	26–33	GTCGGACTW^4^	9	59–67
*G. pfeifferi*	GGCCCGTTT^5^	9	34/35–42/43	GCCTTTGTC^6^	9	57–65
*Ganoderma* sp. E6	ACYGAGCYYGC	11	41–51	TCTTTGCGGGG	11	19–29
*G. australe*	CGAAACGKGCTCG	13	27–39		11	19–29
*Ganoderma* sp. E7	CCCCATGA	8	83/84–90/91	GTCTTTACA	9	59–67
G. aff. adspersum	GGGCCCGTTC	10	33–42	CTTCTTGCGG	10	18–27
*G. adspersum*	AGGCCCGTTC	10	33–42	AGGTTTGTAGGG	12	27/28–38/39

### Phylogenetic relationships in the genus *Ganoderma*

**Figure 3.**
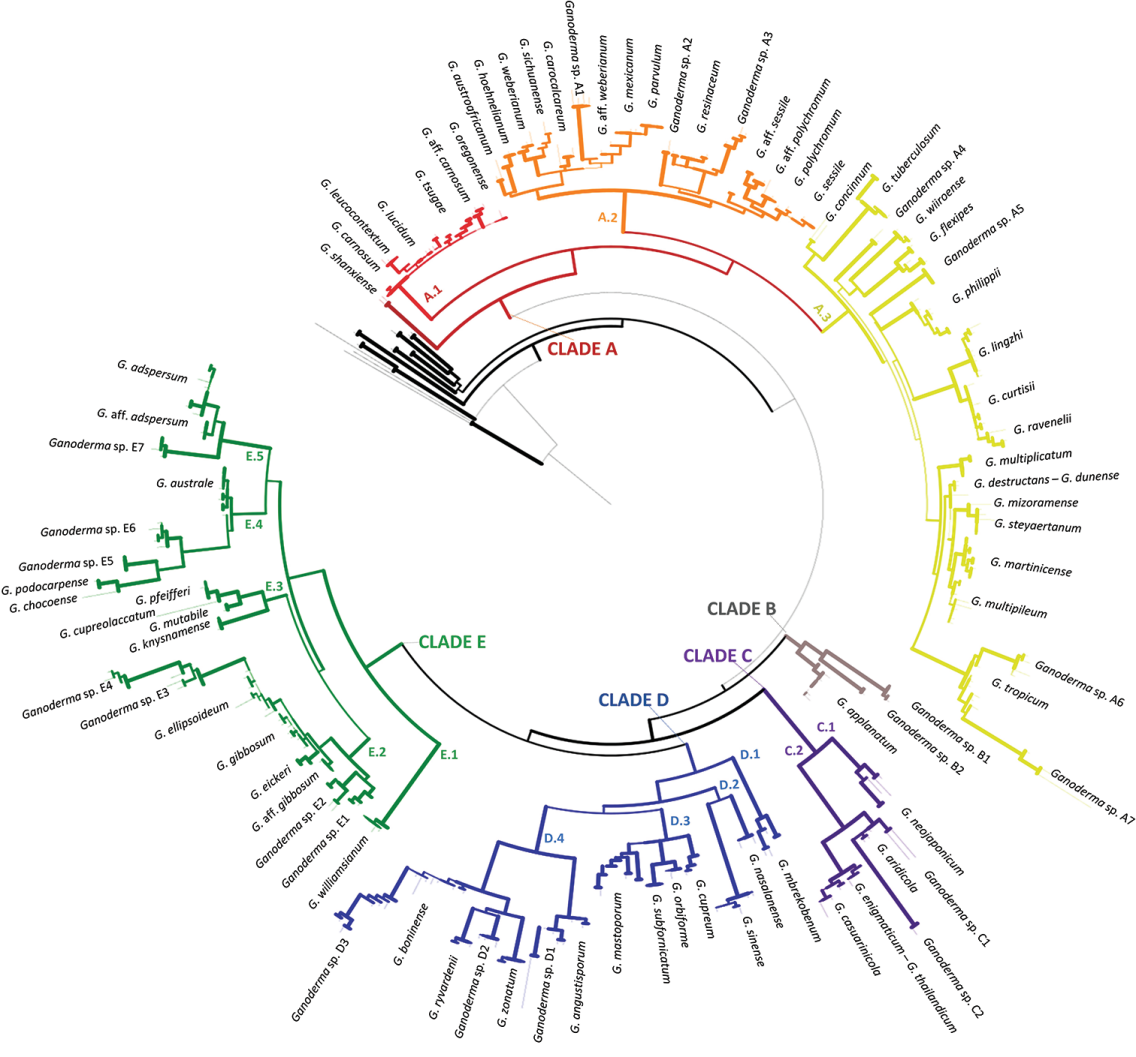
Summary tree of the genus *Ganoderma* inferred from ML analysis, based on ITS sequence data (main dataset, DS; Table 2). Thick lines represent ML bootstrap values (BS) ≥ 65% and Bayesian Posterior Probabilities (BPP) ≥ 0.95. Clades and Clusters within the tree appear as presented in Table 1 and Suppl. material 1: Table S2. Species names correspond to those inferred in this study. Scale bar: 0.01 nucleotide substitutions per site.

The ITS analyses resulted in the formation of well-resolved/supported terminal subclades which led us to accept 80 *Ganoderma* taxa at species level in accordance with the criteria set and in conjunction with literature data available (Fig. 1b and Fig. 3). This number includes at least 21 hitherto unnamed (or not properly/fully identified) distinct phylogenetic entities for which tentative species names are hereby proposed. Six other terminal groups were phylogenetically delineated; however, since they did not fully conform to the criteria set for being recognised at the species-level, they were provisionally named in relation to their closest taxon by using the abbreviation “aff.”. Furthermore, two singletons presented distinct positions in the phylogeny of the genus but their status is ambiguous as discussed below.

**Figure 4.**
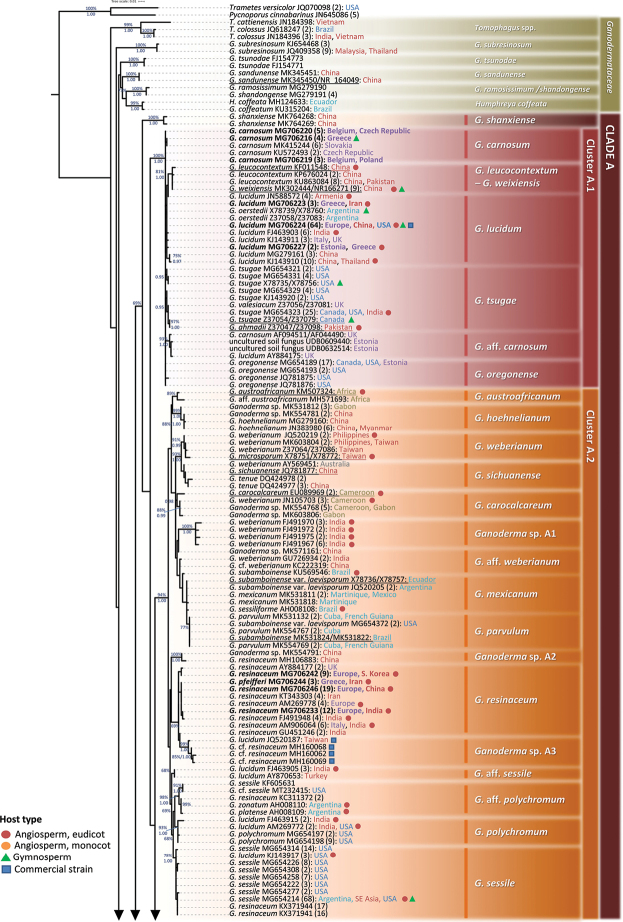
Detail from Fig. 3. Phylogenetic reconstruction of the genus *Ganoderma* inferred from ML analysis, based on ITS sequence data (main dataset, DS; Table 2) for Clade A, Clusters A.1 and A.2. ML bootstrap values (BS) ≥ 65% and Bayesian Posterior Probabilities (BPP) ≥ 0.95 are shown. Sequences names on the left appear as initially labelled and are followed by the respective GenBank/ENA/DDBJ or UNITE accession number, while the total number of identical entries corresponding to a particular sequence is placed in parentheses, followed by the type of host plant (legend for the coloured shapes is found at the lower left side of tree) and geographic origin of the respective material (the latter appears in different font colour depending on the continent of provenance; see also Table 1 and Suppl. material 1: Table S2). Species names on the right correspond to those inferred in this study evaluated in conjunction with literature data. Sequences generated in the present work appear in bold typeface, while underlined sequences are those originating from type material. Scale bar: 0.01 nucleotide substitutions per site.

**Figure 5.**
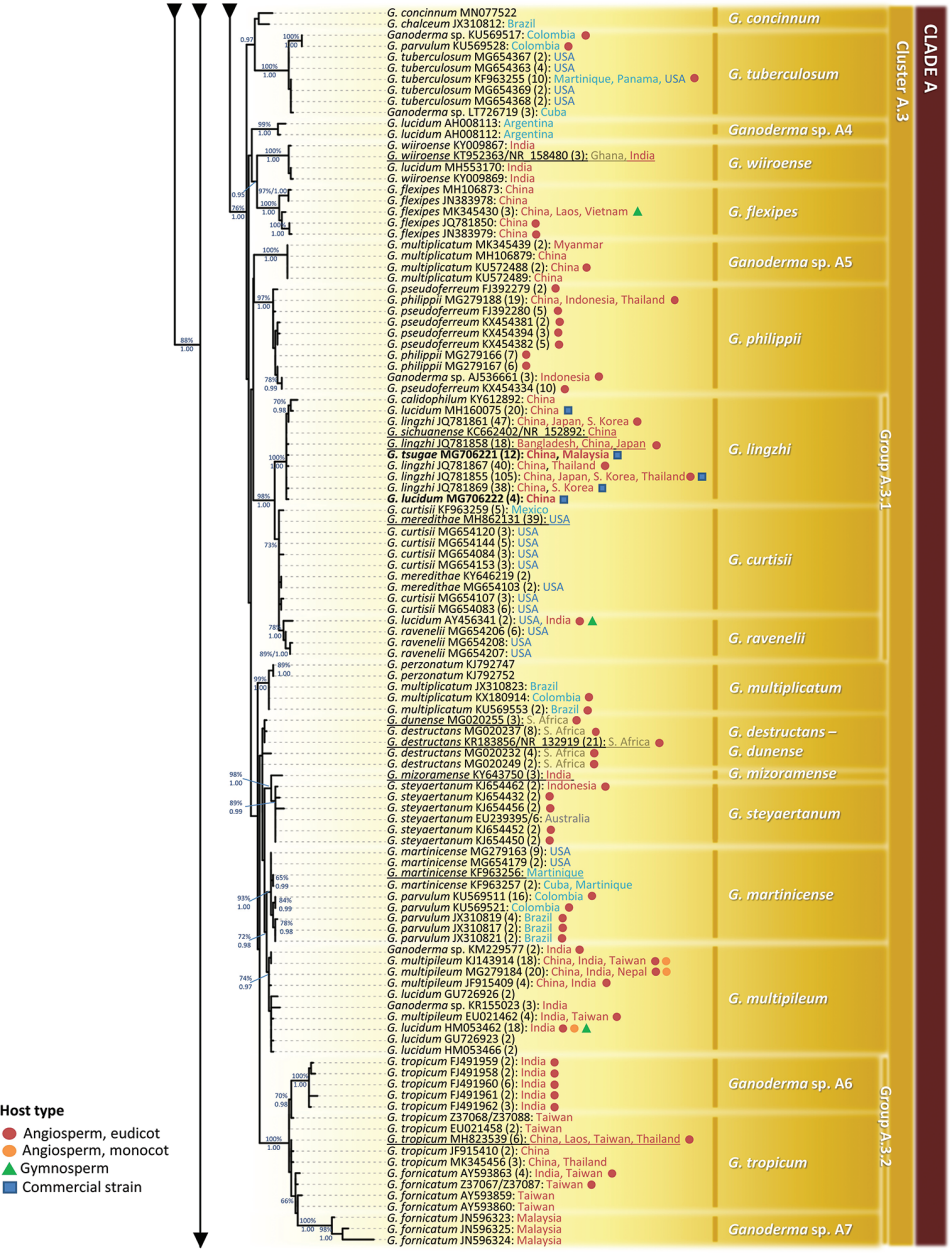
Detail from Fig. 3. Phylogenetic reconstruction of the genus *Ganoderma* inferred from ML analysis, based on ITS sequence data (main dataset, DS; Table 2) for Clade A, Cluster A.3. ML bootstrap values (BS) ≥ 65% and Bayesian Posterior Probabilities (BPP) ≥ 0.95 are shown. Sequences names on the left appear as initially labelled and are followed by the respective GenBank/ENA/DDBJ or UNITE accession number, while the total number of identical entries corresponding to a particular sequence is placed in parentheses, followed by the type of host plant (legend for the coloured shapes is found at the lower left side of the tree) and geographic origin of the respective material (the latter appears in different fonts colour depending on the continent of provenance; see also Table 1 and Suppl. material 1: Table S2). Species names on the right correspond to those inferred in this study evaluated in conjunction with literature data. Sequences generated in the present work appear in bold typeface, while underlined sequences are those originating from type material. Scale bar: 0.01 nucleotide substitutions per site.

**Figure 6.**
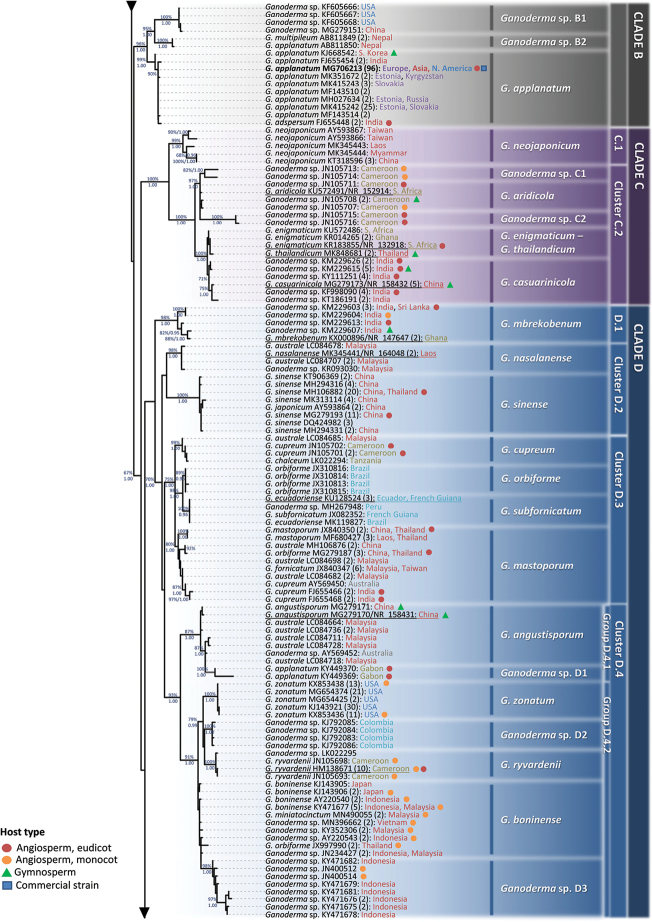
Detail from Fig. 3. Phylogenetic reconstruction of the genus *Ganoderma* inferred from ML analysis, based on ITS sequence data (main dataset, DS; Table 2) for Clades B, C and D. ML bootstrap values (BS) ≥ 65% and Bayesian Posterior Probabilities (BPP) ≥ 0.95 are shown. Sequences names on the left appear as initially labelled and are followed by the respective GenBank/ENA/DDBJ or UNITE accession number, while the total number of identical entries corresponding to a particular sequence is placed in parentheses, followed by the type of host plant (legend for the coloured shapes is found at the lower left side of the tree) and geographic origin of the respective material (the latter appears in different fonts colour depending on the continent of provenance; see also Table 1 and Suppl. material 1: Table S2). Species names on the right correspond to those inferred in this study evaluated in conjunction with literature data. Sequences generated in the present work appear in bold typeface, while underlined sequences are those originating from type material. Scale bar: 0.01 nucleotide substitutions per site.

**Figure 7.**
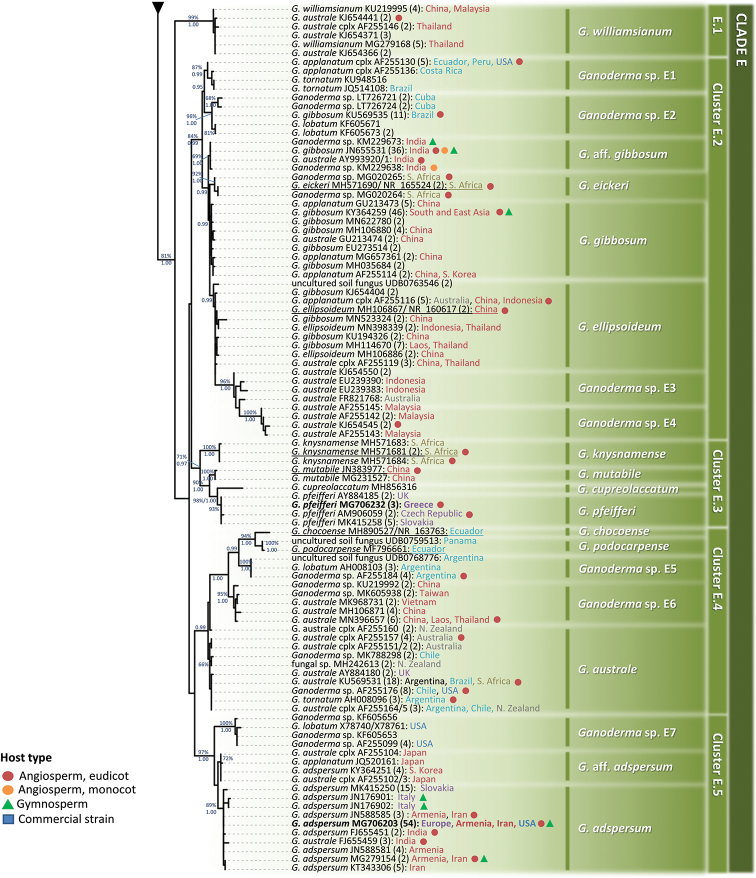
Detail from Fig. 3. Phylogenetic reconstruction of the genus *Ganoderma* inferred from ML analysis, based on ITS sequence data (main dataset, DS; Table 2) for Clade E. ML bootstrap values (BS) ≥ 65% and Bayesian Posterior Probabilities (BPP) ≥ 0.95 are shown. Sequences names on the left appear as initially labelled and are followed by the respective GenBank/ENA/DDBJ or UNITE accession number, while the total number of identical entries corresponding to a particular sequence is placed in parentheses, followed by the type of host plant (legend for the coloured shapes is found at the lower left side of tree) and geographic origin of the respective material (the latter appears in different fonts colour depending on the continent of provenance; see also Table 1 and Suppl. material 1: Table S2). Species names on the right correspond to those inferred in this study evaluated in conjunction with literature data. Sequences generated in the present work appear in bold typeface, while underlined sequences are those originating from type material. Scale bar: 0.01 nucleotide substitutions per site.

In all cases, ML and BI analyses provided almost identical tree topologies with minor differences and, thus, only the trees inferred from the ML analysis are presented. The genus *Ganoderma* exhibits a strongly-supported monophyly (BS: 90%, BPP: 1.00; Fig. 3). The ITS phylogeny reveals three major lineages, i.e. Clade A (72%; Figs 4, 5), Clade B (95%, 1.00; Fig. 6) and the Clades C, D and E (70%, 1.00; Figs 6, 7). Further resolution of phylogenetic origins and relationships amongst and within major Clades requires the use of additional molecular markers.

### Clade A

On the basis of ITS meta-analysis, Clade A is moderately supported only through ML analysis (69%; Fig. 4). It is hereby shown to represent the core of laccate species with a worldwide distribution (subgenus Ganoderma, sect. Ganoderma) and a large variation in morphological characteristics. Taxa nested in Clade A are generally characterised by laccate, usually reddish to dark-brown pilei, mostly annual (rarely biennial or even – allegedly – perennial), often stipitate or sessile to substipitate basidiomes, with elements of pileal crust possessing a regular palisade (hymenoderm) superficially covered or rarely embedded in a resinous-melanin matrix of varying thickness and a mostly pale/light-coloured context (Gilbertson and Ryvarden 1986; Moncalvo and Ryvarden 1997; Wasser et al. 2006).

Clade A includes 1927 entries distributed across 881 unique ITS sequence types of which 240 appear as ASVs representing 1414 entries in GenBank/ENA/DDBJ and UNITE. Clade A could be further divided into three well-supported Clusters (A.1, A.2 and A.3) and to the recently-introduced *G. shanxiense* L. Fan & H. Liu (Liu et al. 2019), i.e. a laccate, thin crust, dark brown context species represented by two singletons deriving from Chinese material (100%, 1.00; Fig. 4). In total, Clade A includes 28 well-supported phylogenetic species plus 14 distinct terminal clades corresponding to taxa not receiving adequate support (Figs 4, 5). Such open issues in delimiting *Ganoderma* are particularly evident in Cluster A.1, where low values of genetic distance are revealed amongst taxa and several of them are not supported – by ITS alone – as phylogenetically distinct or in Clusters A.2 and A.3 where species complexes (i.e. evolutionary-related populations with indiscrete boundaries amongst them) exist.

#### Clade A – Cluster A.1

**Figure 8.**
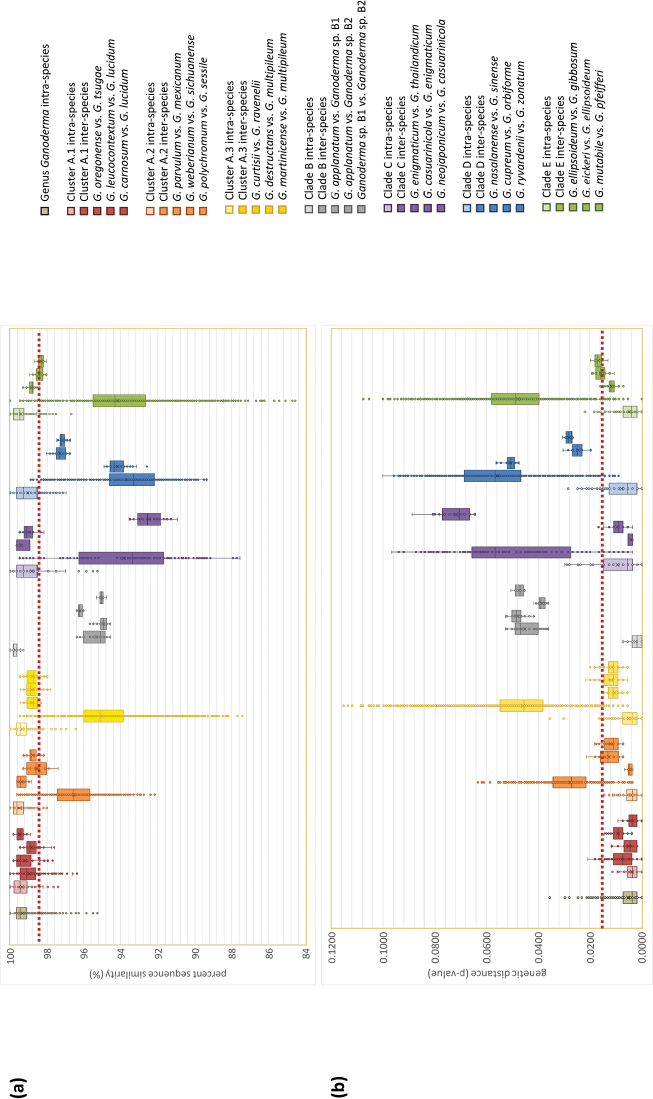
Box plots of **a**ITS sequence similarity (%) and **b** genetic distances (p-values) within (intra) and between (inter) *Ganoderma* species for each one of the main lineages (Clades/Clusters) of the genus, as well as pairwise comparisons between selected species. The size of each box represents 50% of the values, the black horizontal line within each box indicates the median, the ‘x’ represents the average value, the error bars represent interquartile ranges and circles indicate outliers. The red-dotted horizontal line, transversing the plots, represents the value levels accepted in this study for proposing new phylogenetic species.

In the context of this work, Cluster A.1 corresponds to a well-supported clade (100%, 1.00; Fig. 4) and comprises 36 sequences of which 23 are ASVs with 190 entries (Suppl. material 1: Table S2), while the expanded/detailed analysis includes a total of 297 entries (Suppl. material 3: Fig. S2a). Cluster A.1 is formed by material deriving from temperate regions of the Northern Hemisphere (except of two sequences originating from Argentinian specimens) on a large range of host plants under various names (Suppl. material 1: Table S2; Fig. 4). Interspecific genetic distances within Cluster A.1 are very low (i.e. 0.008 ± 0.004) in comparison to values calculated in other *Ganoderma* groups, which, in conjunction with the high sequence similarity values noted (98.96 ± 0.62%), are indicative of low divergence amongst taxa. Therefore, the criteria set in this study for phylogenetic species are not generally met for members of Cluster A.1 since a significant overlap exists between intraspecific and interspecific variability (absence of a barcoding gap; Fig. 8). Hence, in this particular case, ITS demonstrates poor species-level resolution and delimitation of taxa seems to be supported by multigene approaches only (Zhou et al. 2015; Loyd et al. 2018; Ye et al. 2019).

The major group of Cluster A.1 corresponds to *G. lucidum* (Curtis) P. Karst. (*G. lucidum* sensu stricto), which is represented by 10 sequences (or 97 entries) in the main dataset (Fig. 4). The majority of sequences which grouped within this species were found to be accurately identified (107 out of the 153 entries; Table 1); the highest number of erroneously-labelled sequences placed in *G. lucidum* were under the name “*G. tsugae*” (12 in total, six of which derived from China). The majority of *G. lucidum* sequences derive from material originating from Eurasia, growing mainly on hardwoods with some occasional reports of occurrence on conifers, for example, *Larix* and *Pinus* spp. (Suppl. material 1: Table S2). An exception was formed by nine entries from the US (California and Utah); they most probably correspond to introduced material (Loyd et al. 2018), as well as two sequences from Argentina under the name “*G. oerstedii*” (Moncalvo et al. 1995a). On the basis of the outcome of the present study, the latter were apparently misidentified and the respective material belongs to *G. lucidum*; its existence in Argentina could probably be attributed to human-mediated transfer. A distinct subclade within *G. lucidum* consists of 18 entries corresponding to three ASVs and three singletons (75%, 0.97; Fig. 4) from East Asian collections only (Park et al. 2012; Zhou et al. 2015; Xing et al. 2018); two conserved substitutions in both ITS spacers differentiate the respective sequences from the rest of *G. lucidum* (position 225 in ITS1 and position 378 in ITS2; Table 3; Suppl. material 2: Fig. S1).

*G. carnosum* Pat. was first described on *Abies* in southwest France (Pyrenees Mts.) and is distributed throughout Europe (Jahn et al. 1980, 1986; Mattock 2001; Ryvarden and Melo 2017); no reports exist of its occurrence in other continents. With respect to morphology, it resembles *G. lucidum* and the most prominent discriminating characters are the preference for conifers (*Abies* and *Pinus* spp.) and the blackish shiny upper surface when mature. The wider and highly-granulose (rougher) basidiospores, as well as the pore density, are also referred to as being of diagnostic value in taxonomic keys (Kotlaba and Pouzar 1993; Ryvarden and Gilbertson 1993; Ryvarden and Melo 2017). As a result of the ITS meta-analysis, 26 entries of *G. carnosum* originating from Europe (five generated in this study; Table 1 and Fig. 4) formed a group demonstrating high intraspecific sequence similarity. Most of them were accurately labelled (22 out of 32 *G. carnosum* entries available in public databases), while four were initially determined as “*G. lucidum*” (Table 1). The phylogenetically closest taxa to *G. carnosum* are *G. oregonense* and *G. lucidum* with which high values of sequence similarity were noted (99.68 ± 0.20% and 99.49 ± 0.24%, respectively).

Sequences deriving from two U.K. specimens (deposited as *G. carnosum* and *G. lucidum*) and from two environmental samples (Estonia, labelled as “uncultured soil fungus”) formed a distinct group (93%, 1.00; Fig. 4). It is provisionally named “G. aff. carnosum” due to the initial sequence labelling and European distribution and presents high sequence affinity with *G. oregonense* (98.87 ± 0.22%) and *G. carnosum* (98.84 ± 0.16%).

*G. oregonense* Murrill and *G. tsugae* Murrill are closely-associated taxa (Adaskaveg and Gilbertson 1986, 1988; Moncalvo 2000). They are mainly distinguished on the basis of basidiospores size and geographic distribution; the former is mainly found in western USA in temperate woods dominated by *Tsuga heterophylla*, *Pseudotsuga menziesii*, *Picea* spp. and *Abies* spp. However, the inclusion of environmental samples in this study expanded the known distribution of this taxon to Europe (Estonia, UDB0287378). *G. oregonense* is represented by four sequences corresponding to 21 entries (Fig. 4) in our analysis, while six singletons are also associated with this taxon and are included in the expanded dataset (Suppl. material 1: Table S2 and Suppl. material 3: Fig. S2a). On the other hand, *G. tsugae* is mainly recorded in eastern USA in temperate hemlock forests (Loyd et al. 2018), while it is also reported to occur in Canada (Adaskaveg and Gilbertson 1986, Ryvarden and Gilbertson 1993) and in Asia (Zhao 1989; Pegler and Yao 1996; Zhao and Zang 2000). Such reports were confirmed through the outcome of the present work (Table 1). *G. tsugae* is hereby represented by nine sequences corresponding to 41 entries in the databases examined (Fig. 4), while another 13 singletons were added in the expanded analysis (Suppl. material 1: Table S2 and Suppl. material 3: Fig. S2a). Most of them were found to be correctly identified (46 entries out of 54; Table 1; a sequence from the type specimen was also included, Z37054/Z37079). The ITS sequence similarity and genetic distance between *G. tsugae* and *G. oregonense* was found to be high and low, respectively (99.19 ± 0.62% and 0.005 ± 0.003, respectively), demonstrating that their distinct phylogenetic status cannot be evidenced through the use of ITS alone. In addition, the boxplot analysis revealed the absence of a barcoding gap between the two taxa (Fig. 8). However, application of a multigene approach permitted their delimitation (Loyd et al. 2018).

In the past, *G. tsugae* was occasionally reported to be conspecific with *G. carnosum* or *G. lucidum* on the basis of morphological observations (Donk 1974; Ryvarden and Melo 2017). The analysis performed in this work separates *G. tsugae* from *G. carnosum* and *G. lucidum*, which is in agreement with recent reports (Zhou et al. 2015; Loyd et al. 2018), although the interspecific sequence similarity values are high, i.e. 99.15 ± 0.58% and 98.91 ± 0.61%, respectively. In addition, a unique sequence, initially labelled “*G. carnosum*” (Z37057/Z37082; strain JAHN 1197-121, Germany), represents an authentic specimen of *G. atkinsonii* Jahn, Kotl. & Pouzar, which was later synonymised with *G. carnosum* (Jahn et al. 1986) and maintained as such by Moncalvo et al. (1995). The outcome of the present study shows that it forms part of *G. tsugae* (Suppl. material 1: Table S2 and Suppl. material 3: Fig. S2a).

Noteworthy cases pertain to sequences under the names of “*G. valesiacum*” and “*G. ahmadii*” which grouped within *G. tsugae* (Table 1; Fig. 4). *G. valesiacum* Boud. was described by Boudier and Fischer (1895) from a collection on *Larix* in Valais, Switzerland. The type specimen is almost destroyed; however, remains of the cutis are similar to the hymenodermiform anatomy presented by *G. lucidum*, but with smaller basidiospores possessing pronounced surface cambers and a “white, punk-like context” under a sometimes cracking-crust (Ryvarden and Gilbertson 1993; Ryvarden and Melo 2017). Three ITS sequences, labelled as “*G. valesiacum*”, are found in GenBank and were included in this work: (a) Z37056/Z37081 (strain CBS 282.33; UK), also used in previous studies (Moncalvo et al. 1995a; Hong and Yung 2004), is grouped within *G. tsugae* and presents sequence similarity values higher than 97.63% to other members of this taxon; (b) MG711807 (strain LE-BIN 2350; Russia, Altai Mts.) forms a clade within *G. lucidum* presenting an identical sequence with the most common ASV of this species (i.e. MG706224); (c) JQ520218 is grouped within *G. sessile* possessing the same sequence as the most common ASV of this species (i.e. MG654214; Suppl. material 1: Table S2); this particular sequence is erroneously associated with strain CBS 428.84 (USA), which had been earlier sequenced and correctly labelled as *G. tsugae* (X78735/X78756; Moncalvo et al. 1995b). Hence, molecular evidence from sequences labelled as “*G. valesiacum*” is in accordance with the views expressed in previous studies stating that such material is conspecific with either *G. lucidum* or *G. tsugae* on the basis of morphological features alone (Steyaert 1972; Stalpers 1978; Adaskaveg and Gilbertson 1986; Nunez and Ryvarden 2000; Wasser et al. 2006). Still, in the absence of a sequence from the holotype, it is not possible to draw definite conclusions on whether this name corresponds to a valid phylogenetic species. On the other hand, the only ITS sequence representing *G. ahmadii* Steyaert derives from the type material (strain FWP14329, Pakistan; Z37047/Z37098) and presents relatively-high sequence similarity values to members of the *G. tsugae* group (98.63 ± 0.36%). Therefore, on the basis of data available, the status of *G. ahmadii* remains ambiguous and additional specimens need to be studied.

In the frame of this study, four ASVs representing 20 entries formed a well-supported group (81%, 1.00; Fig. 4) including type material of two Chinese taxa recently described, i.e. *G. leucocontextum* T.H. Li, W.Q. Deng, Sheng H. Wu, D.M. Wang & H.P. Hu and *G. weixiensis* Ye et al. (Li et al. 2014; Ye et al. 2019). The two taxa presented high intraspecific sequence similarity indicative of their close geographic origin (i.e. 99.64 ± 0.28%); all studied specimens originated from China, Tibet and Nepal. Hence, ITS data alone could not discriminate the two taxa (Fig. 4) and the use of additional markers was necessary for establishing the latter species (Ye et al. 2019).

#### Clade A – Cluster A.2

Cluster A.2 (94%, 1.00; Fig. 4) is represented by 73 unique sequences; 47 are ASVs deriving from 309 entries in the databases (Table 1 and Suppl. material 1: Table S2). The expanded/detailed tree is formed by 263 sequences representing 517 entries in the databases (Suppl. material 1: Table S2 and Suppl. material 4: Fig. S2b).

A major subclade is formed by specimens collected in southeast Asia and Australia growing mostly on angiosperms (93%, 1.00 and 72%, 1.00, Fig. 4 and Suppl. material 4: Fig. S2b, respectively). The sequences had been deposited under different taxonomic names, i.e. *G. weberianum* (Bres. & Henn. ex Sacc.) Steyaert, *G. sichuanense* J.D. Zhao & X.Q. Zhang, *G. tenue* J.D. Zhao, L.W. Hsu & X.Q. Zhang, *G. lucidum*, *G. microsporum* R.S. Hseu and *Ganoderma* sp. and present rather low heterogeneity as evidenced by the values of sequence similarity and genetic distance obtained (98.87 ± 0.62% and 0.010 ± 0.005, respectively). We consider that this particular subclade is related to *G. weberianum* sensu Steyaert (Moncalvo 2000) and includes at least two not adequately (by ITS alone) resolved groups. One of them corresponds to *G. sichuanense* and consists of 19 entries originally identified as *G. sichuanense* (9), *G. tenue* (2), “*G. weberianum*” (4), “*G. lucidum*” (2), *Ganoderma* sp. (1) and “uncultured soil fungus” (1), which derive from specimens collected in southeast Asia and Australia on angiosperms and gymnosperms (Table 1, Suppl. material 1: Table S2). This taxon demonstrates high sequence similarity (99.40 ± 0.22%) and low genetic distance values (0.006 ± 0.003). Two sequences assigned to *G. sichuanense* (including the holotype: HMAS 42798, JQ781877; Cao et al. 2012) group with sequences identified as *G. tenue* (Figure 3 and Suppl. material 4: Figure S2b); unfortunately, no sequence is available from the type material of *G. tenue* (Zhao et al. 1979a), which makes it difficult to comment on its relationships to associated taxa. The other group is composed of 12 sequences [originally identified as *G. weberianum* (9) and *G. microsporum* (corresponding to the type, RSH 0821; Moncalvo et al. 1995b)] which are placed together (91%, 0.99; Fig. 4) and demonstrate high intraspecific sequence similarity (99.16 ± 0.48%). On the basis of the aforementioned findings, we consider that they form part of *G. weberianum* sensu stricto (originally described from Samoa; Steyaert 1972). Moreover, although it is not possible to draw any definite conclusions about the status of *G. microsporum*, we tend to agree with the view expressed by Smith and Sivasithamparam (2000) that it is a subspecific entity within *G. weberianum*. The two groups corresponding to *G. sichuanense* and *G. weberianum* demonstrate interspecific ITS sequence similarity and genetic distance values (Fig. 8) which do not support the existence of two distinct phylospecies on the basis of the criteria hereby set.

*G. hoehnelianum* Bres. forms a monophyletic group in Cluster A.2 (99%, 1.00; Table 1, Fig. 4) and includes 12 entries deriving from southeast Asian (China, Myanmar) and African (Gabon) material; sequences of the two origins are separated into two subgroups (Fig. 4), but they exhibit low variability (sequence similarity: 99.63 ± 0.23%). On the other hand, *G. austroafricanum* Coetzee, M.J. Wingf., Marinc., Blanchette is also a well-supported species (87%, 0.99; Suppl. material 4: Fig. S2b) represented by two singletons, including the type specimen from South Africa. Moreover, *G. carocalcareum* Douanla-Meli forms a subclade (67%, 0.99; Suppl. material 4: Fig. S2b) consisting of 11 sequences which originate from material collected in Africa (Table 1); two are under this name (including the type specimen), while the rest are labelled either as “*G. weberianum*” (3) or as *Ganoderma* sp. (1) and form a well-supported subgroup within the clade (88%, 0.99; Fig. 4). However, sequence similarity and genetic distance values (98.61 ± 0.36% and 0.013 ± 0.002, respectively) do not permit their dinctinction. The entry MK603806 (MUCL 49272) represents the “C2.2” clade in the study of Cabarroi-Hernández et al. (2019) which is composed of several MUCL specimens from Cameroon and Gabon. Genetic distance and sequence similarity values support the distinct status of *G. carocalcareum* from *G. weberianum* (0.022 ± 0.006 and 97.50±0.78%, respectively).

A new phylogenetic species within Cluster A.2 is hereby proposed and is provisionally named “*Ganoderma* sp. A1” (corresponding to the UNITE DOIs SH1740420.08FU, SH1740444.08FU and SH1740445.08FU); its monophyly is strongly supported in both trees (100%, 1.00; Fig. 4 and Suppl. material 4: Fig. S2b). It is represented by eight unique sequences (i.e. four ASVs and four singletons; Suppl. material 1: Table S2 and Suppl. material 4: Fig. S2b) corresponding to 17 entries. On the basis of available information, all sequences derive from specimens initially identified as “*G. weberianum*” originating from India on a large range of eudicots (Suppl. material 1: Table S2). *Ganoderma* sp. A1 presents relatively-low intraspecific genetic distances (0.010 ± 0.003) and high sequence similarity (99.01 ± 0.18%).

Other four entries of dubious identity derive from material originating from Asia (China and India) labelled as *Ganoderma* sp., “*G. weberianum*” (2) and “Ganoderma cf. weberianum”, as well as from Brazil under the name “*G. subamboinense*” (Table 1 and Suppl. material 1: Table S2) and exhibit low genetic distance and high sequence similarity (0.006 ± 0.003 and 99.18 ± 0.43%, respectively). They form part of the same larger subclade together with *Ganoderma* sp. A1, *G. mexicanum* and *G. parvulum* (Fig. 4); however, they do not retain the same position in the expanded tree (Suppl. material 4: Fig. S2b). For the purposes of this work, we provisionally maintain them as Ganoderma aff. weberianum (corresponding to the UNITE DOI SH1723064.08FU) since it is not possible to determine their exact identity from the data available.

Another major subclade consisting of material originating from the Neotropics is formed by a total of 17 entries. Four of them are singletons, while the other 13 are grouped in six ASVs (Suppl. material 1: Table S2; Fig. 4); the expanded/detailed tree includes 38 entries corresponding to 31 sequences (Suppl. material 4: Fig. S2b). They were deposited under several names: *G. parvulum* Murrill (10 sequences), G. subamboinense var. laevisporum Bazzalo & J.E. Wright (7, including type material), *G. mexicanum* Pat. (6), *G. subamboinense* (Henn.) Bazzalo & J.E. Wright ex Moncalvo and Ryvarden (3, including type material), “*G. tuberculosum*” (1), “*G. weberianum*” (5), *G. sessiliforme* Murrill (1), *G. stipitatum* (Murrill) Murrill (1) and *Ganoderma* sp. (4). The outcome of this work shows that this material forms part of two terminal groups corresponding to *G. mexicanum* and *G. parvulum* (Fig. 4) in accordance with the findings of a recent study by Cabarroi-Hernández et al. (2019). Hence, the former is selected as the earliest valid name to accommodate specimens also reported as “*G. sessiliforme*” and “G. subamboinense var. laevisporum” from Argentina, Brazil, Martinique, Mexico and USA, while the latter for material also labelled as “*G. subamboinense*”, “G. subamboinense var. subamboinense” and “*G. stipitatum*” from Brazil, Colombia, Costa Rica, Cuba, French Guiana and USA (77%; Fig. 4). Such a separation is in accordance with morphological observations made by Ryvarden (2000; 2004) and Cabarroi-Hernández et al. (2019). However, the support that this group of taxa receives by ITS data is not adequate (Suppl. material 4: Fig. S2b) and the respective interspecific sequence similarity and genetic distance values (Fig. 8) do not separate them on the basis of the criteria hereby set.

Another well-supported terminal clade (100%, 1.00; Fig. 4, Suppl. material 4: Fig. S2b) is formed by two entries labelled as “*G. resinaceum*” and *Ganoderma* sp. deriving from China. It is provisionally named *Ganoderma* sp. A2 (corresponding to the UNITE DOI SH2762559.08FU) since it complies with the criteria set in this study and it is hence considered as a new phylospecies. The genetic distance and sequence similarity values versus the most closely-related species (i.e. *G. resinaceum*) are 0.028 ± 0.003 and 97.52 ± 0.23%, respectively.

*G. resinaceum* Boud. is represented by 10 ASVs and 65 entries (Fig. 4), while the expanded/detailed tree includes 65 sequences corresponding to 126 entries (Suppl. material 4: Fig. S2b). Intraspecific genetic distance and sequence similarity values are well within the respective ranges observed for taxa of Clade A (i.e. 0.005 ± 0.003 and 99.46 ± 0.31%, respectively). This species appears to be very common throughout Europe (type locality), but it also occurs in Asia (e.g. China, India, Iran, Iraq, South Korea and Turkey) and Africa (Egypt, South Africa and Tunisia), being reported on a wide range of angiosperms (Suppl. material 1: Table S2). On the other hand, there is great controversy regarding the existence of *G. resinaceum* in the Americas. Its occurrence was reported by several authors in the past, for example, in Mexico (Torres-Torres et al. 2015), Brazil (Loguercio-Leite et al. 2005) and Argentina (Bazzalo and Wright 1982), although it was admittedly confused with other species names (e.g. *G. oerstedii*, *G. parvulum* or *G. subincrustatum*; de Lima Júnior et al. 2014; Torres-Torres et al. 2015). In addition, it has been synonymised with Neotropical taxa, such as *G. chaffangeonii* Pat. (type locality Venezuela; Steyaert 1980; Torres-Torres et al. 2012) and *G. praelongum* Murrill (type locality Cuba). However, no report of its alleged existence in America is so far supported by DNA data. Moreover, in the frame of this study, none of the sequences examined and confirmed to be *G. resinaceum* represents material originating from this continent. Therefore and to the best of our knowledge, the presence of *G. resinaceum* in the Americas cannot be confirmed by molecular evidence and its distribution seems to be restricted to the Old World.

Another new phylogenetic species is hereby proposed, provisionally named “*Ganoderma* sp. A3” (corresponding to the UNITE DOI SH1723084.08FU). It is strongly supported in both trees (100%, 1.00 and 99%, 1.00 in Fig. 4 and Suppl. material 4: Fig. S2b, respectively) and is represented by twelve singletons under the names “*G. resinaceum*” (5), “Ganoderma cf. resinaceum” (3) *Ganoderma* sp. (2), “*G. lucidum*” (1) and “uncultured *Ganoderma*” (1), all deriving from material from east Asia (Table 1 and Suppl. material 1: Table S2). This distinct taxon (intraspecific genetic distance: 0.007 ± 0.002; sequence similarity: 99.22 ± 0.37%) presents at least six different conserved positions in ITS1 and in ITS2 when compared to *G. resinaceum* (Suppl. material 3: Fig. S1), which is the closest phylogenetic relative (genetic distance: 0.024 ± 0.005; sequence similarity: 97.59 ± 0.51%).

*G. sessile* Murrill is a well-supported (78%, 1.00; Fig. 4) and highly-represented species in terms of deposited sequences (227; Suppl. material 1: Table S2) showing high intraspecific sequence similarity (99.73 ± 0.14%). Many of the sequences were initially labelled as “*G. resinaceum*” (60 entries) and “*G. lucidum*” (9). Most *G. sessile* sequences derived from specimens collected in the USA, while the rest originate from Asia (including the Caucasus area) and one from Argentina (JQ520199; originally labelled as *G. resinaceum*), possibly as an outcome of human-mediated transfer.

A closely-related and well-supported (93%, 1.00; Fig. 4) group is formed by sequences belonging to *G. polychromum* (Copel.) Murrill. This species is represented by 19 entries deriving from material collected in either western USA (California, Washington and Oregon; associated with hardwood) or India; some of them were deposited under the names “*G. lucidum*” (6) or “*G. sessile*” (2) (Table 1). However, pairwise comparisons of *G. polychromum* sequences with those belonging to closely-positioned taxa revealed rather low genetic distance and high sequence similarity values (for example, vs. *G. sessile*: 0.012 ± 0.004, 98.76 ± 0.32%, respectively).

A distinct phylogenetic group (98%, 1.00; Fig. 4), sister to *G. polychromum*, consists of seven sequences presenting high intraspecific similarity, i.e. 98.88 ± 0.64%; the respective material derived the Americas and it appears under the names “*G. platense*”, “*G. resinaceum*”, “*G. sessile*” “G. cf. sessile” and “*G. zonatum*” (Table 1). Amongst those, the only one that could possibly have been correctly identified corresponds to *G. platense* Speg. because the correct topologies of *G. resinaceum* and *G. sessile* are found elsewhere within Cluster A.2, while *G. zonatum* forms part of Clade D (Fig. 4). However, since very limited information is available concerning this particular specimen (*G. platense* isolate BAFC384, AH008109; Gottlieb et al. 2000), we prefer not to draw any conclusions concerning the name of this terminal subclade. In addition, because of the close affinity it presents with *G. polychromum* (genetic distance: 0.014 ± 0.004, sequence similarity: 98.10 ± 0.42%), it does not abide (albeit marginally) with the criteria we set in order to be characterised as a distinct phylogenetic species; therefore, it is provisionally named “G. aff. polychromum” (corresponding to the UNITE DOIs SH1723162.08FU and SH1723226.08FU).

Another terminal group, although not adequately supported in any of the trees constructed, forms a sister clade to *G. sessile*/*G. polychromum* (Fig. 4, Suppl. material 4: Fig. S2b) and includes four sequences originally identified as “*G. lucidum*” deriving from India (3) and Turkey on eudicots (i.e. *Cassia fistula* and *Tamarindus indica*). It presents low genetic distance and high sequence similarity values in pairwise comparisons to *G. sessile* (0.014 ± 0.002 and 97.34 ± 0.16%); therefore, its status is dubious and it is provisionally named “G. aff. sessile” (corresponding to the UNITE DOI SH1723202.08FU).

#### Clade A – Cluster A.3

Cluster A.3 (76%, 1.00; Fig. 5) includes material originating from south and east Asia, tropical Africa, Australia and America (no occurrence in Europe) growing mostly on eudicot hosts (Suppl. material 5: Fig. S2c). It is composed of a total of 1385 entries, 889 of which are grouped in 145 ASVs (Suppl. material 1: Table S2) and comprises 19 terminal groups, 14 of which are well-supported in the present analysis.

*G. tuberculosum* Murrill is strongly supported in the generated trees (100%, 1.00; Fig. 5 and Suppl. material 5: Fig. S2c) and is represented by 36 sequences, 23 of which are grouped in six ASVs (Suppl. material 1: Table S2 and Suppl. material 5: Fig. S2c). Most of the respective material was correctly labelled (28 entries) and collections derive entirely from the Neotropics, i.e. southeast USA, Cuba, Martinique, Panama, Colombia and Brazil (Table 1). Murrill’s type material of *G. tuberculosum* from British Honduras was very similar to basidiomes from Martinique examined by Welti and Courtecuisse (2010), thus confirming the wider distribution of this species in the Caribbean as indicated by our analysis. It is worth noting that sequences from Colombia (2) and Texas (1) form a distinct strongly-supported subgroup (99%, 1.00; Suppl. material 5: Fig. S2c) presenting conserved differences in ITS sequences (three to five positions in ITS1 and three in ITS2; Table 3 and Suppl. material 3: Fig. S1); however, their similarity values to the other *G. tuberculosum* sequences are high (i.e. 99.27 ± 0.58%) and do not seem to support a distinct species status. On another issue, recent studies reported the presence of *G. oerstedii* (Fr.) Murrill in Mexico, Costa Rica and Honduras (amongst other areas in the Neotropics) and referred to diagnostic characters, such as “the color of the basidiomata, context with resinous bands, cuticle cells with protuberances and/or branches and partially anastomosed basidiospore pillars” (Mendoza et al. 2011; Torres-Torres et al. 2015). Both Ryvarden (2000) and Torres-Torres et al. (2015) considered *G. tuberculosum* as a synonym of *G. oerstedii*, but this view cannot be supported (but neither contradicted) by the findings of the present study, since sequence data from the holotype or of a correctly-designated epitype of *G. oerstedii* are missing.

Furthermore, one sequence (JX310812) labelled as “*G. chalceum*” originating from Brazilian material and one sequence under the name *G. concinnum* Ryvarden (possibly of South American origin) form a terminal subclade which nested close to *G. tuberculosum* (0.97; Fig. 5). The two sequences present relatively-low sequence similarity (98.01%) and rather high genetic distance (0.020) and their conspecificity is uncertain. Since *G. chalceum* (Cooke) Steyaert is a species described on the basis of material originating from Africa (type specimen from Sierra Leone), southeast Asia and Oceania (Steyaert 1967) and because the only other ITS sequence available under this name is grouped in Clade D of the present study (together with other entries originating from Africa), the real identity of JX310812 remains ambiguous. Therefore, we prefer to use the name *G. concinnum* for describing this particular group.

Two sister subclades (0.99, Suppl. material 5: Fig. S2c) correspond to *G. flexipes* Pat. and *G. wiiroense* E.C. Otto, Blanchette, C.W. Barnes & Held. Both are strongly supported in the generated trees (1.00%, 1.00; Fig. 5 and Suppl. material 5: Fig. S2c). The former consists of seven sequences deriving from material collected on both angiosperms and gymnosperms in tropical Asia (China, Laos and Vietnam; Suppl. material 1: Table S2), presenting relatively-high intraspecies genetic distance (0.011 ± 0.005) which is in agreement with the formation of two well-supported terminal subgroups. The latter also constitutes a monophyletic species represented by 14 entries originating from Ghana (including the type specimen), Senegal and India.

Two new well-supported monophyletic species, provisionally named as “*Ganoderma* sp. A4” (no UNITE DOI available) and “*Ganoderma* sp. A5” (corresponding to the UNITE DOI SH1723120.08FU) are revealed in this study. *Ganoderma* sp. A4 is represented by two sequences deriving from Argentinian material which were originally identified as “*G. lucidum*” (100%, 1.00; Fig. 5 and Suppl. material 5: Fig. S2c). *Ganoderma* sp. A5 comprises seven sequences labelled as “*G. multiplicatum*”, all originating from Asian specimens (100%, 1.00; Fig. 5 and Suppl. material 5: Fig. S2c); this name is apparently misapplied since *G. multiplicatum* occurs in the Neotropics and corresponds to another, rather distant, terminal group. Both *Ganoderma* sp. A3 and *Ganoderma* sp. A4 present high intraspecific sequence similarities (> 98.90%), whereas their respective interspecific values are indicative of their distinct status (93.64 ± 0.29% and 0.058 ± 0.03).

*G. philippii* (Bres. & Henn. ex Sacc.) Bres. constitutes a well-supported species (97%, 1.00; Fig. 5) represented by 102 sequences, of which 69 are grouped in 13 ASVs (Suppl. material 1: Table S2 and Suppl. material 5: Fig. S2c). The respective material originates from south and southeast Asia (China, Indonesia, Malaysia and Thailand) and is mainly deposited under the synonym *G. pseudoferreum* (Wakef.) Overeem & B.A. Steinm. Intraspecific genetic distance and sequence similarity values lie well within the ranges observed for species of Cluster A (i.e. 0.005 ± 0.003, max. 0.009; 99.44 ± 0.25%, min. 98.90%).

Three species, i.e. *G. lingzhi* S.H. Wu, Y. Cao & Y.C. Dai, *G. ravenelii* Steyaert and *G. curtisii* (Berk.) Murrill, form a strongly-supported group (A.3.1; 98%, 1.00; Fig. 5). Amongst them, *G. lingzhi* is distributed in south and east Asia on a wide range of angiosperms (Suppl. material 1: Table S2) and corresponds to a well-supported subclade (100%, 1.00; Fig. 5). It is represented by the largest number of entries for any given species in the present study (615, ca. 16% of the total generic entries; Table 1); 436 of them are grouped in 41 ASVs, while the dominant ASV is represented by 105 identical ITS entries (Suppl. material 1: Tables S2 and S4 and Suppl. material 5: Fig. S2c). Only 54% of these entries were deposited as *G. lingzhi* (including the holotype, JQ781858), while many of them were originally labelled as “*G. lucidum*” (206) since collections of the traditional medicinal fungus ‘Lingzhi’ were mainly identified as such for many years. Cao et al. (2012) finally proposed the name *G. lingzhi* for this fungus and thereby marked the onset of a debate concerning the real identity (correct name) of the fungus, i.e. *G. sichuanense* or *G. lingzhi*. Wang et al. (2012) and Yao et al. (2013) supported the former view by sequencing a so-called epitype (voucher HMAS252081; KC662402) of the original material. However, the epitypification of *G. sichuanense* did not comply with the International Code of Nomenclature for algae, fungi and plants (Zhou et al. 2015). Hence, this particular collection of “*G. sichuanense*” corresponds to *G. lingzhi* as was recently explained (Dai et al. 2017). On the other hand, the holotype represents the true *G. sichuanense* which is not related to *G. lingzhi* (Cao et al. 2012; Liao et al. 2015; this work) and is positioned in Cluster A.2. It is of interest that, although a large number of *G. lingzhi* sequences were included in this meta-analysis, intraspecific genetic divergence was low (0.004 ± 0.003, 99.42 ± 0.32%).

*G. curtisii* (70%, 1.00; Suppl. material 5: Fig. S2c) is closely related to *G. lingzhi* in agreement with previous phylogenies (Zhou et al. 2015; Thawthong et al. 2017). It occurs in North America – being widespread in the eastern parts of the USA – primarily on angiosperms. In the context of the present study, 142 entries were grouped within this species; 89 of them correspond to 20 ASVs (Suppl. material 1: Table S2 and Suppl. material 5: Fig. S2c). The majority (124) were deposited with the correct name in databases, while nine were labelled as *G. meredithae* Adask. & Gilb. (including the type material; MH862131) and three as G. curtisii f. sp. meredithae. However, *G. meredithae* is considered to be a synonym of *G. curtisii* (Moncalvo 2000) as was later confirmed (Loyd et al. 2018). *G. curtisii* demonstrates low levels of variability evidenced by its intraspecific genetic distance and sequence similarity values (0.003 ± 0.001 and 99.49 ± 0.22%, respectively). On the other hand, *G. ravenelii* presents an overlapping geographic distribution with *G. curtisii*, for example, in south and east USA (Florida and North Carolina; Suppl. material 1: Table S2) and it forms a well-supported terminal subgroup (78%, 1.00; Fig. 5) being composed of 12 sequences from specimens isolated on both angiosperms and gymnosperms (Suppl. material 5: Fig. S2c). However, the outcome of the present work shows that these two taxa exhibit high affinity (genetic distance: 0.011 ± 0.003; sequence similarity: 98.93 ± 0.27%) and no clear ITS barcoding gap is evident between them (Fig. 8); their delineation is adequately supported only through the application of a multigene approach (Loyd et al. 2018).

Six other closely-related species are found in Cluster A.3 (i.e. *G. multiplicatum*, *G. destructans*, *G. steyaertanum*, *G. mizoramense*, *G. martinicense* and *G. multipileum*) (Fig. 5, Suppl. material 5: Fig. S2c). Amongst them, *G. multiplicatum* (Mont.) Pat. was originally described from French Guiana (Moncalvo and Ryvarden 1997) and its presence was evidenced in several other areas of South America (Ryvarden 2004; de Lima Júnior et al. 2014; Bolaños et al. 2016; Torres-Torres et al. 2012). Pertinent material, analysed in this study, formed an external subclade with strong support (99%, 1.00; Fig. 5); genetic distance and sequence similarity values (0.002 ± 0.001 and 99.54 ± 0.25%, respectively) are indicative of low intraspecific variability. All seventeen sequences derived from specimens collected in the Neotropics (Mexico, Brazil and Colombia; Table 1); seven of them were initially labelled as “*G. perzonatum*”. However, the exact status of *G. perzonatum* Murrill remains ambiguous; it was originally described from Cuba and its morphological features associate it with *G. parvulum* (Cluster A.2 in this work) as reported by Moncalvo and Ryvarden (1997) and Cabarroi-Hernández et al. (2019). According to the latter study, “*G. perzonatum* could represent another closely related taxon in the vicinity of *G. mexicanum* / *G. parvulum*”; hence, examination of additional specimens is needed to arrive at robust conclusions. *G. multiplicatum* was also recorded in Asia and Africa (Steyaert 1980; Zhao 1989; Wang and Wu 2007; Bhosle et al. 2010). To the best of our knowledge, the occurrence of this species in a region other than the Neotropics was never evidenced through the use of molecular data; the four sequences from China under this name correspond to a distinct phylospecies nested in Cluster A.3, i.e. *Ganoderma* sp. A5 (Table 1, Fig. 5) as previously explained. Therefore, the Asian material that groups in *Ganoderma* sp. A5 does not seem to be associated with the name *G. multiplicatum* in contrast to what was recently reported (Hapuarachchi et al. 2019).

In this study, *G. destructans* M.P.A. Coetzee, Marinc., M.J. Wingf. represented by 39 entries (including the type material; Table 1) is grouped together with *G. dunense* Tchotet, Rajchenb. & Jol. Roux; the latter consists of three entries (one from the type material) which are identical to *G. destructans* sequences (Table 1, Fig. 5 and Suppl. material 5: Fig. S2c). Specimens of both taxa originate from South Africa on eudicots (Coetzee et al. 2015, Tchotet Tchoumi et al. 2018). ITS sequence similarity and genetic distance values for material under these two names are indicative of the existence of a single species (0.006 ± 0.002; 99.73 ± 0.10%); hence, the distinction of these two entities cannot be supported by the use of this marker alone. However, *G. destructans* and *G. dunense* are distinguished following the outcome of a multigene analysis (Tchotet Tchoumi et al. 2018).

*G. steyaertanum* B.J. Smith & Sivasith. forms a well-supported group (89%, 0.99; Fig. 5) which is composed of 39 entries from specimens growing on eudicots in Indonesia and Australia (Table 1). *G. steyaertanum* was proposed as the correct name for the erroneously-labelled “*G. lucidum*” specimens reported to occur in this particular region (Smith and Sivasithamparam 2003). However, none of the sequences hereby examined was originally deposited as “*G. lucidum*”; instead, entries were labelled either as *G. steyaertanum* (34) or as G. aff. steyaertanum (3). This species forms a sister clade (98%, 1.00; Fig. 5) with *G. mizoramense* Zothanzama, Blanchette, Held, C.W. Barnes represented by only three identical sequences, all deriving from India (Suppl. material 1: Table S2 and Suppl. material 5: Fig. S2c). The two taxa appear closely related as evidenced by their genetic distance and sequence similarity values (0.016 ± 0.005 and 98.46 ± 0.31%, respectively), while the respective sequences differ at six conserved positions (Suppl. material 3: Fig. S1); furthermore, three conserved nucleotides in ITS2 are common to both and separate them from other related taxa of Cluster A.3. The very limited representation of *G. mizoramense* does not allow any definite conclusions regarding its taxonomic status.

*G. martinicense* Welti & Courtec. is sister to *G. multipileum* (72%, 0.98; Fig. 5), the two taxa being closely related (98.76 ± 0.36% and 0.012 ± 0.003) with no barcoding gap existing between them (Fig. 8). The former (93%, 1.00; Fig. 5) consists of 49 entries originating from specimens collected in USA, Mexico, Cuba, Martinique, Colombia, Brazil and Argentina which were deposited under various names, i.e. *G. martinicense* (18 entries, including type material), “*G. parvulum*” (24), “*G. perzonatum*” (2), “*G. tuberculosum*” (1), “*G. oerstedii*” (1) and “*G. lucidum*” (1) (Table 1, Suppl. material 5: Fig. S2c). Although at least three (not adequately supported) groups are evident in the expanded tree (which corresponds to different geographic origins, i.e. Colombia, Brazil/Argentina and Cuba/Martinique; Suppl. material 5: Fig. S2c), ‘intergroup’ values for ITS sequence divergence do not justify the existence of more than one phylogenetic species. Therefore and until more information becomes available, we prefer to maintain them all under *G. martinicense*. The name “*G. parvulum*” corresponds to a taxon forming part of Cluster A.2, as previously discussed.

*G. multipileum* Hou is phylogenetically supported (74%, 0.97; Fig. 5) and hereby represented by 243 entries; 130 entries are grouped in 29 ASVs (Suppl. material 1: Tables S2, S4). The respective collections originate from south and east Asia on a wide range of eudicot (mostly) and monocot or gymnosperm hosts, including also commercial strains (Suppl. material 5: Fig. S2c). The majority of sequences were labelled either as “*G. lucidum*” (105) or as *Ganoderma* sp. (112) and only 22 were identified as *G. multipileum*.

A second group (A.3.2; 100%, 1.00; Fig. 5) within Cluster A.3 includes sequences stemming from south and ast Asia. *G. tropicum* (Jungh.) Bres. is a species described from Indonesia (Java) and is widely distributed across subtropical and tropical Asia (Steyaert 1972; Moncalvo and Ryvarden 1997; Luangharn et al. 2019a). According to Corner (1983), this is a complex of pantropical occurrence, comprising many taxonomic varieties characterised by strongly echinulate basidiospores. However, pertinent sequenced material derives only from Asia. Hence, 15 entries, corresponding to specimens from China, Laos, Taiwan and Thailand, were originally identified as *G. tropicum* (type material included; Table 1). Moreover, 12 entries, labelled as “*G. fornicatum*” from specimens collected in India and Taiwan, presented genetic distances and sequence similarity indicative of a high affinity to *G. tropicum* sequences (i.e. 0.010 ± 0.004; 98.93 ± 0.63%, respectively). The type material of *G. fornicatum* (Fr.) Pat. originates from Brazil, but cannot be located and is most probably lost (Ryvarden 1991; Moncalvo and Ryvarden 1997). Furthermore, no recent information exists on the presence of this species in the Neotropics (Wang et al. 2014) and no sequence is available from specimens deriving from this particular region. In contrast, sequences/material under this name originates from Asia only (Imazeki 1939; Zhao and Zhang 2000; Wang and Wu 2008). Last, according to Mycobank, the current name for *G. fornicatum* is *G. orbiforme* (Fr.) Ryvarden. However, the latter is positioned in Clade D of the present analysis and is composed of entries originating solely from Brazil (Fig. 5). In view of the above, we maintain those particular entries identified as “*G. fornicatum*” under *G. tropicum*.

A strongly-supported terminal subclade in Group A.3.2 (100%, 1.00; Fig. 5) consists of 15 sequences (five ASVs) initially labelled “*G. tropicum*”, originating from Indian specimens obtained from various angiosperms, for example, *Ficus benghalensis*, *Terminalia bellirica*, *Delonix regia* and *Cassia fistula* (Arulpandi and Kalaichelvan, unpublished results). It is considered as a new phylogenetic species, hereby referred to as “*Ganoderma* sp. A6” (corresponding to the UNITE DOI SH1723103.08FU) since it is clearly separated from *G. tropicum* by presenting interspecific genetic distance and sequence similarity values of 0.026 ± 0.005 and 97.24 ± 0.68%, respectively.

Furthermore, three singletons from Malaysian material (in this particular case, geographic origin is inferred from the title of the study which appears on the respective GenBank records), initially identified as “*G. fornicatum*”, form another distinct well-supported group (100%, 1.00; Fig. 5). This is also considered as a new phylogenetic species provisionally named “*Ganoderma* sp. A7” (corresponding to the UNITE DOI SH1723183.08FU) and is well distinguished from *G. tropicum* which is the closest taxon amongst those examined (sequence similarity: 94.64 ± 1.56%; genetic distance: 0.046 ± 0.015).

### Clade B

Clade B (96%, 1.00; Fig. 6) includes 16 unique sequences corresponding to 431 individual entries (Table 1). It accommodates three species including the non-laccate *G. applanatum* (Pers.) Pat. (= *G. lipsiense* (Batsch) G.F. Atk.), which produces basidiomes characterised by skeleto-ligative hyphae with intercalary or terminal branching and hyphal pegs (absent in *Ganoderma* taxa of Clade A). Moreover, the pilei possess velutinous pileal surface (“trichodermatous” according to Steyaert), a pileus crust less than 0.5 mm thick, a brown context without resinous deposits and significantly smaller basidiospores than in most other non-laccate *Ganoderma* spp. (i.e. of Clade C). *G. applanatum* is the second-best represented species in the databases including 424 entries (ca. 11% of the total number of generic sequences) deriving from material collected in Europe, Asia and North America on a wide range of angiosperms/gymnosperms, as well as from environmental samples (Table 1). Amongst the latter, one sequence deriving from soil in Antarctica (KC785577, originally deposited as “uncultured *Ganoderma*”) represents the only known sample of this species in the Southern Hemisphere, most possibly a human-mediated introduction by transportation of wood materials. *G. applanatum* is strongly supported (99%, 1.00; Fig. 6) and shows high intraspecific sequence similarity (99.71 ± 0.21%; min. 98.72%) and low genetic distance values (0.003 ± 0.002).

The other two species in Clade B form a well-supported sister clade (95%, 1.00; Fig. 6) and are hereby designated as “*Ganoderma* sp. B1” (corresponding to the UNITE DOI SH1723111.08FU) and “*Ganoderma* sp. B2” (corresponding to the UNITE DOI SH1723166.08FU). The former species includes four sequences from USA and China (in this particular case, geographic origins are inferred from the title of study which appears on the GenBank records), which show high similarity values (99.70 ± 0.15%) and form a terminal clade of strong support (100%, 1.00; Fig. 6). The latter species comprises three entries originating from material collected in Nepal under the names “*G. applanatum*”, “*G. lingzhi*” and “*G. multipileum*” and form a well-supported subclade (100%, 1.00; Fig. 6). Pairwise comparisons of sequences belonging to *Ganoderma* sp. B1 and *Ganoderma* sp. B2 demonstrate that these are well separated on the basis of genetic distance and sequence similarity values (0.048 ± 0.002 and 95.04 ± 0.17%, respectively); both new species present clear barcoding gaps between each other and in the comparisons vs. *G. applanatum* (Fig. 8).

### Clade C

Clade C is strongly supported (100%, 1.00; Fig. 6) and includes 67 unique sequences corresponding to 88 entries in total (Suppl. material 1: Table S2 and Suppl. material 6: Fig. S2d). The respective material presents a paleotropic distribution and originates from angiosperm and gymnosperm hosts. This clade is further divided into two clusters, including six species. Cluster C.1 (99%, 1.00; Fig. 6) corresponds to *G. neojaponicum* Imazeki and comprises eight sequences representing 10 entries from material originating from China, Laos, Myanmar and Taiwan. The taxon is characterised by a rather high sequence heterogeneity as evidenced by the respective intraspecific similarity (97.51 ± 1.44%) and genetic distance values (0.019 ± 0.009); variability in their ITS sequences is expressed by at least four to seven different positions in ITS1 and one to eight in ITS2 (Suppl. material 3: Fig. S1).

Cluster C.2 is strongly supported (100%, 1.00; Fig. 6) and it is composed of 23 sequences corresponding to 42 entries. It is further divided into two sister groups. One of them represents *G. aridicola* J.H. Xing & B.K. Cui and consists of sequences from specimens collected in Cameroon and South Africa growing on a large range of gymnosperms and angiosperms (Table 1; Fig. 6). The *G. aridicola* type material of South African origin grouped together with sequences provisionally labelled “Group 3” and “Group 4” by Kinge et al. (2012) by showing high intraspecific similarity (99.52 ± 0.19%). In addition, four GenBank entries, corresponding to what Kinge et al. (2012) named “Group 5” (JN105713 and JN105714) and “Group 6” (JN105715 and JN105716), formed two distinct well-supported subclades (82%, 1.00 and 100%, 1.00, respectively; Fig. 6). The former (“Group 5”) derives from material isolated on oil palm trees in Lobe (Cameroon) and is well separated from *G. aridicola* on the basis of both genetic distance and sequence similarity values (0.024 ± 0.002 and 96.29 ± 0.48%, respectively); therefore, it is considered as a distinct phylospecies and is hereby designated as “*Ganoderma* sp. C1” (corresponding to the UNITE DOI SH1723198.08FU). Similarly, sequences of “Group 6” originating from Cameroon as well, are also distantly placed from *G. aridicola* (genetic distance and sequence similarity values are 0.042 ± 0.002 and 94.48 ± 0.23%, respectively). Therefore, they are considered to represent a new phylogenetic species provisionally named “*Ganoderma* sp. C2” (corresponding to the UNITE DOIs SH1843092.08FU and SH1843096.08FU). The three taxa compose a well-supported subgroup (97%, 1.00; Fig. 6).

The other group (100%, 1.00; Fig. 6) within Cluster C.2 is formed by *G. enigmaticum*, M.P.A. Coetzee, Marinc., M.J. Wingf., *G. thailandicum* Luangharn, P.E. Mortimer, Karun. & J.C. Xu and *G. casuarinicola* J.H. Xing, B.K. Cui & Y.C. Dai. The former two are represented by six sequences from South Africa, Ghana and the Ivory Coast (including the type material of *G. enigmaticum*), as well as by two identical sequences from Thailand (type material of *G. thailandicum*) showing high sequence similarity (i.e. 99.55 ± 0.37%), which indicates that the Asian and African specimens do not correspond to two phylospecies on the basis of ITS data alone. However, the two taxa are maintained as distinct on the basis of the outcome of a multigene analysis of pertinent material (Luangharn et al. 2019b). On the other hand, *G. casuarinicola* is adequately supported (71%; Fig. 6) and here represented by 63 entries deriving from material (including the type specimen) originating mainly from India, Sri Lanka and China isolated from a large diversity of host plants; only four sequences were identified under this name, whereas the majority (47) were deposited as *Ganoderma* sp. (Table 1). In addition, sequence similarity values of *G. enigmaticum – G. thailandicum* vs. *G. casuarinicola* (98.97 ± 0.36) are indicative of their high affinity since overlapping intra- (e.g. 99.16 ± 0.42% within *G. casuarinicola*) and interspecific values are obtained; hence, a barcoding gap is absent between them (Fig. 8).

### Clade D

Clade D includes 291 sequences representing 449 individual entries separated into four clusters (Suppl. material 7: Fig. S2e); Cluster D.1 is evolutionary distant from Clusters D.2 to D.4 which jointly form a well-supported subclade (70%, 1.00; Fig. 6). Specimens present a tropical/subtropical distribution and originate from both Hemispheres excluding Europe, on angiosperm and gymnosperm hosts (Table 1, Suppl. material 7: Fig. S2e). Clade D is composed of laccate and partly laccate to dull taxa, which are morphologically distinguished from species grouped in Clade A by differences in spore shape (oblong-ellipsoid to ellipsoid, finely echinulate) and/or shape of cuticle cell (often irregular, clavate or cylindrical, with blunt outgrowths or protuberances or slight branches) and by darker pilei and/or context colour. Moreover, taxa placed in Clade D do not produce chlamydospores.

#### Clade D – Cluster D.1

Cluster D.1 is placed at the base of Clade D and it is strongly supported (98%, 1.00; Fig. 6). It consists of five sequences corresponding to eight entries; two entries are under the name *G. mbrekobenum* E.C. Otto, Blanchette, Held, C.W. Barnes & Obodai from Ghana (including the type material representing a laccate stipitate taxon; Otto et al. 2016), while the rest originated from Asia (India and Sri Lanka; Fig. 6) on a broad range of plant hosts and were deposited as *Ganoderma* sp. Similarly, good support for Cluster D.1 was obtained when additional (23) singletons/sequences were included in the analysis (99%, 1.00; Suppl. material 7: Fig. S2e, Suppl. material 1: Table S2); this larger sample-set presented intraspecies sequence similarity of 98.65 ± 0.70%. Although two or more subgroups are formed within this species (Fig. 6 and Suppl. material 7: Fig. S2e), ITS similarity and genetic distance values do not adequately support their distinct status; therefore and in the absence of additional data, we prefer to maintain a single species (i.e. *G. mbrekobenum*) in Cluster D.1.

#### Clade D – Cluster D.2

Cluster D.2 comprises two distantly-related species, i.e. *G. sinense* Zhao, Hsu & Zhang (Zhao et al. 1979b) and *G. nasalanense* Hapuar., Pheng. & K.D. Hyde (Hapuarachchi et al. 2019). The monophyletic *G. sinense* (100%, 1.00; Fig. 6) includes 26 sequences representing 66 entries deriving from Chinese material plus four collections from Thailand and a single collection from Taiwan (Suppl. material 1: Table S2). Most of them were labelled as *G. sinense* (45), while six were deposited under the names “*G. japonicum*”, “*G. formosanum*” and “*G. atrum*”. This finding is in accordance with reports advocating synonymy of *G. sinense* and *G. japonicum* (Fr.) Sawada (Liao et al. 2015; Wang and Yao 2005), whose authors examined specimens from China only. Therefore, in the absence of material from the type locality (Japan), a definite conclusion cannot be drawn regarding the status of *G. japonicum*. In addition, the sole sequence available under the name *G. atrum* Zhao, Hsu & Zhang (JQ886403; China, Hainan Island) grouped together with *G. sinense* in the present phylogeny. Similarly, a sequence (the joinder of X78752 and X78773) from a specimen originally identified as *G. formosanum* T.T. Chang & T. Chen was also positioned within *G. sinense*. On the other hand, *G. nasalanense* is strongly supported (98%, 1.00; Fig. 6) and includes two identical sequences (one from the type material) from Laos plus 13 entries from specimens collected in India, Malaysia and Vietnam, initially labelled as “*G. australe*”, *Ganoderma* sp. and “uncultured soil fungus” (Table 1, Suppl. material 7: Fig. S2e). Sequence similarity and genetic distance values support the distinct phylogenetic status of *G. nasalanense* with respect to *G. sinense* as evidenced by the clear barcoding gap they exhibit (94.17 ± 0.55% and 0.051 ± 0.002, respectively; Fig. 8).

#### Clade D – Cluster D.3

Cluster D.3 is well supported (75%, 1.00; Fig. 6) and includes four species. One corresponds to *G. cupreum* (Cooke) Bres. described on the basis of material collected in Africa and is represented by eight entries; three of them originate from Cameroon, one (deposited as “G. cf. cupreum”) from South Africa, one as “*G. chalceum*” (originating from Tanzania on the basis of the respective submission’s title), one environmental sample from Gabon and one of unknown origin, while a “*G. australe*” entry from Malaysia was placed at the base of the subclade. We maintain the name *G. cupreum* (since it has priority over *G. chalceum*) for the respective phylogenetic species, which forms a well-supported clade (99%, 1.00; Fig. 6) presenting high intraspecific values for sequence similarity (99.61 ± 0.21%) and low for genetic distance (0.004 ± 0.002).

Another terminal subclade is composed of nine entries (98%, 1.00; Fig. 6); five of them were originally deposited as *G. ecuadoriense* W.A. Salazar, C.W. Barnes & Ordóñez (three from Ecuador, including the type specimen and two from Brazil), one as *G. subfornicatum* Murrill, two as *Ganoderma* sp. (Brazil and Peru) and one labelled as “uncultured fungus” (Suppl. material 1: Table S2). The latter is the only one not originating from the Neotropics and derives from soil sampled in India (KJ411557). Material labelled as *G. subfornicatum* was identified as *G. orbiforme* (Fr.) Ryvarden (Ryvarden and de Meijer 2002) and, hence, the former was not included in a subsequent study of Ryvarden (2004) on neotropical polypores. On the other hand, Torres-Torres et al. (2012) treated *G. subfornicatum* and *G. orbiforme* as distinct taxa despite their being very similar in morphology. In addition and in the context of the present study, it was evidenced that the sole sequence available under the name *G. subfornicatum* (JX082352, French Guiana) was placed within the same terminal clade as sequence KU128524 representing the holotype of *G. ecuadoriense*. The latter is a recently-described species from Ecuador (Salazar et al. 2016); however, although those authors conducted a morphological and molecular study on their material, they did not include specimens of *G. subfornicatum*, which commonly occurs in the same larger area (type material from Belize) (Welti and Courtecuisse 2010; Torres-Torres et al. 2012). On the basis of the previously-presented information, *G. subfornicatum* is maintained to describe this terminal subclade (although only one sequence is available under this name in GenBank) and “*G. ecuadoriense*” is abandoned as nom. illeg.

Five sequences, representing *G. orbiforme*, form a well-supported terminal group (89%, 0.96; Fig. 6) which, however, presents high affinity to the sister clade of *G. subfornicatum* (100%, 0.96; Fig. 6), as is evidenced by the absence of a barcoding gap in the pairwise comparison of the two taxa (genetic distance and sequence similarity: 0.011 ± 0.002 and 98.69 ± 0.23%, respectively). *G. orbiforme* was originally described from Guinea, but was also reported from South America (Ryvarden 2000; Baltazar and Gibertoni 2009; Gomes-Silva et al. 2011; Torres-Torres et al. 2012). Although none of these studies included molecular data, in some of them (e.g. Ryvarden 2000; Torres-Torres et al. 2012), the type specimen of *G. orbiforme* was examined alongside *Ganoderma* basidiomes from the Neotropics to assess the identity of the latter. Moreover, four sequences from Brazilian specimens identified as *G. orbiforme* (de Lima Júnior et al. 2014) were grouped within this particular subclade. Therefore, we believe that *G. orbiforme* is the correct name for this phylogenetic species which occurs in the Neotropics. The use of this name for material originating from Asia and Australia (Wang et al. 2014; Hapuarachchi et al. 2019) is not supported by molecular evidence and erroneous conclusions could be attributed to the high morphological variability of specimens belonging to closely-related taxa of Cluster D.3, for example, *G. mastoporum* and *G. orbiforme* (Fig. 6).

Finally, a well-represented and supported terminal subclade of Cluster D.3 (80%, 1.00; Fig. 6) includes 122 entries (95 of them are singletons; Suppl. material 1: Table S2) deriving from specimens collected in southeast Asia and Australia mainly on eudicot angiosperms. It consists of sequences deposited under the names *G. mastoporum* (12), “*G. orbiforme*” (19), “*G. cupreum*” (10), “*G. fornicatum*” (3), “*G. multicornum*” (1) and *Ganoderma* sp. (11), as well as under “*G. australe*” (60, resulting from a single study conducted on material originating from Borneo). On the basis of what was previously mentioned about the correct phylogenetic position of *G. orbiforme* and although sequences labelled as “*G. orbiforme*” deriving from China were included under this name in previous studies (Wang et al. 2014; Hapuarachchi et al. 2018b; Xing et al. 2018), we believe that relevant material originating from southeast Asia and Oceania corresponds to *G. mastoporum* (Lév.) Pat. (initially described on the basis of material collected in Singapore) and that the 12 *G. mastoporum* entries in this particular subclade of Cluster D.3 were correctly identified. Therefore, *G. mastoporum* cannot be considered as a synomym of *G. orbiforme* (as stated in MycoBank and Index Fungorum) since the latter name corresponds to a related – yet distinct – phylogenetic species (interspecific sequence similarity and genetic distance values: 97.34 ± 0.49% and 0.025 ± 0.005, respectively). Similarly, *G. mastoporum* and *G. cupreum* could be separated on the basis of the outcome of the present study because they form distinct well-supported terminal groups (Fig. 6, Suppl. material 7: Fig. S2e) presenting interspecific sequence similarity of 97.20 ± 0.45%. One entry, labelled as *G. multicornum* (MT772000, Suppl. material 1: Table S2; Suppl. material 7: Fig. S2e), shows relatively high affinity (genetic distance: 0.012 ± 0.008, sequence similarity: 98.65 ± 0.70%) with the rest of the entries within the *G. mastoporum* clade (Fig. 6, Suppl. material 7: Fig. S2e).

#### Clade D – Cluster D.4

Cluster D.4 is strongly supported (93%, 1.00; Fig. 6) and is further divided into two sister groups, i.e. Group D.4.1 and D.4.2; the former includes species collected on angiosperms and gymnosperms, while the latter, the so-called “palm group”, comprises sequences from material mostly associated with monocotyledons.

Group D.4.1 (87%, 1.00; Fig. 6) includes the recently-introduced sessile and laccate *G. angustisporum* J.H. Xing, B.K. Cui & Y.C. Dai reported from China on *Casuarina equisetifolia* (Xing et al. 2018). Moreover, sequences from specimens of different origin (India, Malaysia and Australia, labelled as “*G. australe*” and *Ganoderma* sp.; Table 1) growing on other eudicots also nested together with *G. angustisporum* and present high intraspecific similarity values (99.07 ± 0.43%), thus largely expanding the known distribution of this species and the number of host plant taxa it is associated with (Table 1, Suppl. material 7: Fig. S2e). A closely-related entity to *G. angustisporum* is found as a terminal subgroup (100%, 1.00; Fig. 6) and it includes two singletons initially labelled as “*G. applanatum*” from material collected in Africa (Gabon) on eudicots. It presents genetic distances and sequence similarity values of 96.87 ± 1.21% and 0.027 ± 0.004, respectively, vs. *G. angustisporum*. It is therefore considered as a new phylogenetic species and is provisionally labelled as “*Ganoderma* sp. D1” (corresponding to the UNITE DOIs SH1740449.08FU and SH1740450.08FU).

Group D.4.2 (91%, 1.00; Fig. 6) is composed of five species comprising material originating mainly from monocot angiosperms. Three of them are distinguished by a clear barcoding gap (Fig. 8) and form a well-supported terminal subclade (79%, 0.99, Fig. 6). *G. zonatum* Murill (100%, 1.00; Fig. 6 and Suppl. material 7: Fig. S2e) consists of 84 correctly-labelled entries with high similarity values (99.86 ± 0.08%), originating from specimens growing on palms in southeast USA. *G. ryvardenii* Tonjock & Mih (100%, 1.00; Fig. 6) is represented by 22 entries mainly deriving from Cameroon (including the sequence from the type material, Table 1). Moreover, four entries, labelled as *Ganoderma* sp. from Colombia, form a distinct strongly-supported clade (100%, 1.00; Suppl. material 7: Fig. S2e) representing, thus, a new phylogenetic species provisionaly named *Ganoderma* sp. D2 (corresponding to the UNITE DOI SH1723113.08FU).

The other two species comprise material from Asia only; the ‘core’ part corresponds to *G. boninense* Pat. and it is represented by 69 entries deposited as *G. boninense* (32), “*G. miniatocinctum*” (3), “*G. orbiforme*” (2), “*G. zonatum*” (3) and *Ganoderma* sp. (29) (Table 1; Fig. 6). The rest of the entries form a terminal subgroup with strong support (98%, 1.00; Fig. 6) and includes material originating from Indonesia. This material was originally identified to genus level only (12 entries, labelled as *Ganoderma* sp.; Table 1). Sequence similarity and genetic distance values vs. *G. boninense* (97.30 ± 0.67% and 0.029 ± 0.006, respectively) are indicative of the presence of a new phylogenetic species, provisionally named *Ganoderma* sp. D3 (corresponding to the UNITE DOIs SH1723050.08FU and SH1723098.08FU).

On the basis of the results presented above, it is apparent that *G. zonatum* is not related to *G. sessile* (the latter forms part of Cluster A.2), as previously reported by Gottlieb et al. (2000); the former name was misapplied to a specimen originating from Argentina. Neither *G. zonatum* nor *G. boninense* forms part of the *G. lucidum* complex (Zhou et al. 2015) since they are distinctly positioned into Clade D (Fig. 4). In addition, three sequences available under the name *G. miniatocinctum* Steyaert are grouped together with *G. boninense* material.

### Clade E

Clade E is strongly supported (81%, 1.00; Fig. 7). It includes 98 unique sequences representing 393 individual entries (or 367 sequences representing 664 individual entries; Suppl. material 8: Fig. S2f) and comprises 15 well-supported phylospecies (Table 1). The respective material presents a worldwide distribution and originates from angiosperm and gymnosperm hosts (Table 1 and Suppl. material 1: Table S2 and Suppl. material 8: Fig. S2f). Clade E is further subdivided into five Clusters (E.1 to E.5) and includes sequences from specimens that are characterised by sessile and perennial basidiomes, mostly dull and less often laccate (e.g. Cluster E.3). In addition, their pileal crust does not appear as a regular palisade, often consisting of a mixture of randomly orientated, branched arboriform skeletal hyphae and a degenerated palisade of irregular generative hyphal ends. The latter are usually embedded in a resinous matrix which may become very thick in aged basidiomes, making the examination of crust anatomy practically impossible. The presence of species producing either laccate or non-laccate pilei evidences that this particular morphological trait, widely used for grouping *Ganoderma* taxa at the subgeneric level, is not in congruence with phylogenetic data.

#### Clade E – Cluster E.1

Cluster E.1 corresponds to a single strongly-supported phylospecies (99%, 1.00; Fig. 7) distinctly placed at the base of Clade E. It includes material from tropical/subtropical Asia which is mainly associated with the name “*G. australe*” (29 entries) (Table 1); however, *G. williamsianum* Murrill. (7) is the correct name to assign to this terminal group since relevant descriptions and reported occurrence (Steyaert 1972; Corner 1983; Wang and Wu 2010; Xing et al. 2018) are in agreement with the phylogenetic position presented here. In addition, *G. williamsianum* presents high intraspecific similarity values (99.47 ± 0.22%) despite the large number of entries examined and their rather wide geographic origin. Cluster E.1 corresponds to ‘clade 8’ of the phylogeny presented by Moncalvo and Buchanan (2008).

#### Clade E – Cluster E.2

Cluster E.2 (84%, 0.99; Fig. 7) includes 43 sequences representing 176 entries (or 177 sequences deriving from 310 entries in the expanded dataset; Suppl. material 8: Fig. S2f). It consists of seven phylogenetic species, two of which have a neotropical distribution (including USA) while the rest occur in Asia, Africa and Oceania (Table 1).

A well-supported terminal subclade (87%, 0.99; Fig. 7) is formed by 23 entries with high intraspecific sequence similarity (99.64 ± 0.23%, min. 99.11%) deriving from material originating from the Neotropics on eudicots under various names, i.e. “*G. tornatum*” (6), “*G. lobatum*” (3), “*G. parvulum*” (1), “*G. gibbosum*” (1), “*G. applanatum* complex” (8) and *Ganoderma* sp. (4) (Table 1 and Suppl. material 1: Table S2). Since some of these names are in use for other sequences examined in this study (positioned in other Clades/Clusters, for example, as is the case for *G. parvulum* and *G. lobatum*), while for others there is not adequate evidence to support their correct use in this particular case (e.g. *G. tornatum*), we prefer to label this distinct phylogenetic entity as *Ganoderma* sp. E1 (corresponding to the UNITE DOI SH1723047.08FU). This species corresponds to ‘clade 7’ in the study of Moncalvo and Buchanan (2008). As is the case elsewhere in the present study, properly/accurately identified type material (representing one or more of the taxa whose names appear in this subclade) needs to be sequenced in order to arrive at robust conclusions regarding the real identity of this particular phylospecies.

A strongly-supported (96%, 1.00; Fig. 7) sister group to the previous phylospecies consists of 37 sequences deriving from specimens collected in South and Central America, as well as in the USA (Florida) on a wide range of host-plants, for example, Cenostigma pluviosum var. peltophoroides, *Inga vera*, *Jacaranda mimosifolia*, *Leucaena leucocephala* and *Elaeis guineensis*. These sequences, initially deposited under various names (i.e. “*G. gibbosum*” (12), “*G. tornatum*” (8), “*G. lobatum*” (7), “*G. australe*” (2) and “*G. applanatum* complex” (2)), are hereby placed under the name *Ganoderma* sp. E2 (corresponding to the UNITE DOI SH1723047.08FU). We believe that accurate association of this species to any established *Ganoderma* taxon name is not possible until additional information – through the study of relevant type material – becomes available. Although *Ganoderma* sp. E1 and *Ganoderma* sp. E2 demonstrate relatively-high sequence similarity and rather low genetic distances in pairwise comparisons (97.73 ± 0.54% and 0.017 ± 0.005, respectively), there is no overlap between the respective intra- and interspecies values; their distinct species status is therefore proposed.

Cluster E.2 also includes a large terminal subclade (65%, 1.00; Suppl. material 8: Fig. S2f) comprising material from Asia, Africa and Oceania. Clade E.2 corresponds to the *G. gibbosum* complex and consists of (at least) five species, i.e. *G. gibbosum* (Blume & T. Nees) Pat., the recently introduced *G. eickeri* Tchotet, M.P.A. Coetzee, Rajchenb. & Jol. Roux, *G. ellipsoideum* Hapuar., T.C. Wen & K.D. Hyde, as well as two new phylospecies (*Ganoderma* sp. E3 and *Ganoderma* sp. E4). *G. gibbosum* is composed of 107 entries (40 of which are singletons) labelled mainly under the names *G. gibbosum* (59), “*G. applanatum*” (27) and “*G. australe*” (8) (Suppl. material 1: Table S2 and Suppl. material 8: Fig. S2f). It shows low intraspecific sequence variability (sequence similarity: 99.58 ± 0.17%; genetic distance: 0.004 ± 0.002). A distinct entity, here called G. aff. gibbosum, is exclusively composed of sequences of Indian origin (51 entries including 10 singletons; 69%, 1.00; Fig. 7) which are mainly deposited as *Ganoderma* sp. However, the relatively-high affinity exhibited by sequences of G. aff. gibbosum vs. other *G. gibbosum* sequences from Asia (sequence similarity and genetic distance values: 99.04 ± 0.27% and 0.009 ± 0.002, respectively) prevents us from considering it as a distinct phylospecies, at least until further evidence becomes available.

The other terminal clade (92%, 1.00; Fig. 7) represents the recently-introduced *G. eickeri* consisting of four entries, including the type material, which originate from South Africa on angiosperms (Tchotet Tchoumi et al. 2019). However, on the basis of the ITS data evaluated in this study, *G. eickeri* appears closely related to *G. gibbosum* (interspecific sequence similarity and genetic distance values: 98.90 ± 0.25% and 0.011 ± 0.003, respectively).

*G. ellipsoideum* is a recently-described species from Hainan Island, China (Hapuarachchi et al. 2018b). It is here represented by 81 entries corresponding to 10 ASVs and 44 singletons (0.99; Fig. 7). Sequences within the *G. ellipsoideum* subclade (including the one from the type material) were mainly labelled as “*G. gibbosum*” (22), “*G. australe*” (10), “*G. australe* cplx” (10), “*G. applanatum*” (5) and *Ganoderma* sp. (10); they originate from material of broad geographic distribution (south and east Asia, Oceania) on eudicots. In addition, this study revealed that two environmental samples deriving from the USA (UDB0769802 and UDB0763546) formed part of this terminal subclade. As in the case of *G. eickeri*, *G. ellipsoideum* is closely related to *G. gibbosum* (sequence similarity and genetic distance values: 98.84 ± 0.19% and 0.012 ± 0.002, respectively) and no barcoding gaps were detected with respect to the closest related species (i.e. *G. gibbosum* and *G. eickeri*; Fig. 8); therefore, its distinct phylogenetic status is questionable on the basis of ITS data. *G. gibbosum* and *G. ellipsoideum* correspond to ‘clade 5’ of the phylogeny presented by Moncalvo and Buchanan (2008).

The other two phylogenetic species appearing on the terminal subclade of Cluster E.2 (96%, 1.00; Fig. 7) are hereby designated as *Ganoderma* sp. E3 (corresponding to the UNITE DOIs SH1723116.08FU and SH1723270.08FU) and *Ganoderma* sp. E4 (corresponding to the UNITE DOI SH1677211.08FU); both demonstrate high intraspecific sequence similarity values (> 99.31%). The former consists of six sequences deposited as “*G. australe*” deriving from Indonesian and Australian material. The latter species (100%, 1.00; Fig. 7) corresponds to ‘clade 6’ in the study of Moncalvo and Buchanan (2008) and includes 13 entries from specimens originating from Malaysia and Indonesia on eudicots, which were also labelled “*G. australe*” (Table 1). Both species are well separated from each other (*Ganoderma* sp. E3 vs. *Ganoderma* sp. E4: 94.67 ± 1.02% and 0.030 ± 0.006 for sequence similarity and genetic distance values, respectively), as well as from the rest of the species within Cluster E.2, for example, *Ganoderma* sp. E3 vs. *G. ellipsoideum*: 96.84 ± 1.01% and 0.025 ± 0.006, respectively.

#### Clade E – Cluster E.3

Cluster E.3 consists of material corresponding to the laccate taxa *G. pfeifferi* Bres. (17 sequences from Europe only; Table 1), *G. cupreolaccatum* Kalchbr. ex Z. Igmándy (invalid name, type locality: Hungary), *G. mutabile* Cao & Yuan (two sequences from China, including one from the type specimen) and the recently-introduced *G. knysnamense* Tchotet, M.P.A. Coetzee, Rajchenb. & Jol. Roux (four entries from South Africa, including the type; Tchotet Tchoumi et al. 2019), which were adequately supported (71%, 0.97; Fig. 7). Amongst them, *G. knysnamense* is placed at the base of this cluster (100%, 1.00; Fig. 7) and is well separated from the closest species (i.e. *G. mutabile*: 0.036 ± 0.001 and 96.43 ± 0.13%). Furthermore, *G. pfeifferi* and *G. mutabile* are placed on well-supported terminal subclades (90%, 1.00; Fig. 7) and they both present high intraspecific similarity (> 99.84%); however, the respective interspecific values in pairwise comparisons are indicative of their affinity (98.14 ± 0.19%, 0.019 ± 0.005; Fig. 8). *G. mutabile* was introduced as a distinct species after examining only one collection which, according to its authors (Cao and Yuan 2013), resembles *G. pfeifferi* with respect to the dark brown context and the similar spore size; however, it differs by its laccate crust and non-stratified tubes. The other noteworthy sequence within Cluster E.3 derives from a specimen identified as *G. cupreolaccatum*, which was previously considered as a facultative (heterotypic) synonym of *G. pfeifferi* (Mycobank). However, both similarity and genetic distance values of this particular sequence vs. *G. pfeifferi* are indicative of a distinct species (i.e. 94.97 ± 0.17% and 0.030 ± 0.007, respectively). Still, the variability of this unique sequence is located exclusively at the beginning of ITS1, a region generally conserved between closely-related taxa of the genus (Nilsson et al. 2017), while the rest is identical to those of *G. pfeifferi*. Therefore, questions are raised about the quality of this particular sequence and no definite conclusions could be drawn concerning the exact status of *G. cupreolaccatum*.

The close phylogenetic position of *G. pfeifferi* and *G. adspersum* (Schulzer) Donk (Cluster E.5) is congruent with their similar pileus dark-brown context and the complex structure of the crust in contrast to the normal palisade appearance in laccate species of Clade A (Hansen 1958; our observations); still, *G. pfeifferi* clearly differs by the laccate pileus and the width and quotient of spores (Ryvarden and Gilbertson 1993; Niemelä and Miettinen 2008; Ryvarden and Melo 2017). In addition, these characteristics, in conjunction with the cracked/wrinkled resinous layer on the pileus, distinguish *G. pfeifferi* from specimens of *G. lucidum* and *G. resinaceum* (Ryvarden and Gilbertson 1993). Furthermore, the outcome of the present study evidences the grouping of *G. pfeifferi* within Clade E and contrasts previous reports by Hseu et al. (1996) and Cao and Yuan (2013), which linked this taxon with the *G. lucidum* complex and *G. resinaceum*, respectively. This discrepancy is apparently due to an initial misidentification of strain CBS 747.84 (sequences X78738/ X78759) which was labelled as “*G. pfeifferi*” instead of *G. resinaceum* (Moncalvo et al. 1995a).

#### Clade E – Cluster E.4

Cluster E.4 (0.99; Fig. 7, and 82%, 1.00; Suppl. material 8: Fig. S2f) includes five species represented by sequences from material with a world-wide distribution (apart from Europe) occurring on angiosperms. A well-supported subgroup within this cluster is formed by the recently-introduced *G. chocoense* J.A. Flores, C.W. Barnes & Ordoñez, including only the sequence from the type material (Ecuador; Flores et al. 2018) and *G. podocarpense* J.A. Flores, C.W. Barnes & Ordoñez (Flores et al. 2017), consisting of two sequences from Ecuador and Panama. Despite their overlapping distribution range, the values of sequence similarity (97.57 ± 0.13%) and genetic distance (0.025 ± 0.003) are indicative of their distinct status at species level.

A sister group to the former (1.00; Fig. 7 and 95%, 1.00; Suppl. material 8: Fig. S2f) is composed of eight sequences originating from Argentinian material under the names “*G. lobatum*”, “*G. tornatum*” and *Ganoderma* sp. (Table 1). On the basis of the information available, it is not possible to assign a particular taxonomic name to these sequences. Therefore, we prefer to designate this phylogenetic species as *Ganoderma* sp. E5 (corresponding to the UNITE DOI SH1678465.08FU) (100%, 1.00; Fig. 7). The latter entity is well separated from *G. podocarpense*, i.e. sequence similarity: 95.58 ± 0.59% and genetic distance: 0.044 ± 0.006.

A sister terminal subclade to the previous group (*G. chocoense*, *G. podocarpense* and *Ganoderma* sp. E5) consists of sequences originating from material collected in south and southeast Asia, while a single sequence indicates its presence also in Papua New Guinea (95%, 1.00; Fig. 7). They are labelled mainly as “*G. australe*” (19), “*G. applanatum*” (2) and *Ganoderma* sp. (8) (Table 1). Again, none of the initially-assigned names could be maintained; therefore, we refer to this distinct phylogenetic species as *Ganoderma* sp. E6 (corresponding to the UNITE DOI SH1723070.08FU) presenting low interspecific sequence similarity and high genetic distance vs. *Ganoderma* sp. E5 (i.e. 95.59 ± 0.70% and 0.040 ± 0.006, respectively). *Ganoderma* sp. E5 and *Ganoderma* sp. E6 appear as sister subclades in ‘clade 4’ of the phylogeny presented by Moncalvo and Buchanan (2008). Moreover, a single sequence (AF255183; Suppl. material 1: Table S2 and Suppl. material 8: Fig. S2f), originating from New Zealand, seems to correspond to a distinct entity, which however does not fulfil the criteria set in this study to merit recognition as a *Ganoderma* phylospecies (genetic distance and sequence similarity values vs. *Ganoderma* sp. E6: 0.017 ± 0.002 and 98.27 ± 0.18, respectively).

Finally, a distinct group included 80 entries deriving from specimens mainly from southeast Asia, South America and Oceania, as well as from central/north America and South Africa. That said, one sequence originated in the vicinity of the Kew Botanical Gardens, UK, such that we suspect it to have been imported with plant material. These sequences were mostly deposited as “*G. australe*” (27), “*G. australe* complex” (14) and *Ganoderma* sp. (13). Despite the widespread use of the “australe” epithet to describe several entities (14) placed in other terminal clades of the present study, we believe that this particular terminal group coincides with *G. australe* (Fr.) Pat. after evaluating all available ITS sequence data, the geographic distribution of specimens analysed and pertinent publications (Ryvarden and Gilbertson 1993; Moncalvo and Ryvarden 1997; Moncalvo and Buchanan 2008). As regards sequence similarity, intraspecific values are notably high (99.56 ± 0.25%) considering the diverse geographic origin of the respective material, whereas interspecific values to the closest taxon is low (i.e. *Ganoderma* sp. E6; 95.04 ± 0.66%). Although ambiguities exist about the exact distribution range of *G. australe* (Yeh and Chen 1990; Buchanan and Wilkie 1995; Moncalvo and Ryvarden 1997; Smith and Sivasithamparam 2000) and the type specimen originating from the Pacific area is lost, this appears to be the most common *Ganoderma* species throughout the tropics and subtropics and corresponds to ‘clade 3’ in the study of Moncalvo and Buchanan (2008).

#### Clade E – Cluster E.5

Cluster E.5 (97%, 1.00; Fig. 7) comprises sequences from specimens originating from the Northern Hemisphere and it is further divided into two well-supported subclades. One corresponds to a new phylogenetic species hereby designated as *Ganoderma* sp. E7 (corresponding to the UNITE DOI SH1723077.08FU), which is composed of 17 entries (100%, 1.00; Fig. 7) deposited under the name “*Ganoderma* sp.”, “*G. lobatum*” and “*G. applanatum* cplx” deriving from US material only (intraspecific sequence similarity: 98.83 ± 0.90%; interspecific sequence similarity vs. *G. adspersum*: 95.29 ± 0.51%). “*Ganoderma* sp. E7” forms part of ‘clade 2’ in the phylogeny presented by Moncalvo and Buchanan (2008).

A sister subclade to the aforementioned clade corresponds to *G. adspersum* sensu lato and is represented by 155 entries deriving from Europe, south and west Asia and North Africa (1.00; Suppl. material 8: Fig. S2f). *G. adspersum* (Schulzer) Donk is a common species in the Palearctic realm; in Europe, it is usually reported on a wide range of angiosperms (Ryvarden and Gilbertson 1993), but it also appears on gymnosperms (i.e. *Abies cephalonica*; Zervakis et al. 1998; this study). Most of the sequences (117) examined in this study were properly identified as *G. adspersum*. However, several misidentifications were noted, mainly for Asian material labelled as “*G. applanatum*”, “*G. australe*” and “*G. australe* complex” (Suppl. material 1: Table S2). In fact, *G. adspersum* was considered as a synonym of *G. australe* (Ryvarden 1976; Ryvarden and Gilbertson 1993; Ryvarden and Melo 2017), but ITS sequence data clearly separated it from *G. australe*. The outcome of this study reveals that *G. adspersum* includes specimens with a distribution ranging from Europe to India (89%, 1.00; Fig. 7). Moreover, nine entries from samples, originally identified as *G. adspersum*, “*G. australe* complex”, “*G. applanatum*” and “uncultured fungus” (China, Japan and South Korea; Suppl. material 1: Table S2), form a distinct terminal subgroup (72%; Fig. 7), which is hereby named G. aff. adspersum on the basis of its close affinity to *G. adspersum* (i.e. sequence similarity and genetic distance values: 98.47 ± 0.33% and 0.013 ± 0.004, respectively).

In conclusion, the plasticity of morphological characters and substantial overlap of alleged diagnostic features is prevalent in the ‘dull’ taxa of this group; consequently, their taxonomic significance is dubious. Moreover, the massive, heavily-agglutinated matrix of the pileal crust in such non-laccate species often obstructs observation of discriminating features in pileal elements. The situation is further aggravated by the loss of type material of widely and commonly used species names (e.g. *G. australe* and *G. gibbosum*), the absence of (correct) neo-typification (as there are ambiguous synonymies regarding several taxa) and unclear species descriptions (e.g. *G. tornatum* and *G. lobatum*). All of the above could explain the obstacles which prevented the establishment of a stable classification system for species of Clade E. Still, the highly variable morphology of basidiomes and the ITS divergence are indicative of underestimated diversity and the presence of cryptic species is quite certain as is also indicated by the outcome of the present study.

### Variation in ITS spacers of *Ganoderma* sequences

**Figure 9.**
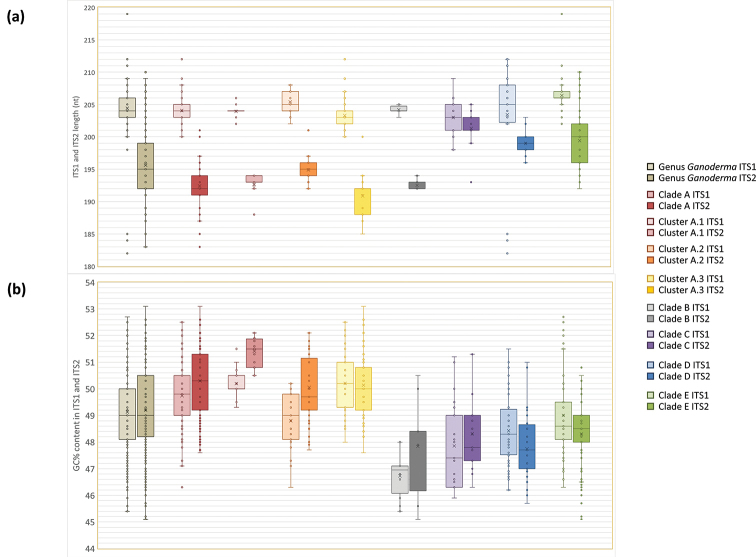
Box plots of **a** length (bases) and **b** GC (%) content of ITS1 and ITS2 sequences for each one of the main lineages (Clades/Clusters) of the genus *Ganoderma*. The size of each box represents 50% of the values, the black horizontal line within each box indicates the median, the ‘x’ represents the average value, the error bars represent interquartile ranges and circles indicate outliers.

As determined from the analysis performed in this study, the combined length of the two spacers (excluding the intercalary 5.8S gene) ranged from 378 (*Ganoderma* sp. D1) to 429 bases (*Ganoderma* sp. E4) with an average value of 400 (± 6.8) bases (Fig. 9). In addition, the ITS1/ITS2 lengths in Clades C (404.3 ± 4.4 bases), D (402.4 ± 7.9) and E (405.70 ± 6.3) are greater than those in Clade A (396.5 ± 4.2) and B (396.7 ± 1.1). The length of ITS1 ranged from 182 (*Ganoderma* sp. D1) to 219 bases (*Ganoderma* sp. E4) with an average value of 204.4 (± 4.1) bases, while the respective values for ITS2 were from 183 (*G. shanxiense*) to 210 bases (*Ganoderma* sp. E4) and average values of 195.6 ± 4.9 bases (Fig. 9). The vast majority of *Ganoderma* species (ca. 95%) exhibit longer ITS1 than ITS2 sequences; the greatest difference was recorded in *G. wiiroense* (average size difference 21.5 bases) and *G. shanxiense* (18.0). In addition, a clear delimitation in this respect was observed amongst various clades since the difference in length between ITS1 and ITS2 was higher in Clades A (11.6 bases in average) and B (11.3) than in E (7.2), D (4.0) and C (2.5). These results are in agreement with those previously reported on the ITS1/ITS2 spacers length in Basidiomycota and Ascomycota; in both cases, ITS1 was longer that ITS2 (Wang et al. 2015). In contrast, data originating from various fungal phyla (including Basidiomycota) showed that ITS2 is generally longer than ITS1 (Yang et al. 2018).

In the ITS1 region (289 sites), 195 (67%) were variable and 159 (55%) were parsimony informative; the ITS2 region (262 sites) included 169 (65%) variable and 137 (52%) parsimony informative sites. This is in accordance with the outcome of previous reports indicating a larger variability for ITS1 in comparison to ITS2 in Fungi (Nilsson et al. 2008). The largest intraspecies variability in ITS1 size was found in *Ganoderma* sp. C1 (203.0 ± 4.2 bases), *G. wiiroense* (209.0 ± 3.6), *G. martinicense* (204.3 ± 2.3), *G. aridicola* (206.0 ± 2.2), *Ganoderma* sp. D1 (183.5 ± 2.1) and *G. subfornicatum* (206.0 ± 2.0), while the greatest intraspecies variability in ITS2 length was observed in *Ganoderma* sp. E3 (206.3 ± 2.5 bases), *Ganoderma* sp. D3 (200.1 ± 2.0), *Ganoderma* sp. E7 (195.0 ± 2.0), *G. angustisporum* (199.0 ± 1.9) and *G. wiiroense* (187.5 ± 1.9). In general, for the entire genus and for individual Clades/Clusters, ITS2 showed a greater variability in length (with the notable exception of Clade D).

The GC content was almost identical in ITS1 and ITS2 spacers when calculated for the entire genus (49.1 ± 1.5% and 49.2 ± 1.7%, respectively) (Fig. 9); however, species of Clade A showed distinctly higher GC content for the two spacers (49.7 ± 1.2% and 50.3 ± 1.2%, respectively) in comparison to the rest of the *Ganoderma* spp., whereas the lowest values were recorded in Clade B (46.7 ± 0.8% and 47.8 ± 1.6%, respectively). Relevant literature data on Basidiomycota refer to slightly higher values of GC content in ITS2 compared to ITS1, i.e. median values of 45% vs. 44% (Wang et al. 2015) or average values of 46.06% vs. 43.49% (Yang et al. 2018), respectively.

### Phylospecies in *Ganoderma*

The application of criteria of wide applicability/suitability for delineating taxa in the genus *Ganoderma* is a particularly challenging task because species exhibit complex evolutionary backgrounds, widespread occurrence and/or problematic taxonomy as previously mentioned. Since this study was principally based on the analysis of ITS metadata, difficulties related to erroneous, fragmentary and/or incomplete information on the origin and the true identity of the sequenced material had to be addressed, together with labelling referring to the genus only (i.e. “*Ganoderma* sp.”) or with unidentified sequences (e.g. “Agaricales sp.”, “uncultured fungus” and “unidentified soil fungus”). The outcome demonstrated that the use of ITS rDNA could confer valuable data on the establishment of phylogenetic species within the genus since the majority of terminal clades were strongly supported and species boundaries were – in the majority of cases – elucidated, although relationships/affinities amongst particular sections or within certain species complexes were not adequately resolved.

Intraspecific ITS sequence similarity values were relatively high (i.e. overall average: 99.32 ± 0.59%) for the *Ganoderma* species examined. Moreover, genetic distance, based on uncorrected p-values, provided an additional effective tool for species delineation which was found to be congruent with ITS sequence similarities (overall average of intraspecies genetic distance within the genus: 0.005 ± 0.004). Hence, in the context of the criteria adopted, these parameters contributed significantly to delineating *Ganoderma* species and were generally in accordance with the concepts of already accepted morpho- and/or phylospecies. In addition, a species hypothesis using a threshold of interspecific values for ITS similarity (≤ 98%) and genetic distance (≥ 0.015) was effectively applied for the 21 putatively new phylospecies hereby proposed, while several other distinct entities of dubious status are revealed in the trees inferred. Finally, 59 terminal groups correspond to already established species of the genus which demonstrate a large variability in genetic distance and sequence similarity when selected pairs of phylogenetically-related taxa are compared within different clades (Fig. 8). This was particularly evident in lineages where the barcoding gap amongst taxa is often minimal (i.e. Cluster A.2) or even non-existent (i.e. Cluster A.1). In such cases, species discrimination on the basis of ITS sequence variability is problematic.

Especially as regards taxa of Cluster A.1, average interspecific distances (0.008 ± 0.004) are close to the respective intraspecific values (0.004 ± 0.003); similarly, sequence similarity in interspecific comparisons is high (99.0 ± 0.6; Fig. 8). In this particular case, only the formation of well-supported terminal clades in ITS phylogenies could help to address taxonomic issues (in conjunction with other criteria, for example, distribution, ecology and distinct morphoanatomical characters where available), as is the case with *G. leucocontextum* – *G. weixiensis*. No other taxon of A.1 is adequately resolved through ITS and multigene data are needed in order to delineate them (Zhou et al. 2015; Loyd et al. 2018; Ye et al. 2019). As regards the rest of Clade A, a barcoding gap – albeit narrow – is present for several species of Clusters A.2 and A.3 (Fig. 8), while the difference between genetic distance values within and amongst species is generally higher than in the case of Cluster A.1 (interspecies: 0.028 ± 0.010 and 96.5 ± 1.2% in A.2 and 0.046 ± 0.016 and 95.0 ± 1.4% in A.3). Still, comparisons between several taxa (e.g. *G. parvulum* and *G. mexicanum*, *G. sichuanense* and *G. weberianum*, *G. sessile* and *G. polychromum*, *G. ravenelii* and *G. curtisii* and *G. multipileum* and *G. martinicense*) produce values of genetic distance and sequence similarity which are lower than 0.015 and/or higher than 98%, respectively. Therefore, a ‘clear-cut’ threshold, based on such parameters, cannot be easily applied to separate them. ITS alone is not effective here and a combination of criteria (or the outcome of multigene phylogenies) is needed to elucidate the status of these taxa.

On the other hand, species in Clade B present a clear barcoding gap and distinct ‘sequence diameters’ (as defined by Schoch et al. 2012) corresponding to high interspecific distance and low sequence similarity values (> 0.045 and < 95.33%, respectively). Despite the large interspecies divergence between taxa of Clade B, the respective intraspecies values (0.003 ± 0.002 and 99.71 ± 0.20%) are similar to the total average of the genus. Hence, all three phylospecies comprising this clade (two of them proposed in this study) are well separated by using ITS only.

As regards Clades C, D and E, barcoding gaps are quite pronounced and delimitation of many species could be made on the basis of the 98% sequence similarity and the 0.015 genetic distance values previously mentioned and used for the establishment of the phylospecies proposed herein (Fig. 8). In particular, Clade C presents interspecific distance and sequence similarity values with considerable variability (0.049 ± 0.022 and 93.83 ± 1.26%, respectively); *G. casuarinicola* and *G. enigmaticum* – *G. thailandicum* are the only taxa demonstrating high phylogenetic affinity (on the basis of ITS data) which questions their distinct taxonomic position. Clade D includes well-separated species with pronounced differences when interspecific values of genetic distance and sequence similarity are considered (0.056 ± 0.017 and 93.68 ± 1.64%, respectively), whereas Clade E presents a rather high variability in the respective values amongst species (0.048 ± 0.016 and 94.19 ± 2.27%, respectively). Most taxa presented a clear barcoding gap between inter- and intraspecific values calculated, with the distinct exception of *G. gibbosum*, *G. eickeri*, G. aff. gibbosum and *G. ellipsoideum* which are closely related.

In conclusion, ITS phylogeny, in conjunction with sequence similarity and genetic distance measurements, do not fully support the delineation of some well-established *Ganoderma* taxa; instead, they advocate their inclusion in monophyletic units representing species complexes. On the other hand, *G. neojaponicum* (intraspecific values: 0.019 ± 0.009; 97.51 ± 1.44%), *Ganoderma* sp. A6 (0.028 ± 0.005; 97.24 ± 1.02), *Ganoderma* sp. C1 (0.029, 95.50%) and *Ganoderma* sp. D1 (0.020, 97.76%) seem to harbour cryptic variation and might correspond to more than one phylospecies. Relatively high intraspecies genetic distance values were also detected in *G. flexipes*, *G. mastoporum*, *G. mbrekobenum* and *Ganoderma* sp. D3 (Fig. 8), where terminal (often well-supported) subgroups were formed according to the ITS phylogeny (Fig. 4).

### Notes on *Ganoderma* biogeography and host range

Members of the genus *Ganoderma* exhibit relatively-complex microanatomy (Ryvarden 1991) and low levels of sequence divergence as evidenced in early studies on ribosomal DNA phylogeny (Moncalvo et al. 1995a). In addition, Moncalvo and Buchanan (2008) showed that neither the Northern Hemisphere nor the Southern Hemisphere *Ganoderma* taxa formed monophyletic groups. This is in conflict with a strict vicariant scenario and shows that global-scale vicariance models (Rosen 1978) are too simplistic to provide the sole explanation for the population structure and evolution of species like those of the genus *Ganoderma*. In this particular case, long-distance (inter-continental) dispersal seems to be more likely since it better explains the broad distribution of *Ganoderma* species evidenced from phylogenetic analyses, indicating strong geographic structure associated with allopatric divergence. On the other hand, little correlation was evident between phylogeny and host relationships (monocot and angiosperms, as well as gymnosperms) in *Ganoderma*, showing that host-based distribution cannot adequately explain the observed geographic pattern with some exceptions (e.g. the ‘palm-clade’, Cluster D.4).

Three main lineages of the genus were identified: Clade A, Clade B and Clades C through E. Clades A and E include taxa with a cosmopolitan distribution, while species of Clade B are distributed across the Holarctic region; species of Clades C occur in the Paleotropics and taxa of Clade D exhibit a pantropical distribution. Their subsequent analysis results are consistent with the hypothesis of a Northern Hemisphere origin (tropical Asia) for *Ganoderma* species with subsequent range expansions to the Southern Hemisphere and by colonisation of the Neotropics through long distance dispersal (Moncalvo and Buchanan 2008). Consequently, a large diversity of *Ganoderma* taxa is found in Asia; amongst the 80 *Ganoderma* species of the present study, the following occur in east – southeast Asia only: *G. leucocontextum* – *G. weixiensis*, *Ganoderma* sp. A1, *G. weberianum*, *Ganoderma* sp. A2, *Ganoderma* sp. A3, *Ganoderma* sp. A5, *G. flexipes*, *G. philippii*, *G. lingzhi*, *Ganoderma* sp. A6, *G. tropicum*, *Ganoderma* sp. A7, *G. mizoramense*, *G. multipileum*, *G. shanxiense*, *Ganoderma* sp. B2, *G. neojaponicum*, *G. casuarinicola*, *G. nasalanense*, *G. sinense*, *G. boninense*, *Ganoderma* sp. D3, *G. williamsianum*, *G. gibbosum*, *Ganoderma* sp. E4 and *G. mutabile*.

Indicative cases of *Ganoderma* species distribution include:

- Palearctic – Eurasian, Old World: *G. lucidum*, *G. resinaceum* and *G. adspersum* occur across Eurasia and share several common host plants, for example, the genera *Quercus*, *Salix*, *Populus*, *Abies* and *Larix*. A strictly European distribution is exhibited by *G. carnosum*, G. aff. carnosum and *G. pfeifferi*. Allopatric speciation seems to be under way between Eurasian *G. resinaceum* and Taiwanese collections corresponding to *Ganoderma* sp. A3. An Old Word distribution is also presented by species of the Cluster E.3, since each one of them has been reported exclusively from Africa (*G. knysnamense*), from Asia (*G. mutabile*) or from Europe (*G. pfeifferi*).
- East Asia – Malay Archipelago – Oceania: *G. weberianum* complex (*G. sichuanense* – *G. weberianum*) and *G. steyaertanum* (Clade A), *G. angustisporum* and *G. mastoporum* (Clade D) and *Ganoderma* sp. E3 and *Ganoderma* sp. E6 (Clade E). Particularly as regards *G. ellipsoideum*, its distribution extends to USA on the basis of sequences deriving from environmental samples.
- East Asia and South Africa (Paleotropic): All taxa of Clade C, as well as *G. hoehnelianum* (Cluster A.2), *G. wiiroense* (Cluster A.3), *G. angustisporum* and *Ganoderma* sp. D1 (Clade D). Moreover, *G. carocalcareum* and *G. austroafricanum* (Cluster A.2), *G. destructans* – *G. dunense* (Cluster A.3), *G. aridicola*, *G. enigmaticum – G. thailandicum*, *Ganoderma* sp. C1 and *Ganoderma* sp. C2 (Clade C), *G. cupreum*, *Ganoderma* sp. D1 and *G. ryvardenii* (Clade D), as well as *G. eickeri* (Clade E) have so far only been recorded in Africa.
- Holoarctic/Nearctic – Palearctic: Within Clade B, *G. applanatum* presents an inter-continental distribution indicating gene exchange between the Palearctic and the Nearctic regions through land bridges which were widely accepted as corridors for such transfers (Hibbett 2001; James et al. 2001; Zervakis et al. 2004; Geml et al. 2006; Geml et al. 2008; Matheny et al. 2009; Feng et al. 2012). The same pattern is presented by members of Cluster A.1. None of the Holarctic groups (Cluster A.1 and Clade B) shows any significant molecular divergence between collections from Europe and North America. Especially, the biogeographic pattern of Clade B evidences a recent common ancestral distribution in the Holoarctic region which explains the inter-continental distribution pattern of *G. applanatum*. In addition, allopatric speciation is evident at the terminal clades of the Cluster E.5, where Eurasian and east Asian collections of *G. adspersum* and G. aff. adspersum, respectively, are well-separated from their sister Nearctic *Ganoderma* sp. E7. A Nearctic distribution is also presented by *G. curtisii* (Cluster A.3).
- East Asian – North American: Several biogeographic studies evidenced migration of fungi from east Asia to North America via the Bering Land Bridge route (Wu and Mueller 1997; Wu et al. 2000; Chapela and Garbelotto 2004; Geml et al. 2006). *Ganoderma* species exhibiting an east Asian – northeast American disjunction are grouped within the *G. lingzhi* and *G. curtisii* – *G. ravenelii* complex (Cluster A.3). The same pattern is present in several sister clades within Cluster A.2, for example, the *G. sessile* – *G. polychromum* complex, since *G. sessile* shows a broad distribution in southeast Asia and America.
- Neotropical: *G. mexicanum* – *G. parvulum* complex, G. aff. polychromum (Cluster A.2), *G. tuberculosum* and *G. martinicense* (Cluster A.3), *Ganoderma* sp. D2 (Clade D) and *Ganoderma* sp. E1 and sp. E2 (Clade E). As regards other species occurring in the Americas only, *G. curtisii* (Cluster A.3), *G. zonatum* (Clade D) and *Ganoderma* sp. E7 (Clade E) are confined to North America. The closely-related *G. podocarpense* and *G. chocoense* (Clade E) were reported only in Central America, whereas *Ganoderma* sp. A4, *G. concinnum* and *G. multiplicatum* (Cluster A.3), *G. orbiforme* (Clade D) and *Ganoderma* sp. E5 (Clade E) were recorded in South America.
- Southern Hemisphere: The phylogenetic analysis of Southern Hemisphere species and complexes (species in Clusters A.2 and A.3, Clusters D.3 and D.4 and E.2 and E.4) indicated a restricted gene flow apparently due to geographic isolation, although episodic long-distance dispersal still occurs (Moncalvo and Buchanan 2008). The sister group relationships amongst the tropical southeast Asian species, South American and African species are not surprising since the two continents were connected after the collision of the African, Australian and Asian plates (McElhinny and Embleton 1974; Hall 2002). In all clades deriving from this analysis, several groups of closely-related taxa were identified, for example, the *G. mexicanum* – *G. parvulum* complex and the group of *G. hoehnelianum*, *G. austroafricanum* and *G. carocalcareum*.

The majority of *Ganoderma* species were collected on angiosperm hosts, 41 species on eudicots and 19 species on monocots, while another 18 species were reported on gymnosperms (Table 1 and Suppl. material 1: Table S2). Seven species were collected on both eudicot and monocots (but not on gymonsperms), i.e. *G. sessile*, *G. zonatum*, *G. ryvardenii*, *Ganoderma* sp. E2, *G. gibbosum*, *Ganoderma* sp. E4 and *G. australe*. On the other hand, *G. lucidum*, *G. leucocontextum* – *G. weixiensis*, *G. ravenelii*, *G. enigmaticum* – *G. thailandicum*, *G. mastoporum*, *G. angustisporum*, and *G. adspersum* were noted on both eudicots and gymnosperms, while *G. multipileum*, *G. applanatum*, *G. aridicola*, *G. casuarinicola*, *G. mbrekobenum*, G. aff. gibbosum and *G. ellipsoideum* were recorded on all three type of hosts. Cluster A.1 comprises species collected on eudicots and gymnosperms; two of them (*G. carnosum* and *G. oregonense*) were recorded on gymnosperm hosts only. Species of Clusters A.2 and A.3 present a marked preference for eudicots; only *G. sessile* (one record), *G. lingzhi* and *G. multipileum* are also reported from monocots, whereas *G. flexipes*, *G. ravenelii* and *G. multipileum* are reported on gymnosperms as well. In Clade B, host data are available only for *G. applanatum*; the respective specimens were collected on a broad range of hosts. In addition, this is the only species for which the host range is expanded to include also monocots (i.e. *Phoenix canariensis* and *Tradescantia zanonia*) as an outcome of including environmental samples in this study. However, these data should be treated with caution since the respective material was obtained from plant leaves. Clades C and D comprise species collected on a broad range of eudicots, monocots and gymnosperms. Cluster D.4 includes five species with a preference for monocots, three of them growing strictly on this particular host type (i.e. *G. boninense*, *Ganoderma* sp. D2 and *Ganoderma* sp. D3). Regarding Clade E, the majority of species were recorded on eudicots and only three on gymnosperms (i.e. *G. adspersum*, *G. aff. gibbosum* and *G. ellipsoideum*).

### Erroneous *Ganoderma* sequences labelling in public depositories

As previously stated, one of the major obstacles for exploiting sequences present in public depositories is that many of them are inaccurate; errors in labelling of metadata were estimated to correspond to as much as 20% – or even 30% according to a recent report – of GenBank accessions including also recent deposits (Vilgalys 2003; Nilsson et al. 2006; Schoch et al. 2014; Hofstetter et al. 2019). The outcome of our study shows that the extent of the problem is even more pronounced in the case of *Ganoderma* entries and it could be clearly exemplified when pertinent data are presented either (a) by the most commonly-used names under which *Ganoderma* sequences were deposited or (b) by the names used much less than they ought. As regards the former, the most widely-used names were *G. lucidum* [482 entries; 375 (78%) were subsequently grouped in 24 species other than *G. lucidum*)], *G. australe* [(212 entries; 185 (87%) of them were found to correspond to 15 species other than *G. australe*)] and *G. resinaceum* [(177 entries; 72 (41%) of them were found to represent five species other than *G. resinaceum*)]. On the basis of the metadata analysis performed, *G. lucidum* is actually represented by 107 entries under this name (meaning that only 22% of the initial identifications were correct), *G. australe* by 27 entries (13% correct) and *G. resinaceum* by 105 entries (59% correct while 34% are erroneously labelled as “*G. sessile*”). On the other hand, 22 out of 23 sequences initially deposited as *G. multipileum* were correct; however, the end result revealed that 243 entries belong to this species (the extra entries were originally labelled either as *Ganoderma* sp. or as “*G. lucidum*”). A similar case is demonstrated by *G. lingzhi*; 333 out of 337 sequences deposited under this name were correct, but the final number of entries which correspond to this species is 615 (the additional sequences were mainly labelled as “*G. lucidum*”, *Ganoderma* sp. or as “*G. sichuanense*”). Moreover, *G. applanatum* species consists of 424 entries; the majority of them (269, 63%) derive from environmental samples (Table 1, Suppl. material 1: Tables S2, S5). In general, sequences originating from environmental samples correspond to ca. 9% of the total *Ganoderma* dataset examined and represent 32 species identified in the frame of the present study (Suppl. material 1: Table S5). In three notable cases, the known distribution of *Ganoderma* species appears to be expanded to other continents thanks to the information provided by such type of data, i.e. the Nearctic *G. oregonense* in Europe (Estonia), the neotropical *G. cupreum* in India and the Australasian *G. ellipsoideum* in USA. All relevant data concerning the identity of environmental samples, as well as the correct names of mis-annotated sequences, are included in Suppl. material 1: Tables S5, S6.

## Conclusions

The study of a large dataset comprising almost four thousand ITS entries proved to be valuable in obtaining a significant amount of phylogenetic information which contributed to elucidating the status of *Ganoderma* species. In addition, it contributed to establishing robust relationships amongst the majority of them, while it also revealed limitations in the use of ITS (alone) to assess certain taxa which have to be addressed through a multigene approach. However, it is interesting that the outcome of recent publications employing more than one marker (by focusing on particular groups in the genus, for example, Loyd et al. 2018; Cabarroi-Hernández et al. 2019; Tchotet Tchoumi et al. 2019) is congruent with the phylogeny obtained from the present meta-analysis. Furthermore, the results of this work demonstrated that even recent sequence deposits in public databases are associated with a remarkably high number of misidentifications or errors. The very high variability/plasticity in morphological characters, the improper use of terms describing anatomical features, the existence of ambiguous synonyms or misapplied names, the non-uniformity of taxonomic criteria adopted by researchers and the expanding number of non-experts working on (and sequencing) *Ganoderma* material are some of the reasons behind the unreliability of such information. Consequently, interpretation of specimen identity vis-à-vis BLAST results often leads to mistaken conclusions. Therefore, a phylogenetic framework is preferable to identify new material since taxonomic determinations, based solely on BLAST results, are often erroneous and should be performed with great care. The effectiveness of DNA barcoding greatly depends on establishing reference sequences from validated (authentic) type specimens after careful evaluation of phylogenetic data. Especially in the case of the genus *Ganoderma*, the holotype of many species is either missing or destroyed, thus the need for epitypification is apparent. In addition, as the present study assessed, annotations of submitted sequences are frequently fragmentary and important information is lacking (e.g. geographic origin, even for those corresponding to newly-described taxa), which is unfortunately widespread in GenBank submissions pertaining to fungal specimens (Schoch et al. 2014; Durkin et al. 2020). A special mention should also be made to the usefulness of morphological characters in *Ganoderma* specimens, which (for the reasons previously explained) must be evaluated with caution and preferably in conjunction with other approaches (including DNA sequencing) to provide data suitable for resolving taxonomic issues and at inferring robust conclusions on species concepts.

At a more general level, this study evidences that significant – yet largely untapped – mycological explanatory power resides in the public DNA sequence corpus and we hope that other mycologists will start scrutinising the sequence data available for their fungal groups of expertise. Our results also demonstrated that the so-called environmental sequences – usually ignored in a taxonomic/phylogenetic context – should be included in such pursuits (cf. Ryberg et al. 2008) since they were found to confer valuable information.

## Supplementary materials

Supplementary material 1 Tables S1–S6 Data type species data Explanation note **Table S1.** Information about the *Ganoderma* material/specimens analyzed for the first time in the frame of this work: species name (the initial name appears in parenthesis when different from the one determined in the present study), specimens code, plant host, geographic origin, collector and date of collection, type of material examined (H: herbarium specimen, C: pure culture) and GenBank accession number of the generated ITS sequences. **Table S2.** Detailed information on the *Ganoderma*ITS sequences used in this study. **Table S3.***Ganoderma* sequences excluded from the analysis since they were either erroneously labelled as *Ganoderma* or whose identity could not be reliably resolved. **Table S4.***Ganoderma* sequences (642) included in the data analysis but excluded from the trees inferred (Figs 4 to 7, and Figs S2a to Fig. S2f) due to overrepresentation of certain species (i.e., > 100 singletons for *G. lingzhi*, *G. multipileum* and *G. applanatum*) or to their particularly high heterogeneity. **Table S5.** Information about the 354 environmental samples analyzed and their identity on the basis of the outcome of the present study. **Table S6.***Ganoderma* entries (3908) analyzed: initial identification (as labelled in GenBank/ENA/DDBJ and UNITE databases), accession number, corresponding UNITE DOI and taxon name on the basis of the outcome of this study. https://binary.pensoft.net/file/481984This dataset is made available under the Open Database License (http://opendatacommons.org/licenses/odbl/1.0/). The Open Database License (ODbL) is a license agreement intended to allow users to freely share, modify, and use this Dataset while maintaining this same freedom for others, provided that the original source and author(s) are credited.Vassiliki Fryssouli, Georgios I. Zervakis, Elias Polemis, Milton A. Typas

Supplementary material 2 Figure S1 Data type molecular data Explanation note Hypervariable regions of potential diagnostic value in ITS1 and ITS2 spacers for the *Ganoderma* taxa appearing in Table 1, Supplementary Table S2 and Fig. 3. https://binary.pensoft.net/file/481985This dataset is made available under the Open Database License (http://opendatacommons.org/licenses/odbl/1.0/). The Open Database License (ODbL) is a license agreement intended to allow users to freely share, modify, and use this Dataset while maintaining this same freedom for others, provided that the original source and author(s) are credited.Vassiliki Fryssouli, Georgios I. Zervakis, Elias Polemis, Milton A. Typas

Supplementary material 3 Figure S2a Data type molecular data Explanation note Phylogenetic reconstruction of the genus *Ganoderma* inferred from ML analysis based on ITS sequence data (pDS1a; Table 2) for Clade A, Cluster A.1. ML bootstrap values (BS) ≥ 65% and Bayesian posterior probabilities (BPP) ≥ 0.95 are shown. https://binary.pensoft.net/file/481986This dataset is made available under the Open Database License (http://opendatacommons.org/licenses/odbl/1.0/). The Open Database License (ODbL) is a license agreement intended to allow users to freely share, modify, and use this Dataset while maintaining this same freedom for others, provided that the original source and author(s) are credited.Vassiliki Fryssouli, Georgios I. Zervakis, Elias Polemis, Milton A. Typas

Supplementary material 4 Figure S2b Data type molecular data Explanation note Phylogenetic reconstruction of the genus *Ganoderma* inferred from ML analysis based on ITS sequence data (pDS1b; Table 2) for Clade A, Cluster A.2. ML bootstrap values (BS) ≥ 65% and Bayesian posterior probabilities (BPP) ≥ 0.95 are shown. https://binary.pensoft.net/file/481987This dataset is made available under the Open Database License (http://opendatacommons.org/licenses/odbl/1.0/). The Open Database License (ODbL) is a license agreement intended to allow users to freely share, modify, and use this Dataset while maintaining this same freedom for others, provided that the original source and author(s) are credited.Vassiliki Fryssouli, Georgios I. Zervakis, Elias Polemis, Milton A. Typas

Supplementary material 5 Figure S2c Data type molecular data Explanation note Phylogenetic reconstruction of the genus *Ganoderma* inferred from ML analysis based on ITS sequence data (pDS1c; Table 2) for Clade A, Cluster A.3. ML bootstrap values (BS) ≥ 65% and Bayesian posterior probabilities (BPP) ≥ 0.95 are shown. https://binary.pensoft.net/file/481988This dataset is made available under the Open Database License (http://opendatacommons.org/licenses/odbl/1.0/). The Open Database License (ODbL) is a license agreement intended to allow users to freely share, modify, and use this Dataset while maintaining this same freedom for others, provided that the original source and author(s) are credited.Vassiliki Fryssouli, Georgios I. Zervakis, Elias Polemis, Milton A. Typas

Supplementary material 6 Figure S2d Data type molecular data Explanation note Phylogenetic reconstruction of the genus *Ganoderma* inferred from ML analysis based on ITS sequence data (pDS2 & pDS3; Table 2) for Clades B & C. ML bootstrap values (BS) ≥ 65% and Bayesian posterior probabilities (BPP) ≥ 0.95 are shown. https://binary.pensoft.net/file/481989This dataset is made available under the Open Database License (http://opendatacommons.org/licenses/odbl/1.0/). The Open Database License (ODbL) is a license agreement intended to allow users to freely share, modify, and use this Dataset while maintaining this same freedom for others, provided that the original source and author(s) are credited.Vassiliki Fryssouli, Georgios I. Zervakis, Elias Polemis, Milton A. Typas

Supplementary material 7 Figure S2e Data type molecular data Explanation note Phylogenetic reconstruction of the genus *Ganoderma* inferred from ML analysis based on ITS sequence data (pDS4; Table 2) for Clade D. ML bootstrap values (BS) ≥ 65% and Bayesian posterior probabilities (BPP) ≥ 0.95 are shown. https://binary.pensoft.net/file/481990This dataset is made available under the Open Database License (http://opendatacommons.org/licenses/odbl/1.0/). The Open Database License (ODbL) is a license agreement intended to allow users to freely share, modify, and use this Dataset while maintaining this same freedom for others, provided that the original source and author(s) are credited.Vassiliki Fryssouli, Georgios I. Zervakis, Elias Polemis, Milton A. Typas

Supplementary material 8 Figure S2f Data type molecular data Explanation note Phylogenetic reconstruction of the genus *Ganoderma* inferred from ML analysis based on ITS sequence data (pDS5; Table 2) for Clade E. ML bootstrap values (BS) ≥ 65% and Bayesian posterior probabilities (BPP) ≥ 0.95 are shown. https://binary.pensoft.net/file/481991This dataset is made available under the Open Database License (http://opendatacommons.org/licenses/odbl/1.0/). The Open Database License (ODbL) is a license agreement intended to allow users to freely share, modify, and use this Dataset while maintaining this same freedom for others, provided that the original source and author(s) are credited.Vassiliki Fryssouli, Georgios I. Zervakis, Elias Polemis, Milton A. Typas
