# Supplementary material for: A global meta-analysis of ITS rDNA sequences from material belonging to the genus Ganoderma (Basidiomycota, Polyporales) including new data from selected taxa
Source: MycoKeys. 2020 Dec 1;75:71–143. doi: 10.3897/mycokeys.75.59872 (PMC7723883; doi:10.3897/mycokeys.75.59872)
Supplement: Supplementary material 1 — Tables S1–S6 [file mycokeys-75-071-s001.pdf]

Supplementary Table S1. Information about the *Ganoderma* material/specimens analyzed for the first time in the frame of this work: species name (the initial name appears in parenthesis when different from the one determined in the present study), specimens code, plant host, geographic origin, collector and date of collection, type of material examined (H: herbarium specimen, C: pure culture) and GenBank accession number of the generated ITS sequences.

a/a	Species	Specimen Code	Plant Host	Geographic Origin	Collector, Date	Type of Material	GenBank Accession No.
1	*G. adspersum*	ACAM 2012-0050	*Populus alba* × *tremula*	Greece, Attiki, Tatoi	E. Polemis, 10 Oct 2012	H	MG706203
2	*G. adspersum*	ACAM A113	*Alnus glutinosa*	Greece, Andros Island	E. Polemis, 17 Dec 2005	H	MG706204
3	*G. adspersum*	ACAM DD1853	*Amygdalus communis*	Greece, Agios Efstratios Island	D. Dimou, 8 Aug 2005	H	MG706205
4	*G. adspersum*	ACAM DD2486	*Abies cephalonica*	Greece, Attiki, Parnitha Mt.	D. Dimou, 9 Nov 2006	H	MG706206
5	*G. adspersum*	LGAM 440	*Abies cephalonica*	Greece, Euboea Island	E. Polemis, 15 Nov 2009	C	MG706207
6	*G. adspersum*	ACAM 2010-0015	*Platanus orientalis*	Greece, Euboea Island	E. Polemis, 17 Oct 2010	H	MG706208
7	*G. adspersum*	ACAM 2013-0011	*Abies cephalonica*	Greece, Euboea Island	E. Polemis, 18 May 2013	H	MG706209
8	*G. adspersum*	MUCL 39327	on dead stump	France, Indre, St Chartier	C. Decock, 1995	C	MG706210
9	*G. adspersum*	NAGREF 341	n/a	Greece	n/a	C	MG706211
10	*G. adspersum*	NAGREF 342	n/a	Greece	n/a	C	MG706212
11	*G. applanatum*	ACAM DD2119	*Fagus sylvatica*	Greece, Phthiotida, Oxya Mt.	D. Dimou, 23 Oct 2005	H	MG706213
12	*G. applanatum*	FBE 13945	*Salix* sp.	Bulgaria	2 May 1978	H	MG706214
13	*G. applanatum*	ACAM 2015-0057	*Fagus sylvatica*	Greece, Phthiotida, Oxya Mt.	E. Polemis, 11 Nov 2015	H	MG706215
14	*G. carnosum*	ACAM DD0519	*Pinus nigra*	Greece, Thessaloniki, Chortiatis	D. Dimou, 22 Nov 1999	H	MG706216
15	*G. carnosum*	LGAM 1642	*Abies cephalonica*	Greece, Messinia, Taygetos Mt.	E. Polemis, 14 Dec 2000	C	MG706217
16	*G. carnosum*	ACAM DD1243	*Abies cephalonica*	Greece, Attiki, Parnitha Mt.	D. Dimou, 9 Sep 2002	H	MG706218
17	*G. carnosum*	MUCL 39429	on dead stump	France, Vosges, Lac de Blanchemer	C. Decock, 1995	H	MG706219
18	*G. carnosum*	MUCL 49464	on dead stump	France, Hautes Pyrénées	C. Decock, 2007	H	MG706220
19	*G. lingzhi* (*G. tsugae*)	LGAM 605	n/a	commercial strain	n/a	C	MG706221
20	*G. lingzhi* (*G. lucidum*)	LGAM 808	n/a	commercial strain	n/a	C	MG706222

21	*G. lucidum*	ACAM A1180	*Quercus ilex*	Greece, Andros Island	E. Polemis, 3 Dec 2012	H	MG706223
22	*G. lucidum*	FBE 10458	*Quercus* sp.	Bulgaria	20 Sep 1972	H	MG706224
23	*G. lucidum*	FBE 14610	*Quercus* sp.	Bulgaria	Sep 1978	H	MG706225
24	*G. lucidum*	LGAM 408	*Quercus coccifera*	Greece, Ileia	G. Koutrotsios, 26 Dec 2006	C	MG706226
25	*G. lucidum*	ACAM 2013-0022	*Quercus coccifera*	Greece, Kastoria, Vitsi	G. Koutrotsios, 12 Jul 2013	H	MG706227
26	*G. lucidum*	LGAM 490 (9720)	n/a	commercial strain	n/a	C	MG706228
27	*G. lucidum*	ACAM 2013-0062	*Quercus coccifera*	Greece, Messinia, Mani	G. Koutrotsios, 2 Dec 2013	H	MG706229
28	*G. lucidum*	MUCL 31549	on dead stump	France, Dordogne	C. Decock, 1991	C	MG706230
29	*G. lucidum*	CCBAS 707	n/a	Czech Republic	n/a	C	MG706231
30	*G. pfeifferi*	ACAM DD2118	*Fagus sylvatica*	Greece, Phthiotida, Oxya Mt.	D. Dimou, 23 Oct 2005	H	MG706232
31	*G. resinaceum*	ACAM 2012-0077	*Olea europaea*	Greece, Attiki, Votanikos	E. Polemis, 31 Oct 2012	H	MG706233
32	*G. resinaceum*	ACAM 2012-0099	*Laurus nobilis*	Greece, Attiki, Tatoi	E. Polemis, 10 Nov 2012	H	MG706234
33	*G. resinaceum*	ACAM 2013-0027	*Eriobotrya japonica*	Greece, Crete Island, Sternes	P. Fryssoulis, 20 Jun 2013	H	MG706235
34	*G. resinaceum*	ACAM 2013-0028	*Quercus ilex*	Greece, Ileia	D. Grigoropoulou, Oct 2013	H	MG706236
35	*G. resinaceum* (*G. lucidum*)	ACAM A645	*Platanus orientalis*	Greece, Andros Island	E. Polemis, 10 Aug 2003	H	MG706237
36	*G. resinaceum*	ACAM DD0863	*Schinus molle*	Greece, Attiki, Votanikos	D. Dimou, 15 Sep 2001	H	MG706238
37	*G. resinaceum* (*G. lucidum*)	ACAM N303	*Acer sempervirens*	Greece, Naxos Island	E. Polemis, 11 Oct 2005	H	MG706239
38	*G. resinaceum*	ACAM N399	*Alnus glutinosa*	Greece, Naxos Island	E. Polemis, 11 Oct 2005	H	MG706240
39	*G. resinaceum*	ACAM N401	*Quercus ithaburensis* spp. *macrolepis*	Greece, Naxos Island	E. Polemis, 11 Oct 2005	H	MG706241
40	*G. resinaceum*	CCBAS	n/a	Czech Republic	n/a	C	MG706242
41	*G. resinaceum*	FBE 14488	*Ulmus* sp.	Bulgaria	14 Jul 1977	H	MG706243
42	*G. resinaceum* (*G. pfeifferi*)	LGAM 306	*Fagus sylvatica*	Greece, Phthiotida, Oxya Mt.	D. Dimou, 4 Jun 2000	C	MG706244
43	*G. resinaceum*	ACAM DD2380	n/a	Greece, Phthiotida, Kammena Vourla	D. Dimou, 20 Oct 2006	H	MG706245
44	*G. resinaceum*	LGAM 353	n/a	Greece	n/a	C	MG706246

45	*G. resinaceum*	ACAM DD2491	*Salix babylonica*	Greece, Crete Island, Herakleio	D. Dimou, 10 Nov 2006	H	MG706247
46	*G. resinaceum*	ACAM 2013-0015	*Ceratonia siliqua*	Greece, Attiki, Votanikos	E. Polemis, 18 Dec 2009	H	MG706248
47	*G. resinaceum*	LGAM 448	*Olea europaea*	Greece, Phthiotida, Ypati	D. Dimou, n/a	C	MG706249
48	*G. resinaceum*	ACAM A990	*Platanus orientalis*	Greece, Andros Island	E. Polemis, 6 Nov 2010	H	MG706250
49	*G. resinaceum*	ACAM 2013-0013	*Quercus frainetto*	Greece, Euboea Island, Telethrio	G. Koutrotsios, 26-May-2013	H	MG706251
50	*G. resinaceum*	LGAM 565	*Populus alba* × *P. tremula*	Greece, Attiki, Tatoi	E. Polemis 17 Oct 2012	C	MG706252
51	*G. resinaceum* (*Ganoderma* sp.)	LGAM 566	n/a	Greece, Euboea Island, Istiaia	n/a	C	MG706253
52	*G. resinaceum* (*Ganoderma* sp.)	LGAM 567	n/a	Greece	n/a	C	MG706254
53	*G. resinaceum* (*G. pfeifferi*)	LGAM 568	*Fagus sylvatica*	Greece, Phthiotida, Oxya Mt.	23-Oct-2005	C	MG706255
54	*G. resinaceum*	MUCL 51491	n/a	Belgium	n/a	H	MG706256

n/a: information not available

Supplementary Table S2. Detailed information on the *Ganoderma* ITS sequences used in this study. Species (marked in bold typeface) appear as they are presented in Clades and/or Clusters according to the outcome of the phylogenetic analysis (Figs. 3 to 7 and Figs. S2a to S2f), and are followed by the names used in the original labelling of sequences together with the number of sequences examined per each name (in parentheses), accompanied with any available information pertaining to the associated host/substrate. Then, ASVs and singletons examined per species are presented together with the following information placed in three columns (from left to right): (a) the name under which a representative sequence (i.e., one with the same name to the majority of those grouped within the particular ASV) was originally deposited at GenBank/ENA/DDBJ and UNITE databases, its accession code no. and the number of other identical sequences existing within the same ASV (in parentheses), (b) the accession code no. of all identical sequences which form part of the same ASV, and (c) the geographic origin (when available) of the material from which the respective sequences were obtained. Sequences generated in this study appear in bold typeface, while underlined sequences are those deriving from type material. DOI's from UNITE's database are quoted in brackets for the new phylospecies proposed in this study (species hypotheses considered at 99% similarity level).

**CLADE A**		
***G. shanxiense* (2)**<br>as *G. shanxiense* (2)<br>from *Quercus* sp.		
G. shanxiense MK764268		China
G. shanxiense MK764269		China

**CLADE A, Cluster A.1**		
***G. tsugae* (54)**<br>as *G. tsugae* (45), *G. lucidum* (3), *G. valesiacum* (2), *G. ahmadii* (1), *G. carnosum* (1), *Ganoderma* sp. (1), *Polyporus tsugae* (1)<br>from *Abies concolor*, *Abies* sp., *Dalbergia sissoo, Pseudotsuga menziesii, Tamarindus indica, Tetrameles nudiflora, Tsuga canadensis*		
G. tsugae MG654323 (25)	DQ206985, DQ425003, FJ655478, KC514828, KF605617, KJ140748, MG231528, MG654325, MG654328, MG654330, MG654333, MG654334, MG654336, MG654337, MG654341, MG654343, MG654344, MG654345, MG654346, MG654347, MG654348, MG654349, MG654350, MH856317	Canada, India, USA (KY, MI, NC, NH, WI)
G. tsugae MG654329 (4)	MG654338, MG654339, MG654340	USA (WI)
G. tsugae MG654331 (4)	DQ425010, MG654332, MT357080	USA (NM, AZ)
G. tsugae KJ143920 (2)	KF605618	USA (CT)
G. tsugae MG654321 (2)	MG654322	USA (AZ)
G. ahmadii Z37047/Z37098		Pakistan
G. tsugae X78735/X78756[1]		USA
G. tsugae Z37054/Z37079		Canada
G. valesiacum Z37056/Z37081		UK
Singletons (13)*	DQ425002, FJ655479, FJ655480, JQ520215, MG654324, MG654326, MG654327, MG654335, MG910999, MH277960, MH277961, Z37055/Z37080, Z37057/Z37082	Canada, Germany, India, USA (AZ, MI, MN, NY, VT, WI)

***G. oregonense* (27)**<br>as *G. oregonense* (15), *G. carnosum* (5), *G. tsugae* (4), *G. lucidum* (2), uncultured soil fungus (1)<br>from *Tsuga heterophylla*, coniferous tree, soil		
G. oregonense MG654189 (17)	EU486458, JQ520185[2], JQ520195, JQ520216, KF605620, KF605621, KJ143919, KJ146707, MG654190, MG654191, MG654192, MG654194, MG654342, MH277958, MH277959, UDB0287378	Canada, Estonia, USA (AK, CA, CT, OR, WI)
G. oregonense MG654193 (2)	MT196417	USA (CA)
G. oregonense JQ781875		USA (WA)
G. oregonense JQ781876		USA (OR)
Singletons (6)*	JN222419, KF605619, MG654195, Z37052/Z37076[2], Z37060/Z37100, Z37061/Z37101	Canada, USA (WA)

***G. carnosum* (26)**<br>as *G. carnosum* (22), *G. lucidum* (4)		

from *Abies cephalonica*, *Pinus nigra*		
G. carnosum MK415244 (6)	MK415261, MK415266, MK415267, MK415276, MK415314	Slovakia
G. carnosum **MG706220** (5)	KF605622[3], KF605625, KU572492[3], KU572494	Belgium, Czech Republic
G. carnosum **MG706216** (4)	**MG706217, MG706218**, KF605626	Greece
G. carnosum **MG706219** (3)	GU731557, JQ627587	Belgium, Poland
G. carnosum KU572493[4] (2)	KF605624[4]	Czech Republic
Singletons (6)*	JN588574, JN588578, KC311369, KF605623, MK415287, MK415312	France, Slovakia, Slovenia
***G.* aff. *carnosum* (4)**<br>as uncultured soil fungus (2), *G. carnosum* (1), *G. lucidum* (1)<br>from soil		
G. carnosum AF094511/AF044490		UK
G. lucidum AY884175		UK
uncultured soil fungus UDB0609440		Estonia
uncultured soil fungus UDB0632514		Estonia
***G. lucidum* (152)**<br>as *G. lucidum* (107), uncultured soil fungus (27), *G. tsugae* (12), *G. oerstedii* (3), *G. oregonense* (1), *G. valesiacum* (1), *Ganoderma* sp. (1)<br>from *Acacia* sp., *Aesculus hippocastanum*, *Betula* sp., *Cassia spectabilis*, *Corylus* sp., *Dendrocalamus* sp., *Fagus* sp., *Larix* sp., *Melia azadirachta*, *Pinus* sp., *Platanus acerifolia*, *Prunus armeniaca*, *Pterocarpus* sp., *Quercus coccifera*, *Q. ilex*, *Quercus* sp., *Tamarindus indica*, *Terminalia bellerica*, *Terminalia* sp., dead wood, soil		
G. lucidum **MG706224** (64)	**MG706225, MG706226, MG706228, MG706230, MG706231,** DQ424972, DQ425011, EU498090, JN176899, JN222422, JN588575, JQ781851, JQ781852, JQ781853, KF605615, KF605616, KJ143909, KJ143912, KJ509596, KJ857252, KP941446, KP941448, KU310900, KU863090, KU863091, KU863092, KU863093, KU863094, MF419230, MF581079, MG279195, MG279196, MG279197, MG654067, MG654070, MG654071, MG654072, MG654073, MG711807, MG911000, MH294309, MH294321, MN396400, MT225392, UDB018875, UDB031872, UDB0109711, UDB0111653, UDB0127747, UDB0246137, UDB0255944, UDB0280598, UDB0282139, UDB0290411, UDB0438898, UDB0498134, UDB0539428, UDB0549396, UDB0552851, UDB0562842, UDB0591478, UDB0735359, UDB0735496	Algeria, Belgium, Bulgaria, China (JL), Czech Republic, Estonia, Finland, France, Greece, Italy, Russia, Sweden, USA (CA, UT), commercial
G. lucidum KJ143910 (10)	MG279181, MG279182, MN396338, MN396339, MN396340, MN396341, MN401402, MN401403, MN401404	China (YN, SC), Thailand
G. lucidum FJ463903 (6)	FJ463911, FJ463916, FJ463922, FJ463927, FJ463930	India
G. lucidum JN588572 (4)	JN588573, KP941444, KP941445	Armenia
G. lucidum KJ143911 (3)	JN176895, JN176897	Italy, UK
G. lucidum MG279161 (3)	FJ216424, MG279162	
G. lucidum **MG706223** (3)	**MG706229,** KX765192	Greece, Iran
G. lucidum **MG706227** (2)	UDB0713002	Estonia, Greece
G. oerstedii X78739/X78760		Argentina
G. oerstedii Z37058/Z37083		Argentina
G. lucidum MK415270 (3)*	MK415271, MK415272	Slovakia
G. lucidum AM906058 (2)*	MT510202	Iran, Italy
G. lucidum FJ463914 (2)*	FJ463918	India
G. lucidum JX082330 (2)*	GU731558	France
G. lucidum KT318593 (2)*	KT318594	
G. lucidum KT343304 (2)*	KT343314	Greece, Iran
G. lucidum MK554777 (2)*	MK554779	France
Singletons (40)*	AM269773, AY884176, JN588576, JN588577, JQ520186, JQ666509, JQ781854, JX162769, KC222321, KC222322, KF605614, KF963254, KJ411559, KT318592, KT318595, KT343297, KT343301, KT805317, MF419231, MG654066, MG654068, MG654069, MG835865, MH160071, MH861340[5], MK050595, MK050948, MK415269, MK415285, MK415286, UDB0118812, UDB0245628, UDB0383764, UDB0394723, UDB0502758, UDB0562707, UDB0609431, X78748/X78769, Z37049/Z37099[5], Z37096/Z37073,	China (JL), Estonia, France, India, Iran, Italy, Norway, Russia, Slovakia, South Korea, Spain, UK, USA (CA), commercial
***G. leucocontextum – G. weixiensis* (33)**<br>as *G. leucocontextum* (24), *G. lucidum* (3), *Ganoderma* sp. (3), *G. weixiensis* (2), *G. carnosum* (1)<br>from *Cyclobalanopsis glauca*, *Pinus* sp., *Rosa indica*, decaying wood		
<u>*G. weixiensis* MK302444/NR_166271[6] (9)</u>	KJ027608, KM396271, KU572485, KX055559, MK302445[7], MN396317, MN396318, MT007546	China (YN, Tibet)

G. leucocontextum KU863084 (8)	KU863085, KU863086, KU863087, KU863088, KX055558, MH294311, MN134012	China, Pakistan
G. leucocontextum KP676024 (2)	KP676025	China (SC, Tibet)
G. leucocontextum KF011548		China (Tibet)
Ganoderma sp. MG066446[6] (2)*	MG066447[7]	
Singletons (11)*	AB763348, KC222320, KC222323, KF372586, KJ027607, KJ027809, KM396272, KX262903, MH294310, MN134518, MK713839	China (SC, Tibet), Nepal, Pakistan

**CLADE A, Cluster A.2**		
***G. austroafricanum* (2)**<br>as *G. austroafricanum* (1), *G.* aff. *austroafricanum* (1)<br>from *Jacaranda mimosifolia*		
G. austroafricanum KM507324		South Africa
G. aff. *austroafricanum* MH571693		South Africa
***G. hoehnelianum* (14)**<br>*G. hoehnelianum* (9), *Ganoderma* sp. (5)<br>from dead and living wood		
G. hoehnelianum JN383980[9] (6)	JX195203, KU219988, KU219989[9], MG279178, MN396316	China (GX, YN), Myanmar
Ganoderma sp. MK531812 (3)	MK554787, MK554789	Gabon
Ganoderma sp. MK554781 (2)	MK554788	China
G. hoehnelianum MG279160		China (GX)
Singletons (2)*	JX195202, MH106881	China (HI)
***G. weberianum* (11)**<br>as *G. weberianum* (9), *G. microsporum* (1), *G. sichuanense* (1)<br>from *Mangifera* sp., *Salix babylonica*		
G. weberianum JQ520219[10] (2)	MH855780[10]	Philippines
G. weberianum MK603804[10] (2)	MK603805	Philippines
G. microsporum X78751/X78772		Taiwan
G. weberianum Z37064/Z37086		Taiwan
Singletons (5)*	KX055552, MH864975, MN622782, MK554790, X78734/X78755[10]	Philippines, Taiwan
***G. sichuanense* (19)**<br>as *G. sichuanense* (9), *G. weberianum* (4), *G. lucidum* (2), *G. tenue* (2), *Ganoderma* sp. (1), uncultured soil fungus (1)<br>from dead angiosperm/wood, soil		
G. tenue DQ424977[11] (3)	DQ424975[12], MN523252	China
G. tenue DQ424978[11] (2)	DQ424976[12]	
G. sichuanense JQ781877		China (SC)
G. weberianum AY569451		Australia
Singletons (12)*	EU239393/4, JF915411, JQ781878, KJ654377, MN523250, MN523251, MN523253, MN523254, MN523255, MN523256, MN523258, UDB0757071	Australia, China (GD, HI), India
***G. carocalcareum* (13)**<br>as *Ganoderma* sp. (8), *G. weberianum* (3), *G. carocalcareum* (2)<br>from *Acacia* sp., *Anthocleista nobilis*		
Ganoderma sp. MK554768 (5)	MK554773, MK554776, MK554780, MK554785	Cameroon, Gabon
G. weberianum JN105703 (3)	JN105704, JN105705	Cameroon
G. carocalcareum EU089969 (2)	EU089970	Cameroon
Ganoderma sp. MK603806		Gabon
Singletons (2)*	MK554774, MK554784	Cameroon, Gabon
***Ganoderma* sp. A1 (17) [SH1740420.08FU, SH1740444.08FU, SH1740445.08FU]**<br>as *G. weberianum* (17)<br>from *Cassia fistula*, *C. spectabilis*, *Delonix regia*, *Ficus benghalensis*, *Mangifera indica*, *Melia azadirachta*, *Tamarindus indica*, *Terminalia bellerica*, *Tetrameles nudiflora*		
G. weberianum FJ491967 (6)	FJ491968, FJ491971, FJ491973, FJ491976, FJ491979	India
G. weberianum FJ491970 (3)	FJ491974, FJ491978	India
G. weberianum FJ491972 (2)	FJ491987	India
G. weberianum FJ491975 (2)	FJ491977	India
Singletons (4)*	FJ491969, FJ491986, FJ491988, FJ655481	India
***G.* aff. *weberianum* (5)**<br>as *G. weberianum* (2), *G.* cf. *weberianum* (1), *G. subamboinense* (1), *Ganoderma* sp. (1)		

from *Caesalpinia ferrea*		
G. weberianum GU726934 (2)	GU726935	India
G. cf. *weberianum* KC222319		China
G. subamboinense KU569546		Brazil
Ganoderma sp. MK571161		China
**_G. mexicanum_ (16)**		
as *G. mexicanum* (6), *G. subamboinense* var. *laevisporum* (5), *G. subamboinense* (2), *G. sessiliforme* (1), *G. tuberculosum* (1), *G. weberianum* (1)		
from *Cassia multijuga*, *Platanus acerifolia*		
G. mexicanum MK531811 (2)	MK531823	Martinique, Mexico
G. subamboinense var. *laevisporum* JQ520205 (2)	DQ425006	Argentina
G. mexicanum MK531818		Martinique
G. sessiliforme AH008108		Brazil
G. subamboinense X78736/X78757		Argentina
Singletons (9)*	AH008107, DQ425005, MG654370, MG654373, MK531815, MK531819, MK531820, MK554771, MT232638,	Argentina, Martinique, Mexico, USA (FL)
**_G. parvulum_ (22)**		
as *G. parvulum* (10), *Ganoderma* sp. (4), *G. weberianum* (4), *G. subamboinense* var. *laevisporum* (2), *G. stipitatum* (1), *G. subamboinense* (1)		
from *Pleiogynium timorense*, decaying wood		
G. parvulum MK554767 (3)	MK554782, MK554783	Cuba
G. parvulum MK531132 (2)	MK531813	Cuba, French Guiana
G. parvulum MK554769 (2)	MK554770	Cuba, French Guiana
G. subamboinense var. *laevisporum* MG654372 (2)	HM800731[8]	USA (FL)
G. subamboinense MK531824/MK531822		Brazil
Singletons (12)*	GU731560, JN637827, KU214242, KC884264[8], LT726730, LT726731, MG654371, MK531814, MK531817, MK531821, MK554792, MT232635	Colombia, Costa Rica, Cuba, French Guiana, Mexico, USA (FL)
**_Ganoderma_ sp. A2 (2) [SH2762559.08FU]**		
as *G. resinaceum* (1), *Ganoderma* sp. (1)		
from decaying wood		
G. resinaceum MH106883		China (HI)
Ganoderma sp. MK554791		China
**_G. resinaceum_ (126)**		
as *G. resinaceum* (102), *Ganoderma* sp. (8), *G. lucidum* (6), *G. pfeifferi* (6), *G.* cf. *resinaceum* (2), Polyporales sp. (2)		
from *Acacia* sp., *Acer platanoides*, *A. sempervirens*, *Aesculus hippocastanum*, *Albizia molli*, *Alnus glutinosa*, *Cassia fistula*, *Celtis australis*, *Citrus aurantium*, *Dalbergia sissoo*, *Delonix regia*, *Eriobotrya japonica*, *Fagus sylvatica*, *Ficus benghalensis*, *Grevillea parallela*, *Jacaranda* sp., *Laurus nobilis*, *Limonia acidissima*, *Manilkara zapota*, *Melia azadirachta*, *Morus* sp., *Olea europae*, *Platanus* × *acerifolia*, *Platanus orientalis*, *Podocarpus* sp., *Prunus armeniaca*, *Quercus cerris*, *Q. conferta*, *Q. ithaburensis* spp. *macrolepis*, *Q. robur*, *Q. rubra*, *Q. suber*, *Quercus* sp., *Salix babylonica*, *Schinus molle*, *Tamarindus indica*, *Tecomella undulata*, *Ulmus* sp., *Vachellia tortilis*, roots		
G. resinaceum **MG706246** (19)	**MG706237, MG706247, MG706248, MG706249, MG706250,** AM906061, JN176880, JN176881, JN176882, JN176885, JN176896, KJ509597, KJ509598, KP941447, KT906370, KT906371, KT921215, KX371596	China, Greece, Italy
G. resinaceum **MG706233** (12)	**MG706234, MG706239, MG706240, MG706245, MG706254,** AM906060, AM906063, FJ491955, FJ491956, MT581895, MT581896	Greece, India, Italy
G. resinaceum **MG706242** (9)	**MG706251**, **MG706256**, EF060007, JQ520198[15], JQ520200[16], JQ520203, JQ627588, KY196415	Belgium, Greece, Netherlands, Poland, South Korea, UK
G. resinaceum AM906064 (6)	FJ491953, FJ491954, FJ491957, FJ665693, FJ665694	India, Italy
G. resinaceum AM269778 (4)	AM269774, JN588588 KJ143916[17]	France, Italy, Netherlands
G. resinaceum FJ491948 (4)	FJ491950, FJ491951, FJ491952	India
G. resinaceum KT343303 (4)	KT343307, KT343308, KT343309	Iran
G. pfeifferi **MG706244** (3)	**MG706255**, JN588589	Greece, Iran
G. resinaceum AY884177 (2)	JN222406	UK
G. resinaceum GU451246 (2)	GU451247	India
G. resinaceum MK415274 (3)*	MK415304, MK415305	Slovakia
G. resinaceum JX082326 (2)*	GU731559	France
G. lucidum KX371594 (2)*	KX371595	

Ganoderma sp. MH290277 (2)*	MH290285	India
G. resinaceum MK415309 (2)*	MK415311	Slovakia
Singletons (50)*	**MG706235, MG706236, MG706238, MG706241, MG706243, MG706252, MG706253**, AM269775, AM269776, AM269777, AM906062, AM906065, FJ491949, FJ805250, JQ312182, JQ312204, JN008875, JN588590, JQ520204, JX082328, KF605627, KF605628, KF975890, KJ143915, KT223756, KT223758, KT343310, KT343316, KT343317, LN774970, MH290273, MH571691, MH571692, MH796122, MH854909[16], MK415241, MK422153, MK531816, MK554766, MK554772, MK554775, MK554786, MN759653, MN240470, MN240471, MN398315, MN448375, X78737/ X78758[17], X78738/X78759[15], Z37062/Z37085[16]	Belgium, Bulgaria, China, Czech Republic, Egypt, France, Greece, India, Iran, Iraq, Italy, Netherlands, Slovakia, South Africa, Tunisia, UK
***Ganoderma* sp. A3 (12) [SH1723084.08FU]**<br>as *G. resinaceum* (5), *G.* cf. *resinaceum* (3), *Ganoderma* sp. (2), *G. lucidum* (1), uncultured *Ganoderma* (1)<br>from Dipterocarpaceae spp., roots		
G. cf. *resinaceum* MH160062		commercial
G. cf. *resinaceum* MH160068		commercial
G. cf. *resinaceum* MH160069		commercial
G. lucidum JQ520187		Taiwan
Singletons (8)*	GQ268631, JX270804, KT693256[13], KT693257[13], KT693258[14], KT693259[14], KT943367, KT943368	Malaysia, commercial
***G.* aff. *sessile* (4)**<br>as *G. lucidum* (4)<br>from *Cassia fistula*, *Tamarindus indica*		
G. lucidum FJ463905 (3)	FJ463913, FJ463923	India
G. lucidum AY870653		Turkey
***G.* aff. *polychromum* (8)**<br>as *G. resinaceum* (3), *G. sessile* (2), *G.* cf. *sessile* (1), *G. platense* (1), *G. zonatum* (1)<br>from *Platanus acerifolia*, *Quercus* sp.		
G. resinaceum KC311372 (2)	KC311374	
G. platense AH008109		Argentina
G. sessile KF605631		
G. zonatum AH008110		Argentina
G. cf. *sessile* MT232415		USA (AR)
Singletons (2)*	AH008111, KC311373	Argentina
***G. polychromum* (19)**<br>as *G. polychromum* (11), *G. lucidum* (6), *G. sessile* (2)<br>from *Acrocarpus fraxinifolius*, *Citrus aurantium*, *Terminalia bellerica*, soil		
G. polychromum MG654198 (9)	DQ424998, KF605632, KF605633, MG654199, MG654201, MG654202, MG654204, MG910492	USA (CA, OR)
G. lucidum AM269772 (2)	FJ463926	India, USA (CA)
G. lucidum FJ463915 (2)	FJ655477	India
G. polychromum MG654197 (2)	MG654203	USA (OR)
Singletons (4)*	MG654196, MG654205, MG910493, MN533787	China, USA (CA, OR, WA)
***G. sessile* (227)**<br>as *G. sessile* (134), *G. resinaceum* (60), *Ganoderma* sp. (15), *G. lucidum* (9), *G. oregonense* (2), *G. boninense* (1), *G. lobatum* (1), *G. neojaponicum* (1), *G. polychromum* (1), *G. valesiacum* (1), *Hericium erinaceum* (1), uncultured root-associated fungus (1)<br>from *Abies concolor*, *Albizia* sp., *Cocos nucifera*, *Dodonaea viscosa*, *Eucalyptus* sp., *Lantana camara*, *Tamarindus indica*, decaying wood		
G. sessile MG654214 (68)	AY636059, DQ424999, EU520249, FJ362324, FJ463924, FJ501561, JN021025, JQ520194, JQ520199, JQ520201[18], JQ520202, JQ520209, JQ520218[1], KC514812, KC514839, KF605638, KX055526, KX055530, KX055540, KX055542, KX055549, MG654219, MG654220, MG654229, MG654244, MG654245, MG654246, MG654247, MG654250, MG654251, MG654252, MG654253, MG654254, MG654255, MG654257, MG654261, MG654262, MG654265, MG654267, MG654268, MG654269, MG654270, MG654276, MG654280, MG654281, MG654282, MG654283, MG654292, MG654295, MG654296, MG654297, MG654298, MG654299, MG654300, MG654301, MG654302, MG654304, , MG654317, MG654318, MG654320, MG773847, MH294318, MH294326, MH855781[18], MN430930, MN622781, MN911336	Argentina, China, India, South Korea, USA (FL, KY, LA, MD, MI, MN, MO, NJ, NH, NM, NY, OH, SC, WV),

G. resinaceum KX371944 (17)	KX371946, KX371948, KX371949, KX371950, KX371951, KX371955, KX371956, KX371957, KX371960, KX371964, KX371965, KX371970, KX371974, KX371979, MH018025	
G. resinaceum KX371941 (16)	KX371945, KX371952, KX371953, KX371958, KX371961, KX371962, KX371966, KX371967, KX371968, KX371973, KX371975, KX371976, KX371978, KX371980, KX371982	
G. sessile MG654314 (14)	JQ520166, MG654200, MG654215, MG654218, MG654259, MG654279, MG654286, MG654294, MG654310, MG654312, MG654313, MG654315, MG65431	USA (CA, FL, MA, MN, MO, NJ, NM, NY, SC, UT)
G. sessile MG654226 (8)	MG654228, MG654230, MG654232[19], MG654235, MG654236, MG654240, MG654241	USA (FL)
G. sessile MG654258 (7)	MG654260, MG654264, MG654273, MG654274, MG654287, MG654288	USA (MA, MI, NY)
G. lucidum KJ143917 (3)	MG654237, MG654293	USA (CT, FL, NY)
G. sessile MG654222 (3)	MF755277, MG654278	USA (MN, TN)
G. sessile MG654277 (2)	JQ520208	USA (MN)
G. sessile MG654308[20] (2)	KY708882[20]	USA (FL)
G. resinaceum KX371938 (2)*	KX371939	
G. resinaceum KX371940 (2)*	KX371969	
G. resinaceum KX371942 (2)*	KX371981	
G. sessile KF605629 (2)*	KF605630	
G. sessile MH160057 (2)*	MH160060	commercial
G. sessile MG654221 (2)*	MG654285	USA (IL, MO)
G. sessile MG654225 (2)*	MG654227[21]	USA (FL)
G. sessile MG654289 (2)*	MG654290	USA (NY)
G. sessile MG654305 (2)*	KY708883[19]	USA (GA)
G. sessile MG654306 (2)*	MG654263	USA (MI, TX)
G. sessile MG654307[22] (2)*	KY646213[22]	USA (FL)
G. sessile MG654309[23] (2)*	KY646214[23]	USA (TX)
Singletons (63)*	AY508882, FJ463906, JQ520193, JQ520197, KF605634, KF605635, KF605636, KF605637, KF605639, KF605640, KF648564, KJ143918, KJ857267, KT223755, KX371947, KX371954, KX371959, KX371963, KX371971, KX371972, KX371977, KX957799, KY708884[21], MG654212, MG654213, MG654216, MG654217, MG654223, MG654224, MG654231, MG654233, MG654234, MG654238, MG654239, MG654242, MG654243, MG654248, MG654249, MG654256, MG654266, MG654271, MG654272, MG654275, MG654284, MG654291, MG654303, MG654311, MG654319, MG910998, MH027637, MH160056, MH160059, MH172163, MH290270, MH290283, MH290286, MH921462, MK554778, MK991834, MT196418, X78749/X78770, Z37051/Z37075, Z37053/Z37077	China, India, Japan, Russia, South Korea, Taiwan, USA (CA, DC, FL, IN, LA, MI, MO, NC, NJ, NY, SC, TX), commercial

**CLADE A, Cluster A.3**		
G. concinnum **(2)**<br>as *G. chalceum* (1), *G. concinnum* (1)		
G. chalceum JX310812		Brazil
G. concinnum MN077522		

G. tuberculosum **(36)**<br>as *G. tuberculosum* (27), *Ganoderma* sp. (6), *Coriolopsis caperata* (1), *G. parvulum* (1), *G. resinaceum* (1)<br>from *Cassia fistula*, *Pithecellobium dulce*, decaying wood		
G. tuberculosum KF963255 (10)	EU030178, KF963258, MG654351, MG654353, MG654355, MG654356, MG654358, MG654360, MG654362	Martinique, USA (FL), Panama
G. tuberculosum MG654363 (4)	MG654364, MG654365, MG654366	USA (FL)
Ganoderma sp. LT726719 (3)	LT726728, LT726729	Cuba
G. tuberculosum MG654367 (2)	KY646216[43]	USA (FL)
G. tuberculosum MG654368[44] (2)	KY708885[43]	USA (FL)
G. tuberculosum MG654369[45] (2)	KY709317[45]	USA (FL)
G. parvulum KU569528		Colombia
Ganoderma sp. KU569517		Colombia
Singletons (11)*	JX310824, KY646215, LT726725, LT726726, MG654352, MG654354[44], MG654357, MG654359, MG654361, MT232634, MT232639	Brazil, Cuba, Mexico, USA (FL, TX)

Ganoderma **sp. A4 (2) [UNITE's DOI not available]**<br>as *G. lucidum* (2)<br>from roots		

G. lucidum AH008112		Argentina
G. lucidum AH008113		Argentina

***G. wiiroense* (13)**<br>as *G. wiiroense* (9), *G. lucidum* (2), *Ganoderma* sp. (2)<br>from angiosperm, dead wood, decaying wood		
G. wiiroense KT952363/NR_158480 (3)	KT952361, KY111253	Ghana, India
G. wiiroense KY009867		India
G. wiiroense KY009869		India
G. lucidum MH553170		India
Singletons (7)*	FJ982798, KJ510534, KY009864, KY009873, KY963355, MF774620, MH889141	India, Senegal

***G. flexipes* (7)**<br>as *G. flexipes* (7)<br>from *Pinus* sp., ground of angiosperms, decaying wood, soil		
G. flexipes MK345430 (3)	MK345431, MN398340	China, Laos, Vietnam
G. flexipes JQ781850		China (HI)
G. flexipes JN383978		China
G. flexipes JN383979		China
G. flexipes MH106873		China (HI)

***Ganoderma* sp. A5 (7) [SH1723120.08FU]**<br>as *G. multiplicatum* (7)<br>from *Quercus* sp., decaying wood		
G. multiplicatum KU572488 (2)	MN401405	China
G. multiplicatum MK345439 (2)	MK345440	Myanmar
G. multiplicatum KU572489		China
G. multiplicatum MH106879		China (HI)
Singleton (1)*	KU572490	China

***G. philippii* (102)**<br>as *G. pseudoferreum* (75), *G. philippii* (15), *Ganoderma* sp. (9), *G. australe* (2), uncultured soil fungus (1)<br>from *Acacia mangium*, *Crudia ripicola*, *Hevea brasiliensis*, *Hevea* sp., dead wood, soil		
G. philippii MG279188 (19)	AJ536663, AJ542521, AJ543431, KX454345[24], KX454346[25], KX454347[26], KX454376[27], KX454377[28], KX454389[28], KX454391[29], KX454397[27], KX454398[28], KX454412[29], KX454413[30], KY379054, MG279189, MN401410, MN401411	China (HI), Indonesia, Thailand
G. pseudoferreum KX454334[27] (10)	FJ374875, KX454336[31] , KX454337[32], KX454338[33], KX454340[34], KX454342[35], KX454343[36], KX454354[37], KX454388[25]	
G. philippii MG279166 (7)	KX454344[35], KX454348[38], KX454351[37], KX454385[36], KX454390[38], KX454411[36]	
G. philippii MG279167 (6)	KX454379[32], KX454387[24], KX454406[36], KX454407[35], KX454408[24]	
G. pseudoferreum FJ392280 (5)	FJ392282, KX454349[29], KX454350[30], KX454400[32]	
G. pseudoferreum KX454382[34] (5)	KX454383[39], KX454384[35], KX454404[39], KX454405[35]	
G. pseudoferreum KX454394[40] (3)	KX454401[33], KX454414[37]	
Ganoderma sp. AJ536661 (3)	AJ577748[40], AJ608712[40]	Indonesia
G. pseudoferreum FJ392279 (2)	KX454395[41]	
G. pseudoferreum KX454381[42] (2)	KX454393[37]	
G. philippii MH152513 (3)*	MH152515, MH152516	China
G. pseudoferreum KX454335[28] (2)*	KX454353[41]	
G. pseudoferreum KX454339[42] (2)*	KX454352[40]	
Singletons (33)*	AJ536662, AJ608710, AJ608711, AJ608713, AJ608714/5, AJ627584, FJ374874, FJ374876, FJ378651, FJ392281, FJ392283, FJ392284, FJ392285, KX454341[39], KX454378[31], KX454380[33], KX454386[35], KX454392[30], KX454396[37], KX454399[31], KX454402[42], KX454403[34], KX454409[25], KX454410[26], KX454415[40], KX454416[41], KX454417[37], LC084725, LC084750, MF942546, MH152514, MK131239, MK131243	China, Indonesia, Malaysia

**Group A.3.1**		
***G. lingzhi* (436)**<br>as *G. lingzhi* (244), *G. lucidum* (131), *Ganoderma* sp. (32), *G. sichuanense* (20), *G. tsugae* (2), *Amauroderma rugosum* (1), *G. boninense* (1), *G. calidophilum* (1), *G. cupreum* (1), *G. luteomarginatum* (1), *Haddowia longipes* (1), *Laccaria bicolor* (1)<br>from *Cassia* sp., *Cassia spectabilis*, *Castanea* sp., *Castanopsis* sp., *Citrus aurantium*, *Delonix regia*, *Ficus benghalensis*, *Mangifera* sp., *Peltophorum* sp., *Pterocarpus* sp., *Quercus glaucoides*, *Quercus* sp., *Sesbania* sp., *Tamarindus indica*, living wood, dead wood, roots, soil		

G. lingzhi JQ781855 (105)	AB462322[46], BD082760, EF188278, EF188279, EU498091, FJ379262, FJ687271, FJ940919, GU213479, GU213481, GU213483, JF915403, JF915407, JN008870, JN197281, JN222405, JN222423, JQ520189, JQ520211, JQ627589, JQ627590, JQ781856, JQ781857[47], KJ143908, KM249919, KM249922, KM249925, KT693250[48], KT693251[48], KT693252[49], KT693253[49], KT693255[50], KU219990, KU863089, KX589250, KY244065, KY364244, KY364245, KY364246, KY364247, KY364248, KY364249, MF476197, MF476200, MF476201, MH042929, MH109548, MH109550, MH109552, MH109557, MH109558, MH109561, MH109563, MH109564, MH109565, MH109567, MH109570, MH109571[51], MH109573, MH109574, MH109575, MH109586[47], MH109591, MH109594, MH109596, MH109597, MH109599, MH109601, MH109602, MH109609, MH109610, MH109613, MH109615, MH109618, MH109621, MH109623, MH109626, MH109627, MH109629, MH109630, MH109632, MH109633, MH109635, MH109640, MH109642, MH109644, MH109646, MH109653, MH109657, MH109662, MH109670, MH109673, MH109674, MH160073, MH160076, MK268933, MN294898, MN396319[52], MN396321[52], MN396322, MN396323, MN911330, MT741782, UDB038354	China (BJ, JS, LN, SC, ZJ), Japan, South Korea, Thailand, commercial
G. lingzhi JQ781861[51] (47)	DQ424989, DQ424993, EU021456, JF915399, JF915408, JN008869, JN008871, JN008872, JN197284, JN222424, JN222425, JN222426, JQ520168, JQ520172, JQ520173, JQ781862[53], KJ143907[54], KM249910, KM249918, KM249931, KX055548, KX055562, KX055563, KX589245, KX589247, KX589248, MF476198, MG739456, MH109549[55], MH109551, MH109556, MH109560, MH109566, MH109569, MH109576, MH109581, MH109608, MH109639, MH109649, MH109661, MH109665, MH109675, MH294300, MK370666, MN431189, MN911333	China (HA, JS, SD, SC, TJ), Japan, South Korea
G. lingzhi JQ781867[56] (40)	HQ689696, JF915400, JQ520188, JQ781868, KU219991[55], KY244061, KY244062, KY244063, KY244066, KY244068, KY404119, LC068794, LC269925, MG279180, MH109553, MH109592, MH109595, MH109598, MH109606, MH109607[54], MH109614, MH109619, MH109620, MH109624, MH109625, MH109628, MH109631, MH109634, MH109637, MH109638, MH109641, MH109643, MH109647, MH109655, MH109658, MH109676, MH294312, MT510201, UDB033504	China (HI, YN, SC), Japan, Laos, Thailand
G. lingzhi JQ781869[58] (38)	FJ379265, FJ501568, GU213480, HQ235630, HQ235631, JF915394, JF915395, JQ520169, JQ520180, JQ520207, JQ781870, KM249914, KX055555, KX589244, KX589246, KX589249, KY364250, MH109555[59], MH109559, MH109578, MH109582, MH109583, MH109584, MH109585[58], MH109589[60], MH109603, MH109656, MH109663, MH109664, MH109671, MK603977, MK809459, MN622726, MN911331, MN911337, MN911339, MN911341	China (FJ, JX, SC, ZJ), South Korea, commercial
G. lingzhi MH160075 (20)	GU213471, KM249921, KX055546, MH109577, MH109579, MH109580, MH160078, MH160083, MH160084, MH160085, MN911326, MN911327, MN911328, MN911329, MN911340, MN911342, MN911344, MN911345, MN911347	China, commercial
<u>*G. lingzhi* JQ781858 (18)</u>	JF915393, JF915402, JN048774[61], JN197283, JQ520182, JQ520183, JQ520184, JQ781859[61], KM249917, KM249924, KX055532, KX055541, KX055547, MH109672, MK182314, MN258634, UDB034543	Bangladesh, China (HA, HB, JX, SD, SC), Japan, Laos
***G. tsugae* MG706221** (12)	GU213478, HQ235632, KM249923, KR093032, KU310901, KX055544, KX055550, KX055551, KX055553, MH109588, MH109636	China, Malaysia, commercial
***G. lucidum* MG706222** (4)	GU213484, KM249928, KM269294	China
G. calidophilum KY612892		China
<u>*G. sichuanense* KC662402/NR_152892</u>		China
G. lingzhi JF915397 (14)*	JQ520176, KC511557, KM249911, KM249919, KM249932, MH109604, MH109611, MH109617, MH109659, MN911332, MN911338, MN911343, MN911346	China (ZJ), South Korea
G. lingzhi JF915405 (11)*	KC415761, KP226861, KP226862, KX055521, KX055522, KX055524, KX055525, KX055531, KX055534, KX055536	China (HI, JX), commercial
G. lingzhi MH294301 (11)*	DQ424970, DQ424983, DQ424987, DQ424997, DQ425001, DQ425007, DQ425013, KC311370, MH294307, MH294314	China

G. lingzhi KM249913 (10)*	FJ501554, FJ501557, FJ501569, FJ501577, KX055533, KX055535, KX055539, KX055543, KX055545	
G. lingzhi JQ781871[59] (8)*	DQ424984, DQ425015, FJ501559, JQ781872, JQ781873[60], MH294327, MN911335	China (FJ, JS, SD), commercial
G. lingzhi JF915396 (6)*	JF915406, JQ781865, KM249927, KX055523, MH109593	China (HB, SD, SH), commercial
G. lingzhi JQ781864 (6)*	MN396324, MN396325, MN396326, MN396327, MN396328	China (AH), Thailand, commercial
G. lingzhi KM249915 (6)*	GU213487, KM249929, KX055527, KX055528, MN911334	China
G. lingzhi MH294306 (6)*	DQ424979, DQ424985, DQ425008, KC311368, MH294328	China
G. lucidum FJ463907 (5)*	FJ463910, FJ463912, FJ463919, FJ463929	India
G. lingzhi MH294304 (5)*	DQ424980, DQ424991, DQ425004, MH294323	China
G. lingzhi MH294308 (5)*	DQ424973, DQ424988, MH294322, MH294333	China
G. lucidum FJ463904 (4)*	FJ463909, FJ463921, FJ463931	India
G. lingzhi JF915404 (4)*	MH109590, MH109622, MH109645	China (SC)
G. lucidum JQ520171 (4)*	GU213485, JQ520181, KF146177	South Korea
G. lingzhi MH160058 (4)*	MH160061, MH160064, MH160066	commercial
G. lingzhi MH294315 (4)*	DQ424992, DQ424994, MH294324	China
G. lingzhi AB811848 (3)*	MN396329, MN396330	Myanmar, Nepal
G. lucidum DQ424971 (3)*	DQ424986, KC311371	
G. lingzhi JQ781860 (3)*	DQ424974, EU021455[46]	China (AH), Japan
G. lingzhi JN197282 (3)*	KM249912, KT906368	China
G. sichuanense KT318600 (3)*	KT318601, KT318602	
G. lucidum DQ424981 (2)*	MH294320	China
G. lucidum FJ501555 (2)*	FJ501576	
G. lucidum GU213476 (2)*	GU213477	
G. lingzhi JQ781863 (2)*	MH109568	China (HA)
G. lucidum MF476199 (2)*	MH294325	China
G. lingzhi MG732948 (2)*	MG732949	
G. lucidum MH018022 (2)*	MH018030	
G. lucidum MH018027 (2)*	MH018028	
G. lingzhi MH294317 (2)*	EU520247	China
G. lingzhi MH294329 (2)*	MH294305	China
G. lingzhi MK345437 (2)*	MK345438	Laos

***G. curtisii* (141)**

as *G. curtisii* (123), *G. meredithae* (11), *G. lucidum* (3), *G. curtisii* f. sp. *meredithae* (2), *Ganoderma* sp. (2) from *Quercus* sp., roots

G. meredithae MH862131 (39)	KF605641, KY708881[62], MF773591, MG654075, MG654078, MG654085, MG654087, MG654088, MG654090, MG654092, MG654094, MG654095, MG654096, MG654097[63], MG654111, MG654112, MG654122, MG654123, MG654124, MG654125, MG654127, MG654131, MG654132, MG654137, MG654140, MG654141, MG654143, MG654149, MG654156, MG654158, MG654159, MG654160, MG654165, MG654166, MG654167, MG654171, MG654187, MG654188[62]	USA (DC, FL, GA, IL, LA, MI, NC, NH, NY, PA, SC, TN, TX)
G. curtisii MG654083 (6)	KY708878[64], MG654093, MG654109, MG654147[64], MK370946	USA (FL, MD, NC)
G. curtisii KF963259 (5)	JX270803, KF605644, LN998989, MH289783	Mexico
G. curtisii MG654144 (5)	KF605642, KT223757, MG654151, MG654172	USA (FL, NC, TX)
G. curtisii MG654084 (3)	MG654133, MG654170	USA (FL, NJ)
G. curtisii MG654107 (3)	MG654108, MG654114	USA (FL)
G. curtisii MG654120 (3)	MG654126, MG654129	USA (GA, MA, NC)
G. curtisii MG654153 (3)	MG654161, MG654168	USA (FL, NC)
G. meredithae KY646219[65] (2)	KY646220[66]	
G. meredithae MG654103[65] (2)	MG654106[66]	USA (FL)
G. curtisii MG654146 (2)*	KY708879[63]	USA (FL)
G. curtisii MG654077 (2)*	MG654154	USA (GA, FL)
G. curtisii MG654079 (2)*	MG654086	USA (FL)
G. curtisii MG654080 (2)*	MG654142	USA (FL, SC)
G. curtisii MG654076 (2)*	MG654162	USA (NC, TX)
G. curtisii MG654100 (2)*	MG654118	USA (FL, GA)
G. curtisii MG654102 (2)*	MG654104	USA (FL)
G. curtisii MG654121 (2)*	MG654135	USA (GA, NJ)
G. curtisii MG654074[67] (2)*	KY646217[67]	USA (NC)
G. curtisii MG654128 (2)*	JQ520191	USA (MO)
G. curtisii MT254976 (2)*	MT254979	
Singletons (48)*	JQ520164[68], JQ520190, JQ781848, JQ781849[68], JX270802, KF605643, KF605646, KY646218, KY708880, KY767033[69], MG654081, MG654082, MG654089, MG654091, MG654098,	Mexico, USA (FL, GA, NC, NJ, NY, OK, SC, VA), commercial

	MG654099, MG654101, MG654105[69], MG654110, MG654113, MG654115, MG654116, MG654117, MG654119, MG654130, MG654134, MG654136, MG654138, MG654139, MG654145, MG654148, MG654150, MG654155, MG654157, MG654163, MG654164, MG654169, MG966318, MH160063, MH160072, MH165297, MH211682, MH347284, MH910547, MK532860, MT254977, MT254978, MT254980	
***G. ravenelii* (12)**<br>as *G. ravenelii* (6), *G. curtisii* (3), *G. lucidum* (2), uncultured fungus (1)<br>from *Pinus taeda*, *Tamarindus indica*		
G. ravenelii MG654206 (6)	KF605645, KM103994, MG654209, MG654210, MG654211	USA (FL)
G. lucidum AY456341 (2)	FJ463928	India, USA (NC)
G. ravenelii MG654207		USA (FL)
G. ravenelii MG654208		USA (FL)
Singletons (2)*	MG654152, MG654173	USA (FL)
***G. multiplicatum* (15)**<br>as *G. multiplicatum* (9), *G. perzonatum* (6)<br>from *Cenostigma pluviosum* var. *peltophoroides*, *Tipuana tipu*, living wood		
G. multiplicatum KU569553 (2)	KJ792745	Brazil
G. multiplicatum JX310823		Brazil
G. multiplicatum KX180914		Colombia
G. perzonatum KJ792747		
G. perzonatum KJ792752		
Singletons (9)*	KJ792749, KJ792750, KU569515, KU569542, KU569549, MG279185, MK119830, MT232637, MT232641	Brazil, Colombia, Mexico
***G. destructans* – *G. dunense* (43)**<br>as *G. destructans* (39), *G. dunense* (3), uncultured soil fungus (1)<br>from *Celtis africana*, *Dracaena braunii*, *Jacaranda mimosifolia*, *Searsia chirindensis*, *Senegalia nigrescens*, *Spathodea campanulata*, *Vachellia xanthophloea*, soil		
G. destructans KR183856/NR_132919 (21)	KR183857, KR183858, MG020234, MG020236, MG020244, MG020245, MG020246, MG020248, MG020251, MG020256, MG020257, MG020259, MG020262, MG020266, MG020267, MG020268, MG020269, MG279177, MH571694, MH571695	South Africa
G. destructans MG020237 (8)	MG020238, MG020240, MG020241, MG020242, MG020243, MG020247, MG020253	South Africa
G. destructans MG020232 (4)	MG020239, MG020250, MG020261	South Africa
G. dunense MG020255 (3)	MG020252, MG020254	South Africa
G. destructans MG020249 (2)	MG020263	South Africa
Singletons (5)*	MG020233, MG020235, MG020258, MG020260, UDB0765706	Cameroon, South Africa
***G. mizoramense* (3)**<br>as *G. mizoramense* (2), *G. lucidum* (1)<br>from angiosperms		
G. mizoramense KY643750 (3)	KY643751, MG437336	India
***G. steyaertanum* (39)**<br>as *G. steyaertanum* (34), *G.* aff. *steyaertanum* (3), *Ganoderma* sp. (2)<br>from *Acacia mangium*, *Albizia lebbeck*, dead wood		
G. steyaertanum KJ654432 (2)	KJ654451	
G. steyaertanum KJ654450 (2)	KJ654459	
G. steyaertanum KJ654452 (2)	KJ654461	
G. steyaertanum KJ654456 (2)	KJ654457	
G. steyaertanum KJ654462 (2)	MK131244	Indonesia
G. steyaertanum EU239395/6		Australia
Singletons (28)*	EU239384, EU239385, EU239388, EU239391/2, KJ654419, KJ654420, KJ654421, KJ654422, KJ654423, KJ654424, KJ654425, KJ654426, KJ654427, KJ654428, KJ654429, KJ654430, KJ654431, KJ654433, KJ654447, KJ654448, KJ654449, KJ654453, KJ654454, KJ654455, KJ654458, KJ654460, KP012964, MF436674	Australia, Indonesia
***G. martinicense* (48)**<br>as *G. parvulum* (24), *G. martinicense* (17), *G. perzonatum* (2), *G. lucidum* (1), *G. oerstedii* (1), *G. tornatum* (1), *G. tuberculosum* (1), *Ganoderma* sp. (1)<br>from *Bauhinia purpurea*, *Cassia fistula*, *Cassia siamea*, *Inga laurina*, *Leucaena leucocephala*, *Pithecellobium dulce*, *Scutia buxifolia*, living wood, dead wood, decaying wood		

G. parvulum KU569511 (16)	KU569512, KU569513, KU569514, KU569516, KU569518, KU569519, KU569520, KU569522, KU569523, KU569524, KU569525, KU569526, KU569527, KU569530, KU569555	Colombia
G. martinicense MG279163 (9)	MG654174, MG654175, MG654177, MG654181, MG654182, MG654183, MG654184, MG654185	USA (GA, NC, SC, TN, TX)
G. parvulum JX310819 (4)	JQ618246, JX310820, JX310822	Brazil
G. parvulum JX310817 (2)	JX310818	Brazil
G. parvulum JX310821 (2)	KJ792744	Brazil
G. martinicense KF963257 (2)	LT726720	Cuba, Martinique
G. martinicense MG654179 (2)	MG654180	USA (TN)
G. martinicense KF963256		Martinique
G. parvulum KU569521		Colombia
Singletons (9)*	AH008114, KJ792746, KT247418, KU569510, KY350855, MG654176, MG654178, MG654186, Z37059/Z37084	Argentina, Colombia, Martinique, USA (AL, NC, TN)

***G. multipileum* (130)**
as *Ganoderma* sp. (59), *G. lucidum* (53), *G. multipileum* (17), *G. leucocontextum* (1)
from *Acacia auriculiformis, A. nilotica, Albizia lebbeck, Albizia* sp., *Areca catechu, Azadirachta indica, Cassia fistula, Cassia* sp., *Casuarina equisetifolia, Cocos nucifera, Dalbergia melanoxylon, Dalbergia sissoo, Ficus benghalensis, Leucaena leucocephala, Mangifera indica, Pinus merkusii, Prosopis cineraria, Senegalia senegal, Thespesia populnea, Vachellia nilotica,* dead wood, decaying wood, roots, soil

G. lucidum HM053462 (24)	KM229564, KM229566, KM229568, KM229570, KM229572, KM229574, KM229582, KM229584, KM229589, KM229591, KM229595, KM229596, KR155072, KT188588, KT188600, KT188603, MF289194, MH290252, MH290255, MH290256, MH290258, MT126496, MT871995	India
G. multipileum MG279184 (20)	GQ249884, GQ249886, GU726928, GU726929, GU726930, GU726931, GU726932, GU726933, HM053463, KM229565, KM229576, KR155075, KR155076, LC149613, MH290251, MH290254, MH290259, MH290260, MK940282	China (SC), India, Nepal
G. multipileum KJ143914[71] (18)	EU021460, EU021461, GQ249880, GU726919, GU726921, HM053443, JQ781874, KF494997[71], , KM229579, KM229580, KM229583, KM229585, KM229594, MG739453, MG739454, MG739455, MN533795	China (HI), India, Taiwan
G. multipileum EU021462 (4)	JN049906, KF998095, KM229588	India, Taiwan
G. multipileum JF915409 (4)	KM229567, KR154956, MH290282	China (SC), India
Ganoderma sp. KR155023 (3)	KR155024, KT188593	India
G. lucidum GU726923 (2)	GU726925	
G. lucidum GU726926 (2)	GU726927	
G. lucidum HM053466 (2)	HM053467	
Ganoderma sp. KM229577 (2)	KM229592	India
G. lucidum HM053457 (5)*	HM053458, HM053459, HM053460, HM053461	
G. lucidum HM053436 (4)*	HM053437, HM053438, HM053439	
G. lucidum HM053440 (3)*	HM053441, HM053442	
G. lucidum HM053446 (3)*	HM053447, HM053448	
G. multipileum KJ143913 (3)*	MN401406[72], MN401407	Taiwan, Thailand
G. multipileum KX055554 (3)*	KX055556, MG450346	
Ganoderma sp. MH290247 (3)*	MH290249, MH290257	India
Ganoderma sp. MH290267 (3)*	MH290275, MH290281	India
G. lucidum HM053455 (2)*	HM053456	
G. lucidum HM053451 (2)*	HM053452	
G. lucidum FJ463908 (2)*	FJ463920	India
G. lucidum HM053453 (2)*	HM053454	
G. lucidum HM053464 (2)*	HM053465	
Ganoderma sp. JN810910 (2)*	KM655758	India
Ganoderma sp. MH290263 (2)*	MH290278	India
Ganoderma sp. MH290276 (2)*	MH290284	India
Ganoderma sp. MH290274 (2)*	MH290280	India
Ganoderma sp. MH290269 (2)*	MH290272	India
G. lucidum MK937782 (2)*	MN729467	India

Group A.3.2

***Ganoderma* sp. A6 (15) [SH1723103.08FU]**
as *G. tropicum* (15)
from *Cassia fistula*, *Cassia* sp., *Delonix regia*, *Ficus benghalensis*, *Podocarpus* sp., *Tamarindus indica*, *Terminalia bellerica*

G. tropicum FJ491960 (6)	FJ491963, FJ491964, FJ491982, FJ491984, FJ491985	India
G. tropicum FJ491962 (3)	FJ491965, FJ491983	India

G. tropicum FJ491959 (2)	FJ491966	India
G. tropicum FJ491958 (2)	FJ491980	India
G. tropicum FJ491961 (2)	FJ491981	India
**G. tropicum (31)**		
as *G. tropicum* (15), *G. fornicatum* (12), *G. williamsianum* (2), *Vanderbylia fraxinea* (2), *Ganoderma* sp. (1), uncultured soil fungus (1) from *Acrocarpus fraxinifolius*, *Artocarpus heterophyllus*, *Dipterocarpus* sp., *Ficus benghalensis*, *Terminalia bellerica*, *Terminalia* sp., *Tetrameles nudiflora*, decaying wood, soil		
G. tropicum MH823539 (6)	JQ781880, KX081091, MK007291, MN523320, UDB033739	China (YN), Laos, Taiwan, Thailand
G. fornicatum AY593863[70] (4)	FJ655472, FJ655473, FJ655475	India, Taiwan
G. tropicum MK345456 (3)	MK345457, MK345458	China (HI), Thailand
G. tropicum EU021458 (2)	KP027536	Taiwan
G. tropicum JF915410 (2)	MG279194	China (HI)
G. fornicatum AY593859[70]		Taiwan
G. fornicatum AY593860[70]		Taiwan
G. fornicatum Z37067/Z37087		Taiwan
G. tropicum Z37068/Z37088		Taiwan
Singletons (10)*	AY593861[70], AY593862[70], EU021457, FJ655471, FJ655474, FJ655476, JQ781879, KC222317, KF495000, MH106884	China (GX, HI), India, Taiwan
***Ganoderma* sp. A7 (3) [SH1723183.08FU]**		
as *G. fornicatum* (3)		
G. fornicatum JN596323		Malaysia
G. fornicatum JN596324		Malaysia
G. fornicatum JN596325		Malaysia
**CLADE B**		
***Ganoderma* sp. B1 (4) [SH1723111.08FU]**		
as *Ganoderma* sp. (4)		
Ganoderma sp. KF605666		USA
Ganoderma sp. KF605667		USA
Ganoderma sp. KF605668		USA
Ganoderma sp. MG279151		China
***Ganoderma* sp. B2 (3) [SH1723166.08FU]**		
as *G. applanatum* (1), *G. lingzhi* (1), *G. multipileum* (1)		
G. multipileum AB811849 (2)	AB811852	Nepal
G. applanatum AB811850		Nepal
***G. applanatum* (158)**		
as *G. applanatum* (90), uncultured soil fungus (29), *G. lipsiense* (18), uncultured fungus (7), uncultured *Ganoderma* (6), *G. adspersum* (4), fungal sp. (1), *G. applanatum* cplx (1), *G.* cf. *applanatum* (1), *G. oregonense* (1) from *Abies holophylla*, *Artocarpus* sp., *Betula pendula*, *Betula* sp., *Dipterocarpus* sp., *Fagus sylvatica*, *Ficus benghalensis*, *Fraxinus excelsior*, *Grevillea parallela*, *Machilus* sp., *Populus nigra*, *P. tremuloides*, *Prunus armeniaca*, *Quercus robur*, *Quercus* sp., *Salix* sp., *Sorbus aucuparia*, *Tilia cordata*, *T. platyphyllos*, dead wood, decaying wood, roots, soil		
G. applanatum **MG706213** (96)	**MG706214, MG706215,** AF255093[73], AF255094, AF255096, AY884178, AY884179, EF059994, EF059995, EF059996, EF059997, EF059998, EF059999, EF060001, EF060002, EF060003, EF060005, EF060006, EU554851, EU554859, FJ609287, FR686556, FR750674, GU256764, GU731555, JN176900, JN588587, JQ520162, JQ520196, JX501311, KC581319, KF605647, KF975891, KF975892, KJ140577, KP941443, KT334783, KT334785, KT334788, KT334790, KU863083, KX055561, KX449492, KY364256, KY364257, KY364258, LC096119, MG279158, MG835863, MH160077, MH191249, MH320562, MH321900, MH855178[73], MK345426, MK351671, MK351677, MK351727, MK351745, MN173820, MN396332, MN396333, MN396334, MN396335, MN396336, MN396337, MN435132, MN435134, MN435135, MN435136, MN435137, MN435138, MN435139, MN435140, MN435141, MN435142, MN435143, MN435144, MN435145, MT236432, UDB011619, UDB0162233, UDB0219498, UDB0246103, UDB0300725, UDB0352927, UDB040056, UDB041383, UDB0556942, UDB0580293, UDB0668753, UDB0680950, UDB0756506, UDB0758107, UDB099583	Armenia, Bulgaria, Canada, China (Tibet), Estonia, France, Germany, Greece, India, Kyrgyzstan, Lithuania, Netherlands, Poland, Russia, South Korea, Thailand, UK, USA (AR, NC, WI), commercial
G. applanatum MK415242 (25)	MK415245, MK415246, MK415247, MK415248, MK415249, MK415251, MK415252, MK415254, MK415255, MK415260,	Estonia, Slovakia

	MK415264, MK415273, MK415275, MK415277, MK415279, MK415281, MK415282, MK415283, MK415284, MK415298, MK415299, MK415308, MK415313, UDB0625825	
G. applanatum MK415243 (3)	MK415253, MK415297	Slovakia
G. adspersum FJ655448 (2)	FJ655450	India
G. applanatum FJ655454 (2)	FJ655455	India
G. applanatum MF143510 (2)	MF143512	
G. applanatum MF143514 (2)	MF143515	
G. applanatum KJ668542 (2)	MH425253	South Korea
G. applanatum MH027634 (2)	UDB0101232	Estonia, Russia
G. applanatum MK351672 (2)	UDB0510029	Estonia, Kyrgyzstan
G. applanatum MG279157 (2)*	UDB0443642	Estonia
G. applanatum KJ857259 (2)*	MK415278	Slovakia
G. applanatum MF143511 (2)*	UDB0380819	Estonia
uncultured soil fungus JQ666457 (2)*	JQ666465	China
uncultured *Ganoderma* FJ626936 (2)*	KC785577	Antarctica, Canada
uncultured *Ganoderma* EU554863 (2)*	HQ021835	USA (NH)
uncultured soil fungus UDB0309683 (2)*	UDB061192	Estonia
uncultured soil fungus UDB050390 (2)*	UDB085994	Estonia
uncultured soil fungus UDB0324687 (2)*	UDB0335181	Estonia
uncultured soil fungus UDB063943 (2)*	UDB0661494	Estonia

**CLADE C, Cluster C.1**		
***G. neojaponicum* (10)**<br>as *G. neojaponicum* (7), *G. calidophilum* (2), *Ganoderma* sp. (1)<br>from *Castanopsis* sp., *Machilus* sp., soil		
G. neojaponicum KT318596 (3)*	MN398337, MN398338	China (HI)
G. neojaponicum AY593866[74]		Taiwan
G. neojaponicum AY593867[74]		Taiwan
G. neojaponicum MK345443		Laos
G. neojaponicum MK345444		Myanmar
Singletons (3)*	AY335163, KT318598, MN871859	China

**CLADE C, Cluster C.2**		
***Ganoderma* sp. C1 (2) [SH1723198.08FU]**<br>as *Ganoderma* sp. (2)<br>from *Elaeis guineensis*		
Ganoderma sp. JN105713		Cameroon
Ganoderma sp. JN105714		Cameroon
***G. aridicola* (7)**<br>as *Ganoderma* sp. (6), *G. aridicola* (1)<br>from *Cassia* sp., *Elaeis guineensis*, *Pinus sylvestris*		
Ganoderma sp. JN105708 (2)	JN105710	Cameroon
<u>*G. aridicola* KU572491/NR_152914</u>		South Africa
Ganoderma sp. JN105707		Cameroon
Ganoderma sp. JN105711		Cameroon
Singletons (2)*	JN105709, JN105712	Cameroon
***Ganoderma* sp. C2 (3) [SH1843092.08FU, SH1843096.08FU]**<br>as *Ganoderma* sp. (3)<br>from *Cassia* sp.		
Ganoderma sp. JN105715		Cameroon
Ganoderma sp. JN105716		Cameroon
Ganoderma sp. JN105717*		Cameroon
***G. enigmaticum* – *G. thailandicum* (10)**<br>as *G. enigmaticum* (7), *G. thailandicum* (2), uncultured soil fungus (1)<br>from *Ceratonia siliqua*, *Acacia cyclops*, *Combretum* sp., *Pinus merkusii*, soil		
G. enigmaticum KR014265 (2)	KR150678	Ghana
<u>*G. thailandicum* MK848681 (2)</u>	MK848682	Thailand
<u>*G. enigmaticum* KR183855/NR_132918</u>		South Africa
G. enigmaticum KU572486		South Africa
Singletons (4)*	KU572487, MH571697, MK453308, UDB0767618	Ghana, Ivory Coast, South Africa
***G. casuarinicola* (56)**<br>as *Ganoderma* sp. (42), *G. casuarinicola* (6), *G. enigmaticum* (4), uncultured fungus (2), *G. carnosum* (1), uncultured *Ganoderma* (1)		

from *Acacia auriculiformis*, *A. nilotica*, *Areca catechu*, *Casuarina equisetifolia*, *Cocos nucifera*, *Moringa oleifera*, *Peltophorum pterocarpum*, *Tamarindus indica*, dead wood, decaying wood		
Ganoderma sp. KM229615 (5)	KM229621, KM229622, KM229631, KM229632	India
G. casuarinicola MG279173/NR_158432 (5)	MG279174, MG279175, MG279176, MK817650	China (GD)
Ganoderma sp. KF998090 (4)	KF998096, KM229628, KM229630	India
Ganoderma sp. KY111251 (4)	KM229623, KM229624, KM229633	India
Ganoderma sp. KM229626 (2)	KM229627	India
Ganoderma sp. KT186191 (2)	KT188605	India
Singletons (34)*	AM773630, KC590320, KM229616, KM229617, KM229618, KM229619, KM229620, KM229625, KM229629, KP780431, KR154917, KR154951, KR154957, KR155019, KR155071, KU712536, KR733545, KR818818, KT186194, KT188601, KT188602, KT188604, KU870313, KU886297, KY009866, KY111250, KY978282, KY978295, MH023509, MK681870, MT126492, MT229201, MT364486, MT364487	China, India, Sri Lanka
**CLADE D, Cluster D.1**		
***G. mbrekobenum* (31)**		
as *Ganoderma* sp. (17), *G. mbrekobenum* (11), *G. applanatum* (1), *G. carnosum* (1), *G. tsugae* (1)		
from *Areca catechu*, *Azadirachta indica*, *Casuarina equisetifolia*, *Citrus × limonia* or *Citrus reticulata*, *Citrus* sp., *Ficus benghalensis*, *Ficus religiosa*, *Jacaranda mimosifolia*		
Ganoderma sp. KM229603 (3)	KP794597, KP794598	India, Sri Lanka
G. mbrekobenum KX000896/NR_147647 (2)	KX000898	Ghana
Ganoderma sp. KM229604		India
Ganoderma sp. KM229607		India
Ganoderma sp. KM229613		India
Singletons (23)*	KJ510532, KM229599, KM229600, KM229601, KM229602, KM229605, KM229606, KM229609, KM229610, KM229611, KM229612, KM229614, KP943501, KY865253, LN774971, MH221092, MH221093, MK453307, MK940286, MK940287, MK940289, MK940290, MN097540	India, Senegal, Sri Lanka
**CLADE D, Cluster D.2**		
***G. nasalanense* (15)**		
as *G. australe* (8), *Ganoderma* sp. (3), *G. nasalanense* (2), uncultured soil fungus (2)		
from decaying hardwood, soil		
G. nasalanense MK345441/NR_164048 (2)	MK345442	Laos
G. australe LC084707 (2)	LC084729	Malaysia
Ganoderma sp. KR093030		Malaysia
G. australe LC084678		Malaysia
Singletons (9)*	LC084677, LC084692, LC084722, LC084731, LC084737, KT965500, KT965501, UDB0760488, UDB0767030	India, Malaysia, Vietnam
***G. sinense* (66)**		
as *G. sinense* (45), *Ganoderma* sp. (8), *G. lucidum* (5), *G. japonicum* (4), *G. subresinosum* (2), *G. atrum* (1), *G. formosanum* (1)		
from *Albizia molli*, *Castanopsis* sp., *Dipterocarpus* sp., *Tectona* sp., decaying wood, soil		
G. sinense MH106882 (20)	AY593865[75], DQ425014, GU213482, HQ235633, HQ235634, JQ520212, MH294319, MK313117[76], MK313119[77], MK313121, MK313124, MK313125[76], MK345452, MK345453, MK968730, MN398319, MN398320, MN398321, MN398322	China (HI), Thailand
G. sinense MG279193 (11)	KF494998, MK313113, MK313115, MK313116, MK313120, MK313122, MK313128, MN398316, MN398317, MN398318	China (HI)
G. sinense MH294316 (4)	MH294302, MK172819, MK313123	China
G. sinense MK313114 (4)	MK313110, MK313111, MK313112	China
G. sinense DQ424982 (3)	DQ424990, DQ424995	
G. japonicum AY593864[75] (2)	GU213475	China
G. sinense KT906369 (2)	MG282563	China
G. sinense MH294331 (2)	MH294313	China
Singletons (18)*	AF506373, JQ520213, JQ886403, KC415760, KM249933, KT318604, KX055529, KX262895, KX262900, KX262901, MH294303, MH294332, MK313126[78], MK313127[77], MK313118[78], MK345454, X78752/X78773, Z37066/Z37103	China (GZ, SC), Taiwan
**CLADE D, Cluster D.3**		

***G. cupreum* (8)**<br>as *G. cupreum* (4), *G. australe* (1), *G.* cf. *cupreum* (1), *G. chalceum* (1), uncultured fungus (1)<br>from *Cassia* sp., *Pterocelastrus tricuspidatus*		
G. cupreum JN105701 (2)	KX055557	Cameroon
G. cupreum JN105702		Cameroon
G. australe LC084685		Malaysia
G. chalceum LK022294		Tanzania
Singletons (3)*	AB828214, KX055560, MH571696	Gabon, South Africa

***G. orbiforme* (5)**<br>as *G. orbiforme* (5)		
G. orbiforme JX310813		Brazil
G. orbiforme JX310814		Brazil
G. orbiforme JX310815		Brazil
G. orbiforme JX310816		Brazil
Singleton (1)*	MK119829	Brazil

***G. subfornicatum* (9)**<br>as *G. ecuadoriense* (5), *Ganoderma* sp. (2), *G. subfornicatum* (1), uncultured fungus (1)<br>from *Hevea brasiliensis*		
G. ecuadoriense KU128524 (3)	KU128525, KU128526	Ecuador
G. subfornicatum JX082352		French Guiana
Ganoderma sp. MH267948		Peru
G. ecuadoriense MK119827		Brazil
Singletons (3)*	KJ411557, KJ832060, MK119828	Brazil, India

***G. mastoporum* (122)**<br>as *G. australe* (60), *G. orbiforme* (19), *G. mastoporum* (12), *Ganoderma* sp. (11), *G. cupreum* (10), uncultured soil fungus (6), *G. fornicatum* (3), *G. multicornum* (1)<br>from *Acacia mangium, Acacia* sp., *Casualina* sp., *Indochinese* sp., *Prunus armeniaca, Terminalia bellerica, Trachylobium hornemannianum,* living wood, dead wood, decaying wood, soil		
G. fornicatum JX840347 (6)	LC084662, LC084702, LC084733, LC084738, LC084748	Malaysia, Taiwan
G. mastoporum MF680427 (3)	MK345446, MK968732	Laos, Thailand
G. orbiforme MG279187 (3)	MN401408, MN401409[72]	China (HI), Thailand
G. cupreum FJ655466 (2)	FJ655470	India
G. cupreum FJ655468 (2)	FJ655469	India
G. mastoporum JX840350 (2)	JN643730	China, Thailand
G. australe LC084682 (2)	LC084735	Malaysia
G. australe LC084698 (2)	LC084730	Malaysia
G. australe MH106876 (2)	MH106877	China (HI)
G. cupreum AY569450		Australia
G. australe LC084703 (2)*	LC084709	Malaysia
Singletons (94)*	AJ537399, AJ537401, AJ627585, AJ627586/7, AJ627588/9, FJ655467, GU213486, FJ655467, JN596328, JN596329, JQ409361, JX195201, JX840345, JX840346, JX840348, JX840349, JX840351, JX840352, KR709152, KT318599, KT965495, LC084660, LC084665, LC084666, LC084668, LC084669, LC084670, LC084671, LC084672, LC084673, LC084674, LC084675, LC084676, LC084679, LC084681, LC084683, LC084684, LC084686, LC084687, LC084688, LC084689, LC084690, LC084693, LC084694, LC084695, LC084697, LC084699, LC084700, LC084701, LC084704, LC084705, LC084708, LC084710, LC084712, LC084713, LC084714, LC084716, LC084719, LC084720, LC084723, LC084724, LC084732, LC084734, LC084741, LC084743, LC084744, LC084745, LC084746, LC084751, MF680428, MG279186, MG448604, MH106874, MH106875, MH106878, MK313108, MK313109, MK345445, MK345447, MK345448, MK345449, MK131241, MK589271, MK589273, MK589274, MK589275, MK589276, MK589277, MT364483, MT772000, UDB039638, UDB0767350, UDB0767363, UDB0767369, UDB0767381, UDB0767392	Australia, China (mainland, HI), India, Indonesia, Laos, Malaysia, Myanmar, Taiwan, Thailand, Vietnam

**CLADE D, Cluster D.4**		
**Group D.4.1**		
***G. angustisporum* (15)**<br>as *G. australe* (8), *Ganoderma* sp. (4), *G. angustisporum* (3)<br>from *Acacia mangium, Casuarina equisetifolia*		
G. australe LC084736 (2)	LC084747	Malaysia

G. angustisporum MG279170		China (FJ)
G. angustisporum MG279171		China (GX)
G. australe LC084664		Malaysia
G. australe LC084711		Malaysia
G. australe LC084718		Malaysia
G. australe LC084728		Malaysia
Ganoderma sp. AY569452		Australia
Singletons (6)*	KJ654552, LC084715, LC084739, MF072394, MG279172, MT449082	Australia, China (GX), Malaysia, India
***Ganoderma* sp. D1 (2) [SH1740449.08FU, SH1740450.08FU]**		
as *G. applanatum* (2)		
from *Scyphocephalium ochocoa*		
G. applanatum KY449369		Gabon
G. applanatum KY449370		Gabon
**Group D.4.2**		
***G. zonatum* (84)**		
as *G. zonatum* (84)		
from *Carpentaria acuminata*, *Cocos nucifera*, *Phoenix canariensis*, *P. roebelenii*, *P. sylvestris*, *Roystonea regia*, *Sabal maritima*, *S. palmetto*, *Syagrus romanzoffiana*		
G. zonatum KJ143921 (30)	KF605678, KJ143922, MG654376, MG654377, MG654379, MG654381, MG654385, MG654386, MG654389, MG654390, MG654391, MG654392, MG654394, MG654396, MG654397, MG654400, MG654401, MG654403, MG654404, MG654405, MG654406, MG654407, MG654411, MG654412, MG654417, MG654418, MG654422, MG654423, MG654424	USA (FL, NC)
G. zonatum MG654374 (21)	KF605679, KY646211[79], MG654378, MG654380, MG654382, MG654383, MG654388, MG654395, MG654398, MG654402[80], MG654408, MG654409, MG654410, MG654413, MG654414, MG654415, MG654416[79], MG654419, MG654420, MG654421	USA (FL, SC)
G. zonatum KX853438 (13)	KX853439, KX853441, KX853443, KX853444, KX853446, KX853449, KX853450, KX853451, KX853452, KX853455, KX853456, KX853459	USA (FL)
G. zonatum KX853436 (11)	X853437, KX853440, KX853442, KX853445, KX853447, KX853448, KX853453, KX853454, KX853457, KX853458	USA (FL)
G. zonatum MG654425 (2)	MG654426	USA (FL)
Singletons (7)*	KY646212[80], KY708886[81], MG654375, MG654384, MG654387, MG654393[81], MG654399	USA (FL)
***Ganoderma* sp. D2 (4) [SH1723113.08FU]**		
as *Ganoderma* sp. (4)		
from *Elaeis guineensis*		
Ganoderma sp. KJ792083		Colombia
Ganoderma sp. KJ792084		Colombia
Ganoderma sp. KJ792085		Colombia
Ganoderma sp. KJ792086		Colombia
***G. ryvardenii* (16)**		
as *G. ryvardenii* (13), *Ganoderma* sp. (3)		
from *Cassia* sp., *Elaeis guineensis*, decaying wood		
G. ryvardenii HM138671 (10)	HM138670, HM138672, JN105691, JN105692, JN105694, JN105695, JN105696, JN105697, JN105699	Cameroon
G. ryvardenii JN105693		Cameroon
G. ryvardenii JN105698		Cameroon
Ganoderma sp. LK022295		
Singletons (3)*	JN105706, MN784437, MN784438	Cameroon
***G. boninense* (61)**		
as *Ganoderma* sp. (29), *G. boninense* (26), *G. miniatocinctum* (2), *G. orbiforme* (2), *G. zonatum* (2)		
from *Areca catechu*, *Areca* sp., *Elaeis guineensis*, *Wodyetia bifurcata*		
G. boninense KY471677 (3)	MG200172, MG200173	Indonesia
G. boninense AY220540 (2)	AY220542	
G. miniatocinctum MN490055 (2)	KM220586	Malaysia
G. boninense KJ143906 (2)	BD082759	Japan
G. orbiforme JX997990 (2)	KX421867	Thailand
Ganoderma sp. AY220543 (2)	KY471674	Indonesia
Ganoderma sp. JN234427 (2)	KY471687	Indonesia, Malaysia

Ganoderma sp. KY352306 (2)	JN400510	Malaysia
Ganoderma sp. MN396662 (2)	MN398324	Vietnam
G. boninense KJ143905		Japan
Singletons (41)*	AB985729, AF255196/7, AY220537, AY220538, AY220539, AY220541, BD082757, BD082758, EF016754, EU239386, JN234428, JN234429, JN400511, JN400513, JN400515, KF164430, KM015454, KM220584, KM271997, KX092000, KX499467, KY471673, KY471680, KY471683, KY471685, KY471686, KY471688, MK605934, MG650116, MK713555, MK713556, MK713557, MK713558, MK713559, MK713560, MK713561, MN148580, MT487851, MN490048, MN490052, MN490054	China, Indonesia, Malaysia, Taiwan, Thailand

***Ganoderma* sp. D3 (12) [SH1723050.08FU, SH1723098.08FU]**<br>as *Ganoderma* sp. (12)<br>from *Elaeis guineensis*		
Ganoderma sp. KY471675 (2)	KY471689	Indonesia
Ganoderma sp. KY471676 (2)	KY471684	Indonesia
Ganoderma sp. JN400512		
Ganoderma sp. JN400514		
Ganoderma sp. KY471678		Indonesia
Ganoderma sp. KY471679		Indonesia
Ganoderma sp. KY471681		Indonesia
Ganoderma sp. KY471682		Indonesia
Singletons (2)*	AY220544, JN400509	

**CLADE E, Cluster E.1**		
***G. williamsianum* (42)**<br>as *G. australe* (29), *G. williamsianum* (7), *G.* cf. *australe* (2), *G. australe* cplx (2), *Ganoderma* sp. (1), uncultured fungus (1)<br>from *Acacia mangium*		
G. williamsianum MG279168 (5)	KJ654367, KJ654444, KJ862063, MG279183	Thailand
G. williamsianum KU219995 (4)	KJ654402, KJ654445, LC084706	China, Malaysia
G. australe KJ654371 (3)	KJ654400, KJ654401	
G. australe KJ654366 (2)	KJ654397	
G. australe KJ654441 (2)	KJ654442	
G. australe cplx AF255146 (2)	AF255147	Thailand
Singletons (24)*	JN596326, JN596327, KJ654368, KJ654369, KJ654370, KJ654398, KJ654399, KJ654403, KJ654443, KU219994, LC084661, LC084667, LC084680, LC084691, LC084696, LC084721, LC084726, LC084727, LC084742, MF942545, MG279169, MH071336	China (HI), Malaysia, Myanmar, Thailand

**CLADE E, Cluster E.2**		
***Ganoderma* sp. E1 (23) [SH1723047.08FU]**<br>as *G. applanatum* cplx (8), *G. tornatum* (6), *Ganoderma* sp. (4), *G. lobatum* (3), *G. gibbosum* (1), *G. parvulum* (1)<br>from *Hevea guianensis*, *Scutia buxifolia*		
G. applanatum cplx AF255130 (5)	AF255139, KF605669, KF605672, MH267949	Ecuador, Peru, USA (FL)
G. applanatum cplx AF255136		Costa Rica
G. tornatum JQ514108		Brazil
G. tornatum KU948516		
Singletons (15)*	AF255134, AF255135, AF255137, AF255138, AF255140, AF255141, JQ514107, JQ514109, JQ514110, JX082353, JX082354, KU569529, KU948517, MT232631, MT232633	Brazil, Colombia, Costa Rica, Ecuador, French Guyana, Mexico

***Ganoderma* sp. E2 (37) [SH1723047.08FU]**<br>as *G. gibbosum* (12), *G. tornatum* (8), *G. lobatum* (7), *Ganoderma* sp. (6), *G. applanatum* cplx (2), *G. australe* (2)<br>from *Cenostigma pluviosum* var. *peltophoroides*, *Elaeis guineensis*, *Jacaranda mimosifolia*, *Leucaena leucocephala*, coniferous tree, decaying wood		
G. gibbosum KU569535 (11)	JQ514100, JQ514101, KU569543, KU569544, KU569548, KU569550, KU569551, KU569552, KU569554, KU569557	Brazil
G. lobatum KF605673 (2)	KF605675	
Ganoderma sp. LT726721 (2)	LT726722	Cuba
Ganoderma sp. LT726724 (2)	LT726727	Cuba
G. lobatum KF605671		
Singletons (19)*	AH008101, AF255131/2, AF255133, HM192933, JQ514102, JQ514103, JQ514104, JQ514105, JQ514106, KF605670, KF605674, KF605676, KF605677, KU315203, KU569537, KU569547, KU569556, LT726723, MF347411	Argentina, Brazil, Colombia, Cuba, Puerto Rico, USA (FL)

***G.* aff. *gibbosum* (46)**<br>as *Ganoderma* sp. (43), *G. australe* (2), *G. gibbosum* (1)<br>from *Acacia auriculiformis*, *Albizia lebbeck*, *Areca catechu*, *Casuarina equisetifolia*, *Cocos nucifera*, *Tamarindus indica*, *Thespesia populnea*, living wood		
G. gibbosum JN655531 (36)	KM229635, KM229637, KM229639, KM229640, KM229641, KM229642, KM229643, KM229644, KM229646, KM229647, KM229648, KM229649, KM229650, KM229651, KM229652, KM229653, KM229654, KM229655, KM229656, KM229657, KM229658, KM229662, KM229663, KM229664, KM229665, KM229667, KM229668, KM229669, KM229670, KM229671, KM229672, KM229675, KM229676, KM229677, KR154930	India
G. australe AY993920/1		India
Ganoderma sp. KM229638		India
Ganoderma sp. KM229673		India
Singletons (7)*	AY968692/AY993913, KM229634, KM229636, KM229645, KM229659, KM229666, KM229674	India
***G. eickeri* (4)**<br>as *G. eickeri* (2), *Ganoderma* sp. (2)<br>from *Acacia cyclops*, *Celtis africana*, *Prunus africana*		
G. eickeri MH571690/ NR_165524 (2)	MH571689	South Africa
Ganoderma sp. MG020264		South Africa
Ganoderma sp. MG020265		South Africa
***G. gibbosum* (107)**<br>as *G. gibbosum* (59), *G. applanatum* (27), *G. australe* (8), *Ganoderma* sp. (3), *G. australe* cplx (2), *G. australe* IG1 (2), *G. lingzhi* (2), Agaricales sp. (1), *Fuscoporia viticola* (1), *G. fulvellum* (1), *G. lucidum* (1)<br>from *Acacia* sp., *Albizia lebbeck*, *A. molli*, *Citrus unshiu*, *Dendrocalamus* sp., *Dipterocarpus* sp., *Machilus* sp., *Mangifera* sp., *Pinus* sp., decaying wood		
G. gibbosum KY364259 (46)	AB733121, AY593856[82], DQ424996, DQ425009, EU273513, FJ478088, FJ582638, JX195200, KM249934, KM249936, KY364260, KY364261, KY364262, KY364263, KY364264, KY364265, KY364266, KY364267, KY364268, KY364269, KY364270, KY364271, KY364272, MG279179, MG657365, MH035681, MH035682, MH035685, MH114668, MK079623, MK280717, MK345432, MN294816, MN396310, MN396311, MN396312, MN396313, MN396314, MN396315, MN398341, MN398342, MN398343, MN398344, MN622779, MN622799	China (GZ, SC, YN), Japan, Laos, Pakistan, South Korea, Taiwan, Thailand
G. applanatum GU213473 (5)	JX195197, MG657363, MG657364, MH035688	China
G. gibbosum MH106880 (4)	EU273557, MG719604, MK345433	China (HB, HI, GZ)
G. applanatum AF255114 (2)	MK268928	China, South Korea
G. australe GU213474 (2)	JX195196	China
G. gibbosum EU273514 (2)	EU326219	
G. applanatum MG657361 (2)	MG657366	China
G. gibbosum MH035684 (2)	MH114667	
G. gibbosum MN622780 (2)	GU213472	
Singletons (40)*	AF255105, AF255106/7, AF255108/9, AF255110/1, AF255112/3, AF506372, AY593854[82], AY593855[82], AY593857[82], EU273555, EU326218, EU918695, HQ891299, JN008873, JX195199, KF494999, KF574247, KJ195663, KM249935, KM609399, KR673513, KX879638, MG231526, MG657360, MG657362, MH035683, MH035687, MH507186, MK268927, MK343538, MK345434, MK345436, MK370672, MK605939, MK809458, MN238820, MN238821, MN294853, MN523326, X78741/X78762, X78750/X78771	China (YN), Japan, Laos, South Korea, Taiwan, Thailand
***G. ellipsoideum* (73)**<br>as *G. gibbosum* (19), *G. australe* cplx (10), *G. australe* (9), *Ganoderma* sp. (8), *G. adspersum* (5), *G. applanatum* (5), *G. ellipsoideum* (5), uncultured soil fungus (5), *G. applanatum* cplx (3), *G.* aff. *steyaertanum* (1), *G. tornatum* (1), uncultured *Ganoderma* (1), *Tomophagus* sp. (1)<br>from *Casuarina equisetifolia*, *Coelogyne viscosa*, *Diospyros buxifolia*, *Gleditsia fera*, *Hevea brasiliensis*, *Mangifera indica*, *Mangifera* sp., *Persea americana*, *Pterocarpus* sp., *Trachylobium hornemannianum*, bamboo, living wood, dead wood, decaying wood, roots, soil		
G. gibbosum MH114670 (7)	KP861881, MK345425, MN396652, MN396653, MN396654, MN396655	Laos, Thailand
G. applanatum cplx AF255116 (5)	AF255117, KP012934, KR632638, MK131240	Australia, China (YN), Indonesia

G. australe cplx AF255119 (3)	AF255115, KU194311	China (HK, YN), Thailand
G. ellipsoideum MH106867/ NR_160617 (2)	MK333264	China (HI)
G. ellipsoideum MH106886 (2)	KT318591	China (HI)
G. ellipsoideum MN398339 (2)	EU239387	Indonesia, Thailand
G. gibbosum KJ654404 (2)	KJ654405	
G. gibbosum KU194326 (2)	KU194305	China (HK)
G. gibbosum MN523324 (2)	MN523325	China
uncultured soil fungus UDB0763546 (2)	UDB0769802	USA
Singletons (44)*	AF255118, AF255120, AF255121, AF255122, AF255123/4, AF255125, AF255126/7, AF255128, AF255129, FJ392286, JN596331, JX195204, JX195205, KF574247, KJ654372, KJ654373, KJ654446, KJ767488, KM229678, KR867655, KT965498, KU194307, KU194312, KU194347, LC084663, LC084717, LC084749, MF072395, MG448603, MG719303, MH101644, MH106868, MH114669, MK131238, MK131242, MK345435, MK404342, MN689631, MN696209, MN747808, MT252597, UDB0755339, UDB0763169, UDB0777839,	Cambodia, China (HI, HK), India, Indonesia, Laos, Malaysia, Myanmar, Papua New Guinea, Sri Lanka, Thailand, Vietnam
***Ganoderma* sp. E3 (7) [SH1723116.08FU, SH1723270.08FU]**		
as *G. australe* (6), uncultured soil fungus (1)		
from soil		
G. australe KJ654550 (2)	KJ654551	
G. australe EU239383		Indonesia
G. australe EU239390		Indonesia
G. australe FR821768		Australia
Singletons (2)*	KJ654549, UDB0755590	Australia
***Ganoderma* sp. E4 (13) [SH1677211.08FU]**		
as *G. australe* (12), *G. tornatum* (1)		
from *Elaeis guineensis*, *Eucalyptus pellita*		
G. australe AF255142 (2)	AF255144	Malaysia
G. australe KJ654545 (2)	KJ654547	
G. australe AF255143		Malaysia
G. australe AF255145		Malaysia
Singletons (7)*	EU239389, KJ654379, KJ654544, KJ654546, KJ654548, KR093031, MN490058	Indonesia, Malaysia
**CLADE E, Cluster E.3**		
***G. knysnamense* (4)**		
as *G. knysnamense* (4)		
from *Olea capensis* subsp. *macrocarpa*		
G. knysnamense MH571681(2)	MH571682	South Africa
G. knysnamense MH571683		South Africa
G. knysnamense MH571684		South Africa
***G. mutabile* (2)**		
as *G. mutabile* (2)		
G. mutabile JN383977		China (YN)
G. mutabile MG231527		
***G. cupreolaccatum* (1)**		
as *G. cupreolaccatum* (1)		
G. cupreolaccatum MH856316		
***G. pfeifferi* (17)**		
as *G. pfeifferi* (17)		
from *Fagus sylvatica*		
G. pfeifferi MK415258 (5)	MK415259, MK415290, MK415296, MK415310	Slovakia
G. pfeifferi AM906059 (3)	KF605659, MG279164	Czech Republic
G. pfeifferi **MG706232** (3)	JN008874, KF605660	Greece
G. pfeifferi AY884185 (2)	JN222420	UK
Singletons (4)*	AY884181, MG279165, MK415263, MK415295	Slovakia, UK
**CLADE E, Cluster E.4**		
***G. chocoense* (1)**		

as *G. chocoense* (1) from decaying wood		
G. chocoense MH890527/NR_163763		Ecuador
***G. podocarpense* (2)**		
as *G. podocarpense* (1), uncultured soil fungus (1) from dead wood, soil		
G. podocarpense MF796661		Ecuador
uncultured soil fungus UDB0759513		Panama
***Ganoderma* sp. E5 (8) [SH1678465.08FU]**		
as *Ganoderma* sp. (4), *G. lobatum* (2), *G. tornatum* (1), uncultured soil fungus (1) from *Salix* sp., soil		
Ganoderma sp. AF255184 (4)	AF255185, AF255186, AF255187	Argentina
G. lobatum AH008103 (3)	AH008104, AH008105	Argentina
uncultured soil fungus UDB0768776		Argentina
***Ganoderma* sp. E6 (35) [SH1723070.08FU]**		
as *G. australe* (18), *Ganoderma* sp. (8), *G. australe* IG2 (2), *G. applanatum* (2), *G. australe* cplx (1), *G.* cf. *australe* (1), *G.* cf. *philippii* (1), uncultured soil fungus (1) from *Dipterocarpus* sp., *Fagus* sp., *Mangifera* sp., *Neocinnamomum* sp., *Pterocarpus* sp., decaying wood, soil		
G. australe MN396657 (6)	MK345428, MN396660, MN396661, MN398335, MN398336	China, Laos, Thailand
G. australe MH106871 (4)	MN396656, MN396658, MN39665	China (HI)
Ganoderma sp. KU219992 (2)	KU219993	China
G. australe MK968731 (2)	AF255188	Vietnam
Ganoderma sp. MK605938 (2)	MK605945	Taiwan
Singletons (17)*	AF255189, AF255190, AF255191/2, AF255193/4, AF255195, AY993918/9, EU232190, JN643731, JX195198, KT318590, KT965497, LT716076, MH106872, MK182313, MK605943, MK774700, UDB0755383 AF255183*	China (HI), India, New Zealand, Papua New Guinea, Taiwan, Thailand, Vietnam
***G. australe* (76)**		
as *G. australe* (26), *G. australe* cplx (14), *Ganoderma* sp. (13), uncultured soil fungus (8), *G. annulare* (2), *G. applanatum* cplx (2), *G. brownii* (2), *G. lobatum* (2), *G. tornatum* (2), *G. adspersum* (1), *G. applanatum* (1), *G. lipsiense* (1), *G. lucidum* (1), fungal sp. (1) from *Acacia mangium*, *A. melanoxylon*, *Arachnitis uniflora*, *Eucalyptus* sp., *Ficus* sp., *Nothofagus alpina*, *Podocarpus* sp., *Pterocelastrus tricuspidatus*, *Quercus agrifolia*, *Umbellularia californica*, *Vaccinium corymbosum*, *Vaccinium* sp., living wood, dead wood, decaying wood, soil		
G. australe KU569531 (18)	AF255149, AF255150, JQ520160, KF605664, KF605665, KU569532, KU569533, KU569534, KU569536, KU569538, KU569539, KU569541, MH571685, MH571686, MH571687, MH571688, UDB0762229	Argentina, Brazil, South Africa
Ganoderma sp. AF255176 (8)	AF255177, AM269768, AM269769, KF605661[83], KF605662, MG279159[83], MK322289	Chile, USA (CA)
G. australe cplx AF255157 (4)	AF255158, HM583824, UDB0768220	Australia
G. tornatum AH008096 (3)	AH008097, AH008098	Argentina
G. australe cplx AF255164/5 (3)	AF255174/5, AF255178/79	Argentina, Chile, New Zealand
G. australe cplx AF255151/2 (2)	AF255153/4	Australia
G. australe cplx AF255160 (2)	UDB0767764	New Zealand
G. australe AY884180 (2)	AJ006685	UK
Fungal sp. MH242613 (2)	UDB0767741	New Zealand
Ganoderma sp. MK788298 (2)	MK934577	Chile
Singletons (30)*	AH008099, AH008100, AH008102, AH008106, AF255148, AF255155/6, AF255159, AF255161/2, AF255163, AF255166/7, AF255168/9, AF255170/1, AF255172/3, AF255180, AF255181/2, AJ608709, AJ627590/1, AJ627592/3, KF605663, KU569540, KU569545, MF436675, MF436676, MK826006, MK826012, MK883702, UDB0759941, UDB0766747, UDB0767760, UDB0768715	Argentina, Australia, Brazil, Chile, Costa Rica, New Zealand, USA
**CLADE E, Cluster E.5**		
***Ganoderma* sp. E7 (16) [SH1723077.08FU]**		
as *Ganoderma* sp. (10), *G. lobatum* (3), *G. applanatum* cplx (2), uncultured soil fungus (1)		
Ganoderma sp. AF255099 (4)	AF255100, KF605654, KF605655	USA (NC)
G. lobatum X78740/X78761		USA
Ganoderma sp. KF605653		
Ganoderma sp. KF605656		

Singletons (9)*	KF605657, KF605658, KF800469, MN749647, MN749648, MN906144, MN906145, MN964001, MN964002	USA (AR, IN)

***G.* aff. *adspersum* (9)**<br>as *G. adspersum* (4), *G. applanatum* (2), *G. australe* cplx (2), uncultured soil fungus (1)<br>from soil		
G. adspersum KY364251 (4)	KY364252, KY364253, KY364254	South Korea
G. applanatum JQ520161		Japan
G. australe cplx AF255104		Japan
G. australe cplx AF255102/3		Japan
Singletons (2)*	MN080319, UDB0765962	China, South Korea

***G. adspersum* (142)**<br>as *G. adspersum* (112), *G. australe* (13), *Ganoderma* sp. (9), *G. applanatum* (4), basidiomycetes sp. (2), uncultured fungus (1), uncultured *Ganoderma* (1)<br>from *Abies cephalonica*, *A. nordmanniana*, *Abies* sp., *Acrocarpus fraxinifolius*, *Aesculus hippocastanum*, *Alnus glutinosa*, *Amygdalus communis*, *Artocarpus heterophyllus*, *Borassus flabellifer*, *Castanea sativa*, *Cercis siliquastrum*, *Fagus sylvatica*, *Ficus benghalensis*, *Fraxinus angustifolia*, *Fraxinus* sp., *Grevillea parallela*, *Juglans regia*, *Laurus nobilis*, *Melia azadirachta*, *Morus* sp., *Pinus pinea*, *P. taede*, *Pinus* sp., *Platanus orientalis*, *Populus alba × tremula*, *Prunus armeniaca*, *P. avium*, *P. dulcis*, *Pterocarya fraxinifolia*, *Quercus ilex*, *Q. pubescens*, *Quercus* sp., *Robinia* sp., *Salix* sp., *Sorghum bicolor*, *Tamarindus indica*, *Terminalia bellerica*, *Tetrameles nudiflora*		
G. adspersum **MG706203** (54)	**MG706205, MG706206, MG706207, MG706208, MG706209, MG706210, MG706211, MG706212**, AF255101, AF455510, AM269767, AM269770, AM269771, AM906055, AM906056, AM906057, AY605704, AY605708, AY884183[84], AY884184, EF060009, EF060010, EF060011, EF060012, FR686555, JN176879, JN176883, JN176884, JN176887, JN176888, JN176889, JN176892, JN176894, JN176908, JN222417, JN222418, KF605651, KF605652, KP941437, KP941438, KP941440, KP941442, KX449505, KY977553, MG066632, MG279152, MG279153, MG835864, MK050589, MK050593, MN218201, MN218202, MN218203	Armenia, Belgium, Croatia, France, Georgia, Germany, Greece, Iran, Italy, UK, USA (CA)
G. adspersum MK415250 (15)	MK415257, MK415265, MK415268, MK415288, MK415289, MK415291, MK415292, MK415293, MK415294, MK415301, MK415302, MK415303, MK415306, MK415307	Slovakia
G. adspersum KT343306 (5)	KT343311, KT343312,KT343313, KT343315	Iran
G. adspersum JN588581 (4)	JN588582, JN588583, JN588584	Armenia
G. adspersum JN588585 (3)	JN588586, KP941439	Armenia, Iran
G. australe FJ655459 (3)	FJ655460, FJ655462	India
G. adspersum FJ655451 (2)	FJ655452	India
G. adspersum MG279154 (2)	KP941436	Armenia, Iran
G. adspersum JN176901		Italy
G. adspersum JN176902		Italy
G. australe FJ655461 (2)*	FJ655463	India
G. australe FJ655464 (2)*	FJ655465	India
G. adspersum JN176890 (2)*	JN176898	Italy
Ganoderma sp. KT343298 (2)*	KT343302	Iran
Singletons (44)*	**MG706204**, AM906054, AY884182, EF060008, EU162053[84], FJ655447, FJ655449, FJ655453, FJ655458, FJ820612, GU731554, JN176886, JN176891, JN176893, JN176903, JN176904, JN176905, JN176906, JN176907, JN222416, JN588579, JN588580, KF605650, KF975893, KP974213, KP941441, KT343300, KT343305, MK018627, MK050589, MK050590, MK050591, MK050592, MK050594, MK415256, MK415262, MK415280, MK415300, MK415315, MK422152, MN121020, MN945139, MT138615, X78742/X78763	Armenia, Belgium, Georgia, Germany, Greece, India, Iran, Italy, Slovakia, Spain, Tunisia, UK, USA (CA)

**Outgroups**		
<u>*G. sandunense* MK345450/NR_164049</u>		China
G. sandunense MK345451		China
G. shandongense MG279191 (4)	EU918700, FJ478127, MG279192	
G. shandongense MG279190		
G. *coffeatum* KU315204		Brazil
H. *coffeata* MH124633		
G. *tsunodae* FJ154771		
G. *tsunodae* FJ154773		
G. subressinosum KJ654468 (3)	KJ654471, KJ654472	

G. subressinosum JQ409358 (9)	KJ654376, KJ654406, KJ654465, KJ654466, LC176756, LC176784, LC176785, MF680429	Malaysia, Thailand
T. colossus JQ618247 (2)	JX310825	Brazil
T. colossus JN184396 (5)	FJ154767, KR155077, MG654431, MG650109	India, Vietnam
T. cattienensis JN184398 (2)	KJ143923	Vietnam
Pycnoporus cinnabarinus JN645086 (5)	AF363756, AF363757, AF363764, AF363765	France
Trametes versicolor JQ070098 (2)	KF573030	USA

Singletons and ASVs marked with asterisk appear only on the expanded/detailed phylogenetic trees (Supplementary Material, Figs. S2a to S2f). Abbreviations for geographic regions in USA and China follow the two-character ISO country codes, i.e., https://www.iso.org/obp/ui/#iso:code:3166:US and https://www.iso.org/obp/ui/#iso:code:3166:CN, respectively. The entries with the same serial number in superscript typeface represent sequence data of the same biological material (or clones) according to their specimen/voucher/strain code appearing in the present Table and in Supplementary Table S4.

Table S3. *Ganoderma* sequences excluded from the analysis since they were either erroneously labelled as *Ganoderma* or whose identity could not be reliably resolved.

	**GenBank accession numbers**
**Sequences erroneously assigned as *Ganoderma* (58)**	AF493072, AJ536659, AJ536660, AJ536664, AJ537402, AJ542516, AJ542517, EF139861, EU232219, EU735845, FJ627253, GU207320, HM602034, HM622152, HQ328947, HQ589215, HQ589226, JQ040846, JQ520163, JQ520165, JQ520192, JQ520217, JQ675674, KC505552, KJ670275, KM375927, KM609397, KM609402, KR155022, KR155025, KR155124, KR155125, KR349638, KR349639, KR349640, KT188607, KX008366, KX224487, KX953305, KY244067, MG489963, MG564643, MG712327, MG751292, MH035686, MK019084, MN394885, MN523257, MN636775, MN636777, MN636778, MN636779, MN636780, MN636781, MN636782, MT534033, MT534034, Z37063/Z37105
**not identified entries (4)**	KJ654380, KJ726747, KY449371, KY449372

Supplementary Table S4. *Ganoderma* sequences (642) included in the data analysis but excluded from the trees inferred (Figs. 4 to 7, and Figs. S2a to Fig. S2f) due to overrepresentation of certain species (i.e., > 100 singletons for *G. lingzhi*, *G. multipileum* and *G. applanatum*) or to their particularly high heterogeneity. Presentation of data follows the format adopted in Supplementary Table S2.

**Clade A**		
**Cluster A.1.**		
***G. tsugae* (3)**		
as uncultured *Ganoderma* (3)	KU712531, KU712533, KU712534	India
***G. lucidum* (1)**		
as uncultured soil fungus (1)	UDB0226767	Estonia
**Cluster A.2.**		
***G. weberianum* (1)**		
as *G. weberianum* (1)	HQ267232	
***G. mexicanum* (1)**		
as *G. weberianum* (1)	MT232642	Mexico
***G. parvulum* (1)**		
as *G. weberianum* (1)	JN596332	
***G. resinaceum* (5)**		
as *G. resinaceum* (3), *G. lucidum* (2)<br>from *Quercus* sp.	MG835866, MK773879, MN114053, MT703901, MT708677	Turkey
***G.* aff. *polychromum* (2)**		
as *G. resinaceum* (2)	FJ850966, FJ850967	
***G. sessile* (1)**		
as *G. lucidum* (1)	MK773881	
**Cluster A.3.**		
***G. tuberculosum* (1)**		
as *G. tuberculosum* (1)	MT232636	Mexico
***G. G. wiiroense* (1)**		
as *Ganoderma* sp. (1)	KY968687	India
***G. lingzhi* (179)**		
as *G. lingzhi* (89), *G. lucidum* (75), *G. sichuanense* (7), *Ganoderma* sp. (5), *G. tsugae* (3)<br>from decaying wood	AB733122, AB733174, AB733175, AF506371, DQ424969, DQ425012, EF188277, EF188280, EU520155, EU520235, EU520137, EU520247, FJ379263, FJ381957, FJ501553, HQ222603, HQ222604, HQ689695, HQ689697, JF915398, JF915401, JF915404, JN222421, JQ520167, JQ520170, JQ520174, JQ520175, JQ520177, JQ520178, JQ520179, JQ520210, JQ520214, JQ781866, JX162755, JX162754, JX162756, JX162757, JX162758, JX162759, JX162760, JX162761, JX162762, JX162763, JX162764, JX162765, JX162766, JX162767, JX162768, JX162770, KC222318, KC414242, KC505544, KM249916, KM249926, KM249930, KT185665, KT247417, KT318603, KT343299, KT693248[85], KT693249[85], KT693254[50], KT717953, KT906367, KT921216, KX055537, KX055538, KX262896, KX262897, KX262898, KX262899, KX262902, KX262904, KX262905, KX262906, KX262907, KX358402, KX358403, KX580183, KY244064, LC090753, MF770158, MG457485, MG732940, MG732941[53], MG732942, MG732943, MG732944, MG732945, MG732946[61], MG732947, MG732948[54], MG732949, MG732950, MG732951, MG732952, MG732953, MG732954, MG732955[54], MG732956, MG732957, MG732958, MG732959, MG732960, MG732961, MG732962, MG732963, MG732964, MG732965, MG732966, MG732967, MG732968, MG732969, MG732970[56], MG732971, MG732972, MG732973, MG732974, MG865278, MG865279, MG865280, MG865281, MH018023, MH018024, MH018026, MH018029, MH018031,	China (HI, SC), India, Iran, Japan, South Korea, Taiwan, Thailand, commercial

	MH109554, MH109562, MH109572, MH109587, MH109600, MH109605, MH109612, MH109616, MH109648, MH109650, MH109651, MH109652, MH109654, MH109660, MH109666, MH109667, MH109668, MH109669, MH109677, MH160065, MH160067, MH160070, MH160074, MH160079, MH160080, MH160081, MH160082, MH160086, MH479057, MK209603, MK282240, MK589272, MK589278, MK773880, MK782568, MK855511, MN372058, MN372059, MN372060, MN372061, MN372062, MN372066, MN372067, MN596944, MN636776, MN888753, MT196416, MT879644, X78746/X78767, X78747/X78768, X87352/X87362, X87354/X87364, Z37050/Z37074, Z37097/Z37078	
***G. curtisii* (1)**		
as *G. curtisii* (1)	MT232640	Mexico
***G. multiplicatum* (2)**		
as *G. multiplicatum* (1), *G. perzonatum* (1)	KJ792751, MT396669	
***G. multipileum* (113)**		
as *Ganoderma* sp. (53), *G. lucidum* (52), *G. multipileum* (5), Agaricales sp. (1), *G. lingzhi* (1), uncultured *Ganoderma* (1) from *Acacia nilotica*, *Acacia* sp., *Acrocarpus* sp., *Areca catechu*, *Azadirachta indica*, *Azadirachta* sp., *Cassia fistula*, *Casuarina equisetifolia*, *Cocos nucifera*, *Colophospermum mopane*, *Dalbergia sissoo*, *Delonix regia*, *Ficus benghalensis*, *Jacaranda mimosifolia*, *Mangifera indica*, *Melia azadirachta*, *Parkia biglandulosa*, *Prosopis cineraria*, *Vachellia nilotica*, *V. tortilis*, decaying wood, soil, roots	AY636058, AY636064, AY636068, AY636069, AY636070, DQ425000, EU021459, FJ463917, FJ463925, GQ249885, GU726920, GU726922, GU726924, HM053444, HM053445, HM053449, HM053450, HM130706, HQ589218, HQ589225, JN099386, JN613281, JN692272, KC807228, KF549493, KF998089, KF998092, KF998093, KF998094, KJ143913, KJ670301, KJ670304, KJ868083, KM229569, KM229571, KM229573, KM229575, KM229578, KM229581, KM229586, KM229587, KM229590, KM229593, KM229597, KM229598, KP886807, KR154928, KR154942, KR154950, KR154952, KR154953, KR154955, KR155020, KR155021, KR155106, KR155073, KR155074, KR155078, KR349633, KT188589, KT188597, KT188598, KT188599, KU194309, KU712535, KX388170, KX610998, KY009865, KY009870, KY009871, KY009872, KY471289, KY865255, KY865256, KY865257, MF289193, MF405163, MF958936, MH290248, MH290250, MH290253, MH290261, MH290262, MH290264, MH290265, MH290266, MH290268, MH290271, MH290279, MK079627, MK849887 (replaced MK773878), MH983559, MK940283, MK940284, MK940285, MK940288, MN006955, MN372063, MN372064, MN372065, MT765061, MT765063, MT765065, X78743/X78764, X78744/X78765, X78745/X78766, X87345/X87355, X87346/X87356, X87347/X87357, X87348/X87358, X87349/X87359, X87350/X87360, X87351/X87361, Z37048/Z37072	China, India, Pakistan, Philippines, Taiwan, commercial
***G. martinicense* (1)**		
as *G. martinicense* (1)	MT232632	Mexico
***G. tropicum* (2)**		
as *G. tropicum* (2)	MT364498, MT364499	
**CLADE B**		
***G. applanatum* (266)**		
as uncultured soil fungus (201), *G. applanatum* (29), Fungi (plant leaf) (15), uncultured *Ganoderma* (9), *G. lipsiense* (3), *G. applanatum* cplx (2), *Ganoderma* sp. (2), *G. adspersum* (1), *G. australe* (1), *G. lobatum* (1), *Trametes* sp. (1), uncultured fungus (1) from *Albizia lebbeck*, *Alnus glutinosa*, *Berberis thunbergii*, *Corylus avellana*, *Crataegus punctata*, *Eucalyptus gunnii*, *Fagus sylvatica*, *Ficus lyrata*, *Gleditsia*	AF255092, AF255095, AF255097, AY968082, DQ421100, DQ421109, DQ421110, DQ421242, EF060000, EF060004, FJ197949, FJ655456, FJ655457, GU083147, GU731556, HQ021834, HQ021836, HQ021975, HQ022119, HQ022243, JN048773, JQ520206, JQ666446, JQ666495, JQ666507, JQ666577, JQ666595, JQ666726, JX675211, JX675216, JX898575, KC505588, KF495001, KF605648, KF605649, KF800100, KJ857258, KJ857265, KJ857266, KR673611, KT318588, KT318589, KU219987, KU948520, KY364255, MF143513, MF161177, MF161255, MF161295, MF686522, MG279155, MG279156, MH114666, MH930900, MK050641, MK345427, MK393922, MN435133, MN435146, MN906143, MN992389, UDB0100231, UDB0103060, UDB0119647, UDB0121485, UDB0122574, UDB0124542, UDB0126096, UDB0130744, UDB0132569, UDB0133733, UDB0134557, UDB0135708, UDB0139875, UDB0142408, UDB0149952, UDB0151129, UDB0152824, UDB0157946, UDB0161689, UDB0167266, UDB0167717, UDB0170440, UDB0179882, UDB0181758, UDB0184967, UDB0186902, UDB0187261, UDB0189759, UDB0201672, UDB0203037, UDB0214566, UDB0215659, UDB0216981, UDB0222995, UDB0224112, UDB0225875, UDB0228887, UDB0232284, UDB0240104, UDB0241972, UDB0247307, UDB0253438, UDB0257893, UDB0261000,	Austria, Canada, China (YN), Czech Republic, Estonia, Hungary, India, South Korea, Kyrgyzstan, Russia, USA (AK, IN, MD, MO, MS, NH, OR, TN, VI)

triacanthos, *Grevillea parallela*, *Phoenix canariensis*, *Pinus tabuliformis*, *Salix alba*, *Salix alba x Salix fragil*, *Sambucus pubescens*, *Thuja occidentalis*, *Tilia cordata*, *Tilia* sp., *Tradescantia zanonia*, *Ulmus glabra*, decaying wood, soil	UDB0261967, UDB0265157, UDB0266885, UDB0267759, UDB0268901, UDB0275826, UDB0276847, UDB0280808, UDB0287464, UDB0295117, UDB0310323, UDB0311723, UDB0322707, UDB0328840, UDB0328942, UDB0331720, UDB0338913, UDB0343801, UDB0351962, UDB0359085, UDB0374592, UDB0374866, UDB0376096, UDB0376950, UDB0388764, UDB0395610, UDB0400307, UDB040056, UDB0401075, UDB0405032, UDB0407497, UDB0408981, UDB041383, UDB0418862, UDB0420804, UDB0421269, UDB0422666, UDB0422845, UDB042325, UDB0423702, UDB0428244, UDB0429138, UDB0430744, UDB0435574, UDB0437183, UDB0438491, UDB0440440, UDB0446806, UDB0454763, UDB0456937, UDB0455904, UDB0456406, UDB0456671, UDB0461993, UDB0463272, UDB0465624, UDB0466209, UDB0467477, UDB0469923, UDB0481250, UDB048343, UDB0484438, UDB0489241, UDB0490998, UDB0492431, UDB0492497, UDB0493384, UDB0497080, UDB0501364, UDB0502251, UDB0504358, UDB0504458, UDB0512395, UDB0512540, UDB0514622, UDB0515632, UDB0518466, UDB0532721, UDB0536161, UDB0541946, UDB0544560, UDB0557948, UDB0566431, UDB0576874, UDB0578535, UDB0578737, UDB0580479, UDB0583647, UDB0584468, UDB0608795, UDB0613546, UDB0613654, UDB0615105, UDB0616935, UDB0622788, UDB0623034, UDB0626374, UDB0630541, UDB0635185, UDB0639460, UDB064483, UDB0647100, UDB0648395, UDB0650310, UDB0652930, UDB0654560, UDB0655465, UDB0658413, UDB0659247, UDB067029, UDB0670319, UDB0670667, UDB0679640, UDB0680005, UDB0680531, UDB0682224, UDB0686694, UDB0688255, UDB0690903, UDB069370, UDB0694220, UDB0699166, UDB070645, UDB0706520, UDB0710049, UDB0715464, UDB0729284, UDB074240, UDB0746367, UDB0746719, UDB0747059, UDB0747170, UDB074757, UDB0748354, UDB0749040, UDB0749357, UDB0749672, UDB0750344, UDB0751565, UDB0752347, UDB0752478, UDB0752676, UDB0752857, UDB0753016, UDB075512, UDB0756286, UDB0756637, UDB0756810, UDB0758114, UDB0758331, UDB0758396, UDB0758712, UDB079462, UDB080030, UDB085254, UDB087835, UDB094231, UDB094616, UDB095181, UDB097422, UDB098207	
**CLADE C**		
***G. casuarinicola* (7)**		
as *Ganoderma* sp. (5), *G. applanatum* (1), *G. lucidum* (1) from *Tamarindus* sp., decaying wood	HQ689698, KR155026, KF998091, KR155123, KT185664, KX957800, MN784439	China (HI), India
**CLADE D**		
***G. mbrekobenum* (5)**		
as *Ganoderma* sp. (4), *G. lucidum* (1) from *Azadirachta indica*	KJ510533, KM229608, KR154954, KT210090, MF614913	India, Senegal
***G. nasalanense* (2)**		
as *G. australe* (1), *Ganoderma* sp. (1)	KT965502, LC084740	Malaysia, Vietnam
***G. orbiforme* (1)**		
as *G. orbiforme* (1)	MT364488	
***G. mastoporum* (1)**		
as *G. mastoporum* (1)	JN596330	
***G. angustisporum* (1)**		
as *Ganoderma* sp. (1)	JN596322	
***G. ryvardenii* (6)**		
as *Ganoderma* sp. (3), *G. ryvardenii* (2), *G. wiiroense* (1) from *Borassus* sp., *Elaeis guineensis*, decayed wood	JN105700, KY963354, MK681871, MK681872, MN784436, MN809325	Cameroon, India

***G. boninense* (8)**		
as *G. boninense* (6), *G. miniatocinctum*. (1), *G. zonatum* (1) from *Elaeis guineensis*	KM271998, KR093028, KR093029, MN490049, MN490050, MN490051, MN490053, MN490056	Malaysia
**CLADE E**		
***G.* aff. *gibbosum* (5)**		
as *Ganoderma* sp. (3), *G. australe* (1), *G. ryvardenii* (1) from *Cocos nucifera*, *Areca catechu*	KJ792748, KM229660, KM229661, MK681873, MK681874	India
***G.. gibbosum* (5)**		
as *G. australe* (2), *G. gibbosum* (2), *G. applanatum* (1)	KY203856, KY950512, MH101642, MH101643, MN258633	China (JX)
***G. ellipsoideum* (8)**		
as *G. gibbosum* (3), *G. tornatum* (2), *Ganoderma* sp. (2), *G. australe* (1) from *Elaeis guineensis*	JN596333, KM220585, KX421866, MF680426, MG231524, MK268932, MK404343, MN490057	China, Malaysia
***Ganoderma* sp. E6 (2)**		
as *G. australe* (1), uncultured soil fungus (1)	KX831667, MK268929	China
***G. australe* (4)**		
as uncultured soil fungus (2), *G. australe* (1), uncultured *Ganoderma* (1)	KU712532, MT076093, UDB0766717, UDB0766748	Argentina, Australia, India
***Ganoderma* sp. E7 (1)**		
as *G. applanatum* cplx (1)	AF255098	USA (NC)
***G.* aff. *adspersum* (2)**		
as *G. applanatum* (2) from eudicots	KR673486, KR673681	South Korea
***G. adspersum* (2)**		
as *G. adspersum* (1), *Ganoderma* sp. (1)	MG835862, MN412653	China

Abbreviations for geographic regions in USA and China follow the two-character ISO country codes, i.e., https://www.iso.org/obp/ui/#iso:code:3166:US and https://www.iso.org/obp/ui/#iso:code:3166:CN, respectively.

Supplementary Table S5: Information about the 354 environmental samples analyzed and their identity on the basis of the outcome of the present study. Presentation of data follows the format adopted in Supplementary Table S2.

**Clade A**	
**Cluster A.1.**	
***G. tsugae* (3)**	
as uncultured *Ganoderma* (3)<br>from tree and ground	
KU712531, KU712533, KU712534	India
***G. oregonense* (1)**	
as uncultured soil fungi (1)	
UDB0287378	Estonia
***G.* aff. *carnosum* (2)**	
as uncultured soil fungus (2)	
UDB0609440, UDB0632514	Estonia
***G. lucidum* (28)**	
as uncultured soil fungus (28)	
JQ666509, KJ411559, UDB0109711, UDB0111653, UDB0118812, UDB0127747, UDB0245628, UDB0246137, UDB0255944, UDB0280598, UDB0282139, UDB0290411, UDB0383764, UDB0394723, UDB0438898, UDB0498134, UDB0502758, UDB0539428, UDB0549396, UDB0552851, UDB0562707, UDB0562842, UDB0591478, UDB0609431, UDB0713002, UDB0735359, UDB0735496, UDB0226767	China, Estonia, India
**Cluster A.2.**	
***G. sichuanense* (1)**	
as uncultured soil fungus (1)	
UDB0757071	India
***Ganoderma* sp. A3 (1)**	
as uncultured *Ganoderma* (1)<br>from ectomycorrhizal root tip	
GQ268631	Malaysia
***G. resinaceum* (2)**	
as Polyporales sp. (2)<br>from respiratory samples of humans	
JQ312182, JQ312204	India
***G. sessile* (1)**	
as uncultured root-associated fungus (1)<br>from roots of *Yucca glauca*	
FJ362324	USA (NM)
**Cluster A.3.**	
***G. philippii* (1)**	
as uncultured fungus (1)<br>from Acacia hybrid wood	
MF942546	Vietnam
***G. ravenelii* (1)**	
as uncultured fungus (1)<br>from pine tree	
KM103994	USA

***G. destructans* (1)**		
as uncultured soil fungus (1)		
singleton (1)	UDB0765706	Cameroon
***G. multipileum* (1)**<br>as uncultured *Ganoderma* (1)<br>from tree and ground		
KU712535		India
***G. tropicum* (1)**		
as uncultured soil fungus (1)		
UDB033739		Laos
**CLADE B**		
***G. applanatum* (268)**<br>as uncultured soil fungus (238), Fungi (15), uncultured *Ganoderma* (15), uncultured fungus (8)<br>from *Alnus glutinosa*, *Berberis thunbergii*, *Corylus avellana*, *Crataegus punctata*, *Eucalyptus gunnii*, *Fagus sylvatica*, *Ficus lyrata*, *Gleditsia triacanthos*, *Phoenix canariensis*, *Pinus tabuliformis*, *Populus sieboldii*, *Quercus robur*, *Salix alba* x *Salix fragil*, *Sambucus pubescens*, *Thuja occidentalis*, *Tilia cordata*, *Tradescantia zanonia*, plant leaves, roots, soil		
DQ421100, DQ421109, DQ421110, DQ421242, EU554851, EU554859, EU554863, FJ197949, FJ626936, FR750674, GU083147, HQ021834, HQ021835, HQ021836, HQ021975, HQ022119, HQ022243, JQ666446, JQ666457, JQ666465, JQ666495, JQ666507, JQ666577, JQ666595, JQ666726, JX675211, JX675216, JX898575, KC785577, KF800100, KT334783, KT334785, KT334788, KT334790, LC096119, MT236432, UDB0100231, UDB0101232, UDB0103060, UDB0119647, UDB0121485, UDB0122574, UDB0124542, UDB0126096, UDB0130744, UDB0132569, UDB0133733, UDB0134557, UDB0135708, UDB0139875, UDB0142408, UDB0149952, UDB0151129, UDB0152824, UDB0157946, UDB0161689, UDB0162233, UDB0167266, UDB0167717, UDB0170440, UDB0179882, UDB0181758, UDB0184967, UDB0186902, UDB0187261, UDB0189759, UDB0201672, UDB0203037, UDB0214566, UDB0215659, UDB0216981, UDB0219498, UDB0222995, UDB0224112, UDB0225875, UDB0228887, UDB0232284, UDB0240104, UDB0241972, UDB0246103, UDB0247307, UDB0253438, UDB0257893, UDB0261000, UDB0261967, UDB0265157, UDB0266885, UDB0267759, UDB0268901, UDB0275826, UDB0276847, UDB0280808, UDB0287464, UDB0295117, UDB0300725, UDB0309683, UDB0310323, UDB0311723, UDB0322707, UDB0324687, UDB0328840, UDB0328942, UDB0331720, UDB0335181, UDB0338913, UDB0343801, UDB0351962, UDB0352927, UDB0359085, UDB0374592, UDB0374866, UDB0376096, UDB0376950, UDB0380819, UDB0388764, UDB0395610, UDB0400307, UDB040056, UDB040056, UDB0401075, UDB0405032, UDB0407497, UDB0408981, UDB041383, UDB041383, UDB0418862, UDB0420804, UDB0421269, UDB0422666, UDB0422845, UDB042325, UDB0423702, UDB0428244, UDB0429138, UDB0430744, UDB0435574, UDB0437183, UDB0438491, UDB0440440, UDB0443642, UDB0446806, UDB0454763, UDB0455904, UDB0456406, UDB0456671, UDB0456937, UDB0461993, UDB0463272, UDB0465624, UDB0466209, UDB0467477, UDB0469923, UDB0481250, UDB048343, UDB0484438, UDB0489241, UDB0490998, UDB0492431, UDB0492497, UDB0493384, UDB0497080, UDB0501364, UDB0502251, UDB050390, UDB0504358, UDB0504458, UDB0510029, UDB0512395, UDB0512540, UDB0514622, UDB0515632, UDB0518466, UDB0532721, UDB0536161, UDB0541946, UDB0544560, UDB0556942, UDB0557948, UDB0566431, UDB0576874, UDB0578535, UDB0578737, UDB0580293, UDB0580479, UDB0583647, UDB0584468, UDB0608795, UDB061192, UDB0613546, UDB0613654, UDB0615105, UDB0616935, UDB0622788, UDB0623034, UDB0625825, UDB0626374, UDB0630541, UDB0635185, UDB063943, UDB0639460, UDB064483, UDB0647100, UDB0648395, UDB0650310, UDB0652930, UDB0654560, UDB0655465, UDB0658413, UDB0659247, UDB0661494, UDB0668753, UDB067029, UDB0670319, UDB0670667, UDB0679640, UDB0680005, UDB0680531, UDB0680950, UDB0682224, UDB0686694, UDB0688255, UDB0690903, UDB069370, UDB0694220, UDB0699166, UDB070645, UDB0706520, UDB0710049, UDB0715464, UDB0729284, UDB074240, UDB0746367, UDB0746719, UDB0747059, UDB0747170, UDB074757, UDB0748354, UDB0749040, UDB0749357, UDB0749672, UDB0750344, UDB0751565, UDB0752347, UDB0752478, UDB0752676, UDB0752857, UDB0753016, UDB075512, UDB0756286, UDB0756506, UDB0756637, UDB0756810, UDB0758107, UDB0758114, UDB0758331, UDB0758396, UDB0758712, UDB079462, UDB080030, UDB085254, UDB085994, UDB087835, UDB094231, UDB094616, UDB095181, UDB097422, UDB098207, UDB099583		Antarctica, Austria, China, Estonia, Germany, Japan, Latvia, Lithuania, Poland, USA (AK, MN, MO, MS, NH, NY, OR)
**CLADE C**		
***G. enigmaticum* (1)**		
as uncultured soil fungus (1)		

UDB0767618	Ivory Coast
***G. casuarinicola* (3)**<br>as uncultured fungus (2), uncultured *Ganoderma* (1)<br>from tree and ground, air	
KU712536, KY978282, KY978295	India
**CLADE D**	
***G. nasalanense* (2)**	
as uncultured soil fungus (2)	
DB0760488, UDB0767030	India, Malaysia
***G. cupreum* (1)**	
as uncultured fungus (1)<br>from *Anthosthema aubryanum*	
AB828214	Gabon
***G. subfornicatum* (1)**	
as uncultured fungus (1)<br>from soil	
KJ411557	India
***G. mastoporum* (6)**<br>as uncultured soil fungus (6)	
UDB039638, UDB0767350, UDB0767363, UDB0767369, UDB0767381, UDB0767392	Indonesia
**CLADE E**	
***G. williamsianum* (1)**	
as uncultured fungus (1)<br>from Acacia hybrid wood	
MF942545	Vietnam
***G. ellipsoideum* (6)**<br>as uncultured soil fungus (5), uncultured Ganodermataceae (1)<br>from *Coelogyne viscosa* roots	
KF574247, UDB0755339, UDB0763169, UDB0763546, UDB0769802, UDB0777839	China, India, Papua New Guinea
***Ganoderma* sp. E3 (1)**<br>as uncultured soil fungus (1)	
UDB0755590	Australia
***G. podocarpense* (1)**<br>as uncultured soil fungus (1)	
UDB0759513	Panama
***Ganoderma* sp. E5 (1)**<br>as uncultured soil fungus (1)	
UDB0768776	Argentina
***Ganoderma* sp. E6 (2)**<br>as uncultured *Ganoderma* (1), uncultured soil fungus (1)<br>from bark of coniferous tree, soil	
KX831667, UDB0755383	India, Papua New Guinea
***G. australe* (12)**<br>as uncultured soil fungus (10), fungal sp. (1), uncultured *Ganoderma* (1)<br>from leaf of *Brachiaria* sp., soil	

KU712532, MH242613, MT076093, UDB0759941, UDB0762229, UDB0766717, UDB0766747, UDB0766748, UDB0767741, UDB0767760, UDB0767764, UDB0768220, UDB0768715	Argentina. Australia, India, New Zealand
***Ganoderma* sp. E7 (1)**<br>as uncultured soil fungus (1)	
KF800469	USA (MO)
***G. adspersum* (2)**<br>as uncultured *Ganoderma* (1), uncultured fungus (1)<br>from air sample, human stool	
FJ820612, KP974213	Germany
***G.* aff. *adspersum* (1)**<br>as uncultured soil fungus (1)	
UDB0765962	China

Abbreviations for geographic regions in USA follow the two-character ISO country code, i.e., https://www.iso.org/obp/ui/#iso:code:3166:US.

Supplementary Table S6: *Ganoderma* entries (3908) analyzed: initial identification (as labelled in GenBank/ENA/DDBJ and UNITE databases), accession number, corresponding UNITE DOI and taxon name on the basis of the outcome of this study. Entries appear in the order that is adopted in Table 1.

a/a	Initial identification	Accession number	UNITE DOI	Taxon name
1	*Ganoderma* sp.	MK764268	SH2766734.08FU	*G. shanxiense*
2	*Ganoderma* sp.	MK764269	SH2766735.08FU	*G. shanxiense*
3	*G. tsugae*	DQ206985		*G. tsugae*
4	*G. tsugae*	FJ655478	SH1723037.08FU	*G. tsugae*
5	*G. tsugae*	FJ655479	SH1723037.08FU	*G. tsugae*
6	*G. tsugae*	FJ655480	SH1723037.08FU	*G. tsugae*
7	*G. tsugae*	JQ520215	SH1723037.08FU	*G. tsugae*
8	*G. tsugae*	KF605617	SH1723037.08FU	*G. tsugae*
9	*G. tsugae*	KF605618	SH1723037.08FU	*G. tsugae*
10	*G. tsugae*	KJ140748	SH1723037.08FU	*G. tsugae*
11	*G. tsugae*	KJ143920	SH1723037.08FU	*G. tsugae*
12	*G. tsugae*	MG231528		*G. tsugae*
13	*G. tsugae*	MG654321	SH1723037.08FU	*G. tsugae*
14	*G. tsugae*	MG654322		*G. tsugae*
15	*G. tsugae*	MG654323	SH1723037.08FU	*G. tsugae*
16	*G. tsugae*	MG654324		*G. tsugae*
17	*G. tsugae*	MG654325	SH1723037.08FU	*G. tsugae*
18	*G. tsugae*	MG654326	SH1723037.08FU	*G. tsugae*
19	*G. tsugae*	MG654327		*G. tsugae*
20	*G. tsugae*	MG654328		*G. tsugae*
21	*G. tsugae*	MG654329		*G. tsugae*
22	*G. tsugae*	MG654330		*G. tsugae*
23	*G. tsugae*	MG654331		*G. tsugae*
24	*G. tsugae*	MG654332		*G. tsugae*
25	*G. tsugae*	MG654333		*G. tsugae*
26	*G. tsugae*	MG654334	SH1723037.08FU	*G. tsugae*
27	*G. tsugae*	MG654335		*G. tsugae*
28	*G. tsugae*	MG654336		*G. tsugae*
29	*G. tsugae*	MG654337		*G. tsugae*
30	*G. tsugae*	MG654338		*G. tsugae*
31	*G. tsugae*	MG654339	SH1723037.08FU	*G. tsugae*
32	*G. tsugae*	MG654340		*G. tsugae*
33	*G. tsugae*	MG654341		*G. tsugae*
34	*G. tsugae*	MG654343		*G. tsugae*
35	*G. tsugae*	MG654344		*G. tsugae*
36	*G. tsugae*	MG654345		*G. tsugae*
37	*G. tsugae*	MG654346		*G. tsugae*
38	*G. tsugae*	MG654347		*G. tsugae*
39	*G. tsugae*	MG654348		*G. tsugae*

40	*G. tsugae*	MG654349		*G. tsugae*
41	*G. tsugae*	MG654350	SH1723037.08FU	*G. tsugae*
42	*G. tsugae*	MG910999		*G. tsugae*
43	*G. tsugae*	MH277960	SH1723037.08FU	*G. tsugae*
44	*G. tsugae*	MH277961		*G. tsugae*
45	*G. tsugae*	MT357080		*G. tsugae*
46	*G. tsugae*	X78735/ X78756 (~JQ520218)		*G. tsugae*
47	*G. tsugae*	Z37054/Z37079		*G. tsugae*
48	*G. tsugae*	Z37055/Z37080		*G. tsugae*
49	*G. lucidum*	DQ425002	SH1723037.08FU	*G. tsugae*
50	*G. lucidum*	DQ425003	SH1723037.08FU	*G. tsugae*
51	*G. lucidum*	DQ425010	SH1723037.08FU	*G. tsugae*
52	*Ganoderma* sp.	KC514828	SH1723037.08FU	*G. tsugae*
53	uncultured *Ganoderma*	KU712531		*G. tsugae*
54	uncultured *Ganoderma*	KU712533		*G. tsugae*
55	uncultured *Ganoderma*	KU712534		*G. tsugae*
56	*Polyporus tsugae*	MH856317	SH1723037.08FU	*G. tsugae*
57	*G. ahmadii*	Z37047/Z37098		*G. tsugae*
58	*G. valesiacum*	Z37056/Z37081		*G. tsugae*
59	*G. carnosum*	Z37057/Z37082		*G. tsugae*
60	*G. oregonense*	JQ520195		*G. oregonense*
61	*G. oregonense*	JQ781875	SH1723037.08FU	*G. oregonense*
62	*G. oregonense*	JQ781876	SH1723037.08FU	*G. oregonense*
63	*G. oregonense*	MG654189	SH1723037.08FU	*G. oregonense*
64	*G. oregonense*	MG654190		*G. oregonense*
65	*G. oregonense*	MG654191		*G. oregonense*
66	*G. oregonense*	MG654192		*G. oregonense*
67	*G. oregonense*	MG654193	SH1723037.08FU	*G. oregonense*
68	*G. oregonense*	MG654194		*G. oregonense*
69	*G. oregonense*	MG654195	SH1723037.08FU	*G. oregonense*
70	*G. oregonense*	MH277958	SH1723037.08FU	*G. oregonense*
71	*G. oregonense*	MH277959	SH1723037.08FU	*G. oregonense*
72	*G. oregonense*	MT196417		*G. oregonense*
73	*G. oregonense*	Z37060/Z37100		*G. oregonense*
74	*G. oregonense*	Z37061/Z37101		*G. oregonense*
75	*G. carnosum*	EU486458	SH1723037.08FU	*G. oregonense*
76	*G. carnosum*	JN222419	SH1723192.08FU	*G. oregonense*
77	*G. lucidum*	JQ520185		*G. oregonense*
78	*G. tsugae*	JQ520216	SH1723037.08FU	*G. oregonense*
79	*G. carnosum*	KF605619	SH1723037.08FU	*G. oregonense*
80	*G. carnosum*	KF605620		*G. oregonense*
81	*G. carnosum*	KF605621	SH1723037.08FU	*G. oregonense*
82	*G. tsugae*	KJ143919		*G. oregonense*
83	*G. tsugae*	KJ146707	SH1723037.08FU	*G. oregonense*
84	*G. tsugae*	MG654342	SH1723037.08FU	*G. oregonense*

85	uncultured soil fungus	UDB0287378	SH1723037.08FU	*G. oregonense*
86	*G. lucidum*	Z37052/Z37076		*G. oregonense*
87	*G. carnosum*	GU731557		*G. carnosum*
88	*G. carnosum*	KF605622		*G. carnosum*
89	*G. carnosum*	KF605623	SH1723037.08FU	*G. carnosum*
90	*G. carnosum*	KF605624	SH1723037.08FU	*G. carnosum*
91	*G. carnosum*	KF605625	SH1723037.08FU	*G. carnosum*
92	*G. carnosum*	KF605626	SH1723037.08FU	*G. carnosum*
93	*G. carnosum*	KU572492		*G. carnosum*
94	*G. carnosum*	KU572493		*G. carnosum*
95	*G. carnosum*	KU572494	SH1723037.08FU	*G. carnosum*
96	*G. carnosum*	MG706216	SH1723037.08FU	*G. carnosum*
97	*G. carnosum*	MG706217		*G. carnosum*
98	*G. carnosum*	MG706218	SH1723037.08FU	*G. carnosum*
99	*G. carnosum*	MG706219	SH1723037.08FU	*G. carnosum*
100	*G. carnosum*	MG706220	SH1723037.08FU	*G. carnosum*
101	*G. carnosum*	MK415244		*G. carnosum*
102	*G. carnosum*	MK415261		*G. carnosum*
103	*G. carnosum*	MK415266		*G. carnosum*
104	*G. carnosum*	MK415267		*G. carnosum*
105	*G. carnosum*	MK415276		*G. carnosum*
106	*G. carnosum*	MK415287		*G. carnosum*
107	*G. carnosum*	MK415312		*G. carnosum*
108	*G. carnosum*	MK415314		*G. carnosum*
109	*G. lucidum*	JN588574	SH1723037.08FU	*G. carnosum*
110	*G. lucidum*	JN588578	SH1723037.08FU	*G. carnosum*
111	*G. lucidum*	JQ627587	SH1723037.08FU	*G. carnosum*
112	*G. lucidum*	KC311369	SH1723037.08FU	*G. carnosum*
113	*G. lucidum*	AF094511/AF044490		*G.* aff. *carnosum*
114	*G. carnosum*	AY884175		*G.* aff. *carnosum*
115	uncultured soil fungus	UDB0609440	SH1723037.08FU	*G.* aff. *carnosum*
116	uncultured soil fungus	UDB0632514	SH1723037.08FU	*G.* aff. *carnosum*
117	*G. lucidum*	AM269773	SH1723037.08FU	*G. lucidum*
118	*G. lucidum*	AM906058		*G. lucidum*
119	*G. lucidum*	AY884176	SH1723037.08FU	*G. lucidum*
120	*G. lucidum*	DQ424972		*G. lucidum*
121	*G. lucidum*	EU498090	SH1723037.08FU	*G. lucidum*
122	*G. lucidum*	FJ216424	SH1723037.08FU	*G. lucidum*
123	*G. lucidum*	FJ463903		*G. lucidum*
124	*G. lucidum*	FJ463911		*G. lucidum*
125	*G. lucidum*	FJ463914		*G. lucidum*
126	*G. lucidum*	FJ463916		*G. lucidum*
127	*G. lucidum*	FJ463918	SH1723188.08FU	*G. lucidum*
128	*G. lucidum*	FJ463922		*G. lucidum*
129	*G. lucidum*	FJ463927		*G. lucidum*

130	*G. lucidum*	FJ463930	SH1723037.08FU	*G. lucidum*
131	*G. lucidum*	GU731558	SH1723037.08FU	*G. lucidum*
132	*G. lucidum*	JN176895		*G. lucidum*
133	*G. lucidum*	JN176897	SH1723037.08FU	*G. lucidum*
134	*G. lucidum*	JN176899	SH1723037.08FU	*G. lucidum*
135	*G. lucidum*	JN222422	SH1723037.08FU	*G. lucidum*
136	*G. lucidum*	JN588572		*G. lucidum*
137	*G. lucidum*	JN588573	SH1723037.08FU	*G. lucidum*
138	*G. lucidum*	JN588575		*G. lucidum*
139	*G. lucidum*	JN588576	SH1723037.08FU	*G. lucidum*
140	*G. lucidum*	JN588577	SH1723037.08FU	*G. lucidum*
141	*G. lucidum*	JQ520186	SH1723037.08FU	*G. lucidum*
142	*G. lucidum*	JQ781851	SH1723037.08FU	*G. lucidum*
143	*G. lucidum*	JQ781852		*G. lucidum*
144	*G. lucidum*	JQ781853	SH1723037.08FU	*G. lucidum*
145	*G. lucidum*	JX082330		*G. lucidum*
146	*G. lucidum*	JX162769	SH1723194.08FU	*G. lucidum*
147	*G. lucidum*	KC222321	SH1723037.08FU	*G. lucidum*
148	*G. lucidum*	KC222322	SH1723037.08FU	*G. lucidum*
149	*G. lucidum*	KF605614	SH1723037.08FU	*G. lucidum*
150	*G. lucidum*	KF605615		*G. lucidum*
151	*G. lucidum*	KF605616	SH1723037.08FU	*G. lucidum*
152	*G. lucidum*	KF963254	SH1723192.08FU	*G. lucidum*
153	*G. lucidum*	KJ143909		*G. lucidum*
154	*G. lucidum*	KJ143910	SH1723037.08FU	*G. lucidum*
155	*G. lucidum*	KJ143911	SH1723037.08FU	*G. lucidum*
156	*G. lucidum*	KJ143912	SH1723037.08FU	*G. lucidum*
157	*G. lucidum*	KJ509596		*G. lucidum*
158	*G. lucidum*	KJ857252	SH1723037.08FU	*G. lucidum*
159	*G. lucidum*	KP941444		*G. lucidum*
160	*G. lucidum*	KP941445	SH1723037.08FU	*G. lucidum*
161	*G. lucidum*	KP941446		*G. lucidum*
162	*G. lucidum*	KP941448	SH1723037.08FU	*G. lucidum*
163	*G. lucidum*	KT318592	SH1723037.08FU	*G. lucidum*
164	*G. lucidum*	KT318593	SH1723037.08FU	*G. lucidum*
165	*G. lucidum*	KT318594	SH1723037.08FU	*G. lucidum*
166	*G. lucidum*	KT318595	SH1723037.08FU	*G. lucidum*
167	*G. lucidum*	KT343297	SH1723037.08FU	*G. lucidum*
168	*G. lucidum*	KT343301	SH1723037.08FU	*G. lucidum*
169	*G. lucidum*	KT343304	SH1723037.08FU	*G. lucidum*
170	*G. lucidum*	KT343314	SH1723037.08FU	*G. lucidum*
171	*G. lucidum*	KT805317	SH1723037.08FU	*G. lucidum*
172	*G. lucidum*	KU310900	SH1723037.08FU	*G. lucidum*
173	*G. lucidum*	KX765192	SH1723037.08FU	*G. lucidum*
174	*G. lucidum*	MF419230	SH1723037.08FU	*G. lucidum*

175	*G. lucidum*	MF419231	SH1723037.08FU	*G. lucidum*
176	*G. lucidum*	MF581079		*G. lucidum*
177	*G. lucidum*	MG279161	SH1723037.08FU	*G. lucidum*
178	*G. lucidum*	MG279162	SH1723037.08FU	*G. lucidum*
179	*G. lucidum*	MG279181	SH1723037.08FU	*G. lucidum*
180	*G. lucidum*	MG279182	SH1723037.08FU	*G. lucidum*
181	*G. lucidum*	MG654066	SH1723037.08FU	*G. lucidum*
182	*G. lucidum*	MG654067		*G. lucidum*
183	*G. lucidum*	MG654068	SH1723037.08FU	*G. lucidum*
184	*G. lucidum*	MG654069	SH1723037.08FU	*G. lucidum*
185	*G. lucidum*	MG654070		*G. lucidum*
186	*G. lucidum*	MG654071		*G. lucidum*
187	*G. lucidum*	MG654072		*G. lucidum*
188	*G. lucidum*	MG654073		*G. lucidum*
189	*G. lucidum*	MG706223	SH1723037.08FU	*G. lucidum*
190	*G. lucidum*	MG706224	SH1723037.08FU	*G. lucidum*
191	*G. lucidum*	MG706225	SH1723037.08FU	*G. lucidum*
192	*G. lucidum*	MG706226	SH1723037.08FU	*G. lucidum*
193	*G. lucidum*	MG706227	SH1723037.08FU	*G. lucidum*
194	*G. lucidum*	MG706228		*G. lucidum*
195	*G. lucidum*	MG706229	SH1723037.08FU	*G. lucidum*
196	*G. lucidum*	MG706230	SH1723037.08FU	*G. lucidum*
197	*G. lucidum*	MG706231	SH1723037.08FU	*G. lucidum*
198	*G. lucidum*	MG835865	SH1723037.08FU	*G. lucidum*
199	*G. lucidum*	MG911000	SH1723037.08FU	*G. lucidum*
200	*G. lucidum*	MH160071	SH1723037.08FU	*G. lucidum*
201	*G. lucidum*	MH861340	SH1723037.08FU	*G. lucidum*
202	*G. lucidum*	MK050595	SH1723037.08FU	*G. lucidum*
203	*G. lucidum*	MK415269		*G. lucidum*
204	*G. lucidum*	MK415270		*G. lucidum*
205	*G. lucidum*	MK415271		*G. lucidum*
206	*G. lucidum*	MK415272		*G. lucidum*
207	*G. lucidum*	MK415285		*G. lucidum*
208	*G. lucidum*	MK415286		*G. lucidum*
209	*G. lucidum*	MK554777		*G. lucidum*
210	*G. lucidum*	MK554779		*G. lucidum*
211	*G. lucidum*	MN396338		*G. lucidum*
212	*G. lucidum*	MN396339		*G. lucidum*
213	*G. lucidum*	MN396340		*G. lucidum*
214	*G. lucidum*	MN396341		*G. lucidum*
215	*G. lucidum*	MN401402		*G. lucidum*
216	*G. lucidum*	MN401403		*G. lucidum*
217	*G. lucidum*	MN401404	SH1723037.08FU	*G. lucidum*
218	*G. lucidum*	MT225392		*G. lucidum*
219	*G. lucidum*	MT510202		*G. lucidum*

220	*G. lucidum*	UDB018875	SH1723037.08FU	*G. lucidum*
221	*G. lucidum*	UDB031872	SH1723037.08FU	*G. lucidum*
222	*G. lucidum*	Z37049/Z37099		*G. lucidum*
223	*G. lucidum*	Z37096/Z37073		*G. lucidum*
224	*G. oerstedii*	DQ425011	SH1723037.08FU	*G. lucidum*
225	uncultured soil fungus	JQ666509	SH1723037.08FU	*G. lucidum*
226	*G. tsugae*	JQ781854	SH1723037.08FU	*G. lucidum*
227	uncultured soil fungus	KJ411559	SH1723037.08FU	*G. lucidum*
228	*G. tsugae*	KU863090	SH1723037.08FU	*G. lucidum*
229	*G. tsugae*	KU863091	SH1723037.08FU	*G. lucidum*
230	*G. tsugae*	KU863092	SH1723037.08FU	*G. lucidum*
231	*G. tsugae*	KU863093	SH1723037.08FU	*G. lucidum*
232	*G. tsugae*	KU863094	SH1723037.08FU	*G. lucidum*
233	*G. tsugae*	MG279195	SH1723037.08FU	*G. lucidum*
234	*G. tsugae*	MG279196	SH1723037.08FU	*G. lucidum*
235	*G. tsugae*	MG279197	SH1723037.08FU	*G. lucidum*
236	*G. valesiacum*	MG711807	SH1723037.08FU	*G. lucidum*
237	*G. oregonense*	MH294309		*G. lucidum*
238	*G. tsugae*	MH294321	SH1723037.08FU	*G. lucidum*
239	*Ganoderma* sp.	MK050948	SH1723037.08FU	*G. lucidum*
240	*G. tsugae*	MN396400	SH1723037.08FU	*G. lucidum*
241	uncultured soil fungus	UDB0109711	SH1723037.08FU	*G. lucidum*
242	uncultured soil fungus	UDB0111653	SH1723037.08FU	*G. lucidum*
243	uncultured soil fungus	UDB0118812	SH1723037.08FU	*G. lucidum*
244	uncultured soil fungus	UDB0127747	SH1723037.08FU	*G. lucidum*
245	uncultured soil fungus	UDB0245628	SH1723037.08FU	*G. lucidum*
246	uncultured soil fungus	UDB0246137	SH1723037.08FU	*G. lucidum*
247	uncultured soil fungus	UDB0255944	SH1723037.08FU	*G. lucidum*
248	uncultured soil fungus	UDB0280598	SH1723037.08FU	*G. lucidum*
249	uncultured soil fungus	UDB0282139	SH1723037.08FU	*G. lucidum*
250	uncultured soil fungus	UDB0290411	SH1723037.08FU	*G. lucidum*
251	uncultured soil fungus	UDB0383764	SH1723037.08FU	*G. lucidum*
252	uncultured soil fungus	UDB0394723	SH1723037.08FU	*G. lucidum*
253	uncultured soil fungus	UDB0438898	SH1723037.08FU	*G. lucidum*
254	uncultured soil fungus	UDB0498134	SH1723037.08FU	*G. lucidum*
255	uncultured soil fungus	UDB0502758	SH1723037.08FU	*G. lucidum*
256	uncultured soil fungus	UDB0539428	SH1723037.08FU	*G. lucidum*
257	uncultured soil fungus	UDB0549396	SH1723037.08FU	*G. lucidum*
258	uncultured soil fungus	UDB0552851	SH1723037.08FU	*G. lucidum*
259	uncultured soil fungus	UDB0562707	SH1723037.08FU	*G. lucidum*
260	uncultured soil fungus	UDB0562842	SH1723037.08FU	*G. lucidum*
261	uncultured soil fungus	UDB0591478	SH1723037.08FU	*G. lucidum*
262	uncultured soil fungus	UDB0609431	SH1723192.08FU	*G. lucidum*
263	uncultured soil fungus	UDB0713002	SH1723037.08FU	*G. lucidum*
264	uncultured soil fungus	UDB0735359	SH1723037.08FU	*G. lucidum*

265	uncultured soil fungus	UDB0735496	SH1723037.08FU	*G. lucidum*
266	*G. oerstedii*	X78739/X78760		*G. lucidum*
267	*G. tsugae*	X78748/X78769		*G. lucidum*
268	*G. oerstedii*	Z37058/Z37083		*G. lucidum*
269	uncultured soil fungus	UDB0226767	SH1723037.08FU	*G. lucidum*
270	*G. leucocontextum*	KF011548	SH1723037.08FU	*G. leucocontextum - G. weixiensis*
271	*G. leucocontextum* (*Ganoderma* sp.)	KF372586	SH1723037.08FU	*G. leucocontextum - G. weixiensis*
272	*G. leucocontextum* (*Ganoderma* sp.)	KJ027607	SH1723037.08FU	*G. leucocontextum - G. weixiensis*
273	*G. leucocontextum* (*Ganoderma* sp.)	KJ027608	SH1723037.08FU	*G. leucocontextum - G. weixiensis*
274	*G. leucocontextum* (*Ganoderma* sp.)	KJ027609	SH1723037.08FU	*G. leucocontextum - G. weixiensis*
275	*G. leucocontextum*	KM396271	SH1723037.08FU	*G. leucocontextum - G. weixiensis*
276	*G. leucocontextum*	KM396272	SH1723037.08FU	*G. leucocontextum - G. weixiensis*
277	*G. leucocontextum*	KP676024	SH1723037.08FU	*G. leucocontextum - G. weixiensis*
278	*G. leucocontextum*	KP676025	SH1723037.08FU	*G. leucocontextum - G. weixiensis*
279	*G. leucocontextum*	KU572485	SH1723037.08FU	*G. leucocontextum - G. weixiensis*
280	*G. leucocontextum*	KU863084		*G. leucocontextum - G. weixiensis*
281	*G. leucocontextum*	KU863085		*G. leucocontextum - G. weixiensis*
282	*G. leucocontextum*	KU863086		*G. leucocontextum - G. weixiensis*
283	*G. leucocontextum*	KU863087	SH1723037.08FU	*G. leucocontextum - G. weixiensis*
284	*G. leucocontextum*	KU863088	SH1723037.08FU	*G. leucocontextum - G. weixiensis*
285	*G. leucocontextum*	KX055558	SH1723037.08FU	*G. leucocontextum - G. weixiensis*
286	*G. leucocontextum*	KX055559	SH1723037.08FU	*G. leucocontextum - G. weixiensis*
287	*G. leucocontextum*	MH294311		*G. leucocontextum - G. weixiensis*
288	*G. leucocontextum*	MK713839	SH1723037.08FU	*G. leucocontextum - G. weixiensis*
289	*G. leucocontextum*	MN134012	SH1723037.08FU	*G. leucocontextum - G. weixiensis*
290	*G. leucocontextum*	MN134518		*G. leucocontextum - G. weixiensis*
291	*G. leucocontextum*	MN396317	SH1723037.08FU	*G. leucocontextum - G. weixiensis*
292	*G. leucocontextum*	MN396318	SH1723037.08FU	*G. leucocontextum - G. weixiensis*
293	*G. leucocontextum*	MT007546		*G. leucocontextum - G. weixiensis*
294	*G. weixiensis*	MK302444/NR_166271		*G. leucocontextum - G. weixiensis*
295	*G. weixiensis*	MK302445		*G. leucocontextum - G. weixiensis*
296	*G. carnosum*	AB763348	SH1723037.08FU	*G. leucocontextum - G. weixiensis*

297	*G. lucidum*	KC222320		*G. leucocontextum - G. weixiensis*
298	*G. lucidum*	KC222323	SH1723037.08FU	*G. leucocontextum - G. weixiensis*
299	*G. lucidum*	KX262903	SH1723037.08FU	*G. leucocontextum - G. weixiensis*
300	*Ganoderma* sp.	MG066446	SH2762385.08FU	*G. leucocontextum - G. weixiensis*
301	*Ganoderma* sp.	MG066447	SH2762385.08FU	*G. leucocontextum - G. weixiensis*
302	*Ganoderma* sp.	MH294310		*G. leucocontextum - G. weixiensis*
303	*G. austroafricanum*	KM507324	SH1723307.08FU	*G. austroafricanum*
304	*G.* aff. *austroafricanum*	MH571693	SH1723307.08FU	*G. austroafricanum*
305	*G. hoehnelianum*	JN383980	SH1723087.08FU	*G. hoehnelianum*
306	*G. hoehnelianum*	JX195202	SH1723087.08FU	*G. hoehnelianum*
307	*G. hoehnelianum*	JX195203	SH1723087.08FU	*G. hoehnelianum*
308	*G. hoehnelianum*	KU219988	SH1723087.08FU	*G. hoehnelianum*
309	*G. hoehnelianum*	KU219989		*G. hoehnelianum*
310	*G. hoehnelianum*	MG279160	SH1723087.08FU	*G. hoehnelianum*
311	*G. hoehnelianum*	MG279178	SH1723087.08FU	*G. hoehnelianum*
312	*G. hoehnelianum*	MH106881	SH1723087.08FU	*G. hoehnelianum*
313	*G. hoehnelianum*	MN396316	SH1723087.08FU	*G. hoehnelianum*
314	*Ganoderma* sp.	MK531812		*G. hoehnelianum*
315	*Ganoderma* sp.	MK554781		*G. hoehnelianum*
316	*Ganoderma* sp.	MK554787		*G. hoehnelianum*
317	*Ganoderma* sp.	MK554788		*G. hoehnelianum*
318	*Ganoderma* sp.	MK554789		*G. hoehnelianum*
319	*G. weberianum*	JQ520219	SH1723064.08FU	*G. weberianum*
320	*G. weberianum*	MH855780	SH1723064.08FU	*G. weberianum*
321	*G. weberianum*	MH864975	SH1723064.08FU	*G. weberianum*
322	*G. weberianum*	MK603804 (=MH855780, X78734/X78755, JQ520219)		*G. weberianum*
323	*G. weberianum*	MK603805		*G. weberianum*
324	*G. weberianum*	MN622782		*G. weberianum*
325	*G. weberianum*	X78734/X78755		*G. weberianum*
326	*G. weberianum*	Z37064/Z37086		*G. weberianum*
327	*G. sichuanense*	KX055552	SH1723064.08FU	*G. weberianum*
328	*Ganoderma* sp.	MK554790		*G. weberianum*
329	*G. microsporum*	X78751/X78772		*G. weberianum*
330	*G. weberianum*	HQ267232	SH2752374.08FU	*G. weberianum*
331	*G. sichuanense*	JQ781877	SH1723064.08FU	*G. sichuanense*
332	*G. sichuanense*	JQ781878	SH1723064.08FU	*G. sichuanense*
333	*G. sichuanense*	MN523250	SH1723064.08FU	*G. sichuanense*
334	*G. sichuanense*	MN523251	SH1723064.08FU	*G. sichuanense*
335	*G. sichuanense*	MN523252	SH1723064.08FU	*G. sichuanense*
336	*G. sichuanense*	MN523253	SH1723064.08FU	*G. sichuanense*
337	*G. sichuanense*	MN523254	SH1723264.08FU	*G. sichuanense*

338	*G. sichuanense*	MN523255	SH1723064.08FU	*G. sichuanense*
339	*G. sichuanense*	MN523256	SH1723064.08FU	*G. sichuanense*
340	*G. weberianum*	AY569451	SH1723064.08FU	*G. sichuanense*
341	*G. lucidum*	DQ424975		*G. sichuanense*
342	*G. lucidum*	DQ424976		*G. sichuanense*
343	*G. tenue*	DQ424977	SH1723064.08FU	*G. sichuanense*
344	*G. tenue*	DQ424978	SH1723064.08FU	*G. sichuanense*
345	*G. weberianum*	EU239393/4		*G. sichuanense*
346	*G. weberianum*	JF915411	SH1723264.08FU	*G. sichuanense*
347	*Ganoderma* sp.	KJ654377	SH1723064.08FU	*G. sichuanense*
348	*G. weberianum*	MN523258	SH1723264.08FU	*G. sichuanense*
349	uncultured soil fungus	UDB0757071	SH1723064.08FU	*G. sichuanense*
350	*G. carocalcareus*	EU089969	SH1723155.08FU	*G. carocalcareus*
351	*G. carocalcareus*	EU089970	SH1723155.08FU	*G. carocalcareus*
352	*G. weberianum*	JN105703	SH1723064.08FU	*G. carocalcareus*
353	*G. weberianum*	JN105704	SH1723064.08FU	*G. carocalcareus*
354	*G. weberianum*	JN105705	SH1723064.08FU	*G. carocalcareus*
355	*Ganoderma* sp.	MK554768		*G. carocalcareus*
356	*Ganoderma* sp.	MK554773		*G. carocalcareus*
357	*Ganoderma* sp.	MK554774		*G. carocalcareus*
358	*Ganoderma* sp.	MK554776		*G. carocalcareus*
359	*Ganoderma* sp.	MK554780		*G. carocalcareus*
360	*Ganoderma* sp.	MK554784		*G. carocalcareus*
361	*Ganoderma* sp.	MK554785		*G. carocalcareus*
362	*Ganoderma* sp.	MK603806		*G. carocalcareus*
363	*G. weberianum*	FJ491967		*Ganoderma* sp. A1
364	*G. weberianum*	FJ491968		*Ganoderma* sp. A1
365	*G. weberianum*	FJ491969	SH1740420.08FU	*Ganoderma* sp. A1
366	*G. weberianum*	FJ491970		*Ganoderma* sp. A1
367	*G. weberianum*	FJ491971		*Ganoderma* sp. A1
368	*G. weberianum*	FJ491972		*Ganoderma* sp. A1
369	*G. weberianum*	FJ491973		*Ganoderma* sp. A1
370	*G. weberianum*	FJ491974		*Ganoderma* sp. A1
371	*G. weberianum*	FJ491975		*Ganoderma* sp. A1
372	*G. weberianum*	FJ491976		*Ganoderma* sp. A1
373	*G. weberianum*	FJ491977	SH1740420.08FU	*Ganoderma* sp. A1
374	*G. weberianum*	FJ491978	SH1740445.08FU	*Ganoderma* sp. A1
375	*G. weberianum*	FJ491979	SH1740420.08FU	*Ganoderma* sp. A1
376	*G. weberianum*	FJ491986	SH1740420.08FU	*Ganoderma* sp. A1
377	*G. weberianum*	FJ491987	SH1740420.08FU	*Ganoderma* sp. A1
378	*G. weberianum*	FJ491988	SH1740420.08FU	*Ganoderma* sp. A1
379	*G. weberianum*	FJ655481	SH1740444.08FU	*Ganoderma* sp. A1
380	*G. weberianum*	GU726934	SH1723064.08FU	*G.* aff. *weberianum*
381	*G. weberianum*	GU726935	SH1723064.08FU	*G.* aff. *weberianum*
382	*G.* cf. *weberianum*	KC222319	SH1723064.08FU	*G.* aff. *weberianum*

383	*G. subamboinense*	KU569546	SH1723064.08FU	*G.* aff. *weberianum*
384	*Ganoderma* sp.	MK571161	SH1723064.08FU	*G.* aff. *weberianum*
385	*G. mexicanum*	MK531811		*G. mexicanum*
386	*G. mexicanum*	MK531815		*G. mexicanum*
387	*G. mexicanum*	MK531818		*G. mexicanum*
388	*G. mexicanum*	MK531819		*G. mexicanum*
389	*G. mexicanum*	MK531820		*G. mexicanum*
390	*G. mexicanum*	MK531823		*G. mexicanum*
391	*G. tuberculosum*	AF169997/8 & AH008107		*G. mexicanum*
392	*G.* aff. *sessiliforme*	AF169999/AF170000 & AH008108		*G. mexicanum*
393	*G. subamboines*	DQ425005	SH1723072.08FU	*G. mexicanum*
394	*G. subamboines*	DQ425006	SH1723072.08FU	*G. mexicanum*
395	*G. subamboinense* var. *laevisporum*	JQ520205	SH1723072.08FU	*G. mexicanum*
396	*G. subamboinense* var. *laevisporum*	MG654370	SH1723072.08FU	*G. mexicanum*
397	*G. subamboinense* var. *laevisporum (G.* cf. *weberianum)*	MG654373	SH1723072.08FU	*G. mexicanum*
398	*G. weberianum*	MK554771		*G. mexicanum*
399	*G. subamboinense* var. *laevisporum*	MT232638		*G. mexicanum*
400	*G. subamboinense* var. *laevisporum*	X78736/X78757		*G. mexicanum*
401	*G. weberianum*	MT232642		*G. mexicanum*
402	*G. parvulum*	MK531132		*G. parvulum*
403	*G. parvulum*	MK531813		*G. parvulum*
404	*G. parvulum*	MK531814		*G. parvulum*
405	*G. parvulum*	MK531817		*G. parvulum*
406	*G. parvulum*	MK531821		*G. parvulum*
407	*G. parvulum*	MK554767		*G. parvulum*
408	*G. parvulum*	MK554769		*G. parvulum*
409	*G. parvulum*	MK554770		*G. parvulum*
410	*G. parvulum*	MK554782		*G. parvulum*
411	*G. parvulum*	MK554783		*G. parvulum*
412	*G. weberianum*	GU731560	SH1723072.08FU	*G. parvulum*
413	*Ganoderma* sp.	HM800731	SH1740422.08FU	*G. parvulum*
414	*G. weberianum*	JN637827	SH1740422.08FU	*G. parvulum*
415	*G. stipitatum*	KC884264	SH1740422.08FU	*G. parvulum*
416	*G. weberianum*	KU214242	SH1723072.08FU	*G. parvulum*
417	*Ganoderma* sp.	LT726730	SH1723072.08FU	*G. parvulum*
418	*Ganoderma* sp.	LT726731	SH1723072.08FU	*G. parvulum*
419	*G. subamboinense* var. *laevisporum*	MG654371		*G. parvulum*
420	*G. subamboinense* var. *laevisporum (G.* cf. *weberianum)*	MG654372	SH1723072.08FU	*G. parvulum*
421	*G. subamboinense*	MK531824/MK531822		*G. parvulum*
422	*Ganoderma* sp.	MK554792		*G. parvulum*
423	*G. weberianum*	MT232635		*G. parvulum*

424	*G. weberianum*	JN596332	SH1678462.08FU	*G. parvulum*
425	*G. resinaceum*	MH106883	SH2762559.08FU	*Ganoderma* sp. A2
426	*Ganoderma* sp.	MK554791		*Ganoderma* sp. A2
427	*G. resinaceum*	AM269775	SH1723046.08FU	*G. resinaceum*
428	*G. resinaceum*	AM269776	SH1723046.08FU	*G. resinaceum*
429	*G. resinaceum*	AM269777	SH1723046.08FU	*G. resinaceum*
430	*G. resinaceum*	AM269778	SH1723046.08FU	*G. resinaceum*
431	*G. resinaceum*	AM906060	SH1723046.08FU	*G. resinaceum*
432	*G. resinaceum*	AM906061	SH1723046.08FU	*G. resinaceum*
433	*G. resinaceum*	AM906062	SH1723046.08FU	*G. resinaceum*
434	*G. resinaceum*	AM906063	SH1723046.08FU	*G. resinaceum*
435	*G. resinaceum*	AM906064	SH1723046.08FU	*G. resinaceum*
436	*G. resinaceum*	AM906065		*G. resinaceum*
437	*G. resinaceum*	AY884177	SH1723046.08FU	*G. resinaceum*
438	*G. resinaceum*	EF060007	SH1723046.08FU	*G. resinaceum*
439	*G. resinaceum*	FJ491948		*G. resinaceum*
440	*G. resinaceum*	FJ491949	SH1723046.08FU	*G. resinaceum*
441	*G. resinaceum*	FJ491950		*G. resinaceum*
442	*G. resinaceum*	FJ491951		*G. resinaceum*
443	*G. resinaceum*	FJ491952	SH1723046.08FU	*G. resinaceum*
444	*G. resinaceum*	FJ491953		*G. resinaceum*
445	*G. resinaceum*	FJ491954		*G. resinaceum*
446	*G. resinaceum*	FJ491955		*G. resinaceum*
447	*G. resinaceum*	FJ491956	SH1723046.08FU	*G. resinaceum*
448	*G. resinaceum*	FJ491957		*G. resinaceum*
449	*G. resinaceum*	FJ665693		*G. resinaceum*
450	*G. resinaceum*	FJ665694		*G. resinaceum*
451	*G. resinaceum*	FJ805250	SH1723046.08FU	*G. resinaceum*
452	*G. resinaceum*	GU451246	SH1723046.08FU	*G. resinaceum*
453	*G. resinaceum*	GU451247	SH1723046.08FU	*G. resinaceum*
454	*G. resinaceum*	GU731559	SH1723046.08FU	*G. resinaceum*
455	*G. resinaceum*	JN008875		*G. resinaceum*
456	*G. resinaceum*	JN176880		*G. resinaceum*
457	*G. resinaceum*	JN176881		*G. resinaceum*
458	*G. resinaceum*	JN176882		*G. resinaceum*
459	*G. resinaceum*	JN176885		*G. resinaceum*
460	*G. resinaceum*	JN176896	SH1723046.08FU	*G. resinaceum*
461	*G. resinaceum*	JN222406	SH1723046.08FU	*G. resinaceum*
462	*G. resinaceum*	JN588588	SH1723046.08FU	*G. resinaceum*
463	*G. resinaceum*	JN588589	SH1723046.08FU	*G. resinaceum*
464	*G. resinaceum*	JN588590	SH1723046.08FU	*G. resinaceum*
465	*G. resinaceum*	JQ520200	SH1723046.08FU	*G. resinaceum*
466	*G. resinaceum*	JQ520203		*G. resinaceum*
467	*G. resinaceum*	JQ520204		*G. resinaceum*
468	*G. resinaceum*	JQ627588	SH1723046.08FU	*G. resinaceum*

469	*G. resinaceum*	JX082326		*G. resinaceum*
470	*G. resinaceum*	JX082328	SH1723046.08FU	*G. resinaceum*
471	*G. resinaceum*	KF605627		*G. resinaceum*
472	*G. resinaceum*	KF605628		*G. resinaceum*
473	*G. resinaceum*	KF975890	SH1723046.08FU	*G. resinaceum*
474	*G. resinaceum*	KJ143915	SH1723046.08FU	*G. resinaceum*
475	*G. resinaceum*	KJ143916	SH1723046.08FU	*G. resinaceum*
476	*G. resinaceum*	KJ509597		*G. resinaceum*
477	*G. resinaceum*	KJ509598	SH1723046.08FU	*G. resinaceum*
478	*G. resinaceum*	KP941447	SH1723046.08FU	*G. resinaceum*
479	*G. resinaceum*	KT343303	SH1723046.08FU	*G. resinaceum*
480	*G. resinaceum*	KT343307	SH1723046.08FU	*G. resinaceum*
481	*G. resinaceum*	KT343308		*G. resinaceum*
482	*G. resinaceum*	KT343309	SH1723046.08FU	*G. resinaceum*
483	*G. resinaceum*	KT343310	SH1723046.08FU	*G. resinaceum*
484	*G. resinaceum*	KT343317	SH1723046.08FU	*G. resinaceum*
485	*G. resinaceum*	KT906370		*G. resinaceum*
486	*G. resinaceum*	KT906371	SH1723046.08FU	*G. resinaceum*
487	*G. resinaceum*	KT921215		*G. resinaceum*
488	*G. resinaceum*	LN774970	SH1723046.08FU	*G. resinaceum*
489	*G. resinaceum*	MG706233		*G. resinaceum*
490	*G. resinaceum*	MG706234	SH1723046.08FU	*G. resinaceum*
491	*G. resinaceum*	MG706235	SH1723046.08FU	*G. resinaceum*
492	*G. resinaceum*	MG706236	SH1723046.08FU	*G. resinaceum*
493	*G. resinaceum*	MG706238	SH1723046.08FU	*G. resinaceum*
494	*G. resinaceum*	MG706240	SH1723046.08FU	*G. resinaceum*
495	*G. resinaceum*	MG706241	SH1723046.08FU	*G. resinaceum*
496	*G. resinaceum*	MG706242	SH1723046.08FU	*G. resinaceum*
497	*G. resinaceum*	MG706243	SH1723046.08FU	*G. resinaceum*
498	*G. resinaceum*	MG706245		*G. resinaceum*
499	*G. resinaceum*	MG706246	SH1723046.08FU	*G. resinaceum*
500	*G. resinaceum*	MG706247	SH1723046.08FU	*G. resinaceum*
501	*G. resinaceum*	MG706248	SH1723046.08FU	*G. resinaceum*
502	*G. resinaceum*	MG706249	SH1723046.08FU	*G. resinaceum*
503	*G. resinaceum*	MG706250	SH1723046.08FU	*G. resinaceum*
504	*G. resinaceum*	MG706251	SH1723046.08FU	*G. resinaceum*
505	*G. resinaceum*	MG706252	SH1723046.08FU	*G. resinaceum*
506	*G. resinaceum*	MG706256	SH1723046.08FU	*G. resinaceum*
507	*G. resinaceum*	MH796122	SH1723046.08FU	*G. resinaceum*
508	*G. resinaceum*	MH854909	SH1723046.08FU	*G. resinaceum*
509	*G. resinaceum*	MK415241		*G. resinaceum*
510	*G. resinaceum*	MK415274		*G. resinaceum*
511	*G. resinaceum*	MK415304		*G. resinaceum*
512	*G. resinaceum*	MK415305		*G. resinaceum*
513	*G. resinaceum*	MK415309		*G. resinaceum*

514	*G. resinaceum*	MK415311		*G. resinaceum*
515	*G. resinaceum*	MK422153	SH1723046.08FU	*G. resinaceum*
516	*G. resinaceum*	MK554766		*G. resinaceum*
517	*G. resinaceum*	MK554772		*G. resinaceum*
518	*G. resinaceum*	MK554775		*G. resinaceum*
519	*G. resinaceum*	MK554786		*G. resinaceum*
520	*G. resinaceum*	MN240470	SH1723046.08FU	*G. resinaceum*
521	*G. resinaceum*	MN240471	SH1723046.08FU	*G. resinaceum*
522	*G. resinaceum*	MN398315	SH1723046.08FU	*G. resinaceum*
523	*G. resinaceum*	MN448375	SH1723046.08FU	*G. resinaceum*
524	*G. resinaceum*	MN759653		*G. resinaceum*
525	*G. resinaceum*	MT581895		*G. resinaceum*
526	*G. resinaceum*	MT581896		*G. resinaceum*
527	*G. resinaceum*	X78737/ X78758 (=KJ143916)		*G. resinaceum*
528	*G. resinaceum*	Z37062/Z37085		*G. resinaceum*
529	*G. pfeifferi*	AM269774	SH1723046.08FU	*G. resinaceum*
530	Polyporales *sp.*	JQ312182	SH1723046.08FU	*G. resinaceum*
531	Polyporales *sp.*	JQ312204	SH1723046.08FU	*G. resinaceum*
532	*G. pfeifferi*	JQ520198		*G. resinaceum*
533	*Ganoderma* sp.	KT223756	SH1723046.08FU	*G. resinaceum*
534	*Ganoderma* sp.	KT223758	SH1723046.08FU	*G. resinaceum*
535	*G. lucidum*	KT343316	SH1723046.08FU	*G. resinaceum*
536	*G. lucidum*	KX371594		*G. resinaceum*
537	*G. lucidum*	KX371595	SH1723046.08FU	*G. resinaceum*
538	*G. lucidum*	KX371596	SH1723046.08FU	*G. resinaceum*
539	*G. pfeifferi*	KY196415	SH1723046.08FU	*G. resinaceum*
540	*G. lucidum*	MG706237	SH1723046.08FU	*G. resinaceum*
541	*G. lucidum*	MG706239	SH1723046.08FU	*G. resinaceum*
542	*G. pfeifferi*	MG706244	SH1723046.08FU	*G. resinaceum*
543	*Ganoderma* sp.	MG706253	SH1723046.08FU	*G. resinaceum*
544	*Ganoderma* sp.	MG706254	SH1723046.08FU	*G. resinaceum*
545	*G. pfeifferi*	MG706255	SH1723046.08FU	*G. resinaceum*
546	*Ganoderma* sp.	MH290273	SH1723046.08FU	*G. resinaceum*
547	*Ganoderma* sp.	MH290277	SH1723046.08FU	*G. resinaceum*
548	*Ganoderma* sp.	MH290285	SH1723046.08FU	*G. resinaceum*
549	*G.* cf. *resinaceum*	MH571691	SH1723046.08FU	*G. resinaceum*
550	*G.* cf. *resinaceum*	MH571692	SH1723046.08FU	*G. resinaceum*
551	*Ganoderma* sp.	MK531816		*G. resinaceum*
552	*G. pfeifferi*	X78738/X78759 (=JQ520198)		*G. resinaceum*
553	*G. resinaceum*	MG835866		*G. resinaceum*
554	*G. resinaceum*	MK773879	SH2758628.08FU	*G. resinaceum*
555	*G. resinaceum*	MN114053	SH2767376.08FU	*G. resinaceum*
556	*G. lucidum*	MT703901		*G. resinaceum*
557	*G. lucidum*	MT708677		*G. resinaceum*
558	uncultured *Ganoderma*	GQ268631	SH1723084.08FU	*Ganoderma* sp. A3

559	*G. lucidum*	JQ520187	SH1723084.08FU	*Ganoderma* sp. A3
560	*G. resinaceum*	JX270804		*Ganoderma* sp. A3
561	*G. resinaceum*	KT693256		*Ganoderma* sp. A3
562	*G. resinaceum*	KT693257		*Ganoderma* sp. A3
563	*G. resinaceum*	KT693258		*Ganoderma* sp. A3
564	*G. resinaceum*	KT693259		*Ganoderma* sp. A3
565	*Ganoderma* sp.	KT943367	SH1723084.08FU	*Ganoderma* sp. A3
566	*Ganoderma* sp.	KT943368	SH1723084.08FU	*Ganoderma* sp. A3
567	*G.* cf. *resinaceum*	MH160062	SH1723084.08FU	*Ganoderma* sp. A3
568	*G.* cf. *resinaceum*	MH160068	SH1723084.08FU	*Ganoderma* sp. A3
569	*G.* cf. *resinaceum*	MH160069	SH1723084.08FU	*Ganoderma* sp. A3
570	*G. platense*	AF170001/2 & AH008109		*G.* aff. *polychromum*
571	*G. zonatum*	AF170003/4 & AH008110		*G.* aff. *polychromum*
572	*G. sessile*	AF170005/6 & AH008111		*G.* aff. *polychromum*
573	*G. resinaceum*	KC311372		*G.* aff. *polychromum*
574	*G. resinaceum*	KC311373	SH1723162.08FU	*G.* aff. *polychromum*
575	*G. resinaceum*	KC311374	SH1723162.08FU	*G.* aff. *polychromum*
576	*G. sessile*	KF605631	SH1723226.08FU	*G.* aff. *polychromum*
577	*G.* cf. *sessile*	MT232415		*G.* aff. *polychromum*
578	*G. resinaceum*	FJ850966		*G.* aff. *polychromum*
579	*G. resinaceum*	FJ850967		*G.* aff. *polychromum*
580	*G. lucidum*	AY870653	SH1723046.08FU	*G.* aff. *sessile*
581	*G. lucidum*	FJ463905	SH1723202.08FU	*G.* aff. *sessile*
582	*G. lucidum*	FJ463913	SH1723202.08FU	*G.* aff. *sessile*
583	*G. lucidum*	FJ463923	SH1723202.08FU	*G.* aff. *sessile*
584	*G. polychromum*	MG654196		*G. polychromum*
585	*G. polychromum*	MG654197		*G. polychromum*
586	*G. polychromum*	MG654198		*G. polychromum*
587	*G. polychromum*	MG654199		*G. polychromum*
588	*G. polychromum*	MG654201		*G. polychromum*
589	*G. polychromum*	MG654202		*G. polychromum*
590	*G. polychromum*	MG654203		*G. polychromum*
591	*G. polychromum*	MG654204		*G. polychromum*
592	*G. polychromum*	MG654205		*G. polychromum*
593	*G. polychromum*	MG910492		*G. polychromum*
594	*G. polychromum*	MG910493		*G. polychromum*
595	*G. lucidum*	AM269772		*G. polychromum*
596	*G. lucidum*	DQ424998		*G. polychromum*
597	*G. lucidum*	FJ463915		*G. polychromum*
598	*G. lucidum*	FJ463926		*G. polychromum*
599	*G. lucidum*	FJ655477	SH1723187.08FU	*G. polychromum*
600	*G. sessile*	KF605632		*G. polychromum*
601	*G. sessile*	KF605633		*G. polychromum*
602	*G. lucidum*	MN533787		*G. polychromum*
603	*G. sessile*	KF605629		*G. sessile*

604	*G. sessile*	KF605630	*G. sessile*
605	*G. sessile*	KF605634	*G. sessile*
606	*G. sessile*	KF605635	*G. sessile*
607	*G. sessile*	KF605636	*G. sessile*
608	*G. sessile*	KF605637	*G. sessile*
609	*G. sessile*	KF605638	*G. sessile*
610	*G. sessile*	KF605639	*G. sessile*
611	*G. sessile*	KF605640	*G. sessile*
612	*G. sessile*	KJ143917	*G. sessile*
613	*G. sessile*	KJ143918	*G. sessile*
614	*G. sessile*	KY646213 (=MG654307)	*G. sessile*
615	*G. sessile*	KY646214 (=MG654309)	*G. sessile*
616	*G. sessile*	KY708882	*G. sessile*
617	*G. sessile*	KY708883	*G. sessile*
618	*G. sessile*	KY708884	*G. sessile*
619	*G. sessile*	MG654212	*G. sessile*
620	G. sessile	MG654213	*G. sessile*
621	G. sessile	MG654214	*G. sessile*
622	*G. sessile*	MG654215	*G. sessile*
623	*G. sessile*	MG654216	*G. sessile*
624	*G. sessile*	MG654217	*G. sessile*
625	*G. sessile*	MG654218	*G. sessile*
626	*G. sessile*	MG654219	*G. sessile*
627	*G. sessile*	MG654220	*G. sessile*
628	*G. sessile*	MG654221	*G. sessile*
629	*G. sessile*	MG654222	*G. sessile*
630	*G. sessile*	MG654223	*G. sessile*
631	*G. sessile*	MG654224	*G. sessile*
632	*G. sessile*	MG654225	*G. sessile*
633	*G. sessile*	MG654226	*G. sessile*
634	*G. sessile*	MG654227	*G. sessile*
635	*G. sessile*	MG654228	*G. sessile*
636	*G. sessile*	MG654229	*G. sessile*
637	*G. sessile*	MG654230	*G. sessile*
638	*G. sessile*	MG654231	*G. sessile*
639	*G. sessile*	MG654232	*G. sessile*
640	*G. sessile*	MG654233	*G. sessile*
641	*G. sessile*	MG654234	*G. sessile*
642	*G. sessile*	MG654235	*G. sessile*
643	*G. sessile*	MG654236	*G. sessile*
644	*G. sessile*	MG654237	*G. sessile*
645	*G. sessile*	MG654238	*G. sessile*
646	*G. sessile*	MG654239	*G. sessile*
647	*G. sessile*	MG654240	*G. sessile*

648	*G. sessile*	MG654241	*G. sessile*
649	*G. sessile*	MG654242	*G. sessile*
650	*G. sessile*	MG654243	*G. sessile*
651	*G. sessile*	MG654244	*G. sessile*
652	*G. sessile*	MG654245	*G. sessile*
653	*G. sessile*	MG654246	*G. sessile*
654	*G. sessile*	MG654247	*G. sessile*
655	*G. sessile*	MG654248	*G. sessile*
656	*G. sessile*	MG654249	*G. sessile*
657	*G. sessile*	MG654250	*G. sessile*
658	*G. sessile*	MG654251	*G. sessile*
659	*G. sessile*	MG654252	*G. sessile*
660	*G. sessile*	MG654253	*G. sessile*
661	*G. sessile*	MG654254	*G. sessile*
662	*G. sessile*	MG654255	*G. sessile*
663	*G. sessile*	MG654256	*G. sessile*
664	*G. sessile*	MG654257	*G. sessile*
665	*G. sessile*	MG654258	*G. sessile*
666	*G. sessile*	MG654259	*G. sessile*
667	*G. sessile*	MG654260	*G. sessile*
668	*G. sessile*	MG654261	*G. sessile*
669	*G. sessile*	MG654262	*G. sessile*
670	*G. sessile*	MG654263	*G. sessile*
671	*G. sessile*	MG654264	*G. sessile*
672	*G. sessile*	MG654265	*G. sessile*
673	*G. sessile*	MG654266	*G. sessile*
674	*G. sessile*	MG654267	*G. sessile*
675	*G. sessile*	MG654268	*G. sessile*
676	*G. sessile*	MG654269	*G. sessile*
677	*G. sessile*	MG654270	*G. sessile*
678	*G. sessile*	MG654271	*G. sessile*
679	*G. sessile*	MG654272	*G. sessile*
680	*G. sessile*	MG654273	*G. sessile*
681	*G. sessile*	MG654274	*G. sessile*
682	*G. sessile*	MG654275	*G. sessile*
683	*G. sessile*	MG654276	*G. sessile*
684	*G. sessile*	MG654277	*G. sessile*
685	*G. sessile*	MG654278	*G. sessile*
686	*G. sessile*	MG654279	*G. sessile*
687	*G. sessile*	MG654280	*G. sessile*
688	*G. sessile*	MG654281	*G. sessile*
689	*G. sessile*	MG654282	*G. sessile*
690	*G. sessile*	MG654283	*G. sessile*
691	*G. sessile*	MG654284	*G. sessile*
692	*G. sessile*	MG654285	*G. sessile*

693	*G. sessile*	MG654286		*G. sessile*
694	*G. sessile*	MG654287		*G. sessile*
695	*G. sessile*	MG654288		*G. sessile*
696	*G. sessile*	MG654289		*G. sessile*
697	*G. sessile*	MG654290		*G. sessile*
698	*G. sessile*	MG654291		*G. sessile*
699	*G. sessile*	MG654292		*G. sessile*
700	*G. sessile*	MG654293		*G. sessile*
701	*G. sessile*	MG654294		*G. sessile*
702	*G. sessile*	MG654295		*G. sessile*
703	*G. sessile*	MG654296		*G. sessile*
704	*G. sessile*	MG654297		*G. sessile*
705	*G. sessile*	MG654298		*G. sessile*
706	*G. sessile*	MG654299		*G. sessile*
707	*G. sessile*	MG654300		*G. sessile*
708	*G. sessile*	MG654301		*G. sessile*
709	*G. sessile*	MG654302		*G. sessile*
710	*G. sessile*	MG654303		*G. sessile*
711	*G. sessile*	MG654304		*G. sessile*
712	*G. sessile*	MG654305		*G. sessile*
713	*G. sessile*	MG654306		*G. sessile*
714	*G. sessile*	MG654307		*G. sessile*
715	*G. sessile*	MG654308		*G. sessile*
716	*G. sessile*	MG654309		*G. sessile*
717	*G. sessile*	MG654310	SH1723037.08FU	*G. sessile*
718	*G. sessile*	MG654311	SH1723037.08FU	*G. sessile*
719	*G. sessile*	MG654312		*G. sessile*
720	*G. sessile*	MG654313		*G. sessile*
721	*G. sessile*	MG654314		*G. sessile*
722	*G. sessile*	MG654315		*G. sessile*
723	*G. sessile*	MG654316		*G. sessile*
724	*G. sessile*	MG654317		*G. sessile*
725	*G. sessile*	MG654318		*G. sessile*
726	*G. sessile*	MG654319		*G. sessile*
727	*G. sessile*	MG654320		*G. sessile*
728	*G. sessile*	MG773847		*G. sessile*
729	*G. sessile*	MG910998		*G. sessile*
730	*G. sessile*	MH160056		*G. sessile*
731	*G. sessile*	MH160057		*G. sessile*
732	*G. sessile*	MH160059		*G. sessile*
733	*G. sessile*	MH160060		*G. sessile*
734	*G. sessile*	MK554778		*G. sessile*
735	*G. sessile*	MN430930		*G. sessile*
736	*G. sessile*	MT196418		*G. sessile*
737	*Ganoderma* sp.	AY508882		*G. sessile*

738	*G. lucidum*	AY636059		*G. sessile*
739	*G. lucidum*	DQ424999		*G. sessile*
740	*Hericium erinaceum*	EU520249		*G. sessile*
741	uncultured *Ganoderma*	FJ362324		*G. sessile*
742	*G. lucidum*	FJ463906		*G. sessile*
743	*G. lucidum*	FJ463924		*G. sessile*
744	*G. lucidum*	FJ501561		*G. sessile*
745	*G. resinaceum*	JN021025		*G. sessile*
746	*G. lobatum*	JQ520166		*G. sessile*
747	*G. neojaponicum*	JQ520193		*G. sessile*
748	*G. oregonense*	JQ520194		*G. sessile*
749	*G. oregonense*	JQ520197		*G. sessile*
750	*G. resinaceum*	JQ520199		*G. sessile*
751	*G. resinaceum*	JQ520201		*G. sessile*
752	*G. resinaceum*	JQ520202		*G. sessile*
753	*Ganoderma* sp.	JQ520208		*G. sessile*
754	*Ganoderma* sp.	JQ520209		*G. sessile*
755	*G. valesiacum*	JQ520218		*G. sessile*
756	*Ganoderma* sp.	KC514812		*G. sessile*
757	*Ganoderma* sp.	KC514839		*G. sessile*
758	*G. lucidum*	KF648564	SH1678469.08FU	*G. sessile*
759	*G. resinaceum*	KJ857267		*G. sessile*
760	*Ganoderma* sp.	KT223755		*G. sessile*
761	*G. resinaceum*	KX055526		*G. sessile*
762	*G. resinaceum*	KX055530		*G. sessile*
763	*G. resinaceum*	KX055540		*G. sessile*
764	*G. resinaceum*	KX055542		*G. sessile*
765	*G. resinaceum*	KX055549		*G. sessile*
766	*G. resinaceum*	KX371938		*G. sessile*
767	*G. resinaceum*	KX371939		*G. sessile*
768	*G. resinaceum*	KX371940		*G. sessile*
769	*G. resinaceum*	KX371941		*G. sessile*
770	*G. resinaceum*	KX371942		*G. sessile*
771	*G. resinaceum*	KX371943		*G. sessile*
772	*G. resinaceum*	KX371944		*G. sessile*
773	*G. resinaceum*	KX371945		*G. sessile*
774	*G. resinaceum*	KX371946		*G. sessile*
775	*G. resinaceum*	KX371947		*G. sessile*
776	*G. resinaceum*	KX371948		*G. sessile*
777	*G. resinaceum*	KX371949		*G. sessile*
778	*G. resinaceum*	KX371950		*G. sessile*
779	*G. resinaceum*	KX371951		*G. sessile*
780	*G. resinaceum*	KX371952		*G. sessile*
781	*G. resinaceum*	KX371953		*G. sessile*

782	*G. resinaceum*	KX371954		*G. sessile*
783	*G. resinaceum*	KX371955		*G. sessile*
784	*G. resinaceum*	KX371956		*G. sessile*
785	*G. resinaceum*	KX371957		*G. sessile*
786	*G. resinaceum*	KX371958		*G. sessile*
787	*G. resinaceum*	KX371959		*G. sessile*
788	*G. resinaceum*	KX371960		*G. sessile*
789	*G. resinaceum*	KX371961		*G. sessile*
790	*G. resinaceum*	KX371962		*G. sessile*
791	*G. resinaceum*	KX371963	SH1723281.08FU	*G. sessile*
792	*G. resinaceum*	KX371964		*G. sessile*
793	*G. resinaceum*	KX371965		*G. sessile*
794	*G. resinaceum*	KX371966		*G. sessile*
795	*G. resinaceum*	KX371967		*G. sessile*
796	*G. resinaceum*	KX371968		*G. sessile*
797	*G. resinaceum*	KX371969		*G. sessile*
798	*G. resinaceum*	KX371970		*G. sessile*
799	*G. resinaceum*	KX371971		*G. sessile*
800	*G. resinaceum*	KX371972		*G. sessile*
801	*G. resinaceum*	KX371973		*G. sessile*
802	*G. resinaceum*	KX371974		*G. sessile*
803	*G. resinaceum*	KX371975		*G. sessile*
804	*G. resinaceum*	KX371976		*G. sessile*
805	*G. resinaceum*	KX371977		*G. sessile*
806	*G. resinaceum*	KX371978		*G. sessile*
807	*G. resinaceum*	KX371979		*G. sessile*
808	*G. resinaceum*	KX371980		*G. sessile*
809	*G. resinaceum*	KX371981		*G. sessile*
810	*G. resinaceum*	KX371982		*G. sessile*
811	*G. resinaceum*	KX957799	SH1678469.08FU	*G. sessile*
812	*G. lucidum*	MF755277		*G. sessile*
813	*G. polychromum*	MG654200		*G. sessile*
814	*Ganoderma* sp.	MH018025		*G. sessile*
815	*G. resinaceum*	MH027637		*G. sessile*
816	*G. resinaceum*	MH172163		*G. sessile*
817	*Ganoderma* sp.	MH290270		*G. sessile*
818	*Ganoderma* sp.	MH290283		*G. sessile*
819	*Ganoderma* sp.	MH290286		*G. sessile*
820	*Ganoderma* sp.	MH294318		*G. sessile*
821	*Ganoderma* sp.	MH294326		*G. sessile*
822	*G. resinaceum*	MH855781		*G. sessile*
823	*Ganoderma* sp.	MH921462		*G. sessile*
824	*Ganoderma* sp.	MK991834		*G. sessile*
825	*G. resinaceum*	MN622781		*G. sessile*
826	*Ganoderma* sp.	MN911336		*G. sessile*

827	*G. boninense*	X78749/X78770		*G. sessile*
828	*G. lucidum*	Z37051/Z37075		*G. sessile*
829	*G. lucidum*	Z37053/Z37077		*G. sessile*
830	*G. lucidum*	MK773881		*G. sessile*
831	*G. concinnum*	MN077522		*G. concinnum*
832	*G. chalceum*	JX310812	SH1723222.08FU	*G. concinnum*
833	*G. tuberculosum*	KF963255		*G. tuberculosum*
834	*G. tuberculosum*	KF963258		*G. tuberculosum*
835	*G. tuberculosum*	KY646215		*G. tuberculosum*
836	*G. tuberculosum*	KY646216		*G. tuberculosum*
837	*G. tuberculosum*	KY708885		*G. tuberculosum*
838	*G. tuberculosum*	KY709317		*G. tuberculosum*
839	*G. tuberculosum*	MG654351		*G. tuberculosum*
840	*G. tuberculosum*	MG654352	SH1723125.08FU	*G. tuberculosum*
841	*G. tuberculosum*	MG654353		*G. tuberculosum*
842	*G. tuberculosum*	MG654354		*G. tuberculosum*
843	*G. tuberculosum*	MG654355		*G. tuberculosum*
844	*G. tuberculosum*	MG654356		*G. tuberculosum*
845	*G. tuberculosum*	MG654357		*G. tuberculosum*
846	*G. tuberculosum*	MG654358		*G. tuberculosum*
847	*G. tuberculosum*	MG654359		*G. tuberculosum*
848	*G. tuberculosum*	MG654360		*G. tuberculosum*
849	*G. tuberculosum*	MG654361		*G. tuberculosum*
850	*G. tuberculosum*	MG654362		*G. tuberculosum*
851	*G. tuberculosum*	MG654363		*G. tuberculosum*
852	*G. tuberculosum*	MG654364		*G. tuberculosum*
853	*G. tuberculosum*	MG654365		*G. tuberculosum*
854	*G. tuberculosum*	MG654366		*G. tuberculosum*
855	*G. tuberculosum*	MG654367		*G. tuberculosum*
856	*G. tuberculosum*	MG654368		*G. tuberculosum*
857	*G. tuberculosum*	MG654369		*G. tuberculosum*
858	*G. tuberculosum*	MT232634		*G. tuberculosum*
859	*G. tuberculosum*	MT232639		*G. tuberculosum*
860	*Coriolopsis caperata*	EU030178		*G. tuberculosum*
861	*G. resinaceum*	JX310824		*G. tuberculosum*
862	*Ganoderma* sp.	KU569517	SH1723125.08FU	*G. tuberculosum*
863	*G. parvulum*	KU569528	SH1723125.08FU	*G. tuberculosum*
864	*Ganoderma* sp.	LT726719		*G. tuberculosum*
865	*Ganoderma* sp.	LT726725		*G. tuberculosum*
866	*Ganoderma* sp.	LT726726	SH1723236.08FU	*G. tuberculosum*
867	*Ganoderma* sp.	LT726728		*G. tuberculosum*
868	*Ganoderma* sp.	LT726729		*G. tuberculosum*
869	*G. tuberculosum*	MT232636		*G. tuberculosum*
870	*G. lucidum*	AF170007/8 & AH008112		*Ganoderma* sp. A4
871	*G. lucidum*	AF170009/10 & AH008113		*Ganoderma* sp. A4

872	*G. wiiroense*	KT952361	SH1720236.08FU	*G. wiiroense*
873	*G. wiiroense*	KT952363/NR_158480	SH1720236.08FU	*G. wiiroense*
874	*G. wiiroense*	KY009864	SH1720246.08FU	*G. wiiroense*
875	*G. wiiroense*	KY009867	SH1720241.08FU	*G. wiiroense*
876	*G. wiiroense*	KY009869	SH1720243.08FU	*G. wiiroense*
877	*G. wiiroense*	KY009873	SH1720236.08FU	*G. wiiroense*
878	*G. wiiroense*	KY111253	SH1720236.08FU	*G. wiiroense*
879	*G. wiiroense*	KY963355	SH1720236.08FU	*G. wiiroense*
880	*G. wiiroense*	MF774620	SH1720236.08FU	*G. wiiroense*
881	*G. lucidum*	FJ982798	SH1720236.08FU	*G. wiiroense*
882	*Ganoderma* sp.	KJ510534	SH1720236.08FU	*G. wiiroense*
883	*G. lucidum*	MH553170		*G. wiiroense*
884	*Ganoderma* sp.	MH889141	SH1720236.08FU	*G. wiiroense*
885	*Ganoderma* sp.	KY968687	SH1852184.08FU	*G. wiiroense*
886	*G. flexipes*	JN383978	SH1723276.08FU	*G. flexipes*
887	*G. flexipes*	JN383979	SH1723166.08FU	*G. flexipes*
888	*G. flexipes*	JQ781850		*G. flexipes*
889	*G. flexipes*	MH106873	SH1723276.08FU	*G. flexipes*
890	*G. flexipes*	MK345430	SH2766297.08FU	*G. flexipes*
891	*G. flexipes*	MK345431	SH2766297.08FU	*G. flexipes*
892	*G. flexipes*	MN398340	SH2766297.08FU	*G. flexipes*
893	*G. multiplicatum*	KU572488	SH1723120.08FU	*G. multiplicatum*
894	*G. multiplicatum*	KU572489	SH1723120.08FU	*G. multiplicatum*
895	*G. multiplicatum*	KU572490	SH1723120.08FU	*G. multiplicatum*
896	*G. multiplicatum*	MH106879	SH1723120.08FU	*G. multiplicatum*
897	*G. multiplicatum*	MK345439		*G. multiplicatum*
898	*G. multiplicatum*	MK345440	SH1723120.08FU	*G. multiplicatum*
899	*G. multiplicatum*	MN401405	SH1723120.08FU	*G. multiplicatum*
900	*G. philippii*	AJ536662	SH1723042.08FU	*G. philippii*
901	*G. philippii*	AJ608710	SH1723042.08FU	*G. philippii*
902	*G. philippii*	AJ608713	SH1723042.08FU	*G. philippii*
903	*G. philippii*	AJ608714/5		*G. philippii*
904	*G. philippii*	AJ627584	SH1723042.08FU	*G. philippii*
905	*G. pseudoferreum*	FJ374874	SH1723042.08FU	*G. philippii*
906	*G. pseudoferreum*	FJ374875	SH1723042.08FU	*G. philippii*
907	*G. pseudoferreum*	FJ374876	SH1723042.08FU	*G. philippii*
908	*G. pseudoferreum*	FJ378651	SH1723042.08FU	*G. philippii*
909	*G. pseudoferreum*	FJ392279	SH1723042.08FU	*G. philippii*
910	*G. pseudoferreum*	FJ392280		*G. philippii*
911	*G. pseudoferreum*	FJ392281	SH1723042.08FU	*G. philippii*
912	*G. pseudoferreum*	FJ392282	SH1723042.08FU	*G. philippii*
913	*G. pseudoferreum*	FJ392283	SH1723042.08FU	*G. philippii*
914	*G. pseudoferreum*	FJ392284	SH1723042.08FU	*G. philippii*
915	*G. pseudoferreum*	FJ392285	SH1723042.08FU	*G. philippii*
916	*G. pseudoferreum*	KX454334		*G. philippii*

917	*G. pseudoferreum*	KX454335		*G. philippii*
918	*G. pseudoferreum*	KX454336		*G. philippii*
919	*G. pseudoferreum*	KX454337	SH1723042.08FU	*G. philippii*
920	*G. pseudoferreum*	KX454338		*G. philippii*
921	*G. pseudoferreum*	KX454339		*G. philippii*
922	*G. pseudoferreum*	KX454340	SH1723042.08FU	*G. philippii*
923	*G. pseudoferreum*	KX454341	SH1723042.08FU	*G. philippii*
924	*G. pseudoferreum*	KX454342		*G. philippii*
925	*G. pseudoferreum*	KX454343	SH1723042.08FU	*G. philippii*
926	*G. pseudoferreum*	KX454344		*G. philippii*
927	*G. pseudoferreum*	KX454345		*G. philippii*
928	*G. pseudoferreum*	KX454346		*G. philippii*
929	*G. pseudoferreum*	KX454347	SH1723042.08FU	*G. philippii*
930	*G. pseudoferreum*	KX454348	SH1723042.08FU	*G. philippii*
931	*G. pseudoferreum*	KX454349		*G. philippii*
932	*G. pseudoferreum*	KX454350	SH1723042.08FU	*G. philippii*
933	*G. pseudoferreum*	KX454351	SH1723042.08FU	*G. philippii*
934	*G. pseudoferreum*	KX454352	SH1723042.08FU	*G. philippii*
935	*G. pseudoferreum*	KX454353	SH1723042.08FU	*G. philippii*
936	*G. pseudoferreum*	KX454354	SH1723042.08FU	*G. philippii*
937	*G. pseudoferreum*	KX454376	SH1723042.08FU	*G. philippii*
938	*G. pseudoferreum*	KX454377	SH1723042.08FU	*G. philippii*
939	*G. pseudoferreum*	KX454378	SH1723042.08FU	*G. philippii*
940	*G. pseudoferreum*	KX454379	SH1723042.08FU	*G. philippii*
941	*G. pseudoferreum*	KX454380	SH1723042.08FU	*G. philippii*
942	*G. pseudoferreum*	KX454381	SH1723042.08FU	*G. philippii*
943	*G. pseudoferreum*	KX454382	SH1723042.08FU	*G. philippii*
944	*G. pseudoferreum*	KX454383	SH1723042.08FU	*G. philippii*
945	*G. pseudoferreum*	KX454384	SH1723042.08FU	*G. philippii*
946	*G. pseudoferreum*	KX454385	SH1723042.08FU	*G. philippii*
947	*G. pseudoferreum*	KX454386	SH1723042.08FU	*G. philippii*
948	*G. pseudoferreum*	KX454387	SH1723042.08FU	*G. philippii*
949	*G. pseudoferreum*	KX454388	SH1723042.08FU	*G. philippii*
950	*G. pseudoferreum*	KX454389	SH1723042.08FU	*G. philippii*
951	*G. pseudoferreum*	KX454390	SH1723042.08FU	*G. philippii*
952	*G. pseudoferreum*	KX454391	SH1723042.08FU	*G. philippii*
953	*G. pseudoferreum*	KX454392		*G. philippii*
954	*G. pseudoferreum*	KX454393	SH1723042.08FU	*G. philippii*
955	*G. pseudoferreum*	KX454394	SH1723042.08FU	*G. philippii*
956	*G. pseudoferreum*	KX454395	SH1723042.08FU	*G. philippii*
957	*G. pseudoferreum*	KX454396		*G. philippii*
958	*G. pseudoferreum*	KX454397	SH1723042.08FU	*G. philippii*
959	*G. pseudoferreum*	KX454398	SH1723042.08FU	*G. philippii*
960	*G. pseudoferreum*	KX454399		*G. philippii*
961	*G. pseudoferreum*	KX454400	SH1723042.08FU	*G. philippii*

962	*G. pseudoferreum*	KX454401	SH1723042.08FU	*G. philippii*
963	*G. pseudoferreum*	KX454402	SH1723042.08FU	*G. philippii*
964	*G. pseudoferreum*	KX454403	SH1723042.08FU	*G. philippii*
965	*G. pseudoferreum*	KX454404	SH1723042.08FU	*G. philippii*
966	*G. pseudoferreum*	KX454405	SH1723042.08FU	*G. philippii*
967	*G. pseudoferreum*	KX454406	SH1723042.08FU	*G. philippii*
968	*G. pseudoferreum*	KX454407	SH1723042.08FU	*G. philippii*
969	*G. pseudoferreum*	KX454408	SH1723042.08FU	*G. philippii*
970	*G. pseudoferreum*	KX454409	SH1723042.08FU	*G. philippii*
971	*G. pseudoferreum*	KX454410	SH1723042.08FU	*G. philippii*
972	*G. pseudoferreum*	KX454411	SH1723042.08FU	*G. philippii*
973	*G. pseudoferreum*	KX454412	SH1723042.08FU	*G. philippii*
974	*G. pseudoferreum*	KX454413	SH1723042.08FU	*G. philippii*
975	*G. pseudoferreum*	KX454414	SH1723042.08FU	*G. philippii*
976	*G. pseudoferreum*	KX454415	SH1723042.08FU	*G. philippii*
977	*G. pseudoferreum*	KX454416	SH1723042.08FU	*G. philippii*
978	*G. pseudoferreum*	KX454417	SH1723042.08FU	*G. philippii*
979	*G. pseudoferreum*	KY379054	SH1723042.08FU	*G. philippii*
980	*G. philippii*	MG279166	SH1723042.08FU	*G. philippii*
981	*G. philippii*	MG279167	SH1723042.08FU	*G. philippii*
982	*G. philippii*	MG279188	SH1723042.08FU	*G. philippii*
983	*G. philippii*	MG279189	SH1723042.08FU	*G. philippii*
984	*G. philippii*	MH152513	SH1723042.08FU	*G. philippii*
985	*G. philippii*	MH152514	SH1723042.08FU	*G. philippii*
986	*G. philippii*	MH152515	SH1723042.08FU	*G. philippii*
987	*G. philippii*	MH152516	SH1723042.08FU	*G. philippii*
988	*G. philippii*	MN401410		*G. philippii*
989	*G. philippii*	MN401411	SH1723042.08FU	*G. philippii*
990	*Ganoderma* sp.	AJ536661	SH1723042.08FU	*G. philippii*
991	*Ganoderma* sp.	AJ536663	SH1723042.08FU	*G. philippii*
992	*Ganoderma* sp.	AJ542521	SH1723042.08FU	*G. philippii*
993	*Ganoderma* sp.	AJ543431	SH1723042.08FU	*G. philippii*
994	*Ganoderma* sp.	AJ577748		*G. philippii*
995	*Ganoderma* sp.	AJ608711	SH1723042.08FU	*G. philippii*
996	*Ganoderma* sp.	AJ608712	SH1723042.08FU	*G. philippii*
997	*G. australe*	LC084725	SH1723042.08FU	*G. philippii*
998	*G. australe*	LC084750	SH1723042.08FU	*G. philippii*
999	uncultured soil fungus	MF942546	SH1723042.08FU	*G. philippii*
1000	*Ganoderma* sp.	MK131239	SH1723042.08FU	*G. philippii*
1001	*Ganoderma* sp.	MK131243	SH1723042.08FU	*G. philippii*
1002	*G. lingzhi*	AB811848	SH1723285.08FU	*G. lingzhi*
1003	*G. lingzhi*	JF915393		*G. lingzhi*
1004	*G. lingzhi*	JF915394	SH1723035.08FU	*G. lingzhi*
1005	*G. lingzhi*	JF915395		*G. lingzhi*
1006	*G. lingzhi*	JF915396		*G. lingzhi*

1007	*G. lingzhi*	JF915397	SH1723035.08FU	*G. lingzhi*
1008	*G. lingzhi*	JF915398	SH1723035.08FU	*G. lingzhi*
1009	*G. lingzhi*	JF915399		*G. lingzhi*
1010	*G. lingzhi*	JF915404	SH1723035.08FU	*G. lingzhi*
1011	*G. lingzhi*	JF915405	SH1723035.08FU	*G. lingzhi*
1012	*G. lingzhi*	JF915406	SH1723035.08FU	*G. lingzhi*
1013	*G. lingzhi*	JF915407		*G. lingzhi*
1014	*G. lingzhi*	JF915408		*G. lingzhi*
1015	*G. lingzhi*	JN197282	SH1723035.08FU	*G. lingzhi*
1016	*G. lingzhi*	JN197283		*G. lingzhi*
1017	*G. lingzhi*	JN197284		*G. lingzhi*
1018	*G. lingzhi*	JQ781855	SH1723035.08FU	*G. lingzhi*
1019	*G. lingzhi*	JQ781856	SH1723035.08FU	*G. lingzhi*
1020	*G. lingzhi*	JQ781857	SH1723035.08FU	*G. lingzhi*
1021	*G. lingzhi*	JQ781858	SH1723035.08FU	*G. lingzhi*
1022	*G. lingzhi*	JQ781859	SH1723035.08FU	*G. lingzhi*
1023	*G. lingzhi*	JQ781860	SH1723035.08FU	*G. lingzhi*
1024	*G. lingzhi*	JQ781861	SH1723035.08FU	*G. lingzhi*
1025	*G. lingzhi*	JQ781862	SH1723035.08FU	*G. lingzhi*
1026	*G. lingzhi*	JQ781863	SH1723035.08FU	*G. lingzhi*
1027	*G. lingzhi*	JQ781864	SH1723035.08FU	*G. lingzhi*
1028	*G. lingzhi*	JQ781865	SH1723035.08FU	*G. lingzhi*
1029	*G. lingzhi*	JQ781866	SH1723035.08FU	*G. lingzhi*
1030	*G. lingzhi*	JQ781867	SH1723035.08FU	*G. lingzhi*
1031	*G. lingzhi*	JQ781868	SH1723035.08FU	*G. lingzhi*
1032	*G. lingzhi*	JQ781869	SH1723035.08FU	*G. lingzhi*
1033	*G. lingzhi*	JQ781870	SH1723035.08FU	*G. lingzhi*
1034	*G. lingzhi*	JQ781871	SH1723035.08FU	*G. lingzhi*
1035	*G. lingzhi*	JQ781872	SH1723035.08FU	*G. lingzhi*
1036	*G. lingzhi*	JQ781873	SH1723035.08FU	*G. lingzhi*
1037	*G. lingzhi*	KC222318	SH1723035.08FU	*G. lingzhi*
1038	*G. lingzhi*	KC415761	SH1723035.08FU	*G. lingzhi*
1039	*G. lingzhi*	KC511557	SH1723035.08FU	*G. lingzhi*
1040	*G. lingzhi*	KJ143907	SH1723035.08FU	*G. lingzhi*
1041	*G. lingzhi*	KJ143908	SH1723035.08FU	*G. lingzhi*
1042	*G. lingzhi*	KM249910		*G. lingzhi*
1043	*G. lingzhi*	KM249911		*G. lingzhi*
1044	*G. lingzhi*	KM249912		*G. lingzhi*
1045	*G. lingzhi*	KM249913	SH1723035.08FU	*G. lingzhi*
1046	*G. lingzhi*	KM249914		*G. lingzhi*
1047	*G. lingzhi*	KM249915		*G. lingzhi*
1048	*G. lingzhi*	KM249916	SH1723035.08FU	*G. lingzhi*
1049	*G. lingzhi*	KM249917		*G. lingzhi*
1050	*G. lingzhi*	KM249918		*G. lingzhi*
1051	*G. lingzhi*	KM249919		*G. lingzhi*

1052	*G. lingzhi*	KM249920		*G. lingzhi*
1053	*G. lingzhi*	KM249921	SH1723035.08FU	*G. lingzhi*
1054	*G. lingzhi*	KM249922		*G. lingzhi*
1055	*G. lingzhi*	KM249923	SH1723035.08FU	*G. lingzhi*
1056	*G. lingzhi*	KM249924		*G. lingzhi*
1057	*G. lingzhi*	KM249925		*G. lingzhi*
1058	*G. lingzhi*	KM249926	SH1723035.08FU	*G. lingzhi*
1059	*G. lingzhi*	KM249927		*G. lingzhi*
1060	*G. lingzhi*	KM249928	SH1723035.08FU	*G. lingzhi*
1061	*G. lingzhi*	KM249929	SH1723035.08FU	*G. lingzhi*
1062	*G. lingzhi*	KM249930	SH1723035.08FU	*G. lingzhi*
1063	*G. lingzhi*	KM249931		*G. lingzhi*
1064	*G. lingzhi*	KM249932		*G. lingzhi*
1065	*G. lingzhi*	KR093032	SH1723035.08FU	*G. lingzhi*
1066	*G. lingzhi*	KU219990	SH1723035.08FU	*G. lingzhi*
1067	*G. lingzhi*	KU219991	SH1723035.08FU	*G. lingzhi*
1068	*G. lingzhi*	KU310901	SH1723035.08FU	*G. lingzhi*
1069	*G. lingzhi*	KX055521	SH1723035.08FU	*G. lingzhi*
1070	*G. lingzhi*	KX055522	SH1723035.08FU	*G. lingzhi*
1071	*G. lingzhi*	KX055523	SH1723035.08FU	*G. lingzhi*
1072	*G. lingzhi*	KX055524	SH1723035.08FU	*G. lingzhi*
1073	*G. lingzhi*	KX055525	SH1723035.08FU	*G. lingzhi*
1074	*G. lingzhi*	KX055527	SH1723035.08FU	*G. lingzhi*
1075	*G. lingzhi*	KX055528	SH1723035.08FU	*G. lingzhi*
1076	*G. lingzhi*	KX055531	SH1723035.08FU	*G. lingzhi*
1077	*G. lingzhi*	KX055532	SH1723035.08FU	*G. lingzhi*
1078	*G. lingzhi*	KX055534	SH1723035.08FU	*G. lingzhi*
1079	*G. lingzhi*	KX055536	SH1723035.08FU	*G. lingzhi*
1080	*G. lingzhi*	KX055537	SH1723035.08FU	*G. lingzhi*
1081	*G. lingzhi*	KX055541	SH1723035.08FU	*G. lingzhi*
1082	*G. lingzhi*	KX055546	SH1723035.08FU	*G. lingzhi*
1083	*G. lingzhi*	KX055547	SH1723035.08FU	*G. lingzhi*
1084	*G. lingzhi*	KX055548	SH1723035.08FU	*G. lingzhi*
1085	*G. lingzhi*	KX055562	SH1723035.08FU	*G. lingzhi*
1086	*G. lingzhi*	KX055563	SH1723035.08FU	*G. lingzhi*
1087	*G. lingzhi*	KY364244	SH1723035.08FU	*G. lingzhi*
1088	*G. lingzhi*	KY364245		*G. lingzhi*
1089	*G. lingzhi*	KY364246	SH1723035.08FU	*G. lingzhi*
1090	*G. lingzhi*	KY364247	SH1723035.08FU	*G. lingzhi*
1091	*G. lingzhi*	KY364248	SH1723035.08FU	*G. lingzhi*
1092	*G. lingzhi*	KY364249		*G. lingzhi*
1093	*G. lingzhi*	KY364250	SH1723035.08FU	*G. lingzhi*
1094	*G. lingzhi*	LC090753	SH1723035.08FU	*G. lingzhi*
1095	*G. lingzhi*	MG279180	SH1723035.08FU	*G. lingzhi*
1096	*G. lingzhi*	MG457485	SH1723285.08FU	*G. lingzhi*

1097	*G. lingzhi*	MG732940		*G. lingzhi*
1098	*G. lingzhi*	MG732941		*G. lingzhi*
1099	*G. lingzhi*	MG732942		*G. lingzhi*
1100	*G. lingzhi*	MG732943		*G. lingzhi*
1101	*G. lingzhi*	MG732944		*G. lingzhi*
1102	*G. lingzhi*	MG732945		*G. lingzhi*
1103	*G. lingzhi*	MG732946		*G. lingzhi*
1104	*G. lingzhi*	MG732947		*G. lingzhi*
1105	*G. lingzhi*	MG732948		*G. lingzhi*
1106	*G. lingzhi*	MG732948		*G. lingzhi*
1107	*G. lingzhi*	MG732949		*G. lingzhi*
1108	*G. lingzhi*	MG732949		*G. lingzhi*
1109	*G. lingzhi*	MG732950	SH1723035.08FU	*G. lingzhi*
1110	*G. lingzhi*	MG732951		*G. lingzhi*
1111	*G. lingzhi*	MG732952		*G. lingzhi*
1112	*G. lingzhi*	MG732953		*G. lingzhi*
1113	*G. lingzhi*	MG732954		*G. lingzhi*
1114	*G. lingzhi*	MG732955		*G. lingzhi*
1115	*G. lingzhi*	MG732956		*G. lingzhi*
1116	*G. lingzhi*	MG732957		*G. lingzhi*
1117	*G. lingzhi*	MG732958		*G. lingzhi*
1118	*G. lingzhi*	MG732959		*G. lingzhi*
1119	*G. lingzhi*	MG732960		*G. lingzhi*
1120	*G. lingzhi*	MG732961		*G. lingzhi*
1121	*G. lingzhi*	MG732962		*G. lingzhi*
1122	*G. lingzhi*	MG732963		*G. lingzhi*
1123	*G. lingzhi*	MG732964		*G. lingzhi*
1124	*G. lingzhi*	MG732965	SH1723035.08FU	*G. lingzhi*
1125	*G. lingzhi*	MG732966	SH1723035.08FU	*G. lingzhi*
1126	*G. lingzhi*	MG732967	SH1723035.08FU	*G. lingzhi*
1127	*G. lingzhi*	MG732968	SH1723035.08FU	*G. lingzhi*
1128	*G. lingzhi*	MG732969	SH1723035.08FU	*G. lingzhi*
1129	*G. lingzhi*	MG732970	SH1723035.08FU	*G. lingzhi*
1130	*G. lingzhi*	MG732971		*G. lingzhi*
1131	*G. lingzhi*	MG732972	SH1723035.08FU	*G. lingzhi*
1132	*G. lingzhi*	MG732973	SH1723035.08FU	*G. lingzhi*
1133	*G. lingzhi*	MG732974	SH1723035.08FU	*G. lingzhi*
1134	*G. lingzhi*	MG739456	SH1723035.08FU	*G. lingzhi*
1135	*G. lingzhi*	MG865278	SH1723035.08FU	*G. lingzhi*
1136	*G. lingzhi*	MG865279	SH1723035.08FU	*G. lingzhi*
1137	*G. lingzhi*	MG865280	SH1723035.08FU	*G. lingzhi*
1138	*G. lingzhi*	MG865281	SH1723035.08FU	*G. lingzhi*
1139	*G. lingzhi*	MH109548		*G. lingzhi*
1140	*G. lingzhi*	MH109549		*G. lingzhi*
1141	*G. lingzhi*	MH109550		*G. lingzhi*

1142	*G. lingzhi*	MH109551		*G. lingzhi*
1143	*G. lingzhi*	MH109552		*G. lingzhi*
1144	*G. lingzhi*	MH109553		*G. lingzhi*
1145	*G. lingzhi*	MH109554		*G. lingzhi*
1146	*G. lingzhi*	MH109555		*G. lingzhi*
1147	*G. lingzhi*	MH109556		*G. lingzhi*
1148	*G. lingzhi*	MH109557		*G. lingzhi*
1149	*G. lingzhi*	MH109558		*G. lingzhi*
1150	*G. lingzhi*	MH109559		*G. lingzhi*
1151	*G. lingzhi*	MH109560		*G. lingzhi*
1152	*G. lingzhi*	MH109561		*G. lingzhi*
1153	*G. lingzhi*	MH109562		*G. lingzhi*
1154	*G. lingzhi*	MH109563		*G. lingzhi*
1155	*G. lingzhi*	MH109564		*G. lingzhi*
1156	*G. lingzhi*	MH109565		*G. lingzhi*
1157	*G. lingzhi*	MH109566		*G. lingzhi*
1158	*G. lingzhi*	MH109567		*G. lingzhi*
1159	*G. lingzhi*	MH109568	SH1723035.08FU	*G. lingzhi*
1160	*G. lingzhi*	MH109569		*G. lingzhi*
1161	*G. lingzhi*	MH109570		*G. lingzhi*
1162	*G. lingzhi*	MH109571		*G. lingzhi*
1163	*G. lingzhi*	MH109572		*G. lingzhi*
1164	*G. lingzhi*	MH109573		*G. lingzhi*
1165	*G. lingzhi*	MH109574		*G. lingzhi*
1166	*G. lingzhi*	MH109575		*G. lingzhi*
1167	*G. lingzhi*	MH109576		*G. lingzhi*
1168	*G. lingzhi*	MH109577		*G. lingzhi*
1169	*G. lingzhi*	MH109578		*G. lingzhi*
1170	*G. lingzhi*	MH109579		*G. lingzhi*
1171	*G. lingzhi*	MH109580	SH1723035.08FU	*G. lingzhi*
1172	*G. lingzhi*	MH109581		*G. lingzhi*
1173	*G. lingzhi*	MH109582		*G. lingzhi*
1174	*G. lingzhi*	MH109583		*G. lingzhi*
1175	*G. lingzhi*	MH109584		*G. lingzhi*
1176	*G. lingzhi*	MH109585		*G. lingzhi*
1177	*G. lingzhi*	MH109586		*G. lingzhi*
1178	*G. lingzhi*	MH109587		*G. lingzhi*
1179	*G. lingzhi*	MH109588		*G. lingzhi*
1180	*G. lingzhi*	MH109589		*G. lingzhi*
1181	*G. lingzhi*	MH109590		*G. lingzhi*
1182	*G. lingzhi*	MH109591		*G. lingzhi*
1183	*G. lingzhi*	MH109592		*G. lingzhi*
1184	*G. lingzhi*	MH109593	SH1723035.08FU	*G. lingzhi*
1185	*G. lingzhi*	MH109594		*G. lingzhi*
1186	*G. lingzhi*	MH109595		*G. lingzhi*

1187	*G. lingzhi*	MH109596		*G. lingzhi*
1188	*G. lingzhi*	MH109597		*G. lingzhi*
1189	*G. lingzhi*	MH109598		*G. lingzhi*
1190	*G. lingzhi*	MH109599		*G. lingzhi*
1191	*G. lingzhi*	MH109600		*G. lingzhi*
1192	*G. lingzhi*	MH109601		*G. lingzhi*
1193	*G. lingzhi*	MH109602		*G. lingzhi*
1194	*G. lingzhi*	MH109603		*G. lingzhi*
1195	*G. lingzhi*	MH109604		*G. lingzhi*
1196	*G. lingzhi*	MH109605	SH1723035.08FU	*G. lingzhi*
1197	*G. lingzhi*	MH109606		*G. lingzhi*
1198	*G. lingzhi*	MH109607	SH1723035.08FU	*G. lingzhi*
1199	*G. lingzhi*	MH109608		*G. lingzhi*
1200	*G. lingzhi*	MH109609		*G. lingzhi*
1201	*G. lingzhi*	MH109610		*G. lingzhi*
1202	*G. lingzhi*	MH109611		*G. lingzhi*
1203	*G. lingzhi*	MH109612		*G. lingzhi*
1204	*G. lingzhi*	MH109613		*G. lingzhi*
1205	*G. lingzhi*	MH109614		*G. lingzhi*
1206	*G. lingzhi*	MH109615		*G. lingzhi*
1207	*G. lingzhi*	MH109616		*G. lingzhi*
1208	*G. lingzhi*	MH109617		*G. lingzhi*
1209	*G. lingzhi*	MH109618		*G. lingzhi*
1210	*G. lingzhi*	MH109619		*G. lingzhi*
1211	*G. lingzhi*	MH109620		*G. lingzhi*
1212	*G. lingzhi*	MH109621		*G. lingzhi*
1213	*G. lingzhi*	MH109622		*G. lingzhi*
1214	*G. lingzhi*	MH109623		*G. lingzhi*
1215	*G. lingzhi*	MH109624		*G. lingzhi*
1216	*G. lingzhi*	MH109625		*G. lingzhi*
1217	*G. lingzhi*	MH109626		*G. lingzhi*
1218	*G. lingzhi*	MH109627		*G. lingzhi*
1219	*G. lingzhi*	MH109628		*G. lingzhi*
1220	*G. lingzhi*	MH109629		*G. lingzhi*
1221	*G. lingzhi*	MH109630		*G. lingzhi*
1222	*G. lingzhi*	MH109631	SH1723035.08FU	*G. lingzhi*
1223	*G. lingzhi*	MH109632		*G. lingzhi*
1224	*G. lingzhi*	MH109633		*G. lingzhi*
1225	*G. lingzhi*	MH109634		*G. lingzhi*
1226	*G. lingzhi*	MH109635		*G. lingzhi*
1227	*G. lingzhi*	MH109636	SH1723035.08FU	*G. lingzhi*
1228	*G. lingzhi*	MH109637		*G. lingzhi*
1229	*G. lingzhi*	MH109638		*G. lingzhi*
1230	*G. lingzhi*	MH109639		*G. lingzhi*
1231	*G. lingzhi*	MH109640		*G. lingzhi*

1232	*G. lingzhi*	MH109641		*G. lingzhi*
1233	*G. lingzhi*	MH109642		*G. lingzhi*
1234	*G. lingzhi*	MH109643		*G. lingzhi*
1235	*G. lingzhi*	MH109644		*G. lingzhi*
1236	*G. lingzhi*	MH109645	SH1723035.08FU	*G. lingzhi*
1237	*G. lingzhi*	MH109646		*G. lingzhi*
1238	*G. lingzhi*	MH109647		*G. lingzhi*
1239	*G. lingzhi*	MH109648		*G. lingzhi*
1240	*G. lingzhi*	MH109649		*G. lingzhi*
1241	*G. lingzhi*	MH109650		*G. lingzhi*
1242	*G. lingzhi*	MH109651		*G. lingzhi*
1243	*G. lingzhi*	MH109652		*G. lingzhi*
1244	*G. lingzhi*	MH109653		*G. lingzhi*
1245	*G. lingzhi*	MH109654		*G. lingzhi*
1246	*G. lingzhi*	MH109655		*G. lingzhi*
1247	*G. lingzhi*	MH109656		*G. lingzhi*
1248	*G. lingzhi*	MH109657		*G. lingzhi*
1249	*G. lingzhi*	MH109658		*G. lingzhi*
1250	*G. lingzhi*	MH109659	SH1723035.08FU	*G. lingzhi*
1251	*G. lingzhi*	MH109660		*G. lingzhi*
1252	*G. lingzhi*	MH109661		*G. lingzhi*
1253	*G. lingzhi*	MH109662		*G. lingzhi*
1254	*G. lingzhi*	MH109663		*G. lingzhi*
1255	*G. lingzhi*	MH109664		*G. lingzhi*
1256	*G. lingzhi*	MH109665		*G. lingzhi*
1257	*G. lingzhi*	MH109666		*G. lingzhi*
1258	*G. lingzhi*	MH109667	SH1723035.08FU	*G. lingzhi*
1259	*G. lingzhi*	MH109668		*G. lingzhi*
1260	*G. lingzhi*	MH109669		*G. lingzhi*
1261	*G. lingzhi*	MH109670		*G. lingzhi*
1262	*G. lingzhi*	MH109671	SH1723035.08FU	*G. lingzhi*
1263	*G. lingzhi*	MH109672	SH1723035.08FU	*G. lingzhi*
1264	*G. lingzhi*	MH109673		*G. lingzhi*
1265	*G. lingzhi*	MH109674	SH1723035.08FU	*G. lingzhi*
1266	*G. lingzhi*	MH109675	SH1723035.08FU	*G. lingzhi*
1267	*G. lingzhi*	MH109676	SH1723035.08FU	*G. lingzhi*
1268	*G. lingzhi*	MH109677	SH1723035.08FU	*G. lingzhi*
1269	*G. lingzhi*	MH160058		*G. lingzhi*
1270	*G. lingzhi*	MH160061		*G. lingzhi*
1271	*G. lingzhi*	MH160064		*G. lingzhi*
1272	*G. lingzhi*	MH160065	SH1723035.08FU	*G. lingzhi*
1273	*G. lingzhi*	MH160066	SH1723035.08FU	*G. lingzhi*
1274	*G. lingzhi*	MH160067	SH1723035.08FU	*G. lingzhi*
1275	*G. lingzhi*	MH160070	SH1723035.08FU	*G. lingzhi*
1276	*G. lingzhi*	MH160073	SH1723035.08FU	*G. lingzhi*

1277	*G. lingzhi*	MH160074	SH1723035.08FU	*G. lingzhi*
1278	*G. lingzhi*	MH160075	SH1723035.08FU	*G. lingzhi*
1279	*G. lingzhi*	MH160076	SH1723035.08FU	*G. lingzhi*
1280	*G. lingzhi*	MH160078	SH1723035.08FU	*G. lingzhi*
1281	*G. lingzhi*	MH160079	SH1723035.08FU	*G. lingzhi*
1282	*G. lingzhi*	MH160080	SH1723035.08FU	*G. lingzhi*
1283	*G. lingzhi*	MH160081	SH1723035.08FU	*G. lingzhi*
1284	*G. lingzhi*	MH160082	SH1723035.08FU	*G. lingzhi*
1285	*G. lingzhi*	MH160083	SH1723035.08FU	*G. lingzhi*
1286	*G. lingzhi*	MH160084	SH1723035.08FU	*G. lingzhi*
1287	*G. lingzhi*	MH160085	SH1723035.08FU	*G. lingzhi*
1288	*G. lingzhi*	MH160086	SH1723035.08FU	*G. lingzhi*
1289	*G. lingzhi*	MH294300	SH1723035.08FU	*G. lingzhi*
1290	*G. lingzhi*	MH294301		*G. lingzhi*
1291	*G. lingzhi*	MH294304		*G. lingzhi*
1292	*G. lingzhi*	MH294306		*G. lingzhi*
1293	*G. lingzhi*	MH294307		*G. lingzhi*
1294	*G. lingzhi*	MH294308		*G. lingzhi*
1295	*G. lingzhi*	MH294312	SH1723035.08FU	*G. lingzhi*
1296	*G. lingzhi*	MH294314		*G. lingzhi*
1297	*G. lingzhi*	MH294315		*G. lingzhi*
1298	*G. lingzhi*	MH294317		*G. lingzhi*
1299	*G. lingzhi*	MH294323		*G. lingzhi*
1300	*G. lingzhi*	MH294327		*G. lingzhi*
1301	*G. lingzhi*	MH294328		*G. lingzhi*
1302	*G. lingzhi*	MH294329		*G. lingzhi*
1303	*G. lingzhi*	MH294333		*G. lingzhi*
1304	*G. lingzhi*	MH479057	SH1723285.08FU	*G. lingzhi*
1305	*G. lingzhi*	MK182314	SH1723035.08FU	*G. lingzhi*
1306	*G. lingzhi*	MK268933	SH1723035.08FU	*G. lingzhi*
1307	*G. lingzhi*	MK282240		*G. lingzhi*
1308	*G. lingzhi*	MK345437		*G. lingzhi*
1309	*G. lingzhi*	MK345438		*G. lingzhi*
1310	*G. lingzhi*	MK589272		*G. lingzhi*
1311	*G. lingzhi*	MK589278		*G. lingzhi*
1312	*G. lingzhi*	MK782568	SH1723035.08FU	*G. lingzhi*
1313	*G. lingzhi*	MK809459	SH1723035.08FU	*G. lingzhi*
1314	*G. lingzhi*	MN258634	SH1723035.08FU	*G. lingzhi*
1315	*G. lingzhi*	MN372058		*G. lingzhi*
1316	*G. lingzhi*	MN372059	SH1723035.08FU	*G. lingzhi*
1317	*G. lingzhi*	MN372061		*G. lingzhi*
1318	*G. lingzhi*	MN372062	SH1723035.08FU	*G. lingzhi*
1319	*G. lingzhi*	MN372066	SH1723035.08FU	*G. lingzhi*
1320	*G. lingzhi*	MN372067	SH1723035.08FU	*G. lingzhi*
1321	*G. lingzhi*	MN396319		*G. lingzhi*

1322	*G. lingzhi*	MN396321		*G. lingzhi*
1323	*G. lingzhi*	MN396322		*G. lingzhi*
1324	*G. lingzhi*	MN396323	SH1723035.08FU	*G. lingzhi*
1325	*G. lingzhi*	MN396324	SH1723182.08FU	*G. lingzhi*
1326	*G. lingzhi*	MN396325	SH1723181.08FU	*G. lingzhi*
1327	*G. lingzhi*	MN396326		*G. lingzhi*
1328	*G. lingzhi*	MN396327		*G. lingzhi*
1329	*G. lingzhi*	MN396328	SH1723152.08FU	*G. lingzhi*
1330	*G. lingzhi*	MN396329	SH1723152.08FU	*G. lingzhi*
1331	*G. lingzhi*	MT196416		*G. lingzhi*
1332	*G. lingzhi*	MT510201		*G. lingzhi*
1333	*G. lingzhi*	MT741782		*G. lingzhi*
1334	*G. lucidum*	AB462322	SH1723035.08FU	*G. lingzhi*
1335	*G. lucidum*	AB733122	SH1723035.08FU	*G. lingzhi*
1336	*G. lucidum*	AB733174	SH1723035.08FU	*G. lingzhi*
1337	*G. lucidum*	AB733175		*G. lingzhi*
1338	*G. lucidum*	AF506371	SH1723035.08FU	*G. lingzhi*
1339	*G. boninense*	BD082760		*G. lingzhi*
1340	*G. lucidum*	DQ424969	SH1723035.08FU	*G. lingzhi*
1341	*G. lucidum*	DQ424970		*G. lingzhi*
1342	*G. lucidum*	DQ424971		*G. lingzhi*
1343	*G. lucidum*	DQ424973		*G. lingzhi*
1344	*G. lucidum*	DQ424974	SH1723035.08FU	*G. lingzhi*
1345	*G. lucidum*	DQ424979		*G. lingzhi*
1346	*G. lucidum*	DQ424980		*G. lingzhi*
1347	*G. lucidum*	DQ424981	SH1723035.08FU	*G. lingzhi*
1348	*G. lucidum*	DQ424983		*G. lingzhi*
1349	*G. lucidum*	DQ424984		*G. lingzhi*
1350	*G. lucidum*	DQ424985		*G. lingzhi*
1351	*G. lucidum*	DQ424986	SH1723035.08FU	*G. lingzhi*
1352	*G. lucidum*	DQ424987		*G. lingzhi*
1353	*G. lucidum*	DQ424988	SH1723035.08FU	*G. lingzhi*
1354	*G. lucidum*	DQ424989		*G. lingzhi*
1355	*G. lucidum*	DQ424991		*G. lingzhi*
1356	*G. lucidum*	DQ424992		*G. lingzhi*
1357	*G. lucidum*	DQ424993	SH1723035.08FU	*G. lingzhi*
1358	*G. lucidum*	DQ424994	SH1723035.08FU	*G. lingzhi*
1359	*G. lucidum*	DQ424997		*G. lingzhi*
1360	*G. tsugae*	DQ425001		*G. lingzhi*
1361	*G. lucidum*	DQ425004	SH1723035.08FU	*G. lingzhi*
1362	*G. lucidum*	DQ425007		*G. lingzhi*
1363	*G. lucidum*	DQ425008	SH1723035.08FU	*G. lingzhi*
1364	*G. lucidum*	DQ425012	SH1723035.08FU	*G. lingzhi*
1365	*G. lucidum*	DQ425013	SH1723035.08FU	*G. lingzhi*
1366	*G. lucidum*	DQ425015	SH1723035.08FU	*G. lingzhi*

1367	*G. lucidum*	EF188277	SH1723035.08FU	*G. lingzhi*
1368	*G. lucidum*	EF188278	SH1723035.08FU	*G. lingzhi*
1369	*G. lucidum*	EF188279	SH1723035.08FU	*G. lingzhi*
1370	*G. lucidum*	EF188280	SH1723035.08FU	*G. lingzhi*
1371	*G. lucidum*	EU021455	SH1723035.08FU	*G. lingzhi*
1372	*G. lucidum*	EU021456	SH1723035.08FU	*G. lingzhi*
1373	*G. lucidum*	EU498091	SH1723035.08FU	*G. lingzhi*
1374	*G. lucidum*	EU520155		*G. lingzhi*
1375	*G. lucidum*	EU520235		*G. lingzhi*
1376	*G. lucidum*	EU520247		*G. lingzhi*
1377	*G. lucidum*	FJ379262	SH1723035.08FU	*G. lingzhi*
1378	*G. lucidum*	FJ379263	SH1723035.08FU	*G. lingzhi*
1379	*G. lucidum*	FJ379265	SH1723035.08FU	*G. lingzhi*
1380	*G. lucidum*	FJ463904		*G. lingzhi*
1381	*G. lucidum*	FJ463907		*G. lingzhi*
1382	*G. lucidum*	FJ463909		*G. lingzhi*
1383	*G. lucidum*	FJ463910		*G. lingzhi*
1384	*G. lucidum*	FJ463912		*G. lingzhi*
1385	*G. lucidum*	FJ463919	SH1723037.08FU	*G. lingzhi*
1386	*G. lucidum*	FJ463921		*G. lingzhi*
1387	*G. lucidum*	FJ463929		*G. lingzhi*
1388	*G. lucidum*	FJ463931	SH1723035.08FU	*G. lingzhi*
1389	*G. lucidum*	FJ501553	SH1723035.08FU	*G. lingzhi*
1390	*G. lucidum*	FJ501554		*G. lingzhi*
1391	*G. lucidum*	FJ501555		*G. lingzhi*
1392	*G. lucidum*	FJ501557	SH1723037.08FU	*G. lingzhi*
1393	*G. lucidum*	FJ501559	SH1723035.08FU	*G. lingzhi*
1394	*G. lucidum*	FJ501568	SH1723035.08FU	*G. lingzhi*
1395	*G. lucidum*	FJ501569		*G. lingzhi*
1396	*G. lucidum*	FJ501576	SH1723035.08FU	*G. lingzhi*
1397	*G. lucidum*	FJ501577	SH1723035.08FU	*G. lingzhi*
1398	*G. lucidum*	FJ687271	SH1723035.08FU	*G. lingzhi*
1399	*G. lucidum*	FJ940919	SH1723035.08FU	*G. lingzhi*
1400	*G. lucidum*	GU213471	SH1723035.08FU	*G. lingzhi*
1401	*G. lucidum*	GU213476		*G. lingzhi*
1402	*G. lucidum*	GU213477		*G. lingzhi*
1403	*G. lucidum*	GU213478	SH1723035.08FU	*G. lingzhi*
1404	*G. lucidum*	GU213479	SH1723035.08FU	*G. lingzhi*
1405	*G. lucidum*	GU213480	SH1723035.08FU	*G. lingzhi*
1406	*G. lucidum*	GU213481	SH1723035.08FU	*G. lingzhi*
1407	*G. lucidum*	GU213483	SH1723035.08FU	*G. lingzhi*
1408	*G. lucidum*	GU213484	SH1723035.08FU	*G. lingzhi*
1409	*G. lucidum*	GU213485	SH1723035.08FU	*G. lingzhi*
1410	*G. lucidum*	GU213487	SH1723035.08FU	*G. lingzhi*
1411	*G. lucidum*	HQ222603	SH1723273.08FU	*G. lingzhi*

1412	*G. lucidum*	HQ222604	SH1723223.08FU	*G. lingzhi*
1413	*G. lucidum*	HQ235630		*G. lingzhi*
1414	*G. lucidum*	HQ235631	SH1723035.08FU	*G. lingzhi*
1415	*G. lucidum*	HQ235632	SH1723035.08FU	*G. lingzhi*
1416	*Ganoderma* sp.	HQ689695	SH1723035.08FU	*G. lingzhi*
1417	*Ganoderma* sp.	HQ689696	SH1723035.08FU	*G. lingzhi*
1418	*Ganoderma* sp.	HQ689697	SH1723035.08FU	*G. lingzhi*
1419	*G. sichuanense*	JF915400	SH1723035.08FU	*G. lingzhi*
1420	*G. sichuanense*	JF915401	SH1723035.08FU	*G. lingzhi*
1421	*G. sichuanense*	JF915402	SH1723035.08FU	*G. lingzhi*
1422	*G. sichuanense*	JF915403		*G. lingzhi*
1423	*G. lucidum*	JN008869		*G. lingzhi*
1424	*G. lucidum*	JN008870		*G. lingzhi*
1425	*G. lucidum*	JN008871	SH1723035.08FU	*G. lingzhi*
1426	*G. lucidum*	JN008872		*G. lingzhi*
1427	*G. lucidum*	JN048774	SH1723035.08FU	*G. lingzhi*
1428	*G. sichuanense*	JN197281		*G. lingzhi*
1429	*G. lucidum*	JN222405		*G. lingzhi*
1430	*G. lucidum*	JN222421	SH1723035.08FU	*G. lingzhi*
1431	*G. lucidum*	JN222423	SH1723035.08FU	*G. lingzhi*
1432	*G. lucidum*	JN222424	SH1723035.08FU	*G. lingzhi*
1433	*G. lucidum*	JN222425	SH1723035.08FU	*G. lingzhi*
1434	*G. lucidum*	JN222426		*G. lingzhi*
1435	*G. lucidum*	JQ520167	SH1723035.08FU	*G. lingzhi*
1436	*G. lucidum*	JQ520168		*G. lingzhi*
1437	*G. lucidum*	JQ520169		*G. lingzhi*
1438	*G. lucidum*	JQ520170	SH1723035.08FU	*G. lingzhi*
1439	*G. lucidum*	JQ520171		*G. lingzhi*
1440	*G. lucidum*	JQ520172		*G. lingzhi*
1441	*G. lucidum*	JQ520173		*G. lingzhi*
1442	*G. lucidum*	JQ520174	SH1723035.08FU	*G. lingzhi*
1443	*G. lucidum*	JQ520175	SH1723035.08FU	*G. lingzhi*
1444	*G. lucidum*	JQ520176	SH1723035.08FU	*G. lingzhi*
1445	*G. lucidum*	JQ520177	SH1723035.08FU	*G. lingzhi*
1446	*G. lucidum*	JQ520178	SH1723035.08FU	*G. lingzhi*
1447	*G. lucidum*	JQ520179	SH1723035.08FU	*G. lingzhi*
1448	*G. lucidum*	JQ520180	SH1723035.08FU	*G. lingzhi*
1449	*G. lucidum*	JQ520181	SH1723035.08FU	*G. lingzhi*
1450	*G. lucidum*	JQ520182		*G. lingzhi*
1451	*G. lucidum*	JQ520183		*G. lingzhi*
1452	*G. lucidum*	JQ520184	SH1723035.08FU	*G. lingzhi*
1453	*G. lucidum*	JQ520188		*G. lingzhi*
1454	*G. lucidum*	JQ520189		*G. lingzhi*
1455	*Ganoderma* sp.	JQ520207		*G. lingzhi*
1456	*Ganoderma* sp.	JQ520210	SH1723035.08FU	*G. lingzhi*

1457	*Ganoderma* sp.	JQ520211		*G. lingzhi*
1458	*Ganoderma* sp.	JQ520214	SH1723035.08FU	*G. lingzhi*
1459	*G. lucidum*	JQ627589		*G. lingzhi*
1460	*G. lucidum*	JQ627590		*G. lingzhi*
1461	*G. lucidum*	JX162754	SH1723035.08FU	*G. lingzhi*
1462	*G. lucidum*	JX162755		*G. lingzhi*
1463	*G. lucidum*	JX162756	SH1723035.08FU	*G. lingzhi*
1464	*G. lucidum*	JX162757	SH1723035.08FU	*G. lingzhi*
1465	*G. lucidum*	JX162758		*G. lingzhi*
1466	*G. lucidum*	JX162759	SH1723035.08FU	*G. lingzhi*
1467	*G. lucidum*	JX162760		*G. lingzhi*
1468	*G. lucidum*	JX162761		*G. lingzhi*
1469	*G. lucidum*	JX162762	SH1723035.08FU	*G. lingzhi*
1470	*G. lucidum*	JX162763	SH1723035.08FU	*G. lingzhi*
1471	*G. lucidum*	JX162764	SH1723035.08FU	*G. lingzhi*
1472	*G. lucidum*	JX162765		*G. lingzhi*
1473	*G. lucidum*	JX162766	SH1723035.08FU	*G. lingzhi*
1474	*G. lucidum*	JX162767	SH1723035.08FU	*G. lingzhi*
1475	*G. lucidum*	JX162768		*G. lingzhi*
1476	*G. lucidum*	JX162770		*G. lingzhi*
1477	*G. lucidum*	KC311368		*G. lingzhi*
1478	*G. lucidum*	KC311370		*G. lingzhi*
1479	*G. lucidum*	KC311371		*G. lingzhi*
1480	*G. lucidum*	KC414242	SH1723035.08FU	*G. lingzhi*
1481	*G. sichuanense*	KC505544	SH1723035.08FU	*G. lingzhi*
1482	*G. sichuanense*	KC662402/ NR_152892	SH1723035.08FU	*G. lingzhi*
1483	*G. lucidum*	KF146177	SH1723035.08FU	*G. lingzhi*
1484	*G. lucidum*	KM269294	SH1723035.08FU	*G. lingzhi*
1485	*G. luteomarginatum*	KP226861	SH1723035.08FU	*G. lingzhi*
1486	*Haddowia longipes*	KP226862	SH1723035.08FU	*G. lingzhi*
1487	*Ganoderma* sp.	KT185665		*G. lingzhi*
1488	*G. sichuanense*	KT318600	SH1723035.08FU	*G. lingzhi*
1489	*G. sichuanense*	KT318601	SH1723035.08FU	*G. lingzhi*
1490	*G. sichuanense*	KT318602	SH1723035.08FU	*G. lingzhi*
1491	*G. sichuanense*	KT318603	SH1723035.08FU	*G. lingzhi*
1492	*G. lucidum*	KT343299	SH1723035.08FU	*G. lingzhi*
1493	*G. sichuanense*	KT693248	SH1723035.08FU	*G. lingzhi*
1494	*G. sichuanense*	KT693249	SH1723035.08FU	*G. lingzhi*
1495	*G. sichuanense*	KT693250	SH1723035.08FU	*G. lingzhi*
1496	*G. sichuanense*	KT693251	SH1723035.08FU	*G. lingzhi*
1497	*G. sichuanense*	KT693252	SH1723035.08FU	*G. lingzhi*
1498	*G. sichuanense*	KT693253	SH1723035.08FU	*G. lingzhi*
1499	*G. sichuanense*	KT693254	SH1723035.08FU	*G. lingzhi*
1500	*G. sichuanense*	KT693255	SH1723035.08FU	*G. lingzhi*
1501	*G. lucidum*	KT717953	SH1723035.08FU	*G. lingzhi*

1502	*G. lucidum*	KT906367	SH1723035.08FU	*G. lingzhi*
1503	*G. lucidum*	KT906368	SH1723035.08FU	*G. lingzhi*
1504	*G. lucidum*	KT921216	SH1723035.08FU	*G. lingzhi*
1505	*G. lucidum*	KU863089	SH1723035.08FU	*G. lingzhi*
1506	*G. lucidum*	KX055533	SH1723035.08FU	*G. lingzhi*
1507	*G. lucidum*	KX055535	SH1723035.08FU	*G. lingzhi*
1508	*G. lucidum*	KX055538	SH1723035.08FU	*G. lingzhi*
1509	*G. lucidum*	KX055539	SH1723035.08FU	*G. lingzhi*
1510	*G. lucidum*	KX055543	SH1723035.08FU	*G. lingzhi*
1511	*G. lucidum*	KX055544	SH1723035.08FU	*G. lingzhi*
1512	*G. lucidum*	KX055545	SH1723035.08FU	*G. lingzhi*
1513	*G. lucidum*	KX055550	SH1723035.08FU	*G. lingzhi*
1514	*G. lucidum*	KX055551	SH1723035.08FU	*G. lingzhi*
1515	*G. lucidum*	KX055553	SH1723035.08FU	*G. lingzhi*
1516	*G. cupreum*	KX055555	SH1723035.08FU	*G. lingzhi*
1517	*G. lucidum*	KX262896	SH1723035.08FU	*G. lingzhi*
1518	*G. lucidum*	KX262897	SH1723035.08FU	*G. lingzhi*
1519	*G. lucidum*	KX262898	SH1723035.08FU	*G. lingzhi*
1520	*G. lucidum*	KX262899	SH1723035.08FU	*G. lingzhi*
1521	*G. lucidum*	KX262902	SH1723035.08FU	*G. lingzhi*
1522	*G. lucidum*	KX262904	SH1723035.08FU	*G. lingzhi*
1523	*G. lucidum*	KX262905	SH1723035.08FU	*G. lingzhi*
1524	*G. lucidum*	KX262906	SH1723035.08FU	*G. lingzhi*
1525	*G. lucidum*	KX262907	SH1723035.08FU	*G. lingzhi*
1526	*G. lucidum*	KX358402	SH1723035.08FU	*G. lingzhi*
1527	*G. lucidum*	KX358403	SH1723035.08FU	*G. lingzhi*
1528	*G. lucidum*	KX580183	SH1723206.08FU	*G. lingzhi*
1529	*G. lucidum*	KX589244	SH1723035.08FU	*G. lingzhi*
1530	*G. lucidum*	KX589245	SH1723035.08FU	*G. lingzhi*
1531	*G. lucidum*	KX589246	SH1723035.08FU	*G. lingzhi*
1532	*G. lucidum*	KX589247	SH1723035.08FU	*G. lingzhi*
1533	*G. lucidum*	KX589248	SH1723035.08FU	*G. lingzhi*
1534	*G. lucidum*	KX589249	SH1723035.08FU	*G. lingzhi*
1535	*G. lucidum*	KX589250	SH1723035.08FU	*G. lingzhi*
1536	*G. sichuanense*	KY244061	SH1723035.08FU	*G. lingzhi*
1537	*G. sichuanense*	KY244062	SH1723035.08FU	*G. lingzhi*
1538	*G. sichuanense*	KY244063	SH1723035.08FU	*G. lingzhi*
1539	*G. sichuanense*	KY244064	SH1723035.08FU	*G. lingzhi*
1540	*G. sichuanense*	KY244065	SH1723035.08FU	*G. lingzhi*
1541	*G. sichuanense*	KY244066	SH1723035.08FU	*G. lingzhi*
1542	*G. sichuanense*	KY244068	SH1723035.08FU	*G. lingzhi*
1543	*G. sichuanense*	KY404119	SH1723035.08FU	*G. lingzhi*
1544	*G. calidophilum*	KY612892	SH1723035.08FU	*G. lingzhi*
1545	*G. lucidum*	LC068794	SH1723035.08FU	*G. lingzhi*
1546	*G. lucidum*	LC269925		*G. lingzhi*

1547	*G. lucidum*	MF476197	SH1723035.08FU	*G. lingzhi*
1548	*G. lucidum*	MF476198	SH1723035.08FU	*G. lingzhi*
1549	*G. lucidum*	MF476199	SH1723035.08FU	*G. lingzhi*
1550	*G. lucidum*	MF476200	SH1723035.08FU	*G. lingzhi*
1551	*G. lucidum*	MF476201	SH1723035.08FU	*G. lingzhi*
1552	*G. lucidum*	MF770158	SH1723035.08FU	*G. lingzhi*
1553	*G. tsugae*	MG706221	SH1723035.08FU	*G. lingzhi*
1554	*G. lucidum*	MG706222	SH1723035.08FU	*G. lingzhi*
1555	*G. lucidum*	MH018022	SH1723035.08FU	*G. lingzhi*
1556	*G. lucidum*	MH018023	SH1723035.08FU	*G. lingzhi*
1557	*G. lucidum*	MH018024	SH1723035.08FU	*G. lingzhi*
1558	*G. lucidum*	MH018026	SH1723035.08FU	*G. lingzhi*
1559	*G. lucidum*	MH018027	SH1723035.08FU	*G. lingzhi*
1560	*G. lucidum*	MH018028	SH1723035.08FU	*G. lingzhi*
1561	*G. lucidum*	MH018029	SH1723035.08FU	*G. lingzhi*
1562	*G. lucidum*	MH018030	SH1723035.08FU	*G. lingzhi*
1563	*G. lucidum*	MH018031	SH1723035.08FU	*G. lingzhi*
1564	*G. lucidum*	MH042929	SH1723035.08FU	*G. lingzhi*
1565	*Ganoderma* sp.	MH294305		*G. lingzhi*
1566	*Ganoderma* sp.	MH294320		*G. lingzhi*
1567	*Ganoderma* sp.	MH294322		*G. lingzhi*
1568	*Ganoderma* sp.	MH294324		*G. lingzhi*
1569	*Ganoderma* sp.	MH294325		*G. lingzhi*
1570	*G. lucidum*	MK209603	SH1723035.08FU	*G. lingzhi*
1571	*G. lucidum*	MK370666	SH1723035.08FU	*G. lingzhi*
1572	*G. lucidum*	MK603977		*G. lingzhi*
1573	*G. lucidum*	MK773880	SH2758630.08FU	*G. lingzhi*
1574	*G. lucidum*	MK855511	SH1723035.08FU	*G. lingzhi*
1575	*G. lucidum*	MN294898		*G. lingzhi*
1576	*G. lucidum*	MN372060	SH1723035.08FU	*G. lingzhi*
1577	*Ganoderma* sp.	MN396330	SH1723285.08FU	*G. lingzhi*
1578	*G. lucidum*	MN431189		*G. lingzhi*
1579	*G. lucidum*	MN596944		*G. lingzhi*
1580	*Laccaria bicolor*	MN622726		*G. lingzhi*
1581	*G. lucidum*	MN636776		*G. lingzhi*
1582	*G. lucidum*	MN888753		*G. lingzhi*
1583	*Ganoderma* sp.	MN911326		*G. lingzhi*
1584	*Ganoderma* sp.	MN911327		*G. lingzhi*
1585	*Ganoderma* sp.	MN911328		*G. lingzhi*
1586	*Ganoderma* sp.	MN911329		*G. lingzhi*
1587	*Ganoderma* sp.	MN911330		*G. lingzhi*
1588	*Ganoderma* sp.	MN911331		*G. lingzhi*
1589	*Ganoderma* sp.	MN911332		*G. lingzhi*
1590	*Ganoderma* sp.	MN911333		*G. lingzhi*
1591	*Ganoderma* sp.	MN911334		*G. lingzhi*

1592	*Ganoderma* sp.	MN911335		*G. lingzhi*
1593	*Ganoderma* sp.	MN911337		*G. lingzhi*
1594	*Ganoderma* sp.	MN911338		*G. lingzhi*
1595	*Ganoderma* sp.	MN911339		*G. lingzhi*
1596	*Ganoderma* sp.	MN911340		*G. lingzhi*
1597	*Ganoderma* sp.	MN911341		*G. lingzhi*
1598	*Ganoderma* sp.	MN911342		*G. lingzhi*
1599	*Ganoderma* sp.	MN911343		*G. lingzhi*
1600	*Ganoderma* sp.	MN911344		*G. lingzhi*
1601	*Ganoderma* sp.	MN911345		*G. lingzhi*
1602	*Ganoderma* sp.	MN911346		*G. lingzhi*
1603	*Ganoderma* sp.	MN911347		*G. lingzhi*
1604	*G. lucidum*	MT879644		*G. lingzhi*
1605	*Ganoderma* sp.	UDB033504	SH1723035.08FU	*G. lingzhi*
1606	*Ganoderma* sp.	UDB034543	SH1723035.08FU	*G. lingzhi*
1607	*Amauroderma rugosum*	UDB038354	SH1723035.08FU	*G. lingzhi*
1608	*G. tsugae*	X78746/X78767		*G. lingzhi*
1609	*G. tsugae*	X78747/X78768		*G. lingzhi*
1610	*G. tsugae*	X87352/X87362		*G. lingzhi*
1611	*G. lucidum*	X87354/X87364		*G. lingzhi*
1612	*G. lucidum*	Z37050/Z37074		*G. lingzhi*
1613	*G. lucidum*	Z37097/Z37078		*G. lingzhi*
1614	*G. lingzhi*	KT247417	SH1847826.08FU	*G. lingzhi*
1615	*G. lucidum*	EU520137	SH1740453.08FU	*G. lingzhi*
1616	*G. lucidum*	FJ381957		*G. lingzhi*
1617	*G. curtisii*	JQ520164	SH1723039.08FU	*G. curtisii*
1618	*G. meredithae*	JQ520190	SH1723039.08FU	*G. curtisii*
1619	*G. meredithae*	JQ520191	SH1723039.08FU	*G. curtisii*
1620	*G. curtisii*	JQ781848	SH1723039.08FU	*G. curtisii*
1621	*G. curtisii*	JQ781849	SH1723039.08FU	*G. curtisii*
1622	*G. curtisii*	KF605641	SH1723039.08FU	*G. curtisii*
1623	*G. curtisii*	KF605642	SH1723039.08FU	*G. curtisii*
1624	*G. curtisii*	KF605643		*G. curtisii*
1625	*G. curtisii*	KF605644	SH1723039.08FU	*G. curtisii*
1626	*G. curtisii*	KF605646	SH1723039.08FU	*G. curtisii*
1627	*G. curtisii*	KF963259	SH1723039.08FU	*G. curtisii*
1628	*G. curtisii*	KY646217	SH1723039.08FU	*G. curtisii*
1629	*G. curtisii*	KY646218	SH1723039.08FU	*G. curtisii*
1630	*G. meredithae*	KY646219		*G. curtisii*
1631	*G. meredithae*	KY646220	SH1723039.08FU	*G. curtisii*
1632	*G. curtisii*	KY708878	SH1723039.08FU	*G. curtisii*
1633	*G. curtisii*	KY708879	SH1723039.08FU	*G. curtisii*
1634	*G. curtisii*	KY708880	SH1723039.08FU	*G. curtisii*
1635	*G. meredithae*	KY708881	SH1723039.08FU	*G. curtisii*
1636	*G. curtisii*	KY767033	SH1723039.08FU	*G. curtisii*

1637	*G. curtisii*	MF773591	SH1723039.08FU	*G. curtisii*
1638	*G. curtisii*	MG654074		*G. curtisii*
1639	*G. curtisii*	MG654075	SH1723039.08FU	*G. curtisii*
1640	*G. curtisii*	MG654076	SH1723039.08FU	*G. curtisii*
1641	*G. curtisii*	MG654077	SH1723039.08FU	*G. curtisii*
1642	*G. curtisii*	MG654078	SH1723039.08FU	*G. curtisii*
1643	*G. curtisii*	MG654079	SH1723039.08FU	*G. curtisii*
1644	*G. curtisii*	MG654080	SH1723039.08FU	*G. curtisii*
1645	*G. curtisii*	MG654081	SH1723039.08FU	*G. curtisii*
1646	*G. curtisii*	MG654082	SH1723039.08FU	*G. curtisii*
1647	*G. curtisii*	MG654083	SH1723039.08FU	*G. curtisii*
1648	*G. curtisii*	MG654084	SH1723039.08FU	*G. curtisii*
1649	*G. curtisii*	MG654085	SH1723039.08FU	*G. curtisii*
1650	*G. curtisii*	MG654086	SH1723039.08FU	*G. curtisii*
1651	*G. curtisii*	MG654087	SH1723039.08FU	*G. curtisii*
1652	*G. curtisii*	MG654088		*G. curtisii*
1653	*G. curtisii*	MG654089	SH1723039.08FU	*G. curtisii*
1654	*G. curtisii*	MG654090		*G. curtisii*
1655	*G. curtisii*	MG654091	SH1723039.08FU	*G. curtisii*
1656	*G. curtisii*	MG654092		*G. curtisii*
1657	*G. curtisii*	MG654093		*G. curtisii*
1658	*G. curtisii*	MG654094		*G. curtisii*
1659	*G. curtisii*	MG654095		*G. curtisii*
1660	*G. curtisii*	MG654096		*G. curtisii*
1661	*G. curtisii*	MG654097		*G. curtisii*
1662	*G. curtisii*	MG654098	SH1723039.08FU	*G. curtisii*
1663	*G. curtisii*	MG654099	SH1723039.08FU	*G. curtisii*
1664	*G. curtisii*	MG654100		*G. curtisii*
1665	*G. curtisii*	MG654101	SH1723039.08FU	*G. curtisii*
1666	*G. curtisii*	MG654102		*G. curtisii*
1667	*G. meredithae*	MG654103		*G. curtisii*
1668	*G. curtisii*	MG654104	SH1723039.08FU	*G. curtisii*
1669	*G. curtisii*	MG654105	SH1723039.08FU	*G. curtisii*
1670	*G. curtisii* f. sp. *meredithae*	MG654106	SH1723039.08FU	*G. curtisii*
1671	*G. curtisii*	MG654107		*G. curtisii*
1672	*G. curtisii*	MG654108		*G. curtisii*
1673	*G. curtisii*	MG654109	SH1723039.08FU	*G. curtisii*
1674	*G. meredithae*	MG654110	SH1723039.08FU	*G. curtisii*
1675	*G. curtisii*	MG654111		*G. curtisii*
1676	*G. curtisii*	MG654112		*G. curtisii*
1677	*G. curtisii*	MG654113	SH1723039.08FU	*G. curtisii*
1678	*G. curtisii*	MG654114	SH1723039.08FU	*G. curtisii*
1679	*G. curtisii*	MG654115	SH1723039.08FU	*G. curtisii*
1680	*G. curtisii*	MG654116	SH1723039.08FU	*G. curtisii*
1681	*G. curtisii*	MG654117	SH1723039.08FU	*G. curtisii*

1682	*G. curtisii*	MG654118	SH1723039.08FU	*G. curtisii*
1683	*G. curtisii*	MG654119	SH1723039.08FU	*G. curtisii*
1684	*G. curtisii*	MG654120		*G. curtisii*
1685	*G. curtisii*	MG654121		*G. curtisii*
1686	*G. curtisii*	MG654122		*G. curtisii*
1687	*G. curtisii*	MG654123		*G. curtisii*
1688	*G. curtisii*	MG654124		*G. curtisii*
1689	*G. curtisii*	MG654125		*G. curtisii*
1690	*G. curtisii*	MG654126		*G. curtisii*
1691	*G. curtisii*	MG654127		*G. curtisii*
1692	*G. curtisii*	MG654128	SH1723039.08FU	*G. curtisii*
1693	*G. curtisii*	MG654129	SH1723039.08FU	*G. curtisii*
1694	*G. curtisii*	MG654130	SH1723039.08FU	*G. curtisii*
1695	*G. curtisii*	MG654131		*G. curtisii*
1696	*G. curtisii*	MG654132		*G. curtisii*
1697	*G. curtisii*	MG654133	SH1723039.08FU	*G. curtisii*
1698	*G. curtisii*	MG654134	SH1723039.08FU	*G. curtisii*
1699	*G. curtisii*	MG654135	SH1723039.08FU	*G. curtisii*
1700	*G. curtisii*	MG654136	SH1723039.08FU	*G. curtisii*
1701	*G. curtisii*	MG654137		*G. curtisii*
1702	*G. curtisii*	MG654138	SH1723039.08FU	*G. curtisii*
1703	*G. curtisii*	MG654139	SH1723039.08FU	*G. curtisii*
1704	*G. curtisii*	MG654140		*G. curtisii*
1705	*G. curtisii*	MG654141		*G. curtisii*
1706	*G. curtisii*	MG654142	SH1723039.08FU	*G. curtisii*
1707	*G. curtisii*	MG654143	SH1723039.08FU	*G. curtisii*
1708	*G. curtisii*	MG654144	SH1723039.08FU	*G. curtisii*
1709	*G. curtisii*	MG654145	SH1723039.08FU	*G. curtisii*
1710	*G. curtisii*	MG654146		*G. curtisii*
1711	*G. curtisii*	MG654147		*G. curtisii*
1712	*G. curtisii*	MG654148	SH1723039.08FU	*G. curtisii*
1713	*G. curtisii*	MG654149	SH1723039.08FU	*G. curtisii*
1714	*G. curtisii*	MG654150	SH1723039.08FU	*G. curtisii*
1715	*G. curtisii*	MG654151	SH1723039.08FU	*G. curtisii*
1716	*G. curtisii*	MG654153	SH1723039.08FU	*G. curtisii*
1717	*G. curtisii*	MG654154	SH1723039.08FU	*G. curtisii*
1718	*G. curtisii*	MG654155	SH1723039.08FU	*G. curtisii*
1719	*G. curtisii*	MG654156	SH1723039.08FU	*G. curtisii*
1720	*G. curtisii*	MG654157	SH1723039.08FU	*G. curtisii*
1721	*G. curtisii*	MG654158	SH1723039.08FU	*G. curtisii*
1722	*G. curtisii*	MG654159	SH1723039.08FU	*G. curtisii*
1723	*G. curtisii*	MG654160	SH1723039.08FU	*G. curtisii*
1724	*G. curtisii*	MG654161	SH1723039.08FU	*G. curtisii*
1725	*G. curtisii*	MG654162	SH1723039.08FU	*G. curtisii*
1726	*G. curtisii*	MG654163	SH1723039.08FU	*G. curtisii*

1727	*G. curtisii*	MG654164	SH1723039.08FU	*G. curtisii*
1728	*G. curtisii*	MG654165	SH1723039.08FU	*G. curtisii*
1729	*G. curtisii*	MG654166	SH1723039.08FU	*G. curtisii*
1730	*G. curtisii*	MG654167	SH1723039.08FU	*G. curtisii*
1731	*G. curtisii*	MG654168	SH1723039.08FU	*G. curtisii*
1732	*G. curtisii*	MG654169	SH1723039.08FU	*G. curtisii*
1733	*G. curtisii*	MG654170	SH1723039.08FU	*G. curtisii*
1734	*G. curtisii*	MG654171	SH1723039.08FU	*G. curtisii*
1735	*G. curtisii*	MG654172	SH1723039.08FU	*G. curtisii*
1736	*G. meredithae*	MG654187	SH1723039.08FU	*G. curtisii*
1737	*G. curtisii* f. sp. *meredithae*	MG654188		*G. curtisii*
1738	*G. curtisii*	MG966318	SH1723039.08FU	*G. curtisii*
1739	*G. curtisii*	MH160063	SH1723039.08FU	*G. curtisii*
1740	*G. curtisii*	MH160072	SH1723039.08FU	*G. curtisii*
1741	*G. curtisii*	MH165297	SH1723039.08FU	*G. curtisii*
1742	*G. meredithae*	MH289783	SH1723039.08FU	*G. curtisii*
1743	*G. curtisii*	MH347284	SH1723039.08FU	*G. curtisii*
1744	*G. meredithae*	MH862131/ NR_164435	SH1723039.08FU	*G. curtisii*
1745	*G. curtisii*	MH910547	SH1723039.08FU	*G. curtisii*
1746	*G. curtisii*	MK370946	SH1723039.08FU	*G. curtisii*
1747	*G. meredithae*	MK532860	SH1723039.08FU	*G. curtisii*
1748	*G. curtisii*	MT254976		*G. curtisii*
1749	*G. curtisii*	MT254977		*G. curtisii*
1750	*G. curtisii*	MT254978		*G. curtisii*
1751	*G. curtisii*	MT254979		*G. curtisii*
1752	*G. curtisii*	MT254980		*G. curtisii*
1753	*G. lucidum*	JX270802	SH1723039.08FU	*G. curtisii*
1754	*G. lucidum*	JX270803	SH1723039.08FU	*G. curtisii*
1755	*Ganoderma* sp.	KT223757	SH1723039.08FU	*G. curtisii*
1756	*G. lucidum*	LN998989	SH1723039.08FU	*G. curtisii*
1757	*Ganoderma* sp.	MH211682	SH1723039.08FU	*G. curtisii*
1758	*G. curtisii*	MT232640		*G. curtisii*
1759	*G. ravenelli*	MG654206	SH1723039.08FU	*G. ravenelli*
1760	*G. ravenelli*	MG654207	SH1723039.08FU	*G. ravenelli*
1761	*G. ravenelli*	MG654208	SH1723039.08FU	*G. ravenelli*
1762	*G. ravenelli*	MG654209		*G. ravenelli*
1763	*G. ravenelli*	MG654210	SH1723039.08FU	*G. ravenelli*
1764	*G. ravenelli*	MG654211	SH1723039.08FU	*G. ravenelli*
1765	*G. lucidum*	AY456341	SH1723039.08FU	*G. ravenelli*
1766	*G. lucidum*	FJ463928	SH1723039.08FU	*G. ravenelli*
1767	*G. curtisii*	KF605645	SH1723039.08FU	*G. ravenelli*
1768	uncultured fungus	KM103994	SH1723039.08FU	*G. ravenelli*
1769	*G. curtisii*	MG654152	SH1723039.08FU	*G. ravenelli*
1770	*G. curtisii*	MG654173	SH1723039.08FU	*G. ravenelli*

1771	*G. multiplicatum*	JX310823	SH1723118.08FU	*G. multiplicatum*
1772	*G. multiplicatum*	KU569515	SH1720237.08FU	*G. multiplicatum*
1773	*G. multiplicatum*	KU569542	SH1720237.08FU	*G. multiplicatum*
1774	*G. multiplicatum*	KU569549	SH1720237.08FU	*G. multiplicatum*
1775	*G. multiplicatum*	KU569553	SH1723118.08FU	*G. multiplicatum*
1776	*G. multiplicatum*	KX180914	SH1720237.08FU	*G. multiplicatum*
1777	*G. multiplicatum*	MG279185	SH1720237.08FU	*G. multiplicatum*
1778	*G. multiplicatum*	MT232637		*G. multiplicatum*
1779	*G. multiplicatum*	MT232641		*G. multiplicatum*
1780	*G. perzonatum*	KJ792745	SH1720237.08FU	*G. multiplicatum*
1781	*G. perzonatum*	KJ792747	SH1723118.08FU	*G. multiplicatum*
1782	*G. perzonatum*	KJ792749	SH1720237.08FU	*G. multiplicatum*
1783	*G. perzonatum*	KJ792750	SH1720237.08FU	*G. multiplicatum*
1784	*G. perzonatum*	KJ792752	SH1720237.08FU	*G. multiplicatum*
1785	*G. perzonatum*	MK119830		*G. multiplicatum*
1786	*G. multiplicatum*	MT396669		*G. multiplicatum*
1787	*G. perzonatum*	KJ792751	SH1723240.08FU	*G. multiplicatum*
1788	*G. destructans*	KR183856/ NR_132919	SH1723036.08FU	*G. destructans*
1789	*G. destructans*	KR183857	SH1723036.08FU	*G. destructans*
1790	*G. destructans*	KR183858	SH1723036.08FU	*G. destructans*
1791	*G. destructans*	MG020232	SH1723036.08FU	*G. destructans*
1792	*G. destructans*	MG020233	SH1723036.08FU	*G. destructans*
1793	*G. destructans*	MG020234	SH1723036.08FU	*G. destructans*
1794	*G. destructans*	MG020235	SH1723036.08FU	*G. destructans*
1795	*G. destructans*	MG020236	SH1723036.08FU	*G. destructans*
1796	*G. destructans*	MG020237	SH1723036.08FU	*G. destructans*
1797	*G. destructans*	MG020238	SH1723036.08FU	*G. destructans*
1798	*G. destructans*	MG020239	SH1723036.08FU	*G. destructans*
1799	*G. destructans*	MG020240	SH1723036.08FU	*G. destructans*
1800	*G. destructans*	MG020241	SH1723036.08FU	*G. destructans*
1801	*G. destructans*	MG020242		*G. destructans*
1802	*G. destructans*	MG020243	SH1723036.08FU	*G. destructans*
1803	*G. destructans*	MG020244	SH1723036.08FU	*G. destructans*
1804	*G. destructans*	MG020245	SH1723036.08FU	*G. destructans*
1805	*G. destructans*	MG020246	SH1723036.08FU	*G. destructans*
1806	*G. destructans*	MG020247	SH1723036.08FU	*G. destructans*
1807	*G. destructans*	MG020250	SH1723036.08FU	*G. destructans*
1808	*G. destructans*	MG020251	SH1723036.08FU	*G. destructans*
1809	*G. destructans*	MG020252	SH1723036.08FU	*G. destructans*
1810	*G. destructans*	MG020253	SH1723036.08FU	*G. destructans*
1811	*G. destructans*	MG020254	SH1723036.08FU	*G. destructans*
1812	*G. destructans*	MG020256		*G. destructans*
1813	*G. destructans*	MG020257		*G. destructans*
1814	*G. destructans*	MG020258	SH1723036.08FU	*G. destructans*
1815	*G. destructans*	MG020259	SH1723036.08FU	*G. destructans*

1816	*G. destructans*	MG020260	SH1723036.08FU	*G. destructans*
1817	*G. destructans*	MG020261	SH1723036.08FU	*G. destructans*
1818	*G. destructans*	MG020262	SH1723036.08FU	*G. destructans*
1819	*G. destructans*	MG020263	SH1723036.08FU	*G. destructans*
1820	*G. destructans*	MG020266	SH1723036.08FU	*G. destructans*
1821	*G. destructans*	MG020267	SH1723036.08FU	*G. destructans*
1822	*G. destructans*	MG020268	SH1723036.08FU	*G. destructans*
1823	*G. destructans*	MG020269	SH1723036.08FU	*G. destructans*
1824	*G. destructans*	MG279177	SH1723036.08FU	*G. destructans*
1825	*G. destructans*	MH571694	SH1723036.08FU	*G. destructans*
1826	*G. destructans*	MH571695	SH1723036.08FU	*G. destructans*
1827	*G. dunense*	MG020248	SH1723036.08FU	*G. destructans*
1828	*G. dunense*	MG020249	SH1723036.08FU	*G. destructans*
1829	*G. dunense*	MG020255	SH1723036.08FU	*G. destructans*
1830	uncultured soil fungus	UDB0765706	SH1723036.08FU	*G. destructans*
1831	*G. mizoramense*	KY643750	SH1723164.08FU	*G. mizoramense*
1832	*G. mizoramense*	KY643751	SH1723164.08FU	*G. mizoramense*
1833	*G. lucidum*	MG437336	SH1723164.08FU	*G. mizoramense*
1834	*G. steyaertanum*	EU239391/2		*G. steyaertanum*
1835	*G. steyaertanum*	EU239395/6		*G. steyaertanum*
1836	*G. steyaertanum*	KJ654419	SH1723051.08FU	*G. steyaertanum*
1837	*G. steyaertanum*	KJ654420	SH1723051.08FU	*G. steyaertanum*
1838	*G. steyaertanum*	KJ654421	SH1723051.08FU	*G. steyaertanum*
1839	*G. steyaertanum*	KJ654422	SH1723051.08FU	*G. steyaertanum*
1840	*G. steyaertanum*	KJ654423	SH1723051.08FU	*G. steyaertanum*
1841	*G. steyaertanum*	KJ654424	SH1723051.08FU	*G. steyaertanum*
1842	*G. steyaertanum*	KJ654425	SH1723051.08FU	*G. steyaertanum*
1843	*G. steyaertanum*	KJ654426	SH1723051.08FU	*G. steyaertanum*
1844	*G. steyaertanum*	KJ654427	SH1723051.08FU	*G. steyaertanum*
1845	*G. steyaertanum*	KJ654428	SH1723051.08FU	*G. steyaertanum*
1846	*G. steyaertanum*	KJ654429	SH1723051.08FU	*G. steyaertanum*
1847	*G. steyaertanum*	KJ654430	SH1723051.08FU	*G. steyaertanum*
1848	*G. steyaertanum*	KJ654431	SH1723051.08FU	*G. steyaertanum*
1849	*G. steyaertanum*	KJ654432	SH1723051.08FU	*G. steyaertanum*
1850	*G. steyaertanum*	KJ654433	SH1723051.08FU	*G. steyaertanum*
1851	*G. steyaertanum*	KJ654447	SH1723051.08FU	*G. steyaertanum*
1852	*G. steyaertanum*	KJ654448	SH1723051.08FU	*G. steyaertanum*
1853	*G. steyaertanum*	KJ654449	SH1723051.08FU	*G. steyaertanum*
1854	*G. steyaertanum*	KJ654450	SH1723051.08FU	*G. steyaertanum*
1855	*G. steyaertanum*	KJ654451	SH1723051.08FU	*G. steyaertanum*
1856	*G. steyaertanum*	KJ654452	SH1723051.08FU	*G. steyaertanum*
1857	*G. steyaertanum*	KJ654453	SH1723051.08FU	*G. steyaertanum*
1858	*G. steyaertanum*	KJ654454	SH1723051.08FU	*G. steyaertanum*
1859	*G. steyaertanum*	KJ654455	SH1723051.08FU	*G. steyaertanum*
1860	*G. steyaertanum*	KJ654456	SH1723051.08FU	*G. steyaertanum*

1861	*G. steyaertanum*	KJ654457	SH1723051.08FU	*G. steyaertanum*
1862	*G. steyaertanum*	KJ654458	SH1723051.08FU	*G. steyaertanum*
1863	*G. steyaertanum*	KJ654459	SH1723051.08FU	*G. steyaertanum*
1864	*G. steyaertanum*	KJ654460	SH1723051.08FU	*G. steyaertanum*
1865	*G. steyaertanum*	KJ654461	SH1723051.08FU	*G. steyaertanum*
1866	*G. steyaertanum*	KJ654462	SH1723051.08FU	*G. steyaertanum*
1867	*G. steyaertanum*	KP012964	SH1723051.08FU	*G. steyaertanum*
1868	*G.* aff. *steyaertanum*	EU239384		*G. steyaertanum*
1869	*G.* aff. *steyaertanum*	EU239385	SH1723051.08FU	*G. steyaertanum*
1870	*G.* aff. *steyaertanum*	EU239388	SH2751398.08FU	*G. steyaertanum*
1871	*Ganoderma* sp.	MF436674	SH1723051.08FU	*G. steyaertanum*
1872	*Ganoderma* sp.	MK131244	SH1723051.08FU	*G. steyaertanum*
1873	*G. martinicense*	KF963256	SH1723036.08FU	*G. martinicense*
1874	*G. martinicense*	KF963257	SH1723036.08FU	*G. martinicense*
1875	*G. martinicense*	KT247418	SH1720242.08FU	*G. martinicense*
1876	*G. martinicense*	MG279163	SH1723036.08FU	*G. martinicense*
1877	*G. martinicense*	MG654174	SH1723036.08FU	*G. martinicense*
1878	*G. martinicense*	MG654175	SH1723036.08FU	*G. martinicense*
1879	*G. martinicense*	MG654176	SH1723036.08FU	*G. martinicense*
1880	*G. martinicense*	MG654177		*G. martinicense*
1881	*G. martinicense*	MG654178	SH1723036.08FU	*G. martinicense*
1882	*G. martinicense*	MG654179		*G. martinicense*
1883	*G. martinicense*	MG654180	SH1723036.08FU	*G. martinicense*
1884	*G. martinicense*	MG654181	SH1723036.08FU	*G. martinicense*
1885	*G. martinicense*	MG654182	SH1723036.08FU	*G. martinicense*
1886	*G. martinicense*	MG654183	SH1723036.08FU	*G. martinicense*
1887	*G. martinicense*	MG654184		*G. martinicense*
1888	*G. martinicense*	MG654185	SH1723036.08FU	*G. martinicense*
1889	*G. martinicense*	MG654186	SH1723036.08FU	*G. martinicense*
1890	*G. tuberculosum*	AF17001/2 & AH008114	SH1723215.08FU	*G. martinicense*
1891	*G. tornatum*	JQ618246	SH1723036.08FU	*G. martinicense*
1892	*G. parvulum*	JX310817	SH1723138.08FU	*G. martinicense*
1893	*G. parvulum*	JX310818	SH1723138.08FU	*G. martinicense*
1894	*G. parvulum*	JX310819	SH1723036.08FU	*G. martinicense*
1895	*G. parvulum*	JX310820	SH1723036.08FU	*G. martinicense*
1896	*G. parvulum*	JX310821	SH1723036.08FU	*G. martinicense*
1897	*G. parvulum*	JX310822	SH1723036.08FU	*G. martinicense*
1898	*G. perzonatum*	KJ792744		*G. martinicense*
1899	*G. perzonatum*	KJ792746	SH1723266.08FU	*G. martinicense*
1900	*G. parvulum*	KU569510	SH1723036.08FU	*G. martinicense*
1901	*G. parvulum*	KU569511	SH1723036.08FU	*G. martinicense*
1902	*G. parvulum*	KU569512	SH1723036.08FU	*G. martinicense*
1903	*G. parvulum*	KU569513	SH1723036.08FU	*G. martinicense*
1904	*G. parvulum*	KU569514	SH1723036.08FU	*G. martinicense*

1905	*G. parvulum*	KU569516	SH1723036.08FU	*G. martinicense*
1906	*G. parvulum*	KU569518	SH1723036.08FU	*G. martinicense*
1907	*G. parvulum*	KU569519	SH1723036.08FU	*G. martinicense*
1908	*G. parvulum*	KU569520	SH1723036.08FU	*G. martinicense*
1909	*G. parvulum*	KU569521	SH1723036.08FU	*G. martinicense*
1910	*G. parvulum*	KU569522	SH1723036.08FU	*G. martinicense*
1911	*G. parvulum*	KU569523	SH1723036.08FU	*G. martinicense*
1912	*G. parvulum*	KU569524	SH1723036.08FU	*G. martinicense*
1913	*G. parvulum*	KU569525	SH1723036.08FU	*G. martinicense*
1914	*G. parvulum*	KU569526	SH1723036.08FU	*G. martinicense*
1915	*G. parvulum*	KU569527	SH1723036.08FU	*G. martinicense*
1916	*G. parvulum*	KU569530	SH1723036.08FU	*G. martinicense*
1917	*G. parvulum*	KU569555	SH1723036.08FU	*G. martinicense*
1918	*G. lucidum*	KY350855	SH1723036.08FU	*G. martinicense*
1919	*Ganoderma* sp.	LT726720		*G. martinicense*
1920	*G. oerstedii*	Z37059/Z37084		*G. martinicense*
1921	*G. martinicense*	MT232632		*G. martinicense*
1922	*G. multipileum*	EU021462	SH1723036.08FU	*G. multipileum*
1923	*G. multipileum*	JF915409	SH1723036.08FU	*G. multipileum*
1924	*G. multipileum*	JQ781874	SH1723036.08FU	*G. multipileum*
1925	*G. multipileum*	KF494997	SH1723036.08FU	*G. multipileum*
1926	*G. multipileum*	KJ143913	SH1723036.08FU	*G. multipileum*
1927	*G. multipileum*	KJ143914	SH1723036.08FU	*G. multipileum*
1928	*G. multipileum*	KJ868083	SH1723036.08FU	*G. multipileum*
1929	*G. multipileum*	KX055554		*G. multipileum*
1930	*G. multipileum*	KY865255	SH1720253.08FU	*G. multipileum*
1931	*G. multipileum*	LC149613	SH1723036.08FU	*G. multipileum*
1932	*G. multipileum*	MF405163	SH1723036.08FU	*G. multipileum*
1933	*G. multipileum*	MG279184	SH1723036.08FU	*G. multipileum*
1934	*G. multipileum*	MG450346	SH1720248.08FU	*G. multipileum*
1935	*G. multipileum*	MG739453	SH1723036.08FU	*G. multipileum*
1936	*G. multipileum*	MG739454	SH1723036.08FU	*G. multipileum*
1937	*G. multipileum*	MG739455	SH1723036.08FU	*G. multipileum*
1938	*G. multipileum*	MN401406 (~MN401409)		*G. multipileum*
1939	*G. multipileum*	MN401407	SH1723036.08FU	*G. multipileum*
1940	*G. multipileum*	MN533795	SH1723036.08FU	*G. multipileum*
1941	*G. multipileum*	MT765063		*G. multipileum*
1942	*G. multipileum*	MT765065		*G. multipileum*
1943	*G. multipileum*	MT871995		*G. multipileum*
1944	*G. lucidum*	AY636058	SH1723036.08FU	*G. multipileum*
1945	*Ganoderma* sp.	AY636064		*G. multipileum*
1946	*G. lucidum*	AY636068	SH1723036.08FU	*G. multipileum*
1947	*Ganoderma* sp.	AY636069	SH1723036.08FU	*G. multipileum*
1948	*Ganoderma* sp.	AY636070	SH1723036.08FU	*G. multipileum*
1949	*G. lucidum*	DQ425000	SH1723036.08FU	*G. multipileum*

1950	*G. lucidum*	EU021459	SH1720238.08FU	*G. multipileum*
1951	*G. lucidum*	EU021460	SH1723036.08FU	*G. multipileum*
1952	*G. lucidum*	EU021461	SH1723036.08FU	*G. multipileum*
1953	*G. lucidum*	FJ463908		*G. multipileum*
1954	*G. lucidum*	FJ463917	SH1723036.08FU	*G. multipileum*
1955	*G. lucidum*	FJ463920	SH1723036.08FU	*G. multipileum*
1956	*G. lucidum*	FJ463925	SH1723036.08FU	*G. multipileum*
1957	*G. lucidum*	GQ249880	SH1723036.08FU	*G. multipileum*
1958	*G. lucidum*	GQ249884	SH1723036.08FU	*G. multipileum*
1959	*G. lucidum*	GQ249885	SH1723036.08FU	*G. multipileum*
1960	*G. lucidum*	GQ249886	SH1723036.08FU	*G. multipileum*
1961	*G. lucidum*	GU726919		*G. multipileum*
1962	*G. lucidum*	GU726920	SH1723036.08FU	*G. multipileum*
1963	*G. lucidum*	GU726921	SH1723036.08FU	*G. multipileum*
1964	*G. lucidum*	GU726922	SH1723036.08FU	*G. multipileum*
1965	*G. lucidum*	GU726923	SH1723036.08FU	*G. multipileum*
1966	*G. lucidum*	GU726924	SH1723036.08FU	*G. multipileum*
1967	*G. lucidum*	GU726925	SH1723036.08FU	*G. multipileum*
1968	*G. lucidum*	GU726926	SH1723036.08FU	*G. multipileum*
1969	*G. lucidum*	GU726927	SH1723036.08FU	*G. multipileum*
1970	*G. lucidum*	GU726928		*G. multipileum*
1971	*G. lucidum*	GU726929	SH1723036.08FU	*G. multipileum*
1972	*G. lucidum*	GU726930	SH1723036.08FU	*G. multipileum*
1973	*G. lucidum*	GU726931	SH1723036.08FU	*G. multipileum*
1974	*G. lucidum*	GU726932		*G. multipileum*
1975	*G. lucidum*	GU726933	SH1723036.08FU	*G. multipileum*
1976	*G. lucidum*	HM053436	SH1723036.08FU	*G. multipileum*
1977	*G. lucidum*	HM053437	SH1723036.08FU	*G. multipileum*
1978	*G. lucidum*	HM053438	SH1723036.08FU	*G. multipileum*
1979	*G. lucidum*	HM053439	SH1723036.08FU	*G. multipileum*
1980	*G. lucidum*	HM053440	SH1720238.08FU	*G. multipileum*
1981	*G. lucidum*	HM053441	SH1720238.08FU	*G. multipileum*
1982	*G. lucidum*	HM053442	SH1720238.08FU	*G. multipileum*
1983	*G. lucidum*	HM053443	SH1723036.08FU	*G. multipileum*
1984	*G. lucidum*	HM053444	SH1723243.08FU	*G. multipileum*
1985	*G. lucidum*	HM053445	SH1720244.08FU	*G. multipileum*
1986	*G. lucidum*	HM053446	SH1723036.08FU	*G. multipileum*
1987	*G. lucidum*	HM053447	SH1723036.08FU	*G. multipileum*
1988	*G. lucidum*	HM053448	SH1723036.08FU	*G. multipileum*
1989	*G. lucidum*	HM053449	SH1723036.08FU	*G. multipileum*
1990	*G. lucidum*	HM053450	SH1723036.08FU	*G. multipileum*
1991	*G. lucidum*	HM053451	SH1723036.08FU	*G. multipileum*
1992	*G. lucidum*	HM053452	SH1723036.08FU	*G. multipileum*
1993	*G. lucidum*	HM053453	SH1723036.08FU	*G. multipileum*
1994	*G. lucidum*	HM053454	SH1723036.08FU	*G. multipileum*

1995	*G. lucidum*	HM053455		*G. multipileum*
1996	*G. lucidum*	HM053456	SH1720245.08FU	*G. multipileum*
1997	*G. lucidum*	HM053457		*G. multipileum*
1998	*G. lucidum*	HM053458		*G. multipileum*
1999	*G. lucidum*	HM053459		*G. multipileum*
2000	*G. lucidum*	HM053460		*G. multipileum*
2001	*G. lucidum*	HM053461	SH1723036.08FU	*G. multipileum*
2002	*G. lucidum*	HM053462	SH1723036.08FU	*G. multipileum*
2003	*G. lucidum*	HM053463	SH1723036.08FU	*G. multipileum*
2004	*G. lucidum*	HM053464	SH1723036.08FU	*G. multipileum*
2005	*G. lucidum*	HM053465	SH1723036.08FU	*G. multipileum*
2006	*G. lucidum*	HM053466		*G. multipileum*
2007	*G. lucidum*	HM053467	SH1723036.08FU	*G. multipileum*
2008	*G. lucidum*	HM130706	SH1723036.08FU	*G. multipileum*
2009	*G. lucidum*	HQ589218	SH1723036.08FU	*G. multipileum*
2010	*G. lucidum*	HQ589225	SH1723036.08FU	*G. multipileum*
2011	*Ganoderma* sp.	JN049906	SH1723036.08FU	*G. multipileum*
2012	*G. lucidum*	JN099386	SH1723284.08FU	*G. multipileum*
2013	*G. lucidum*	JN613281	SH1723036.08FU	*G. multipileum*
2014	*Ganoderma* sp.	JN810910	SH1720239.08FU	*G. multipileum*
2015	*G. lucidum*	KC807228	SH1720238.08FU	*G. multipileum*
2016	*G. lucidum*	KF549493	SH1723036.08FU	*G. multipileum*
2017	*Ganoderma* sp.	KF998089	SH1723229.08FU	*G. multipileum*
2018	*G. lucidum*	KF998092	SH1720251.08FU	*G. multipileum*
2019	*G. lucidum*	KF998093	SH1720249.08FU	*G. multipileum*
2020	*G. lucidum*	KF998094	SH1723036.08FU	*G. multipileum*
2021	*G. lucidum*	KF998095	SH1723036.08FU	*G. multipileum*
2022	*Ganoderma* sp.	KJ670301	SH1723036.08FU	*G. multipileum*
2023	*Ganoderma* sp.	KJ670304	SH1723036.08FU	*G. multipileum*
2024	*Ganoderma* sp.	KM229564		*G. multipileum*
2025	*Ganoderma* sp.	KM229565	SH1723036.08FU	*G. multipileum*
2026	*Ganoderma* sp.	KM229566	SH1723036.08FU	*G. multipileum*
2027	*Ganoderma* sp.	KM229567		*G. multipileum*
2028	*Ganoderma* sp.	KM229568	SH1723036.08FU	*G. multipileum*
2029	*Ganoderma* sp.	KM229569	SH1723036.08FU	*G. multipileum*
2030	*Ganoderma* sp.	KM229570	SH1723036.08FU	*G. multipileum*
2031	*Ganoderma* sp.	KM229571	SH1723036.08FU	*G. multipileum*
2032	*Ganoderma* sp.	KM229572	SH1723036.08FU	*G. multipileum*
2033	*Ganoderma* sp.	KM229573	SH1723036.08FU	*G. multipileum*
2034	*Ganoderma* sp.	KM229574	SH1723036.08FU	*G. multipileum*
2035	*Ganoderma* sp.	KM229575	SH1723036.08FU	*G. multipileum*
2036	*Ganoderma* sp.	KM229576	SH1723036.08FU	*G. multipileum*
2037	*Ganoderma* sp.	KM229577	SH1723036.08FU	*G. multipileum*
2038	*Ganoderma* sp.	KM229578	SH1723036.08FU	*G. multipileum*
2039	*Ganoderma* sp.	KM229579	SH1723036.08FU	*G. multipileum*

2040	*Ganoderma* sp.	KM229580	SH1723036.08FU	*G. multipileum*
2041	*Ganoderma* sp.	KM229581	SH1723036.08FU	*G. multipileum*
2042	*Ganoderma* sp.	KM229582		*G. multipileum*
2043	*Ganoderma* sp.	KM229583	SH1723036.08FU	*G. multipileum*
2044	*Ganoderma* sp.	KM229584	SH1723036.08FU	*G. multipileum*
2045	*Ganoderma* sp.	KM229585	SH1723036.08FU	*G. multipileum*
2046	*Ganoderma* sp.	KM229586	SH1723036.08FU	*G. multipileum*
2047	*Ganoderma* sp.	KM229587	SH1723036.08FU	*G. multipileum*
2048	*Ganoderma* sp.	KM229588	SH1723036.08FU	*G. multipileum*
2049	*Ganoderma* sp.	KM229589	SH1723036.08FU	*G. multipileum*
2050	*Ganoderma* sp.	KM229590	SH1723036.08FU	*G. multipileum*
2051	*Ganoderma* sp.	KM229591	SH1723036.08FU	*G. multipileum*
2052	*Ganoderma* sp.	KM229592	SH1723036.08FU	*G. multipileum*
2053	*Ganoderma* sp.	KM229593	SH1723036.08FU	*G. multipileum*
2054	*Ganoderma* sp.	KM229594	SH1723036.08FU	*G. multipileum*
2055	*Ganoderma* sp.	KM229595	SH1723036.08FU	*G. multipileum*
2056	*Ganoderma* sp.	KM229596	SH1723036.08FU	*G. multipileum*
2057	*Ganoderma* sp.	KM229597	SH1723036.08FU	*G. multipileum*
2058	*Ganoderma* sp.	KM229598	SH1723036.08FU	*G. multipileum*
2059	*Ganoderma* sp.	KM655758		*G. multipileum*
2060	*G. lucidum*	KP886807	SH1723036.08FU	*G. multipileum*
2061	*Ganoderma* sp.	KR154928	SH1723036.08FU	*G. multipileum*
2062	*Ganoderma* sp.	KR154942	SH1723036.08FU	*G. multipileum*
2063	*Ganoderma* sp.	KR154953	SH1723036.08FU	*G. multipileum*
2064	*Ganoderma* sp.	KR154955	SH1723036.08FU	*G. multipileum*
2065	*Ganoderma* sp.	KR154956	SH1723036.08FU	*G. multipileum*
2066	*Ganoderma* sp.	KR155021	SH1723036.08FU	*G. multipileum*
2067	*Ganoderma* sp.	KR155023	SH1723036.08FU	*G. multipileum*
2068	*Ganoderma* sp.	KR155024	SH1723036.08FU	*G. multipileum*
2069	*Ganoderma* sp.	KR155072	SH1723036.08FU	*G. multipileum*
2070	*Ganoderma* sp.	KR155073	SH1723036.08FU	*G. multipileum*
2071	*Ganoderma* sp.	KR155074	SH1723036.08FU	*G. multipileum*
2072	*Ganoderma* sp.	KR155075	SH1723036.08FU	*G. multipileum*
2073	*Ganoderma* sp.	KR155076	SH1723036.08FU	*G. multipileum*
2074	*Ganoderma* sp.	KR155078	SH1723036.08FU	*G. multipileum*
2075	*Ganoderma* sp.	KR349633		*G. multipileum*
2076	*Ganoderma* sp.	KT188588		*G. multipileum*
2077	*Ganoderma* sp.	KT188593		*G. multipileum*
2078	*Ganoderma* sp.	KT188597		*G. multipileum*
2079	*Ganoderma* sp.	KT188599	SH1723036.08FU	*G. multipileum*
2080	*Ganoderma* sp.	KT188600	SH1723036.08FU	*G. multipileum*
2081	*Ganoderma* sp.	KT188603	SH1723036.08FU	*G. multipileum*
2082	*G. lucidum*	KU194309	SH1723036.08FU	*G. multipileum*
2083	uncultured *Ganoderma*	KU712535	SH1723036.08FU	*G. multipileum*
2084	*G. leucocontextum*	KX055556		*G. multipileum*

2085	*G. lucidum*	KX388170	SH1723036.08FU	*G. multipileum*
2086	*G. lucidum*	KX610998	SH1720248.08FU	*G. multipileum*
2087	*Ganoderma* sp.	KY009865	SH1723250.08FU	*G. multipileum*
2088	*Ganoderma* sp.	KY009871	SH1723036.08FU	*G. multipileum*
2089	*Ganoderma* sp.	KY009872	SH1723232.08FU	*G. multipileum*
2090	*Ganoderma* sp.	KY471289	SH1720247.08FU	*G. multipileum*
2091	*G. lucidum*	KY865256	SH1723036.08FU	*G. multipileum*
2092	*G. lucidum*	MF289193	SH1723036.08FU	*G. multipileum*
2093	*G. lucidum*	MF289194	SH1723036.08FU	*G. multipileum*
2094	*G. lucidum*	MF958936	SH1723036.08FU	*G. multipileum*
2095	*Ganoderma* sp.	MH290247		*G. multipileum*
2096	*Ganoderma* sp.	MH290248	SH1723036.08FU	*G. multipileum*
2097	*Ganoderma* sp.	MH290249		*G. multipileum*
2098	*Ganoderma* sp.	MH290250	SH1723036.08FU	*G. multipileum*
2099	*Ganoderma* sp.	MH290251		*G. multipileum*
2100	*Ganoderma* sp.	MH290252	SH1723036.08FU	*G. multipileum*
2101	*Ganoderma* sp.	MH290253	SH1720248.08FU	*G. multipileum*
2102	*Ganoderma* sp.	MH290254	SH1720248.08FU	*G. multipileum*
2103	*Ganoderma* sp.	MH290255	SH1723036.08FU	*G. multipileum*
2104	*Ganoderma* sp.	MH290256	SH1723036.08FU	*G. multipileum*
2105	*Ganoderma* sp.	MH290257	SH1723036.08FU	*G. multipileum*
2106	*Ganoderma* sp.	MH290258	SH1723036.08FU	*G. multipileum*
2107	*Ganoderma* sp.	MH290259		*G. multipileum*
2108	*Ganoderma* sp.	MH290260		*G. multipileum*
2109	*Ganoderma* sp.	MH290261		*G. multipileum*
2110	*Ganoderma* sp.	MH290262	SH1723036.08FU	*G. multipileum*
2111	*Ganoderma* sp.	MH290263	SH1720248.08FU	*G. multipileum*
2112	*Ganoderma* sp.	MH290264	SH1720248.08FU	*G. multipileum*
2113	*Ganoderma* sp.	MH290265	SH1720248.08FU	*G. multipileum*
2114	*Ganoderma* sp.	MH290266	SH1720248.08FU	*G. multipileum*
2115	*Ganoderma* sp.	MH290267	SH1720248.08FU	*G. multipileum*
2116	*Ganoderma* sp.	MH290268	SH1720239.08FU	*G. multipileum*
2117	*Ganoderma* sp.	MH290269	SH1723036.08FU	*G. multipileum*
2118	*Ganoderma* sp.	MH290271	SH1720248.08FU	*G. multipileum*
2119	*Ganoderma* sp.	MH290272	SH1723036.08FU	*G. multipileum*
2120	*Ganoderma* sp.	MH290274	SH1720248.08FU	*G. multipileum*
2121	*Ganoderma* sp.	MH290275	SH1720248.08FU	*G. multipileum*
2122	*Ganoderma* sp.	MH290276		*G. multipileum*
2123	*Ganoderma* sp.	MH290278	SH1720248.08FU	*G. multipileum*
2124	*Ganoderma* sp.	MH290279	SH1720248.08FU	*G. multipileum*
2125	*Ganoderma* sp.	MH290280	SH1720248.08FU	*G. multipileum*
2126	*Ganoderma* sp.	MH290281	SH1720248.08FU	*G. multipileum*
2127	*Ganoderma* sp.	MH290282	SH1720248.08FU	*G. multipileum*
2128	*Ganoderma* sp.	MH290284	SH1720248.08FU	*G. multipileum*
2129	Agaricales sp.	MK079627	SH1723036.08FU	*G. multipileum*

2130	*G. lingzhi*	MK849887	SH2758629.08FU	*G. multipileum*
2131	*G. lucidum*	MK937782	SH1723036.08FU	*G. multipileum*
2132	*G. lucidum*	MK940282	SH1720248.08FU	*G. multipileum*
2133	*G. lucidum*	MK940283	SH1720248.08FU	*G. multipileum*
2134	*G. lucidum*	MK940284	SH1720248.08FU	*G. multipileum*
2135	*G. lucidum*	MK940285	SH1720248.08FU	*G. multipileum*
2136	*G. lucidum*	MK940288	SH1720239.08FU	*G. multipileum*
2137	*Ganoderma* sp.	MN006955		*G. multipileum*
2138	*G. lucidum*	MN372063	SH2766568.08FU	*G. multipileum*
2139	*G. lucidum*	MN372064	SH1720248.08FU	*G. multipileum*
2140	*G. lucidum*	MN372065	SH1720248.08FU	*G. multipileum*
2141	*G. lucidum*	MN729467		*G. multipileum*
2142	*G. lucidum*	MT126496		*G. multipileum*
2143	*G. lucidum*	MT765061		*G. multipileum*
2144	*G. lucidum*	X78743/X78764		*G. multipileum*
2145	*G. lucidum*	X78744/X78765		*G. multipileum*
2146	*G. lucidum*	X78745/X78766		*G. multipileum*
2147	*G. lucidum*	X87345/X87355		*G. multipileum*
2148	*G. lucidum*	X87346/X87356		*G. multipileum*
2149	*G. lucidum*	X87347/X87357		*G. multipileum*
2150	*G. lucidum*	X87348/X87358		*G. multipileum*
2151	*G. lucidum*	X87349/X87359		*G. multipileum*
2152	*G. lucidum*	X87350/X87360		*G. multipileum*
2153	*G. lucidum*	X87351/X87361		*G. multipileum*
2154	*G. lucidum*	Z37048/Z37072		*G. multipileum*
2155	*G. lucidum*	JN692272	SH1720240.08FU	*G. multipileum*
2156	*Ganoderma* sp.	KR154950	SH1723179.08FU	*G. multipileum*
2157	*Ganoderma* sp.	KR154952	SH1732427.08FU	*G. multipileum*
2158	*Ganoderma* sp.	KR155020	SH1844227.08FU	*G. multipileum*
2159	*Ganoderma* sp.	KR155106	SH1849935.08FU	*G. multipileum*
2160	*Ganoderma* sp.	KT188589	SH1844227.08FU	*G. multipileum*
2161	*Ganoderma* sp.	KT188598		*G. multipileum*
2162	*Ganoderma* sp.	KY009870	SH1849007.08FU	*G. multipileum*
2163	*G. lucidum*	KY865257	SH1720250.08FU	*G. multipileum*
2164	*Ganoderma* sp. ‘multipelium’	MH983559	SH2762233.08FU	*G. multipileum*
2165	*G. tropicum*	FJ491958		*Ganoderma* sp. A5
2166	*G. tropicum*	FJ491959		*Ganoderma* sp. A5
2167	*G. tropicum*	FJ491960		*Ganoderma* sp. A5
2168	*G. tropicum*	FJ491961		*Ganoderma* sp. A5
2169	*G. tropicum*	FJ491962		*Ganoderma* sp. A5
2170	*G. tropicum*	FJ491963		*Ganoderma* sp. A5
2171	*G. tropicum*	FJ491964		*Ganoderma* sp. A5
2172	*G. tropicum*	FJ491965		*Ganoderma* sp. A5
2173	*G. tropicum*	FJ491966	SH1723103.08FU	*Ganoderma* sp. A5
2174	*G. tropicum*	FJ491980		*Ganoderma* sp. A5

2175	*G. tropicum*	FJ491981	SH1723103.08FU	*Ganoderma* sp. A5
2176	*G. tropicum*	FJ491982		*Ganoderma* sp. A5
2177	*G. tropicum*	FJ491983	SH1723103.08FU	*Ganoderma* sp. A5
2178	*G. tropicum*	FJ491984		*Ganoderma* sp. A5
2179	*G. tropicum*	FJ491985	SH1723103.08FU	*Ganoderma* sp. A5
2180	*G. tropicum*	EU021457	SH1723057.08FU	*G. tropicum*
2181	*G. tropicum*	EU021458	SH1723057.08FU	*G. tropicum*
2182	*G. tropicum*	JF915410	SH1723057.08FU	*G. tropicum*
2183	*G. tropicum*	JQ781879	SH1723057.08FU	*G. tropicum*
2184	*G. tropicum*	JQ781880	SH1723057.08FU	*G. tropicum*
2185	*G. tropicum*	KC222317	SH1723057.08FU	*G. tropicum*
2186	*G. tropicum*	KF495000	SH1723057.08FU	*G. tropicum*
2187	*G. tropicum*	MG279194	SH1723057.08FU	*G. tropicum*
2188	*G. tropicum*	MH106884	SH1723057.08FU	*G. tropicum*
2189	*G. tropicum*	MH823539	SH1723057.08FU	*G. tropicum*
2190	*G. tropicum*	MK007291	SH1723057.08FU	*G. tropicum*
2191	*G. tropicum*	MK345456		*G. tropicum*
2192	*G. tropicum*	Z37068/Z37088		*G. tropicum*
2193	*G. fornicatum*	AY593859	SH1723057.08FU	*G. tropicum*
2194	*G. fornicatum*	AY593860	SH1723057.08FU	*G. tropicum*
2195	*G. fornicatum*	AY593861	SH1723057.08FU	*G. tropicum*
2196	*G. fornicatum*	AY593862	SH1723057.08FU	*G. tropicum*
2197	*G. fornicatum*	AY593863	SH1723057.08FU	*G. tropicum*
2198	*G. fornicatum*	FJ655471	SH1723057.08FU	*G. tropicum*
2199	*G. fornicatum*	FJ655472	SH1723057.08FU	*G. tropicum*
2200	*G. fornicatum*	FJ655473		*G. tropicum*
2201	*G. fornicatum*	FJ655474	SH1723057.08FU	*G. tropicum*
2202	*G. fornicatum*	FJ655475	SH1723057.08FU	*G. tropicum*
2203	*G. fornicatum*	FJ655476	SH1723057.08FU	*G. tropicum*
2204	*G. fornicatum*	Z37067/Z37087		*G. tropicum*
2205	*Ganoderma* sp.	KP027536	SH1723057.08FU	*G. tropicum*
2206	*Vanderbylia fraxinea*	KX081091	SH1723057.08FU	*G. tropicum*
2207	*G. williamsianum*	MK345457		*G. tropicum*
2208	*G. williamsianum*	MK345458	SH1723057.08FU	*G. tropicum*
2209	*Vanderbylia fraxinea*	MN523320	SH1723057.08FU	*G. tropicum*
2210	uncultured soil fungus	UDB033739	SH1723057.08FU	*G. tropicum*
2211	*G. tropicum*	MT364498		*G. tropicum*
2212	*G. tropicum*	MT364499		*G. tropicum*
2213	*G. fornicatum*	JN596323	SH1723183.08FU	*Ganoderma* sp. A7
2214	*G. fornicatum*	JN596324		*Ganoderma* sp. A7
2215	*G. fornicatum*	JN596325		*Ganoderma* sp. A7
2216	*Ganoderma* sp.	KF605666	SH1723111.08FU	*Ganoderma* sp. B1
2217	*Ganoderma* sp.	KF605667	SH1723111.08FU	*Ganoderma* sp. B1
2218	*Ganoderma* sp.	KF605668	SH1723111.08FU	*Ganoderma* sp. B1
2219	*Ganoderma* sp.	MG279151	SH1723111.08FU	*Ganoderma* sp. B1

2220	*G. multipileum*	AB811849		*Ganoderma* sp. B2
2221	*G. applanatum*	AB811850	SH1723166.08FU	*Ganoderma* sp. B2
2222	*G. lingzhi*	AB811852		*Ganoderma* sp. B2
2223	*G. applanatum*	AF255092		*G. applanatum*
2224	*G. applanatum*	AF255093	SH1179604.08FU	*G. applanatum*
2225	*G. applanatum*	AY884178	SH1179604.08FU	*G. applanatum*
2226	*G. applanatum*	AY884179	SH1179604.08FU	*G. applanatum*
2227	*G. applanatum*	FJ655454		*G. applanatum*
2228	*G. applanatum*	FJ655455		*G. applanatum*
2229	*G. applanatum*	FJ655456	SH1179604.08FU	*G. applanatum*
2230	*G. applanatum*	FJ655457	SH1179604.08FU	*G. applanatum*
2231	*G. applanatum*	FR686556	SH1179604.08FU	*G. applanatum*
2232	*G. applanatum*	GU256764	SH1179604.08FU	*G. applanatum*
2233	*G. applanatum*	JN176900	SH1179604.08FU	*G. applanatum*
2234	*G. applanatum*	JN588587	SH1179604.08FU	*G. applanatum*
2235	*G. applanatum*	JQ520162		*G. applanatum*
2236	*G. applanatum*	JX501311	SH1179604.08FU	*G. applanatum*
2237	*G. applanatum*	KC505588		*G. applanatum*
2238	*G. applanatum*	KC581319	SH1179604.08FU	*G. applanatum*
2239	*G. applanatum*	KF495001		*G. applanatum*
2240	*G. applanatum*	KF605647	SH1179604.08FU	*G. applanatum*
2241	*G. applanatum*	KF605648		*G. applanatum*
2242	*G. applanatum*	KF605649		*G. applanatum*
2243	*G. applanatum*	KF975891		*G. applanatum*
2244	*G. applanatum*	KF975892	SH1179604.08FU	*G. applanatum*
2245	*G. applanatum*	KJ140577	SH1179604.08FU	*G. applanatum*
2246	*G. applanatum*	KJ668542		*G. applanatum*
2247	*G. applanatum*	KJ857258		*G. applanatum*
2248	*G. applanatum*	KJ857259	SH1179604.08FU	*G. applanatum*
2249	*G. applanatum*	KJ857265	SH1179604.08FU	*G. applanatum*
2250	*G. applanatum*	KJ857266	SH1179604.08FU	*G. applanatum*
2251	*G. applanatum*	KR673611		*G. applanatum*
2252	*G. applanatum*	KT318588	SH1179604.08FU	*G. applanatum*
2253	*G. applanatum*	KT318589	SH1179604.08FU	*G. applanatum*
2254	*G. applanatum*	KU219987		*G. applanatum*
2255	*G. applanatum*	KU863083	SH1179604.08FU	*G. applanatum*
2256	*G. applanatum*	KU948520	SH1179604.08FU	*G. applanatum*
2257	*G. applanatum*	KX055561	SH1179604.08FU	*G. applanatum*
2258	*G. applanatum*	KX449492		*G. applanatum*
2259	*G. applanatum*	KY364255		*G. applanatum*
2260	*G. applanatum*	KY364256	SH1179604.08FU	*G. applanatum*
2261	*G. applanatum*	KY364257		*G. applanatum*
2262	*G. applanatum*	KY364258	SH1179604.08FU	*G. applanatum*
2263	*G. applanatum*	MF143510	SH1179604.08FU	*G. applanatum*
2264	*G. applanatum*	MF143511	SH1179604.08FU	*G. applanatum*

2265	*G. applanatum*	MF143512	SH1179604.08FU	*G. applanatum*
2266	*G. applanatum*	MF143513	SH1179604.08FU	*G. applanatum*
2267	*G. applanatum*	MF143514		*G. applanatum*
2268	*G. applanatum*	MF143515		*G. applanatum*
2269	*G. applanatum*	MF161177		*G. applanatum*
2270	*G. applanatum*	MF161255	SH1179604.08FU	*G. applanatum*
2271	*G. applanatum*	MF161295		*G. applanatum*
2272	*G. applanatum*	MG279155		*G. applanatum*
2273	*G. applanatum*	MG279156		*G. applanatum*
2274	*G. applanatum*	MG279157	SH1179604.08FU	*G. applanatum*
2275	*G. applanatum*	MG279158	SH1179604.08FU	*G. applanatum*
2276	*G. applanatum*	MG706213	SH1179604.08FU	*G. applanatum*
2277	*G. applanatum*	MG706214		*G. applanatum*
2278	*G. applanatum*	MG706215	SH1179604.08FU	*G. applanatum*
2279	*G. applanatum*	MG835863	SH1179604.08FU	*G. applanatum*
2280	*G. applanatum*	MH027634	SH1179604.08FU	*G. applanatum*
2281	*G. applanatum*	MH114666	SH1179604.08FU	*G. applanatum*
2282	*G. applanatum*	MH160077	SH1179604.08FU	*G. applanatum*
2283	*G. applanatum*	MH191249		*G. applanatum*
2284	*G. applanatum*	MH320562	SH1179604.08FU	*G. applanatum*
2285	*G. applanatum*	MH321900	SH1179604.08FU	*G. applanatum*
2286	*G. applanatum*	MH425253		*G. applanatum*
2287	*G. applanatum*	MH930900	SH1179604.08FU	*G. applanatum*
2288	*G. applanatum*	MK345426		*G. applanatum*
2289	*G. applanatum*	MK345427		*G. applanatum*
2290	*G. applanatum*	MK351671	SH1179604.08FU	*G. applanatum*
2291	*G. applanatum*	MK351672	SH1179604.08FU	*G. applanatum*
2292	*G. applanatum*	MK351677	SH1179604.08FU	*G. applanatum*
2293	*G. applanatum*	MK351727	SH1179604.08FU	*G. applanatum*
2294	*G. applanatum*	MK351745	SH1179604.08FU	*G. applanatum*
2295	*G. applanatum*	MK415242		*G. applanatum*
2296	*G. applanatum*	MK415243		*G. applanatum*
2297	*G. applanatum*	MK415245		*G. applanatum*
2298	*G. applanatum*	MK415246		*G. applanatum*
2299	*G. applanatum*	MK415247		*G. applanatum*
2300	*G. applanatum*	MK415248		*G. applanatum*
2301	*G. applanatum*	MK415249		*G. applanatum*
2302	*G. applanatum*	MK415251		*G. applanatum*
2303	*G. applanatum*	MK415252		*G. applanatum*
2304	*G. applanatum*	MK415253		*G. applanatum*
2305	*G. applanatum*	MK415254		*G. applanatum*
2306	*G. applanatum*	MK415255		*G. applanatum*
2307	*G. applanatum*	MK415260		*G. applanatum*
2308	*G. applanatum*	MK415264		*G. applanatum*
2309	*G. applanatum*	MK415273		*G. applanatum*

2310	*G. applanatum*	MK415275		*G. applanatum*
2311	*G. applanatum*	MK415277		*G. applanatum*
2312	*G. applanatum*	MK415278		*G. applanatum*
2313	*G. applanatum*	MK415279		*G. applanatum*
2314	*G. applanatum*	MK415281		*G. applanatum*
2315	*G. applanatum*	MK415282		*G. applanatum*
2316	*G. applanatum*	MK415283		*G. applanatum*
2317	*G. applanatum*	MK415284		*G. applanatum*
2318	*G. applanatum*	MK415297		*G. applanatum*
2319	*G. applanatum*	MK415298		*G. applanatum*
2320	*G. applanatum*	MK415299		*G. applanatum*
2321	*G. applanatum*	MK415308		*G. applanatum*
2322	*G. applanatum*	MK415313		*G. applanatum*
2323	*G. applanatum*	MN173820		*G. applanatum*
2324	*G. applanatum*	MN435132		*G. applanatum*
2325	*G. applanatum*	MN435133		*G. applanatum*
2326	*G. applanatum*	MN435134		*G. applanatum*
2327	*G. applanatum*	MN435135		*G. applanatum*
2328	*G. applanatum*	MN435136		*G. applanatum*
2329	*G. applanatum*	MN435137		*G. applanatum*
2330	*G. applanatum*	MN435138		*G. applanatum*
2331	*G. applanatum*	MN435139		*G. applanatum*
2332	*G. applanatum*	MN435140		*G. applanatum*
2333	*G. applanatum*	MN435141		*G. applanatum*
2334	*G. applanatum*	MN435142		*G. applanatum*
2335	*G. applanatum*	MN435143		*G. applanatum*
2336	*G. applanatum*	MN435144		*G. applanatum*
2337	*G. applanatum*	MN435145		*G. applanatum*
2338	*G. applanatum*	MN435146		*G. applanatum*
2339	*G. applanatum*	MN906143		*G. applanatum*
2340	*G. applanatum*	MN992389		*G. applanatum*
2341	*G. applanatum*	UDB011619	SH1179604.08FU	*G. applanatum*
2342	*G. applanatum* cplx	AF255094	SH1179604.08FU	*G. applanatum*
2343	*G. applanatum* cplx	AF255095	SH1179604.08FU	*G. applanatum*
2344	*G.* cf. *applanatum*	AF255096	SH1179604.08FU	*G. applanatum*
2345	*G. applanatum* cplx	AF255097		*G. applanatum*
2346	*Trametes* sp.	AY968082		*G. applanatum*
2347	uncultured soil fungus	DQ421100		*G. applanatum*
2348	uncultured soil fungus	DQ421109		*G. applanatum*
2349	uncultured soil fungus	DQ421110		*G. applanatum*
2350	uncultured soil fungus	DQ421242		*G. applanatum*
2351	*G. lipsiense*	EF059994		*G. applanatum*
2352	*G. lipsiense*	EF059995		*G. applanatum*
2353	*G. lipsiense*	EF059996		*G. applanatum*
2354	*G. lipsiense*	EF059997		*G. applanatum*

2355	*G. lipsiense*	EF059998		*G. applanatum*
2356	*G. lipsiense*	EF059999		*G. applanatum*
2357	*G. lipsiense*	EF060000		*G. applanatum*
2358	*G. lipsiense*	EF060001		*G. applanatum*
2359	*G. lipsiense*	EF060002		*G. applanatum*
2360	*G. lipsiense*	EF060003		*G. applanatum*
2361	*G. lipsiense*	EF060004		*G. applanatum*
2362	*G. lipsiense*	EF060005		*G. applanatum*
2363	*G. lipsiense*	EF060006	SH1179604.08FU	*G. applanatum*
2364	uncultured fungus	EU554851		*G. applanatum*
2365	uncultured fungus	EU554859	SH1179604.08FU	*G. applanatum*
2366	uncultured fungus	EU554863		*G. applanatum*
2367	uncultured *Ganoderma*	FJ197949	SH1179604.08FU	*G. applanatum*
2368	fungal sp.	FJ609287	SH1179604.08FU	*G. applanatum*
2369	uncultured fungus	FJ626936		*G. applanatum*
2370	*G. adspersum*	FJ655448		*G. applanatum*
2371	*G. adspersum*	FJ655450		*G. applanatum*
2372	uncultured fungus	FR750674	SH1179604.08FU	*G. applanatum*
2373	uncultured soil fungus	GU083147		*G. applanatum*
2374	*G. adspersum*	GU731555	SH1179604.08FU	*G. applanatum*
2375	*G. adspersum*	GU731556	SH2752132.08FU	*G. applanatum*
2376	uncultured *Ganoderma*	HQ021834		*G. applanatum*
2377	uncultured *Ganoderma*	HQ021835		*G. applanatum*
2378	uncultured *Ganoderma*	HQ021836		*G. applanatum*
2379	uncultured *Ganoderma*	HQ021975		*G. applanatum*
2380	uncultured *Ganoderma*	HQ022119		*G. applanatum*
2381	uncultured *Ganoderma*	HQ022243	SH1179604.08FU	*G. applanatum*
2382	*G. australe*	JN048773		*G. applanatum*
2383	*G. oregonense*	JQ520196	SH1179604.08FU	*G. applanatum*
2384	*Ganoderma* sp.	JQ520206		*G. applanatum*
2385	uncultured soil fungus	JQ666446		*G. applanatum*
2386	uncultured soil fungus	JQ666457		*G. applanatum*
2387	uncultured soil fungus	JQ666465		*G. applanatum*
2388	uncultured soil fungus	JQ666495		*G. applanatum*
2389	uncultured soil fungus	JQ666507		*G. applanatum*
2390	uncultured soil fungus	JQ666577		*G. applanatum*
2391	uncultured soil fungus	JQ666595		*G. applanatum*
2392	uncultured soil fungus	JQ666726		*G. applanatum*
2393	uncultured *Ganoderma*	JX675211		*G. applanatum*
2394	uncultured *Ganoderma*	JX675216		*G. applanatum*
2395	uncultured *Ganoderma*	KC785577		*G. applanatum*
2396	uncultured fungus	KF800100		*G. applanatum*
2397	*G. adspersum*	KP941443	SH1179604.08FU	*G. applanatum*
2398	uncultured *Ganoderma*	KT334783	SH1179604.08FU	*G. applanatum*
2399	uncultured *Ganoderma*	KT334785	SH1179604.08FU	*G. applanatum*

2400	uncultured *Ganoderma*	KT334788	SH1179604.08FU	*G. applanatum*
2401	uncultured *Ganoderma*	KT334790	SH1179604.08FU	*G. applanatum*
2402	uncultured fungus	LC096119		*G. applanatum*
2403	*G. lipsiense*	MF686522	SH1179604.08FU	*G. applanatum*
2404	*G. lipsiense*	MH855178	SH1179604.08FU	*G. applanatum*
2405	*Ganoderma* sp.	MK050641		*G. applanatum*
2406	*G. lobatum*	MK393922	SH1179604.08FU	*G. applanatum*
2407	*G. lipsiense*	MN396332		*G. applanatum*
2408	*G. lipsiense*	MN396333		*G. applanatum*
2409	*G. lipsiense*	MN396334		*G. applanatum*
2410	*G. lipsiense*	MN396335		*G. applanatum*
2411	*G. lipsiense*	MN396336		*G. applanatum*
2412	*G. lipsiense*	MN396337		*G. applanatum*
2413	uncultured fungus	MT236432		*G. applanatum*
2414	uncultured soil fungus	UDB0100231	SH1179604.08FU	*G. applanatum*
2415	uncultured soil fungus	UDB0101232	SH1179604.08FU	*G. applanatum*
2416	uncultured soil fungus	UDB0103060	SH1179604.08FU	*G. applanatum*
2417	uncultured soil fungus	UDB0119647	SH1179604.08FU	*G. applanatum*
2418	uncultured soil fungus	UDB0121485	SH1179604.08FU	*G. applanatum*
2419	uncultured soil fungus	UDB0122574	SH1179604.08FU	*G. applanatum*
2420	uncultured soil fungus	UDB0124542	SH1179604.08FU	*G. applanatum*
2421	uncultured soil fungus	UDB0126096	SH1179604.08FU	*G. applanatum*
2422	uncultured soil fungus	UDB0130744	SH1179604.08FU	*G. applanatum*
2423	uncultured soil fungus	UDB0132569	SH1179604.08FU	*G. applanatum*
2424	uncultured soil fungus	UDB0133733	SH1179604.08FU	*G. applanatum*
2425	uncultured soil fungus	UDB0134557	SH1179604.08FU	*G. applanatum*
2426	uncultured soil fungus	UDB0135708	SH1179604.08FU	*G. applanatum*
2427	uncultured soil fungus	UDB0139875	SH1179604.08FU	*G. applanatum*
2428	uncultured soil fungus	UDB0142408	SH1179604.08FU	*G. applanatum*
2429	uncultured soil fungus	UDB0149952	SH1179604.08FU	*G. applanatum*
2430	uncultured soil fungus	UDB0151129	SH1179604.08FU	*G. applanatum*
2431	uncultured soil fungus	UDB0152824	SH1179604.08FU	*G. applanatum*
2432	uncultured soil fungus	UDB0157946	SH1179604.08FU	*G. applanatum*
2433	uncultured soil fungus	UDB0161689	SH1179604.08FU	*G. applanatum*
2434	uncultured soil fungus	UDB0162233	SH1179604.08FU	*G. applanatum*
2435	uncultured soil fungus	UDB0167266	SH1179604.08FU	*G. applanatum*
2436	uncultured soil fungus	UDB0167717	SH1179604.08FU	*G. applanatum*
2437	uncultured soil fungus	UDB0170440	SH1179604.08FU	*G. applanatum*
2438	uncultured soil fungus	UDB0179882	SH1179604.08FU	*G. applanatum*
2439	uncultured soil fungus	UDB0181758	SH1179604.08FU	*G. applanatum*
2440	uncultured soil fungus	UDB0184967	SH1179604.08FU	*G. applanatum*
2441	uncultured soil fungus	UDB0186902	SH1179604.08FU	*G. applanatum*
2442	uncultured soil fungus	UDB0187261	SH1179604.08FU	*G. applanatum*
2443	uncultured soil fungus	UDB0189759	SH1179604.08FU	*G. applanatum*
2444	uncultured soil fungus	UDB0201672	SH1179604.08FU	*G. applanatum*

2445	uncultured soil fungus	UDB0203037	SH1179604.08FU	*G. applanatum*
2446	uncultured soil fungus	UDB0214566	SH1179604.08FU	*G. applanatum*
2447	uncultured soil fungus	UDB0215659	SH1179604.08FU	*G. applanatum*
2448	uncultured soil fungus	UDB0216981	SH1179604.08FU	*G. applanatum*
2449	uncultured soil fungus	UDB0219498	SH1179604.08FU	*G. applanatum*
2450	uncultured soil fungus	UDB0222995	SH1179604.08FU	*G. applanatum*
2451	uncultured soil fungus	UDB0224112	SH1179604.08FU	*G. applanatum*
2452	uncultured soil fungus	UDB0225875	SH1179604.08FU	*G. applanatum*
2453	uncultured soil fungus	UDB0228887	SH1179604.08FU	*G. applanatum*
2454	uncultured soil fungus	UDB0232284	SH1179604.08FU	*G. applanatum*
2455	uncultured soil fungus	UDB0241972	SH1179604.08FU	*G. applanatum*
2456	uncultured soil fungus	UDB0246103	SH1179604.08FU	*G. applanatum*
2457	uncultured soil fungus	UDB0247307	SH1179604.08FU	*G. applanatum*
2458	uncultured soil fungus	UDB0253438	SH1179604.08FU	*G. applanatum*
2459	uncultured soil fungus	UDB0257893	SH1179604.08FU	*G. applanatum*
2460	uncultured soil fungus	UDB0261000	SH1179604.08FU	*G. applanatum*
2461	uncultured soil fungus	UDB0261967	SH1179604.08FU	*G. applanatum*
2462	uncultured soil fungus	UDB0265157	SH1179604.08FU	*G. applanatum*
2463	uncultured soil fungus	UDB0266885	SH1179604.08FU	*G. applanatum*
2464	uncultured soil fungus	UDB0268901	SH1179604.08FU	*G. applanatum*
2465	uncultured soil fungus	UDB0275826	SH1179604.08FU	*G. applanatum*
2466	uncultured soil fungus	UDB0276847	SH1179604.08FU	*G. applanatum*
2467	uncultured soil fungus	UDB0280808	SH1179604.08FU	*G. applanatum*
2468	uncultured soil fungus	UDB0287464	SH1179604.08FU	*G. applanatum*
2469	uncultured soil fungus	UDB0295117	SH1179604.08FU	*G. applanatum*
2470	uncultured soil fungus	UDB0300725	SH1179604.08FU	*G. applanatum*
2471	uncultured soil fungus	UDB0309683	SH1179604.08FU	*G. applanatum*
2472	uncultured soil fungus	UDB0310323	SH1179604.08FU	*G. applanatum*
2473	uncultured soil fungus	UDB0311723	SH1179604.08FU	*G. applanatum*
2474	uncultured soil fungus	UDB0322707	SH1179604.08FU	*G. applanatum*
2475	uncultured soil fungus	UDB0324687	SH1179604.08FU	*G. applanatum*
2476	uncultured soil fungus	UDB0328840	SH1179604.08FU	*G. applanatum*
2477	uncultured soil fungus	UDB0328942	SH1179604.08FU	*G. applanatum*
2478	uncultured soil fungus	UDB0331720	SH1179604.08FU	*G. applanatum*
2479	uncultured soil fungus	UDB0335181	SH1179604.08FU	*G. applanatum*
2480	uncultured soil fungus	UDB0338913	SH1179604.08FU	*G. applanatum*
2481	uncultured soil fungus	UDB0343801	SH1179604.08FU	*G. applanatum*
2482	uncultured soil fungus	UDB0351962	SH1179604.08FU	*G. applanatum*
2483	uncultured soil fungus	UDB0352927	SH1179604.08FU	*G. applanatum*
2484	uncultured soil fungus	UDB0359085	SH1179604.08FU	*G. applanatum*
2485	uncultured soil fungus	UDB0374866	SH1179604.08FU	*G. applanatum*
2486	uncultured soil fungus	UDB0376096	SH1179604.08FU	*G. applanatum*
2487	uncultured soil fungus	UDB0376950	SH1179604.08FU	*G. applanatum*
2488	uncultured soil fungus	UDB0380819	SH1179604.08FU	*G. applanatum*
2489	uncultured soil fungus	UDB0388764	SH1179604.08FU	*G. applanatum*

2490	uncultured soil fungus	UDB0395610	SH1179604.08FU	*G. applanatum*
2491	uncultured soil fungus	UDB0400307	SH1179604.08FU	*G. applanatum*
2492	uncultured soil fungus	UDB040056	SH1179604.08FU	*G. applanatum*
2493	uncultured soil fungus	UDB040056	SH1179604.08FU	*G. applanatum*
2494	uncultured soil fungus	UDB0401075	SH1179604.08FU	*G. applanatum*
2495	uncultured soil fungus	UDB0405032	SH1179604.08FU	*G. applanatum*
2496	uncultured soil fungus	UDB0407497	SH1179604.08FU	*G. applanatum*
2497	uncultured soil fungus	UDB0408981	SH1179604.08FU	*G. applanatum*
2498	uncultured soil fungus	UDB041383	SH1179604.08FU	*G. applanatum*
2499	uncultured soil fungus	UDB041383	SH1179604.08FU	*G. applanatum*
2500	uncultured soil fungus	UDB0418862	SH1179604.08FU	*G. applanatum*
2501	uncultured soil fungus	UDB0420804	SH1179604.08FU	*G. applanatum*
2502	uncultured soil fungus	UDB0421269	SH1179604.08FU	*G. applanatum*
2503	uncultured soil fungus	UDB0422666	SH1179604.08FU	*G. applanatum*
2504	uncultured soil fungus	UDB0422845	SH1179604.08FU	*G. applanatum*
2505	uncultured soil fungus	UDB042325	SH1179604.08FU	*G. applanatum*
2506	uncultured soil fungus	UDB0423702	SH1179604.08FU	*G. applanatum*
2507	uncultured soil fungus	UDB0428244	SH1179604.08FU	*G. applanatum*
2508	uncultured soil fungus	UDB0429138	SH1179604.08FU	*G. applanatum*
2509	uncultured soil fungus	UDB0430744	SH1179604.08FU	*G. applanatum*
2510	uncultured soil fungus	UDB0435574	SH1179604.08FU	*G. applanatum*
2511	uncultured soil fungus	UDB0437183	SH1179604.08FU	*G. applanatum*
2512	uncultured soil fungus	UDB0438491	SH1179604.08FU	*G. applanatum*
2513	uncultured soil fungus	UDB0440440	SH1179604.08FU	*G. applanatum*
2514	uncultured soil fungus	UDB0443642	SH1179604.08FU	*G. applanatum*
2515	uncultured soil fungus	UDB0446806	SH1179604.08FU	*G. applanatum*
2516	uncultured soil fungus	UDB0454763	SH1179604.08FU	*G. applanatum*
2517	uncultured soil fungus	UDB0455904	SH1179604.08FU	*G. applanatum*
2518	uncultured soil fungus	UDB0456406	SH1179604.08FU	*G. applanatum*
2519	uncultured soil fungus	UDB0456671	SH1179604.08FU	*G. applanatum*
2520	uncultured soil fungus	UDB0461993	SH1179604.08FU	*G. applanatum*
2521	uncultured soil fungus	UDB0463272	SH1179604.08FU	*G. applanatum*
2522	uncultured soil fungus	UDB0465624	SH1179604.08FU	*G. applanatum*
2523	uncultured soil fungus	UDB0466209	SH1179604.08FU	*G. applanatum*
2524	uncultured soil fungus	UDB0467477	SH1179604.08FU	*G. applanatum*
2525	uncultured soil fungus	UDB0469923	SH1179604.08FU	*G. applanatum*
2526	uncultured soil fungus	UDB0481250	SH1179604.08FU	*G. applanatum*
2527	uncultured soil fungus	UDB048343	SH1179604.08FU	*G. applanatum*
2528	uncultured soil fungus	UDB0484438	SH1179604.08FU	*G. applanatum*
2529	uncultured soil fungus	UDB0489241	SH1179604.08FU	*G. applanatum*
2530	uncultured soil fungus	UDB0490998	SH1179604.08FU	*G. applanatum*
2531	uncultured soil fungus	UDB0492431	SH1179604.08FU	*G. applanatum*
2532	uncultured soil fungus	UDB0493384	SH1179604.08FU	*G. applanatum*
2533	uncultured soil fungus	UDB0497080	SH1179604.08FU	*G. applanatum*
2534	uncultured soil fungus	UDB0501364	SH1179604.08FU	*G. applanatum*

2535	uncultured soil fungus	UDB0502251	SH1179604.08FU	*G. applanatum*
2536	uncultured soil fungus	UDB050390	SH1179604.08FU	*G. applanatum*
2537	uncultured soil fungus	UDB0504358	SH1179604.08FU	*G. applanatum*
2538	uncultured soil fungus	UDB0504458	SH1179604.08FU	*G. applanatum*
2539	uncultured soil fungus	UDB0510029	SH1179604.08FU	*G. applanatum*
2540	uncultured soil fungus	UDB0512395	SH1179604.08FU	*G. applanatum*
2541	uncultured soil fungus	UDB0512540	SH1179604.08FU	*G. applanatum*
2542	uncultured soil fungus	UDB0514622	SH1179604.08FU	*G. applanatum*
2543	uncultured soil fungus	UDB0515632	SH1179604.08FU	*G. applanatum*
2544	uncultured soil fungus	UDB0518466	SH1179604.08FU	*G. applanatum*
2545	uncultured soil fungus	UDB0532721	SH1179604.08FU	*G. applanatum*
2546	uncultured soil fungus	UDB0536161	SH1179604.08FU	*G. applanatum*
2547	uncultured soil fungus	UDB0541946	SH1179604.08FU	*G. applanatum*
2548	uncultured soil fungus	UDB0544560	SH1179604.08FU	*G. applanatum*
2549	uncultured soil fungus	UDB0556942	SH1179604.08FU	*G. applanatum*
2550	uncultured soil fungus	UDB0557948	SH1179604.08FU	*G. applanatum*
2551	uncultured soil fungus	UDB0566431	SH1179604.08FU	*G. applanatum*
2552	uncultured soil fungus	UDB0576874	SH1179604.08FU	*G. applanatum*
2553	uncultured soil fungus	UDB0578535	SH1179604.08FU	*G. applanatum*
2554	uncultured soil fungus	UDB0578737	SH1179604.08FU	*G. applanatum*
2555	uncultured soil fungus	UDB0580293	SH1179604.08FU	*G. applanatum*
2556	uncultured soil fungus	UDB0580479	SH1179604.08FU	*G. applanatum*
2557	uncultured soil fungus	UDB0583647	SH1179604.08FU	*G. applanatum*
2558	uncultured soil fungus	UDB0584468	SH1179604.08FU	*G. applanatum*
2559	uncultured soil fungus	UDB0608795	SH1179604.08FU	*G. applanatum*
2560	uncultured soil fungus	UDB061192	SH1179604.08FU	*G. applanatum*
2561	uncultured soil fungus	UDB0613546	SH1179604.08FU	*G. applanatum*
2562	uncultured soil fungus	UDB0613654	SH1179604.08FU	*G. applanatum*
2563	uncultured soil fungus	UDB0615105	SH1179604.08FU	*G. applanatum*
2564	uncultured soil fungus	UDB0616935	SH1179604.08FU	*G. applanatum*
2565	uncultured soil fungus	UDB0622788	SH1179604.08FU	*G. applanatum*
2566	uncultured soil fungus	UDB0623034	SH1179604.08FU	*G. applanatum*
2567	uncultured soil fungus	UDB0625825	SH1179604.08FU	*G. applanatum*
2568	uncultured soil fungus	UDB0626374	SH1179604.08FU	*G. applanatum*
2569	uncultured soil fungus	UDB0630541	SH1179604.08FU	*G. applanatum*
2570	uncultured soil fungus	UDB0635185	SH1179604.08FU	*G. applanatum*
2571	uncultured soil fungus	UDB063943	SH1179604.08FU	*G. applanatum*
2572	uncultured soil fungus	UDB0639460	SH1179604.08FU	*G. applanatum*
2573	uncultured soil fungus	UDB064483	SH1179604.08FU	*G. applanatum*
2574	uncultured soil fungus	UDB0647100	SH1179604.08FU	*G. applanatum*
2575	uncultured soil fungus	UDB0652930	SH1179604.08FU	*G. applanatum*
2576	uncultured soil fungus	UDB0654560	SH1179604.08FU	*G. applanatum*
2577	uncultured soil fungus	UDB0655465	SH1179604.08FU	*G. applanatum*
2578	uncultured soil fungus	UDB0658413	SH1179604.08FU	*G. applanatum*
2579	uncultured soil fungus	UDB0659247	SH1179604.08FU	*G. applanatum*

2580	uncultured soil fungus	UDB0661494	SH1179604.08FU	*G. applanatum*
2581	uncultured soil fungus	UDB0668753	SH1179604.08FU	*G. applanatum*
2582	uncultured soil fungus	UDB067029	SH1179604.08FU	*G. applanatum*
2583	uncultured soil fungus	UDB0670319	SH1179604.08FU	*G. applanatum*
2584	uncultured soil fungus	UDB0670667	SH1179604.08FU	*G. applanatum*
2585	uncultured soil fungus	UDB0679640	SH1179604.08FU	*G. applanatum*
2586	uncultured soil fungus	UDB0680005	SH1179604.08FU	*G. applanatum*
2587	uncultured soil fungus	UDB0680531	SH1179604.08FU	*G. applanatum*
2588	uncultured soil fungus	UDB0680950	SH1179604.08FU	*G. applanatum*
2589	uncultured soil fungus	UDB0682224	SH1179604.08FU	*G. applanatum*
2590	uncultured soil fungus	UDB0686694	SH1179604.08FU	*G. applanatum*
2591	uncultured soil fungus	UDB0688255	SH1179604.08FU	*G. applanatum*
2592	uncultured soil fungus	UDB0690903	SH1179604.08FU	*G. applanatum*
2593	uncultured soil fungus	UDB069370	SH1179604.08FU	*G. applanatum*
2594	uncultured soil fungus	UDB0694220	SH1179604.08FU	*G. applanatum*
2595	uncultured soil fungus	UDB0699166	SH1179604.08FU	*G. applanatum*
2596	uncultured soil fungus	UDB070645	SH1179604.08FU	*G. applanatum*
2597	uncultured soil fungus	UDB0706520	SH1179604.08FU	*G. applanatum*
2598	uncultured soil fungus	UDB0710049	SH1179604.08FU	*G. applanatum*
2599	uncultured soil fungus	UDB0715464	SH1179604.08FU	*G. applanatum*
2600	uncultured soil fungus	UDB0729284	SH1179604.08FU	*G. applanatum*
2601	uncultured soil fungus	UDB074240	SH1179604.08FU	*G. applanatum*
2602	Fungi (plant leaf)	UDB0746367	SH1179604.08FU	*G. applanatum*
2603	Fungi (plant leaf)	UDB0746719	SH1179604.08FU	*G. applanatum*
2604	Fungi (plant leaf)	UDB0747059	SH1179604.08FU	*G. applanatum*
2605	Fungi (plant leaf)	UDB0747170	SH1179604.08FU	*G. applanatum*
2606	uncultured soil fungus	UDB074757	SH1179604.08FU	*G. applanatum*
2607	Fungi (plant leaf)	UDB0748354	SH1179604.08FU	*G. applanatum*
2608	Fungi (plant leaf)	UDB0749040	SH1179604.08FU	*G. applanatum*
2609	Fungi (plant leaf)	UDB0749357	SH1179604.08FU	*G. applanatum*
2610	Fungi (plant leaf)	UDB0749672	SH1179604.08FU	*G. applanatum*
2611	Fungi (plant leaf)	UDB0750344	SH1179604.08FU	*G. applanatum*
2612	Fungi (plant leaf)	UDB0751565	SH1179604.08FU	*G. applanatum*
2613	Fungi (plant leaf)	UDB0752347	SH1179604.08FU	*G. applanatum*
2614	Fungi (plant leaf)	UDB0752478	SH1179604.08FU	*G. applanatum*
2615	Fungi (plant leaf)	UDB0752676	SH1179604.08FU	*G. applanatum*
2616	Fungi (plant leaf)	UDB0752857	SH1179604.08FU	*G. applanatum*
2617	Fungi (plant leaf)	UDB0753016	SH1179604.08FU	*G. applanatum*
2618	uncultured soil fungus	UDB075512	SH1179604.08FU	*G. applanatum*
2619	uncultured soil fungus	UDB0756286	SH1179604.08FU	*G. applanatum*
2620	uncultured soil fungus	UDB0756506	SH1179604.08FU	*G. applanatum*
2621	uncultured soil fungus	UDB0756637	SH1179604.08FU	*G. applanatum*
2622	uncultured soil fungus	UDB0756810	SH1179604.08FU	*G. applanatum*
2623	uncultured soil fungus	UDB0758107	SH1179604.08FU	*G. applanatum*
2624	uncultured soil fungus	UDB0758114	SH1179604.08FU	*G. applanatum*

2625	uncultured soil fungus	UDB0758331	SH1179604.08FU	*G. applanatum*
2626	uncultured soil fungus	UDB0758396	SH1179604.08FU	*G. applanatum*
2627	uncultured soil fungus	UDB080030	SH1179604.08FU	*G. applanatum*
2628	uncultured soil fungus	UDB085254	SH1179604.08FU	*G. applanatum*
2629	uncultured soil fungus	UDB085994	SH1179604.08FU	*G. applanatum*
2630	uncultured soil fungus	UDB087835	SH1179604.08FU	*G. applanatum*
2631	uncultured soil fungus	UDB094231	SH1179604.08FU	*G. applanatum*
2632	uncultured soil fungus	UDB094616	SH1179604.08FU	*G. applanatum*
2633	uncultured soil fungus	UDB095181	SH1179604.08FU	*G. applanatum*
2634	uncultured soil fungus	UDB097422	SH1179604.08FU	*G. applanatum*
2635	uncultured soil fungus	UDB098207	SH1179604.08FU	*G. applanatum*
2636	uncultured soil fungus	UDB099583	SH1179604.08FU	*G. applanatum*
2637	uncultured *Ganoderma*	JX898575		*G. applanatum*
2638	uncultured soil fungus	UDB0240104	SH1179604.08FU	*G. applanatum*
2639	uncultured soil fungus	UDB0267759	SH1179604.08FU	*G. applanatum*
2640	uncultured soil fungus	UDB0374592	SH1179604.08FU	*G. applanatum*
2641	uncultured soil fungus	UDB0456937	SH1179604.08FU	*G. applanatum*
2642	uncultured soil fungus	UDB0492497	SH1179604.08FU	*G. applanatum*
2643	uncultured soil fungus	UDB0648395	SH1179604.08FU	*G. applanatum*
2644	uncultured soil fungus	UDB0650310	SH1179604.08FU	*G. applanatum*
2645	uncultured soil fungus	UDB0758712	SH1179604.08FU	*G. applanatum*
2646	uncultured soil fungus	UDB079462	SH1179604.08FU	*G. applanatum*
2647	*G. neojaponicum*	AY335163		*G. neojaponicum*
2648	*G. neojaponicum*	AY593866	SH1740446.08FU	*G. neojaponicum*
2649	*G. neojaponicum*	AY593867	SH1740448.08FU	*G. neojaponicum*
2650	*G. neojaponicum*	KT318596	SH1740455.08FU	*G. neojaponicum*
2651	*G. neojaponicum*	KT318598		*G. neojaponicum*
2652	*G. neojaponicum*	MK345443	SH2767715.08FU	*G. neojaponicum*
2653	*G. neojaponicum*	MK345444	SH1740455.08FU	*G. neojaponicum*
2654	*G. calidophilum*	MN398337		*G. neojaponicum*
2655	*G. calidophilum*	MN398338	SH1740455.08FU	*G. neojaponicum*
2656	*Ganoderma* sp.	MN871859		*G. neojaponicum*
2657	*Ganoderma* sp.	JN105713	SH1723189.08FU	*Ganoderma* sp. C1
2658	*Ganoderma* sp.	JN105714	SH1723198.08FU	*Ganoderma* sp. C1
2659	*G. aridicola*	KU572491/NR_152914	SH1723079.08FU	*G. aridicola*
2660	*Ganoderma* sp.	JN105707	SH1723079.08FU	*G. aridicola*
2661	*Ganoderma* sp.	JN105708	SH1723079.08FU	*G. aridicola*
2662	*Ganoderma* sp.	JN105709	SH1723079.08FU	*G. aridicola*
2663	*Ganoderma* sp.	JN105710	SH1723079.08FU	*G. aridicola*
2664	*Ganoderma* sp.	JN105711	SH1723079.08FU	*G. aridicola*
2665	*Ganoderma* sp.	JN105712	SH1723079.08FU	*G. aridicola*
2666	*Ganoderma* sp.	JN105715	SH1843092.08FU	*Ganoderma* sp. C2
2667	*Ganoderma* sp.	JN105716	SH1843092.08FU	*Ganoderma* sp. C2
2668	*Ganoderma* sp.	JN105717	SH2753096.08FU	*Ganoderma* sp. C2

2669	*G. enigmaticum*	KR014265	SH1723049.08FU	*G. enigmaticum - G. thailandicum*
2670	*G. enigmaticum*	KR150678	SH1723049.08FU	*G. enigmaticum - G. thailandicum*
2671	*G. enigmaticum*	KR183855/NR_132918/ NG_058156	SH1723049.08FU	*G. enigmaticum - G. thailandicum*
2672	*G. enigmaticum*	KU572486	SH1723049.08FU	*G. enigmaticum - G. thailandicum*
2673	*G. enigmaticum*	KU572487	SH1723049.08FU	*G. enigmaticum - G. thailandicum*
2674	*G. enigmaticum*	MH571697	SH1723049.08FU	*G. enigmaticum - G. thailandicum*
2675	*G. enigmaticum*	MK453308	SH1723049.08FU	*G. enigmaticum - G. thailandicum*
2676	*G. thailandicum*	MK848681		*G. enigmaticum - G. thailandicum*
2677	*G. thailandicum*	MK848682	SH1723049.08FU	*G. enigmaticum - G. thailandicum*
2678	uncultured soil fungus	UDB0767618	SH1723049.08FU	*G. enigmaticum - G. thailandicum*
2679	*G. casuarinicola*	MG279173/ NR_158432	SH1723049.08FU	*G. casuarinicola*
2680	*G. casuarinicola*	MG279174	SH1723049.08FU	*G. casuarinicola*
2681	*G. casuarinicola*	MG279175	SH1723049.08FU	*G. casuarinicola*
2682	*G. casuarinicola*	MG279176	SH1723049.08FU	*G. casuarinicola*
2683	*G. casuarinicola*	MK817650	SH1723049.08FU	*G. casuarinicola*
2684	*G. casuarinicola*	MT126492		*G. casuarinicola*
2685	*Ganoderma* sp.	AM773630	SH1723049.08FU	*G. casuarinicola*
2686	*Ganoderma* sp.	KC590320	SH1723049.08FU	*G. casuarinicola*
2687	*Ganoderma* sp.	KF998090	SH1723049.08FU	*G. casuarinicola*
2688	*Ganoderma* sp.	KF998096	SH1723049.08FU	*G. casuarinicola*
2689	*Ganoderma* sp.	KM229615	SH1723049.08FU	*G. casuarinicola*
2690	*Ganoderma* sp.	KM229616	SH1723049.08FU	*G. casuarinicola*
2691	*Ganoderma* sp.	KM229617	SH1723049.08FU	*G. casuarinicola*
2692	*Ganoderma* sp.	KM229618	SH1723049.08FU	*G. casuarinicola*
2693	*Ganoderma* sp.	KM229619	SH1723049.08FU	*G. casuarinicola*
2694	*Ganoderma* sp.	KM229620	SH1723049.08FU	*G. casuarinicola*
2695	*Ganoderma* sp.	KM229621	SH1723049.08FU	*G. casuarinicola*
2696	*Ganoderma* sp.	KM229622	SH1723049.08FU	*G. casuarinicola*
2697	*Ganoderma* sp.	KM229623	SH1723049.08FU	*G. casuarinicola*
2698	*Ganoderma* sp.	KM229624	SH1723049.08FU	*G. casuarinicola*
2699	*Ganoderma* sp.	KM229625	SH1723049.08FU	*G. casuarinicola*
2700	*Ganoderma* sp.	KM229626	SH1723049.08FU	*G. casuarinicola*
2701	*Ganoderma* sp.	KM229627	SH1723049.08FU	*G. casuarinicola*
2702	*Ganoderma* sp.	KM229628	SH1723049.08FU	*G. casuarinicola*
2703	*Ganoderma* sp.	KM229629	SH1723049.08FU	*G. casuarinicola*
2704	*Ganoderma* sp.	KM229630	SH1723049.08FU	*G. casuarinicola*
2705	*Ganoderma* sp.	KM229631	SH1723049.08FU	*G. casuarinicola*
2706	*Ganoderma* sp.	KM229632	SH1723049.08FU	*G. casuarinicola*
2707	*Ganoderma* sp.	KM229633	SH1723049.08FU	*G. casuarinicola*
2708	*Ganoderma* sp.	KP780431	SH1723049.08FU	*G. casuarinicola*

2709	*Ganoderma* sp.	KR154917	SH1723049.08FU	*G. casuarinicola*
2710	*Ganoderma* sp.	KR154951	SH1723049.08FU	*G. casuarinicola*
2711	*Ganoderma* sp.	KR154957	SH1723049.08FU	*G. casuarinicola*
2712	*Ganoderma* sp.	KR155019	SH1723049.08FU	*G. casuarinicola*
2713	*Ganoderma* sp.	KR155071	SH1723049.08FU	*G. casuarinicola*
2714	*G. carnosum*	KR733545	SH1723049.08FU	*G. casuarinicola*
2715	*Ganoderma* sp.	KR818818	SH1723049.08FU	*G. casuarinicola*
2716	*Ganoderma* sp.	KT186191		*G. casuarinicola*
2717	*Ganoderma* sp.	KT186194	SH1723049.08FU	*G. casuarinicola*
2718	*Ganoderma* sp.	KT188601	SH1723268.08FU	*G. casuarinicola*
2719	*Ganoderma* sp.	KT188602	SH1723268.08FU	*G. casuarinicola*
2720	*Ganoderma* sp.	KT188604	SH1723049.08FU	*G. casuarinicola*
2721	*Ganoderma* sp.	KT188605	SH1723049.08FU	*G. casuarinicola*
2722	uncultured *Ganoderma*	KU712536	SH1723049.08FU	*G. casuarinicola*
2723	*G. enigmaticum*	KU870313	SH1723049.08FU	*G. casuarinicola*
2724	*Ganoderma* sp.	KU886297	SH1723049.08FU	*G. casuarinicola*
2725	*Ganoderma* sp.	KY009866	SH1723245.08FU	*G. casuarinicola*
2726	*Ganoderma* sp.	KY111250	SH1723170.08FU	*G. casuarinicola*
2727	*Ganoderma* sp.	KY111251	SH1723049.08FU	*G. casuarinicola*
2728	uncultured fungus	KY978282	SH1723049.08FU	*G. casuarinicola*
2729	uncultured fungus	KY978295	SH1723049.08FU	*G. casuarinicola*
2730	*Ganoderma* sp.	MH023509	SH1723049.08FU	*G. casuarinicola*
2731	*Ganoderma* sp.	MK681870	SH1723049.08FU	*G. casuarinicola*
2732	*G. enigmaticum*	MT229201		*G. casuarinicola*
2733	*G. enigmaticum*	MT364486		*G. casuarinicola*
2734	*G. enigmaticum*	MT364487		*G. casuarinicola*
2735	*Ganoderma* sp.	HQ689698	SH1677216.08FU	*G. casuarinicola*
2736	*G. lucidum*	KF998091		*G. casuarinicola*
2737	*Ganoderma* sp.	KR155026		*G. casuarinicola*
2738	*Ganoderma* sp.	KR155123	SH1851024.08FU	*G. casuarinicola*
2739	*G. applanatum*	KT185664	SH1723175.08FU	*G. casuarinicola*
2740	*Ganoderma* sp.	KX957800		*G. casuarinicola*
2741	*Ganoderma* sp.	MN784439		*G. casuarinicola*
2742	*G. mbrekobenum*	KJ510532	SH1723058.08FU	*G. mbrekobenum*
2743	*G. mbrekobenum*	KX000896/NR_147647	SH1723058.08FU	*G. mbrekobenum*
2744	*G. mbrekobenum*	KX000898	SH1723058.08FU	*G. mbrekobenum*
2745	*G. mbrekobenum*	KY865253	SH1723058.08FU	*G. mbrekobenum*
2746	*G. mbrekobenum*	MH221092	SH1723058.08FU	*G. mbrekobenum*
2747	*G. mbrekobenum*	MK453307	SH1723058.08FU	*G. mbrekobenum*
2748	*G. mbrekobenum*	MK940286		*G. mbrekobenum*
2749	*G. mbrekobenum*	MK940287		*G. mbrekobenum*
2750	*G. mbrekobenum*	MK940289	SH1723058.08FU	*G. mbrekobenum*
2751	*G. mbrekobenum*	MK940290	SH1723058.08FU	*G. mbrekobenum*
2752	*G. mbrekobenum*	MN097540	SH1723058.08FU	*G. mbrekobenum*
2753	*Ganoderma* sp.	KM229599	SH1723058.08FU	*G. mbrekobenum*

2754	*Ganoderma* sp.	KM229600	SH1723058.08FU	*G. mbrekobenum*
2755	*Ganoderma* sp.	KM229601	SH1723058.08FU	*G. mbrekobenum*
2756	*Ganoderma* sp.	KM229602	SH1723058.08FU	*G. mbrekobenum*
2757	*Ganoderma* sp.	KM229603	SH1743714.08FU	*G. mbrekobenum*
2758	*Ganoderma* sp.	KM229604	SH1723110.08FU	*G. mbrekobenum*
2759	*Ganoderma* sp.	KM229605	SH1723269.08FU	*G. mbrekobenum*
2760	*Ganoderma* sp.	KM229606	SH1723058.08FU	*G. mbrekobenum*
2761	*Ganoderma* sp.	KM229607	SH1723058.08FU	*G. mbrekobenum*
2762	*Ganoderma* sp.	KM229609	SH1723058.08FU	*G. mbrekobenum*
2763	*Ganoderma* sp.	KM229610	SH1723058.08FU	*G. mbrekobenum*
2764	*Ganoderma* sp.	KM229611	SH1723058.08FU	*G. mbrekobenum*
2765	*Ganoderma* sp.	KM229612	SH1723058.08FU	*G. mbrekobenum*
2766	*Ganoderma* sp.	KM229613	SH1723058.08FU	*G. mbrekobenum*
2767	*Ganoderma* sp.	KM229614	SH1723058.08FU	*G. mbrekobenum*
2768	*G. applanatum*	KP794597	SH1723110.08FU	*G. mbrekobenum*
2769	*G. tsugae*	KP794598	SH1723110.08FU	*G. mbrekobenum*
2770	*G. carnosum*	KP943501	SH1723110.08FU	*G. mbrekobenum*
2771	*Ganoderma* sp.	LN774971	SH1723058.08FU	*G. mbrekobenum*
2772	*Ganoderma* sp.	MH221093	SH1723058.08FU	*G. mbrekobenum*
2773	*Ganoderma* sp.	KJ510533	SH1677219.08FU	*G. mbrekobenum*
2774	*Ganoderma* sp.	KM229608	SH1723235.08FU	*G. mbrekobenum*
2775	*Ganoderma* sp.	KR154954	SH1743705.08FU	*G. mbrekobenum*
2776	*G. lucidum*	KT210090	SH1723212.08FU	*G. mbrekobenum*
2777	*Ganoderma* sp.	MF614913	SH1723058.08FU	*G. mbrekobenum*
2778	*G. nasalanense*	MK345441/NR_164048		*G. nasalanense*
2779	*G. nasalanense*	MK345442	SH1723131.08FU	*G. nasalanense*
2780	*Ganoderma* sp.	KR093030	SH1723131.08FU	*G. nasalanense*
2781	*Ganoderma* sp.	KT965500	SH1723303.08FU	*G. nasalanense*
2782	*Ganoderma* sp.	KT965501	SH1723131.08FU	*G. nasalanense*
2783	*G. australe*	LC084677	SH1723131.08FU	*G. nasalanense*
2784	*G. australe*	LC084678	SH1723131.08FU	*G. nasalanense*
2785	*G. australe*	LC084692	SH1723131.08FU	*G. nasalanense*
2786	*G. australe*	LC084707		*G. nasalanense*
2787	*G. australe*	LC084722	SH1723131.08FU	*G. nasalanense*
2788	*G. australe*	LC084729	SH1723131.08FU	*G. nasalanense*
2789	*G. australe*	LC084731	SH1723131.08FU	*G. nasalanense*
2790	*G. australe*	LC084737	SH1723131.08FU	*G. nasalanense*
2791	uncultured soil fungus	UDB0760488	SH1723131.08FU	*G. nasalanense*
2792	uncultured soil fungus	UDB0767030	SH1723131.08FU	*G. nasalanense*
2793	*Ganoderma* sp.	KT965502	SH1730468.08FU	*G. nasalanense*
2794	*G. australe*	LC084740	SH2762643.08FU	*G. nasalanense*
2795	*G. sinense*	DQ424982		*G. sinense*
2796	*G. sinense*	DQ424990		*G. sinense*
2797	*G. sinense*	DQ424995	SH1723061.08FU	*G. sinense*
2798	*G. sinense*	DQ425014	SH1723061.08FU	*G. sinense*

2799	*G. sinense*	HQ235633		*G. sinense*
2800	*G. sinense*	HQ235634	SH1723061.08FU	*G. sinense*
2801	*G. sinense*	KC415760	SH1723061.08FU	*G. sinense*
2802	*G. sinense*	KF494998	SH1723061.08FU	*G. sinense*
2803	*G. sinense*	KM249933	SH1723061.08FU	*G. sinense*
2804	*G. sinense*	KT318604	SH1723277.08FU	*G. sinense*
2805	*G. sinense*	KT906369	SH1723061.08FU	*G. sinense*
2806	*G. sinense*	MG279193	SH1723061.08FU	*G. sinense*
2807	*G. sinense*	MH106882	SH1723061.08FU	*G. sinense*
2808	*G. sinense*	MH294316	SH1723061.08FU	*G. sinense*
2809	*G. sinense*	MH294331	SH1723061.08FU	*G. sinense*
2810	*G. sinense*	MK172819	SH1723061.08FU	*G. sinense*
2811	*G. sinense*	MK313110	SH1723061.08FU	*G. sinense*
2812	*G. sinense*	MK313111	SH1723061.08FU	*G. sinense*
2813	*G. sinense*	MK313112	SH1723061.08FU	*G. sinense*
2814	*G. sinense*	MK313113	SH1723061.08FU	*G. sinense*
2815	*G. sinense*	MK313114	SH1723277.08FU	*G. sinense*
2816	*G. sinense*	MK313115	SH1723061.08FU	*G. sinense*
2817	*G. sinense*	MK313116	SH1723061.08FU	*G. sinense*
2818	*G. sinense*	MK313117	SH1723061.08FU	*G. sinense*
2819	*G. sinense*	MK313118	SH1723061.08FU	*G. sinense*
2820	*G. sinense*	MK313119	SH1723061.08FU	*G. sinense*
2821	*G. sinense*	MK313120	SH1723061.08FU	*G. sinense*
2822	*G. sinense*	MK313121	SH1723061.08FU	*G. sinense*
2823	*G. sinense*	MK313122	SH1723061.08FU	*G. sinense*
2824	*G. sinense*	MK313123	SH1723061.08FU	*G. sinense*
2825	*G. sinense*	MK313124	SH1723061.08FU	*G. sinense*
2826	*G. sinense*	MK313125	SH1723061.08FU	*G. sinense*
2827	*G. sinense*	MK313126	SH1723277.08FU	*G. sinense*
2828	*G. sinense*	MK313127	SH1723277.08FU	*G. sinense*
2829	*G. sinense*	MK313128	SH1723061.08FU	*G. sinense*
2830	*G. sinense*	MK345452	SH1723061.08FU	*G. sinense*
2831	*G. sinense*	MK345453	SH1723061.08FU	*G. sinense*
2832	*G. sinense*	MK345454	SH1723061.08FU	*G. sinense*
2833	*G. sinense*	MK968730	SH1723061.08FU	*G. sinense*
2834	*G. sinense*	MN398316		*G. sinense*
2835	*G. sinense*	MN398317		*G. sinense*
2836	*G. sinense*	MN398318	SH1723061.08FU	*G. sinense*
2837	*G. sinense*	MN398319		*G. sinense*
2838	*G. sinense*	MN398320		*G. sinense*
2839	*G. sinense*	Z37066/Z37103		*G. sinense*
2840	*G. lucidum*	AF506373	SH1723061.08FU	*G. sinense*
2841	*G. japonicum*	AY593864	SH1723061.08FU	*G. sinense*
2842	*G. japonicum*	AY593865	SH1723061.08FU	*G. sinense*
2843	*G. japonicum*	GU213475	SH1723061.08FU	*G. sinense*

2844	*G. lucidum*	GU213482	SH1723061.08FU	*G. sinense*
2845	*Ganoderma* sp.	JQ520212	SH1723061.08FU	*G. sinense*
2846	*Ganoderma* sp.	JQ520213	SH1723061.08FU	*G. sinense*
2847	*G. atrum*	JQ886403	SH1723061.08FU	*G. sinense*
2848	*G. japonicum*	KX055529	SH1723277.08FU	*G. sinense*
2849	*G. lucidum*	KX262895	SH1723061.08FU	*G. sinense*
2850	*G. lucidum*	KX262900	SH1723061.08FU	*G. sinense*
2851	*G. lucidum*	KX262901	SH1723061.08FU	*G. sinense*
2852	*Ganoderma* sp.	MG282563		*G. sinense*
2853	*Ganoderma* sp.	MH294302		*G. sinense*
2854	*Ganoderma* sp.	MH294303	SH1723061.08FU	*G. sinense*
2855	*Ganoderma* sp.	MH294313	SH1723061.08FU	*G. sinense*
2856	*Ganoderma* sp.	MH294319	SH1723061.08FU	*G. sinense*
2857	*Ganoderma* sp.	MH294332	SH1723061.08FU	*G. sinense*
2858	*G. subresinosum*	MN398321		*G. sinense*
2859	*G. subresinosum*	MN398322	SH1723061.08FU	*G. sinense*
2860	*G. formosanum*	X78752/X78773		*G. sinense*
2861	*G. cupreum*	JN105701	SH1678458.08FU	*G. cupreum*
2862	*G. cupreum*	JN105702	SH1723122.08FU	*G. cupreum*
2863	*G. cupreum*	KX055557	SH1678458.08FU	*G. cupreum*
2864	*G. cupreum*	KX055560	SH1723122.08FU	*G. cupreum*
2865	uncultured fungus	AB828214	SH1678458.08FU	*G. cupreum*
2866	*G. australe*	LC084685	SH1723122.08FU	*G. cupreum*
2867	*G. chalceum*	LK022294	SH1723122.08FU	*G. cupreum*
2868	*G.* cf. *cupreum*	MH571696	SH1678458.08FU	*G. cupreum*
2869	*G. subfornicatum*	JX082352	SH1723089.08FU	*G. subfornicatum*
2870	*G. ecuadoriense*	KU128524		*G. subfornicatum*
2871	*G. ecuadoriense*	KU128525		*G. subfornicatum*
2872	*G. ecuadoriense*	KU128526	SH1723089.08FU	*G. subfornicatum*
2873	*G. ecuadoriense*	MK119827		*G. subfornicatum*
2874	*G. ecuadoriense*	MK119828		*G. subfornicatum*
2875	uncultured fungus	KJ411557	SH1723089.08FU	*G. subfornicatum*
2876	*Ganoderma* sp.	KJ832060	SH1723089.08FU	*G. subfornicatum*
2877	*Ganoderma* sp.	MH267948	SH1723089.08FU	*G. subfornicatum*
2878	*G. orbiforme*	JX310813	SH1723108.08FU	*G. orbiforme*
2879	*G. orbiforme*	JX310814	SH1723108.08FU	*G. orbiforme*
2880	*G. orbiforme*	JX310815	SH1723108.08FU	*G. orbiforme*
2881	*G. orbiforme*	JX310816	SH1723108.08FU	*G. orbiforme*
2882	*G. orbiforme*	MK119829		*G. orbiforme*
2883	*G. orbiforme*	MT364488		*G. orbiforme*
2884	*G. mastoporum*	AJ627585	SH1723055.08FU	*G. mastoporum*
2885	*G. mastoporum*	GU213486	SH1723055.08FU	*G. mastoporum*
2886	*G. mastoporum*	JN643730	SH1723055.08FU	*G. mastoporum*
2887	*G. mastoporum*	JQ409361	SH1723055.08FU	*G. mastoporum*
2888	*G. mastoporum*	JX195201	SH1723055.08FU	*G. mastoporum*

2889	*G. mastoporum*	JX840350	SH1723055.08FU	*G. mastoporum*
2890	*G. mastoporum*	JX840351	SH1723055.08FU	*G. mastoporum*
2891	*G. mastoporum*	JX840352		*G. mastoporum*
2892	*G. mastoporum*	MF680427	SH1723055.08FU	*G. mastoporum*
2893	*G. mastoporum*	MF680428	SH1723055.08FU	*G. mastoporum*
2894	*G. mastoporum*	MG448604	SH1723055.08FU	*G. mastoporum*
2895	*G. mastoporum*	MK968732	SH1723248.08FU	*G. mastoporum*
2896	*Ganoderma* sp.	AJ537399	SH1723055.08FU	*G. mastoporum*
2897	*Ganoderma* sp.	AJ537401	SH1723055.08FU	*G. mastoporum*
2898	*G. cupreum*	AJ627586/7		*G. mastoporum*
2899	*G. cupreum*	AJ627588/9		*G. mastoporum*
2900	*G. cupreum*	AY569450	SH1723055.08FU	*G. mastoporum*
2901	*G. cupreum*	FJ655466		*G. mastoporum*
2902	*G. cupreum*	FJ655467	SH1723055.08FU	*G. mastoporum*
2903	*G. cupreum*	FJ655468		*G. mastoporum*
2904	*G. cupreum*	FJ655469	SH1723055.08FU	*G. mastoporum*
2905	*G. cupreum*	FJ655470	SH1723055.08FU	*G. mastoporum*
2906	*G. cupreum*	JN596328	SH1723152.08FU	*G. mastoporum*
2907	*G. cupreum*	JN596329	SH1723152.08FU	*G. mastoporum*
2908	*G. orbiforme*	JX840345	SH1723055.08FU	*G. mastoporum*
2909	*G. orbiforme*	JX840346	SH1723248.08FU	*G. mastoporum*
2910	*G. fornicatum*	JX840347	SH1723055.08FU	*G. mastoporum*
2911	*G. fornicatum*	JX840348	SH1723055.08FU	*G. mastoporum*
2912	*G. fornicatum*	JX840349	SH1723055.08FU	*G. mastoporum*
2913	*Ganoderma* sp.	KR709152		*G. mastoporum*
2914	*G. orbiforme*	KT318599	SH1723055.08FU	*G. mastoporum*
2915	*Ganoderma* sp.	KT965495	SH1723055.08FU	*G. mastoporum*
2916	*G. australe*	LC084660	SH1723055.08FU	*G. mastoporum*
2917	*G. australe*	LC084662	SH1723055.08FU	*G. mastoporum*
2918	*G. australe*	LC084665	SH1723055.08FU	*G. mastoporum*
2919	*G. australe*	LC084666	SH1723055.08FU	*G. mastoporum*
2920	*G. australe*	LC084668	SH1723055.08FU	*G. mastoporum*
2921	*G. australe*	LC084669	SH1723055.08FU	*G. mastoporum*
2922	*G. australe*	LC084670		*G. mastoporum*
2923	*G. australe*	LC084671		*G. mastoporum*
2924	*G. australe*	LC084672	SH1723055.08FU	*G. mastoporum*
2925	*G. australe*	LC084673	SH1723055.08FU	*G. mastoporum*
2926	*G. australe*	LC084674	SH1723055.08FU	*G. mastoporum*
2927	*G. australe*	LC084675	SH1723055.08FU	*G. mastoporum*
2928	*G. australe*	LC084676	SH1723055.08FU	*G. mastoporum*
2929	*G. australe*	LC084679	SH1723055.08FU	*G. mastoporum*
2930	*G. australe*	LC084681	SH1723055.08FU	*G. mastoporum*
2931	*G. australe*	LC084682	SH1723055.08FU	*G. mastoporum*
2932	*G. australe*	LC084683	SH1723055.08FU	*G. mastoporum*
2933	*G. australe*	LC084684	SH1723055.08FU	*G. mastoporum*

2934	*G. australe*	LC084686	SH1723055.08FU	*G. mastoporum*
2935	*G. australe*	LC084687	SH1723055.08FU	*G. mastoporum*
2936	*G. australe*	LC084688	SH1723055.08FU	*G. mastoporum*
2937	*G. australe*	LC084689		*G. mastoporum*
2938	*G. australe*	LC084690	SH1723055.08FU	*G. mastoporum*
2939	*G. australe*	LC084693	SH1723055.08FU	*G. mastoporum*
2940	*G. australe*	LC084694	SH1723055.08FU	*G. mastoporum*
2941	*G. australe*	LC084695		*G. mastoporum*
2942	*G. australe*	LC084697		*G. mastoporum*
2943	*G. australe*	LC084698	SH1723055.08FU	*G. mastoporum*
2944	*G. australe*	LC084699	SH1723055.08FU	*G. mastoporum*
2945	*G. australe*	LC084700		*G. mastoporum*
2946	*G. australe*	LC084701	SH1723055.08FU	*G. mastoporum*
2947	*G. australe*	LC084702	SH1723055.08FU	*G. mastoporum*
2948	*G. australe*	LC084703		*G. mastoporum*
2949	*G. australe*	LC084704		*G. mastoporum*
2950	*G. australe*	LC084705		*G. mastoporum*
2951	*G. australe*	LC084708	SH1723055.08FU	*G. mastoporum*
2952	*G. australe*	LC084709	SH1723055.08FU	*G. mastoporum*
2953	*G. australe*	LC084710	SH1723055.08FU	*G. mastoporum*
2954	*G. australe*	LC084712	SH1723055.08FU	*G. mastoporum*
2955	*G. australe*	LC084713		*G. mastoporum*
2956	*G. australe*	LC084714	SH1723055.08FU	*G. mastoporum*
2957	*G. australe*	LC084716	SH1723055.08FU	*G. mastoporum*
2958	*G. australe*	LC084719	SH1723055.08FU	*G. mastoporum*
2959	*G. australe*	LC084720	SH1723055.08FU	*G. mastoporum*
2960	*G. australe*	LC084723	SH1723055.08FU	*G. mastoporum*
2961	*G. australe*	LC084724	SH1723055.08FU	*G. mastoporum*
2962	*G. australe*	LC084730		*G. mastoporum*
2963	*G. australe*	LC084732	SH1723055.08FU	*G. mastoporum*
2964	*G. australe*	LC084733	SH1723055.08FU	*G. mastoporum*
2965	*G. australe*	LC084734	SH1723055.08FU	*G. mastoporum*
2966	*G. australe*	LC084735	SH1723055.08FU	*G. mastoporum*
2967	*G. australe*	LC084738	SH1723055.08FU	*G. mastoporum*
2968	*G. australe*	LC084741	SH1723055.08FU	*G. mastoporum*
2969	*G. australe*	LC084743	SH1723055.08FU	*G. mastoporum*
2970	*G. australe*	LC084744		*G. mastoporum*
2971	*G. australe*	LC084745	SH1723055.08FU	*G. mastoporum*
2972	*G. australe*	LC084746	SH1723055.08FU	*G. mastoporum*
2973	*G. australe*	LC084748	SH1723055.08FU	*G. mastoporum*
2974	*G. australe*	LC084751	SH1723055.08FU	*G. mastoporum*
2975	*G. orbiforme*	MG279186	SH1723055.08FU	*G. mastoporum*
2976	*G. orbiforme*	MG279187	SH1723055.08FU	*G. mastoporum*
2977	*G. orbiforme*	MH106874	SH1723055.08FU	*G. mastoporum*
2978	*G. orbiforme*	MH106875	SH1723055.08FU	*G. mastoporum*

2979	*G. orbiforme*	MH106876		*G. mastoporum*
2980	*G. orbiforme*	MH106877	SH1723055.08FU	*G. mastoporum*
2981	*G. orbiforme*	MH106878	SH1723055.08FU	*G. mastoporum*
2982	*Ganoderma* sp.	MK131241	SH1723055.08FU	*G. mastoporum*
2983	*G. orbiforme*	MK313108	SH1723248.08FU	*G. mastoporum*
2984	*G. orbiforme*	MK313109	SH1723055.08FU	*G. mastoporum*
2985	*G. orbiforme*	MK345445	SH1723055.08FU	*G. mastoporum*
2986	*G. orbiforme*	MK345446	SH1723248.08FU	*G. mastoporum*
2987	*G. orbiforme*	MK345447	SH1723055.08FU	*G. mastoporum*
2988	*G. orbiforme*	MK345448	SH1723248.08FU	*G. mastoporum*
2989	*G. orbiforme*	MK345449	SH1723248.08FU	*G. mastoporum*
2990	*Ganoderma* sp.	MK589271		*G. mastoporum*
2991	*Ganoderma* sp.	MK589273		*G. mastoporum*
2992	*Ganoderma* sp.	MK589274		*G. mastoporum*
2993	*Ganoderma* sp.	MK589275		*G. mastoporum*
2994	*Ganoderma* sp.	MK589276		*G. mastoporum*
2995	*Ganoderma* sp.	MK589277		*G. mastoporum*
2996	*G. orbiforme*	MN401408		*G. mastoporum*
2997	*G. orbiforme*	MN401409	SH1723055.08FU	*G. mastoporum*
2998	*G. australe*	MT364483		*G. mastoporum*
2999	uncultured soil fungus	UDB039638	SH1723248.08FU	*G. mastoporum*
3000	uncultured soil fungus	UDB0767350	SH1723055.08FU	*G. mastoporum*
3001	uncultured soil fungus	UDB0767363	SH1723055.08FU	*G. mastoporum*
3002	uncultured soil fungus	UDB0767369	SH1723055.08FU	*G. mastoporum*
3003	uncultured soil fungus	UDB0767381	SH1723055.08FU	*G. mastoporum*
3004	uncultured soil fungus	UDB0767392	SH1723055.08FU	*G. mastoporum*
3005	*G. multicornum*	MT772000		*G. mastoporum*
3006	*G. mastoporum*	JN596330		*G. mastoporum*
3007	*G. angustisporum*	MG279170/NR_158431	SH1740439.08FU	*G. angustisporum*
3008	*G. angustisporum*	MG279171	SH1740424.08FU	*G. angustisporum*
3009	*G. angustisporum*	MG279172	SH1740424.08FU	*G. angustisporum*
3010	*Ganoderma* sp.	AY569452	SH1740452.08FU	*G. angustisporum*
3011	*Ganoderma* sp.	KJ654552	SH1740424.08FU	*G. angustisporum*
3012	*G. australe*	LC084664	SH1740424.08FU	*G. angustisporum*
3013	*G. australe*	LC084711	SH1740424.08FU	*G. angustisporum*
3014	*G. australe*	LC084715	SH1740424.08FU	*G. angustisporum*
3015	*G. australe*	LC084718	SH1740424.08FU	*G. angustisporum*
3016	*G. australe*	LC084728	SH1740424.08FU	*G. angustisporum*
3017	*G. australe*	LC084736	SH1740424.08FU	*G. angustisporum*
3018	*G. australe*	LC084739	SH1740424.08FU	*G. angustisporum*
3019	*G. australe*	LC084747	SH1740424.08FU	*G. angustisporum*
3020	*Ganoderma* sp.	MF072394	SH1740424.08FU	*G. angustisporum*
3021	*Ganoderma* sp.	MT449082		*G. angustisporum*
3022	*Ganoderma* sp.	JN596322	SH1740447.08FU	*G. angustisporum*
3023	*G. applanatum*	KY449369	SH1740449.08FU	*Ganoderma* sp. D1

3024	*G. applanatum*	KY449370	SH1740450.08FU	*Ganoderma* sp. D1
3025	*G. zonatum*	KF605678	SH1723053.08FU	*G. zonatum*
3026	*G. zonatum*	KF605679	SH1723053.08FU	*G. zonatum*
3027	*G. zonatum*	KJ143921		*G. zonatum*
3028	*G. zonatum*	KJ143922	SH1723053.08FU	*G. zonatum*
3029	*G. zonatum*	KX853436		*G. zonatum*
3030	*G. zonatum*	KX853437		*G. zonatum*
3031	*G. zonatum*	KX853438		*G. zonatum*
3032	*G. zonatum*	KX853439		*G. zonatum*
3033	*G. zonatum*	KX853440		*G. zonatum*
3034	*G. zonatum*	KX853441		*G. zonatum*
3035	*G. zonatum*	KX853442		*G. zonatum*
3036	*G. zonatum*	KX853443		*G. zonatum*
3037	*G. zonatum*	KX853444		*G. zonatum*
3038	*G. zonatum*	KX853445		*G. zonatum*
3039	*G. zonatum*	KX853446		*G. zonatum*
3040	*G. zonatum*	KX853447		*G. zonatum*
3041	*G. zonatum*	KX853448		*G. zonatum*
3042	*G. zonatum*	KX853449		*G. zonatum*
3043	*G. zonatum*	KX853450		*G. zonatum*
3044	*G. zonatum*	KX853451		*G. zonatum*
3045	*G. zonatum*	KX853452		*G. zonatum*
3046	*G. zonatum*	KX853453		*G. zonatum*
3047	*G. zonatum*	KX853454		*G. zonatum*
3048	*G. zonatum*	KX853455		*G. zonatum*
3049	*G. zonatum*	KX853456		*G. zonatum*
3050	*G. zonatum*	KX853457		*G. zonatum*
3051	*G. zonatum*	KX853458	SH1723053.08FU	*G. zonatum*
3052	*G. zonatum*	KX853459	SH1723053.08FU	*G. zonatum*
3053	*G. zonatum*	KY646211	SH1723053.08FU	*G. zonatum*
3054	*G. zonatum*	KY646212	SH1723053.08FU	*G. zonatum*
3055	*G. zonatum*	KY708886	SH1723053.08FU	*G. zonatum*
3056	*G. zonatum*	MG654374	SH1723053.08FU	*G. zonatum*
3057	*G. zonatum*	MG654375	SH1723053.08FU	*G. zonatum*
3058	*G. zonatum*	MG654376	SH1723053.08FU	*G. zonatum*
3059	*G. zonatum*	MG654377		*G. zonatum*
3060	*G. zonatum*	MG654378		*G. zonatum*
3061	*G. zonatum*	MG654379		*G. zonatum*
3062	*G. zonatum*	MG654380		*G. zonatum*
3063	*G. zonatum*	MG654381		*G. zonatum*
3064	*G. zonatum*	MG654382		*G. zonatum*
3065	*G. zonatum*	MG654383		*G. zonatum*
3066	*G. zonatum*	MG654384	SH1723053.08FU	*G. zonatum*
3067	*G. zonatum*	MG654385		*G. zonatum*
3068	*G. zonatum*	MG654386		*G. zonatum*

3069	*G. zonatum*	MG654387	SH1723053.08FU	*G. zonatum*
3070	*G. zonatum*	MG654388		*G. zonatum*
3071	*G. zonatum*	MG654389		*G. zonatum*
3072	*G. zonatum*	MG654390		*G. zonatum*
3073	*G. zonatum*	MG654391		*G. zonatum*
3074	*G. zonatum*	MG654392		*G. zonatum*
3075	*G. zonatum*	MG654393	SH1723053.08FU	*G. zonatum*
3076	*G. zonatum*	MG654394		*G. zonatum*
3077	*G. zonatum*	MG654395		*G. zonatum*
3078	*G. zonatum*	MG654396		*G. zonatum*
3079	*G. zonatum*	MG654397		*G. zonatum*
3080	*G. zonatum*	MG654398		*G. zonatum*
3081	*G. zonatum*	MG654399	SH1723053.08FU	*G. zonatum*
3082	*G. zonatum*	MG654400		*G. zonatum*
3083	*G. zonatum*	MG654401		*G. zonatum*
3084	*G. zonatum*	MG654402		*G. zonatum*
3085	*G. zonatum*	MG654403		*G. zonatum*
3086	*G. zonatum*	MG654404		*G. zonatum*
3087	*G. zonatum*	MG654405		*G. zonatum*
3088	*G. zonatum*	MG654406		*G. zonatum*
3089	*G. zonatum*	MG654407		*G. zonatum*
3090	*G. zonatum*	MG654408		*G. zonatum*
3091	*G. zonatum*	MG654409		*G. zonatum*
3092	*G. zonatum*	MG654410		*G. zonatum*
3093	*G. zonatum*	MG654411		*G. zonatum*
3094	*G. zonatum*	MG654412	SH1723053.08FU	*G. zonatum*
3095	*G. zonatum*	MG654413		*G. zonatum*
3096	*G. zonatum*	MG654414		*G. zonatum*
3097	*G. zonatum*	MG654415	SH1723053.08FU	*G. zonatum*
3098	*G. zonatum*	MG654416		*G. zonatum*
3099	*G. zonatum*	MG654417	SH1723053.08FU	*G. zonatum*
3100	*G. zonatum*	MG654418	SH1723053.08FU	*G. zonatum*
3101	*G. zonatum*	MG654419	SH1723053.08FU	*G. zonatum*
3102	*G. zonatum*	MG654420	SH1723053.08FU	*G. zonatum*
3103	*G. zonatum*	MG654421	SH1723053.08FU	*G. zonatum*
3104	*G. zonatum*	MG654422	SH1723053.08FU	*G. zonatum*
3105	*G. zonatum*	MG654423	SH1723053.08FU	*G. zonatum*
3106	*G. zonatum*	MG654424	SH1723053.08FU	*G. zonatum*
3107	*G. zonatum*	MG654425	SH1723053.08FU	*G. zonatum*
3108	*G. zonatum*	MG654426	SH1723053.08FU	*G. zonatum*
3109	*Ganoderma* sp.	KJ792083	SH1723113.08FU	*Ganoderma* sp. D2
3110	*Ganoderma* sp.	KJ792084	SH1723113.08FU	*Ganoderma* sp. D2
3111	*Ganoderma* sp.	KJ792085	SH1723113.08FU	*Ganoderma* sp. D2
3112	*Ganoderma* sp.	KJ792086	SH1723113.08FU	*Ganoderma* sp. D2
3113	*G. ryvardenii*	HM138670	SH1723066.08FU	*G. ryvardenii*

3114	*G. ryvardenii*	HM138671	SH1723066.08FU	*G. ryvardenii*
3115	*G. ryvardenii*	HM138672	SH1723066.08FU	*G. ryvardenii*
3116	*G. ryvardenii*	JN105691	SH1723066.08FU	*G. ryvardenii*
3117	*G. ryvardenii*	JN105692	SH1723066.08FU	*G. ryvardenii*
3118	*G. ryvardenii*	JN105693	SH1723066.08FU	*G. ryvardenii*
3119	*G. ryvardenii*	JN105694	SH1723066.08FU	*G. ryvardenii*
3120	*G. ryvardenii*	JN105695	SH1723066.08FU	*G. ryvardenii*
3121	*G. ryvardenii*	JN105696	SH1723066.08FU	*G. ryvardenii*
3122	*G. ryvardenii*	JN105697	SH1723066.08FU	*G. ryvardenii*
3123	*G. ryvardenii*	JN105698	SH1723066.08FU	*G. ryvardenii*
3124	*G. ryvardenii*	JN105699	SH1723066.08FU	*G. ryvardenii*
3125	*G. ryvardenii*	JN105706	SH1723247.08FU	*G. ryvardenii*
3126	*Ganoderma* sp.	LK022295	SH1723066.08FU	*G. ryvardenii*
3127	*Ganoderma* sp.	MN784437		*G. ryvardenii*
3128	*Ganoderma* sp.	MN784438		*G. ryvardenii*
3129	*G. ryvardenii*	JN105700	SH1723278.08FU	*G. ryvardenii*
3130	*Ganoderma* sp.	KY963354	SH1723304.08FU	*G. ryvardenii*
3131	*Ganoderma* sp.	MK681871		*G. ryvardenii*
3132	*Ganoderma* sp.	MK681872		*G. ryvardenii*
3133	*G. ryvardenii*	MN784436		*G. ryvardenii*
3134	*G. wiiroense*	MN809325		*G. ryvardenii*
3135	*G. boninense*	AB985729	SH1723050.08FU	*G. boninense*
3136	*G. boninense*	AF255196/7		*G. boninense*
3137	*G. boninense*	BD082757		*G. boninense*
3138	*G. boninense*	BD082758		*G. boninense*
3139	*G. boninense*	BD082759		*G. boninense*
3140	*G. boninense*	KF164430	SH1723050.08FU	*G. boninense*
3141	*G. boninense*	KJ143905	SH1723050.08FU	*G. boninense*
3142	*G. boninense*	KJ143906	SH1723050.08FU	*G. boninense*
3143	*G. boninense*	KM015454	SH1723050.08FU	*G. boninense*
3144	*G. boninense*	KM220584	SH1723050.08FU	*G. boninense*
3145	*G. boninense*	KM271997	SH1723050.08FU	*G. boninense*
3146	*G. boninense*	KX092000	SH1723050.08FU	*G. boninense*
3147	*G. boninense*	KX499467	SH1723050.08FU	*G. boninense*
3148	*G. boninense*	MG200172		*G. boninense*
3149	*G. boninense*	MG200173	SH1723050.08FU	*G. boninense*
3150	*G. boninense*	MG650116	SH1723050.08FU	*G. boninense*
3151	*G. boninense*	MK713555	SH1723050.08FU	*G. boninense*
3152	*G. boninense*	MK713556	SH1723050.08FU	*G. boninense*
3153	*G. boninense*	MK713557	SH1723050.08FU	*G. boninense*
3154	*G. boninense*	MK713558	SH1723050.08FU	*G. boninense*
3155	*G. boninense*	MK713559	SH1723050.08FU	*G. boninense*
3156	*G. boninense*	MK713560	SH1723050.08FU	*G. boninense*
3157	*G. boninense*	MK713561	SH1723050.08FU	*G. boninense*
3158	*G. boninense*	MN148580	SH1723050.08FU	*G. boninense*

3159	*G. boninense*	MN490048	SH1723050.08FU	*G. boninense*
3160	*G. boninense*	MT487851		*G. boninense*
3161	*Ganoderma* sp.	AY220537	SH1723050.08FU	*G. boninense*
3162	*Ganoderma* sp.	AY220538	SH1723050.08FU	*G. boninense*
3163	*Ganoderma* sp.	AY220539	SH1723050.08FU	*G. boninense*
3164	*Ganoderma* sp.	AY220540		*G. boninense*
3165	*Ganoderma* sp.	AY220541	SH1723137.08FU	*G. boninense*
3166	*Ganoderma* sp.	AY220542	SH1723050.08FU	*G. boninense*
3167	*Ganoderma* sp.	AY220543	SH1723050.08FU	*G. boninense*
3168	*Ganoderma* sp.	EF016754	SH1723050.08FU	*G. boninense*
3169	*Ganoderma* sp.	EU239386	SH1723050.08FU	*G. boninense*
3170	*Ganoderma* sp.	JN234427	SH1723050.08FU	*G. boninense*
3171	*Ganoderma* sp.	JN234428	SH1723050.08FU	*G. boninense*
3172	*Ganoderma* sp.	JN234429	SH1723050.08FU	*G. boninense*
3173	*Ganoderma* sp.	JN400510	SH1723050.08FU	*G. boninense*
3174	*Ganoderma* sp.	JN400511	SH1723050.08FU	*G. boninense*
3175	*Ganoderma* sp.	JN400513	SH1723050.08FU	*G. boninense*
3176	*Ganoderma* sp.	JN400515	SH1723050.08FU	*G. boninense*
3177	*G. orbiforme*	JX997990	SH1723050.08FU	*G. boninense*
3178	*G. miniatocinctum*	KM220586	SH1723050.08FU	*G. boninense*
3179	*G. orbiforme*	KX421867	SH1723050.08FU	*G. boninense*
3180	*Ganoderma* sp.	KY352306	SH1723050.08FU	*G. boninense*
3181	*Ganoderma* sp.	KY471673	SH1723050.08FU	*G. boninense*
3182	*Ganoderma* sp.	KY471674	SH1723050.08FU	*G. boninense*
3183	*Ganoderma* sp.	KY471677	SH1723050.08FU	*G. boninense*
3184	*Ganoderma* sp.	KY471680	SH1723050.08FU	*G. boninense*
3185	*Ganoderma* sp.	KY471683	SH1723137.08FU	*G. boninense*
3186	*Ganoderma* sp.	KY471685	SH1723050.08FU	*G. boninense*
3187	*Ganoderma* sp.	KY471686	SH1723050.08FU	*G. boninense*
3188	*Ganoderma* sp.	KY471687	SH1723050.08FU	*G. boninense*
3189	*Ganoderma* sp.	KY471688	SH1723050.08FU	*G. boninense*
3190	*Ganoderma* sp.	MK605934	SH1723050.08FU	*G. boninense*
3191	*Ganoderma* sp.	MN396662		*G. boninense*
3192	*Ganoderma* sp.	MN398324	SH1723050.08FU	*G. boninense*
3193	*G. zonatum*	MN490052	SH1723050.08FU	*G. boninense*
3194	*G. miniatocinctum*	MN490055	SH1723050.08FU	*G. boninense*
3195	*G. boninense*	KM271998	SH2753886.08FU	*G. boninense*
3196	*G. boninense*	KR093028	SH1845795.08FU	*G. boninense*
3197	*G. boninense*	KR093029	SH2754513.08FU	*G. boninense*
3198	*G. boninense*	MN490049	SH2766091.08FU	*G. boninense*
3199	*G. boninense*	MN490050		*G. boninense*
3200	*G. boninense*	MN490051	SH2766090.08FU	*G. boninense*
3201	*G. zonatum*	MN490053	SH2766089.08FU	*G. boninense*
3202	*G. zonatum*	MN490054	SH1723050.08FU	*G. boninense*
3203	*G. miniatocinctum*	MN490056	SH2766088.08FU	*G. boninense*

3204	*Ganoderma* sp.	AY220544	SH1723238.08FU	*Ganoderma* sp. D3
3205	*Ganoderma* sp.	JN400509	SH1723098.08FU	*Ganoderma* sp. D3
3206	*Ganoderma* sp.	JN400512	SH1723050.08FU	*Ganoderma* sp. D3
3207	*Ganoderma* sp.	JN400514	SH1723050.08FU	*Ganoderma* sp. D3
3208	*Ganoderma* sp.	KY471675		*Ganoderma* sp. D3
3209	*Ganoderma* sp.	KY471676		*Ganoderma* sp. D3
3210	*Ganoderma* sp.	KY471678	SH1723098.08FU	*Ganoderma* sp. D3
3211	*Ganoderma* sp.	KY471679	SH1723050.08FU	*Ganoderma* sp. D3
3212	*Ganoderma* sp.	KY471681	SH1723098.08FU	*Ganoderma* sp. D3
3213	*Ganoderma* sp.	KY471682	SH1723050.08FU	*Ganoderma* sp. D3
3214	*Ganoderma* sp.	KY471684	SH1723098.08FU	*Ganoderma* sp. D3
3215	*Ganoderma* sp.	KY471689	SH1723098.08FU	*Ganoderma* sp. D3
3216	*G. williamsianum*	KU219994	SH1723054.08FU	*G. williamsianum*
3217	*G. williamsianum*	KU219995	SH1723054.08FU	*G. williamsianum*
3218	*G. williamsianum*	MG279168	SH1723054.08FU	*G. williamsianum*
3219	*G. williamsianum*	MG279169	SH1723054.08FU	*G. williamsianum*
3220	*G. williamsianum*	MG279183	SH1723054.08FU	*G. williamsianum*
3221	*G. williamsianum*	MH071336	SH1723054.08FU	*G. williamsianum*
3222	*G. williamsianum*	MN398323	SH1723054.08FU	*G. williamsianum*
3223	*G. australe cplx*	AF255146	SH1723054.08FU	*G. williamsianum*
3224	*G. australe cplx*	AF255147	SH1723054.08FU	*G. williamsianum*
3225	*G.* cf. *australe*	JN596326	SH1723153.08FU	*G. williamsianum*
3226	*G.* cf. *australe*	JN596327	SH1723153.08FU	*G. williamsianum*
3227	*G. australe*	KJ654366	SH1723054.08FU	*G. williamsianum*
3228	*G. australe*	KJ654367	SH1723054.08FU	*G. williamsianum*
3229	*G. australe*	KJ654368	SH1723054.08FU	*G. williamsianum*
3230	*G. australe*	KJ654369	SH1723054.08FU	*G. williamsianum*
3231	*G. australe*	KJ654370	SH1723054.08FU	*G. williamsianum*
3232	*G. australe*	KJ654371	SH1723054.08FU	*G. williamsianum*
3233	*G. australe*	KJ654397	SH1723054.08FU	*G. williamsianum*
3234	*G. australe*	KJ654398	SH1723054.08FU	*G. williamsianum*
3235	*G. australe*	KJ654399	SH1723054.08FU	*G. williamsianum*
3236	*G. australe*	KJ654400		*G. williamsianum*
3237	*G. australe*	KJ654401	SH1723054.08FU	*G. williamsianum*
3238	*G. australe*	KJ654402	SH1723054.08FU	*G. williamsianum*
3239	*G. australe*	KJ654403	SH1723054.08FU	*G. williamsianum*
3240	*G. australe*	KJ654441	SH1723054.08FU	*G. williamsianum*
3241	*G. australe*	KJ654442	SH1723054.08FU	*G. williamsianum*
3242	*G. australe*	KJ654443	SH1723054.08FU	*G. williamsianum*
3243	*G. australe*	KJ654444	SH1723054.08FU	*G. williamsianum*
3244	*G. australe*	KJ654445	SH1723054.08FU	*G. williamsianum*
3245	*Ganoderma* sp.	KJ862063	SH1723054.08FU	*G. williamsianum*
3246	*G. australe*	LC084661	SH1723054.08FU	*G. williamsianum*
3247	*G. australe*	LC084667	SH1723054.08FU	*G. williamsianum*
3248	*G. australe*	LC084680	SH1723054.08FU	*G. williamsianum*

3249	*G. australe*	LC084691	SH1723054.08FU	*G. williamsianum*
3250	*G. australe*	LC084696	SH1723054.08FU	*G. williamsianum*
3251	*G. australe*	LC084706	SH1723054.08FU	*G. williamsianum*
3252	*G. australe*	LC084721	SH1723054.08FU	*G. williamsianum*
3253	*G. australe*	LC084726	SH1723054.08FU	*G. williamsianum*
3254	*G. australe*	LC084727	SH1723054.08FU	*G. williamsianum*
3255	*G. australe*	LC084742	SH1723054.08FU	*G. williamsianum*
3256	uncultured fungus	MF942545	SH1723054.08FU	*G. williamsianum*
3257	*G. australe*	MK345429	SH1723054.08FU	*G. williamsianum*
3258	*G. applanatum* cplx	AF255130	SH1723047.08FU	*Ganoderma* sp. E1
3259	*G. applanatum* cplx	AF255134	SH1723047.08FU	*Ganoderma* sp. E1
3260	*Ganoderma* sp.	AF255135	SH1723047.08FU	*Ganoderma* sp. E1
3261	*G. applanatum* cplx	AF255136	SH1723047.08FU	*Ganoderma* sp. E1
3262	*G. applanatum* cplx	AF255137	SH1723047.08FU	*Ganoderma* sp. E1
3263	*G. applanatum* cplx	AF255138	SH1723047.08FU	*Ganoderma* sp. E1
3264	*G. applanatum* cplx	AF255139	SH1723047.08FU	*Ganoderma* sp. E1
3265	*G. applanatum* cplx	AF255140	SH1723047.08FU	*Ganoderma* sp. E1
3266	*G. applanatum* cplx	AF255141	SH1723047.08FU	*Ganoderma* sp. E1
3267	*G. tornatum*	JQ514107	SH1723047.08FU	*Ganoderma* sp. E1
3268	*G. tornatum*	JQ514108	SH1723047.08FU	*Ganoderma* sp. E1
3269	*G. tornatum*	JQ514109	SH1723047.08FU	*Ganoderma* sp. E1
3270	*G. tornatum*	JQ514110		*Ganoderma* sp. E1
3271	*Ganoderma* sp.	JX082353	SH1723047.08FU	*Ganoderma* sp. E1
3272	*Ganoderma* sp.	JX082354	SH1723047.08FU	*Ganoderma* sp. E1
3273	*G. lobatum*	KF605669		*Ganoderma* sp. E1
3274	*G. lobatum*	KF605672	SH1723047.08FU	*Ganoderma* sp. E1
3275	*G. gibbosum*	KU569529	SH1723047.08FU	*Ganoderma* sp. E1
3276	*G. tornatum*	KU948516	SH1723047.08FU	*Ganoderma* sp. E1
3277	*G. tornatum*	KU948517	SH1723047.08FU	*Ganoderma* sp. E1
3278	*Ganoderma* sp.	MH267949	SH1723047.08FU	*Ganoderma* sp. E1
3279	*G. lobatum*	MT232631		*Ganoderma* sp. E1
3280	*G. tornatum*	MT232633		*Ganoderma* sp. E1
3281	*G. tornatum*	AF169985/6 & AH008101		*Ganoderma* sp. E2
3282	*G. applanatum* cplx	AF255131/2		*Ganoderma* sp. E2
3283	*G. applanatum* cplx	AF255133		*Ganoderma* sp. E2
3284	*Ganoderma* sp.	HM192933	SH1723047.08FU	*Ganoderma* sp. E2
3285	*G. tornatum*	JQ514100		*Ganoderma* sp. E2
3286	*G. tornatum*	JQ514101	SH1723047.08FU	*Ganoderma* sp. E2
3287	*G. tornatum*	JQ514102	SH1723047.08FU	*Ganoderma* sp. E2
3288	*G. tornatum*	JQ514103	SH1723047.08FU	*Ganoderma* sp. E2
3289	*G. tornatum*	JQ514104	SH1723047.08FU	*Ganoderma* sp. E2
3290	*G. australe*	JQ514105	SH1723047.08FU	*Ganoderma* sp. E2
3291	*G. tornatum*	JQ514106	SH1723047.08FU	*Ganoderma* sp. E2
3292	*G. lobatum*	KF605670	SH1723047.08FU	*Ganoderma* sp. E2
3293	*G. lobatum*	KF605671	SH1723047.08FU	*Ganoderma* sp. E2

3294	*G. lobatum*	KF605673		*Ganoderma* sp. E2
3295	*G. lobatum*	KF605674	SH1723047.08FU	*Ganoderma* sp. E2
3296	*G. lobatum*	KF605675	SH1723047.08FU	*Ganoderma* sp. E2
3297	*G. lobatum*	KF605676	SH1723047.08FU	*Ganoderma* sp. E2
3298	*G. lobatum*	KF605677	SH1723047.08FU	*Ganoderma* sp. E2
3299	*G. australe*	KU315203	SH1723047.08FU	*Ganoderma* sp. E2
3300	*G. gibbosum*	KU569535	SH1723047.08FU	*Ganoderma* sp. E2
3301	*G. gibbosum*	KU569537	SH1723047.08FU	*Ganoderma* sp. E2
3302	*G. gibbosum*	KU569543	SH1723047.08FU	*Ganoderma* sp. E2
3303	*G. gibbosum*	KU569544	SH1723047.08FU	*Ganoderma* sp. E2
3304	*G. gibbosum*	KU569547	SH1723047.08FU	*Ganoderma* sp. E2
3305	*G. gibbosum*	KU569548	SH1723047.08FU	*Ganoderma* sp. E2
3306	*G. gibbosum*	KU569550	SH1723047.08FU	*Ganoderma* sp. E2
3307	*G. gibbosum*	KU569551	SH1723047.08FU	*Ganoderma* sp. E2
3308	*G. gibbosum*	KU569552	SH1723047.08FU	*Ganoderma* sp. E2
3309	*G. gibbosum*	KU569554	SH1723047.08FU	*Ganoderma* sp. E2
3310	*G. gibbosum*	KU569556	SH1723047.08FU	*Ganoderma* sp. E2
3311	*G. gibbosum*	KU569557	SH1723047.08FU	*Ganoderma* sp. E2
3312	*Ganoderma* sp.	LT726721		*Ganoderma* sp. E2
3313	*Ganoderma* sp.	LT726722	SH1723047.08FU	*Ganoderma* sp. E2
3314	*Ganoderma* sp.	LT726723	SH1723047.08FU	*Ganoderma* sp. E2
3315	*Ganoderma* sp.	LT726724		*Ganoderma* sp. E2
3316	*Ganoderma* sp.	LT726727	SH1723047.08FU	*Ganoderma* sp. E2
3317	*G. tornatum*	MF347411	SH1723047.08FU	*Ganoderma* sp. E2
3318	*G. australe*	AY968692/AY993913		*G.* aff. *gibbosum*
3319	*G. australe*	AY993920/1		*G.* aff. *gibbosum*
3320	*G. gibbosum*	JN655531		*G.* aff. *gibbosum*
3321	*Ganoderma* sp.	KM229634		*G.* aff. *gibbosum*
3322	*Ganoderma* sp.	KM229635		*G.* aff. *gibbosum*
3323	*Ganoderma* sp.	KM229636		*G.* aff. *gibbosum*
3324	*Ganoderma* sp.	KM229637		*G.* aff. *gibbosum*
3325	*Ganoderma* sp.	KM229638		*G.* aff. *gibbosum*
3326	*Ganoderma* sp.	KM229639		*G.* aff. *gibbosum*
3327	*Ganoderma* sp.	KM229640		*G.* aff. *gibbosum*
3328	*Ganoderma* sp.	KM229641		*G.* aff. *gibbosum*
3329	*Ganoderma* sp.	KM229642		*G.* aff. *gibbosum*
3330	*Ganoderma* sp.	KM229643		*G.* aff. *gibbosum*
3331	*Ganoderma* sp.	KM229644		*G.* aff. *gibbosum*
3332	*Ganoderma* sp.	KM229645		*G.* aff. *gibbosum*
3333	*Ganoderma* sp.	KM229646		*G.* aff. *gibbosum*
3334	*Ganoderma* sp.	KM229647		*G.* aff. *gibbosum*
3335	*Ganoderma* sp.	KM229648		*G.* aff. *gibbosum*
3336	*Ganoderma* sp.	KM229649		*G.* aff. *gibbosum*
3337	*Ganoderma* sp.	KM229650		*G.* aff. *gibbosum*
3338	*Ganoderma* sp.	KM229651		*G.* aff. *gibbosum*

3339	*Ganoderma* sp.	KM229652		*G.* aff. *gibbosum*
3340	*Ganoderma* sp.	KM229653		*G.* aff. *gibbosum*
3341	*Ganoderma* sp.	KM229654		*G.* aff. *gibbosum*
3342	*Ganoderma* sp.	KM229655		*G.* aff. *gibbosum*
3343	*Ganoderma* sp.	KM229656		*G.* aff. *gibbosum*
3344	*Ganoderma* sp.	KM229657		*G.* aff. *gibbosum*
3345	*Ganoderma* sp.	KM229658		*G.* aff. *gibbosum*
3346	*Ganoderma* sp.	KM229659		*G.* aff. *gibbosum*
3347	*Ganoderma* sp.	KM229662		*G.* aff. *gibbosum*
3348	*Ganoderma* sp.	KM229663		*G.* aff. *gibbosum*
3349	*Ganoderma* sp.	KM229664		*G.* aff. *gibbosum*
3350	*Ganoderma* sp.	KM229665		*G.* aff. *gibbosum*
3351	*Ganoderma* sp.	KM229666		*G.* aff. *gibbosum*
3352	*Ganoderma* sp.	KM229667		*G.* aff. *gibbosum*
3353	*Ganoderma* sp.	KM229668		*G.* aff. *gibbosum*
3354	*Ganoderma* sp.	KM229669		*G.* aff. *gibbosum*
3355	*Ganoderma* sp.	KM229670		*G.* aff. *gibbosum*
3356	*Ganoderma* sp.	KM229671		*G.* aff. *gibbosum*
3357	*Ganoderma* sp.	KM229672		*G.* aff. *gibbosum*
3358	*Ganoderma* sp.	KM229673		*G.* aff. *gibbosum*
3359	*Ganoderma* sp.	KM229674		*G.* aff. *gibbosum*
3360	*Ganoderma* sp.	KM229675		*G.* aff. *gibbosum*
3361	*Ganoderma* sp.	KM229676		*G.* aff. *gibbosum*
3362	*Ganoderma* sp.	KM229677		*G.* aff. *gibbosum*
3363	*Ganoderma* sp.	KR154930		*G.* aff. *gibbosum*
3364	*G. australe*	KJ792748		*G.* aff. *gibbosum*
3365	*Ganoderma* sp.	KM229660	SH1730465.08FU	*G.* aff. *gibbosum*
3366	*Ganoderma* sp.	KM229661	SH1723272.08FU	*G.* aff. *gibbosum*
3367	*Ganoderma* sp.	MK681873	SH2759056.08FU	*G.* aff. *gibbosum*
3368	*G. ryvardenii*	MK681874	SH2759055.08FU	*G.* aff. *gibbosum*
3369	*G. eickeri*	MH571689		*G. eickeri*
3370	*G. eickeri*	MH571690/NR_165524		*G. eickeri*
3371	*Ganoderma* sp.	MG020264		*G. eickeri*
3372	*Ganoderma* sp.	MG020265		*G. eickeri*
3373	*G. gibbosum*	AB733121		*G. gibbosum*
3374	*G. gibbosum*	AY593854		*G. gibbosum*
3375	*G. gibbosum*	AY593855		*G. gibbosum*
3376	*G. gibbosum*	AY593856		*G. gibbosum*
3377	*G. gibbosum*	AY593857		*G. gibbosum*
3378	*G. gibbosum*	EU273513		*G. gibbosum*
3379	*G. gibbosum*	EU273514		*G. gibbosum*
3380	*G. gibbosum*	EU273555		*G. gibbosum*
3381	*G. gibbosum*	EU273557		*G. gibbosum*
3382	*G. gibbosum*	EU326218		*G. gibbosum*
3383	*G. gibbosum*	EU326219		*G. gibbosum*

3384	*G. gibbosum*	EU918695	*G. gibbosum*
3385	*G. gibbosum*	FJ582638	*G. gibbosum*
3386	*G. gibbosum*	KJ195663	*G. gibbosum*
3387	*G. gibbosum*	KR673513	*G. gibbosum*
3388	*G. gibbosum*	KX879638	*G. gibbosum*
3389	*G. gibbosum*	KY364259	*G. gibbosum*
3390	*G. gibbosum*	KY364260	*G. gibbosum*
3391	*G. gibbosum*	KY364261	*G. gibbosum*
3392	*G. gibbosum*	KY364262	*G. gibbosum*
3393	*G. gibbosum*	KY364263	*G. gibbosum*
3394	*G. gibbosum*	KY364264	*G. gibbosum*
3395	*G. gibbosum*	KY364265	*G. gibbosum*
3396	*G. gibbosum*	KY364266	*G. gibbosum*
3397	*G. gibbosum*	KY364267	*G. gibbosum*
3398	*G. gibbosum*	KY364268	*G. gibbosum*
3399	*G. gibbosum*	KY364269	*G. gibbosum*
3400	*G. gibbosum*	KY364270	*G. gibbosum*
3401	*G. gibbosum*	KY364271	*G. gibbosum*
3402	*G. gibbosum*	KY364272	*G. gibbosum*
3403	*G. gibbosum*	MH035681	*G. gibbosum*
3404	*G. gibbosum*	MH035682	*G. gibbosum*
3405	*G. gibbosum*	MH035683	*G. gibbosum*
3406	*G. gibbosum*	MH035684	*G. gibbosum*
3407	*G. gibbosum*	MH035685	*G. gibbosum*
3408	*G. gibbosum*	MH035687	*G. gibbosum*
3409	*G. gibbosum*	MH035688	*G. gibbosum*
3410	*G. gibbosum*	MH106880	*G. gibbosum*
3411	*G. gibbosum*	MK280717	*G. gibbosum*
3412	*G. gibbosum*	MK345432	*G. gibbosum*
3413	*G. gibbosum*	MK345433	*G. gibbosum*
3414	*G. gibbosum*	MK345434	*G. gibbosum*
3415	*G. gibbosum*	MK345436	*G. gibbosum*
3416	*G. gibbosum*	MN396310	*G. gibbosum*
3417	*G. gibbosum*	MN396311	*G. gibbosum*
3418	*G. gibbosum*	MN396312	*G. gibbosum*
3419	*G. gibbosum*	MN396313	*G. gibbosum*
3420	*G. gibbosum*	MN396314	*G. gibbosum*
3421	*G. gibbosum*	MN396315	*G. gibbosum*
3422	*G. gibbosum*	MN398341	*G. gibbosum*
3423	*G. gibbosum*	MN398342	*G. gibbosum*
3424	*G. gibbosum*	MN398343	*G. gibbosum*
3425	*G. gibbosum*	MN398344	*G. gibbosum*
3426	*G. gibbosum*	MN523326	*G. gibbosum*
3427	*G. gibbosum*	MN622779	*G. gibbosum*
3428	*G. gibbosum*	MN622780	*G. gibbosum*

3429	*G. gibbosum*	MN622799		*G. gibbosum*
3430	*G. gibbosum*	X78741/X78762		*G. gibbosum*
3431	*G. gibbosum*	MK370672		*G. gibbosum*
3432	*G. australe* cplx	AF255105		*G. gibbosum*
3433	*G. australe*	AF255106/7		*G. gibbosum*
3434	*G. australe* cplx	AF255108/9		*G. gibbosum*
3435	*G. australe* IG 1	AF255110/1		*G. gibbosum*
3436	*G. australe* IG 1	AF255112/3		*G. gibbosum*
3437	*G. applanatum*	AF255114		*G. gibbosum*
3438	*G. lucidum*	AF506372		*G. gibbosum*
3439	*G. applanatum*	DQ424996		*G. gibbosum*
3440	*G. applanatum*	DQ425009		*G. gibbosum*
3441	*G. fulvellum*	FJ478088		*G. gibbosum*
3442	*G. applanatum*	GU213472		*G. gibbosum*
3443	*G. applanatum*	GU213473		*G. gibbosum*
3444	*G. australe*	GU213474		*G. gibbosum*
3445	*Ganoderma* sp.	HQ891299		*G. gibbosum*
3446	*G. applanatum*	JN008873		*G. gibbosum*
3447	*G. australe*	JX195196		*G. gibbosum*
3448	*G. australe*	JX195197		*G. gibbosum*
3449	*G. australe*	JX195199		*G. gibbosum*
3450	*G. australe*	JX195200		*G. gibbosum*
3451	*G. applanatum*	KF494999		*G. gibbosum*
3452	uncultured *Ganoderma*	KF574247		*G. gibbosum*
3453	*G. applanatum*	KM249934		*G. gibbosum*
3454	*G. applanatum*	KM249935		*G. gibbosum*
3455	*G. applanatum*	KM249936		*G. gibbosum*
3456	*G. applanatum*	KM609399	SH1723291.08FU	*G. gibbosum*
3457	*Fuscoporia viticola*	MG231526		*G. gibbosum*
3458	*G. lingzhi*	MG279179		*G. gibbosum*
3459	*G. applanatum*	MG657360		*G. gibbosum*
3460	*G. applanatum*	MG657361		*G. gibbosum*
3461	*G. applanatum*	MG657362		*G. gibbosum*
3462	*G. applanatum*	MG657363		*G. gibbosum*
3463	*G. applanatum*	MG657364		*G. gibbosum*
3464	*G. applanatum*	MG657365		*G. gibbosum*
3465	*G. applanatum*	MG657366		*G. gibbosum*
3466	*G. australe*	MG719604		*G. gibbosum*
3467	*G. applanatum*	MH114667		*G. gibbosum*
3468	*G. applanatum*	MH114668		*G. gibbosum*
3469	*Ganoderma* sp.	MH507186		*G. gibbosum*
3470	Agaricales sp.	MK079623		*G. gibbosum*
3471	*G. applanatum*	MK268927		*G. gibbosum*
3472	*G. applanatum*	MK268928		*G. gibbosum*
3473	*G. lingzhi*	MK343538		*G. gibbosum*

3474	*Ganoderma* sp.	MK605939		*G. gibbosum*
3475	*G. applanatum*	MK809458		*G. gibbosum*
3476	*G. applanatum*	MN238820		*G. gibbosum*
3477	*G. applanatum*	MN238821		*G. gibbosum*
3478	*G. applanatum*	MN294816		*G. gibbosum*
3479	*G. applanatum*	MN294853		*G. gibbosum*
3480	*G. australe*	X78750/X78771		*G. gibbosum*
3481	*G. gibbosum*	KY203856		*G. gibbosum*
3482	*G. gibbosum*	KY950512		*G. gibbosum*
3483	*G. australe*	MH101642		*G. gibbosum*
3484	*G. australe*	MH101643		*G. gibbosum*
3485	*G. applanatum*	MN258633		*G. gibbosum*
3486	*G. ellipsoideum*	MH106867/NR_160617		*G. ellipsoideum*
3487	*G. ellipsoideum*	MH106868		*G. ellipsoideum*
3488	*G. ellipsoideum*	MH106886		*G. ellipsoideum*
3489	*G. ellipsoideum*	MK333264		*G. ellipsoideum*
3490	*G. ellipsoideum*	MN398339		*G. ellipsoideum*
3491	*G. applanatum* cplx	AF255115		*G. ellipsoideum*
3492	*G. applanatum* cplx	AF255116		*G. ellipsoideum*
3493	*G. applanatum* cplx	AF255117		*G. ellipsoideum*
3494	*G. australe* cplx	AF255118		*G. ellipsoideum*
3495	*G. australe* cplx	AF255119		*G. ellipsoideum*
3496	*G. australe* cplx	AF255120		*G. ellipsoideum*
3497	*G. australe* cplx	AF255121		*G. ellipsoideum*
3498	*G. australe* cplx	AF255122		*G. ellipsoideum*
3499	*G. australe* cplx	AF255123/4		*G. ellipsoideum*
3500	*G. australe* cplx	AF255125		*G. ellipsoideum*
3501	*G. australe* cplx	AF255126/7		*G. ellipsoideum*
3502	*G. australe* cplx	AF255128		*G. ellipsoideum*
3503	*G. australe* cplx	AF255129		*G. ellipsoideum*
3504	*G.* aff. *steyaertanum*	EU239387		*G. ellipsoideum*
3505	*G. gibbosum*	FJ392286		*G. ellipsoideum*
3506	*G. gibbosum*	JN596331	SH1723251.08FU	*G. ellipsoideum*
3507	*G. australe*	JX195204		*G. ellipsoideum*
3508	*G. australe*	JX195205		*G. ellipsoideum*
3509	*G. gibbosum*	KJ654372		*G. ellipsoideum*
3510	*G. gibbosum*	KJ654373		*G. ellipsoideum*
3511	*G. gibbosum*	KJ654404		*G. ellipsoideum*
3512	*G. gibbosum*	KJ654405		*G. ellipsoideum*
3513	*G. gibbosum*	KJ654446		*G. ellipsoideum*
3514	*G. tornatum*	KJ767488		*G. ellipsoideum*
3515	*Ganoderma* sp.	KM229678		*G. ellipsoideum*
3516	*Ganoderma* sp.	KP012934		*G. ellipsoideum*
3517	*Ganoderma* sp.	KP861881		*G. ellipsoideum*
3518	*Tomophagus* sp.	KR632638		*G. ellipsoideum*

3519	*G. applanatum*	KR867655		*G. ellipsoideum*
3520	*G. gibbosum*	KT318591		*G. ellipsoideum*
3521	*Ganoderma* sp.	KT965498	SH1723186.08FU	*G. ellipsoideum*
3522	*Ganoderma* sp.	KU194305		*G. ellipsoideum*
3523	*G. gibbosum*	KU194307	SH1723186.08FU	*G. ellipsoideum*
3524	*G. gibbosum*	KU194311		*G. ellipsoideum*
3525	*G. applanatum*	KU194312	SH1723186.08FU	*G. ellipsoideum*
3526	*G. gibbosum*	KU194326		*G. ellipsoideum*
3527	*G. gibbosum*	KU194347		*G. ellipsoideum*
3528	*G. australe*	LC084663		*G. ellipsoideum*
3529	*G. australe*	LC084717		*G. ellipsoideum*
3530	*G. australe*	LC084749		*G. ellipsoideum*
3531	*G. applanatum*	MF072395		*G. ellipsoideum*
3532	*G. applanatum*	MG448603		*G. ellipsoideum*
3533	*G. applanatum*	MG719303		*G. ellipsoideum*
3534	*G. australe*	MH101644		*G. ellipsoideum*
3535	*G. gibbosum*	MH114669		*G. ellipsoideum*
3536	*G. gibbosum*	MH114670		*G. ellipsoideum*
3537	*Ganoderma* sp.	MK131238		*G. ellipsoideum*
3538	*Ganoderma* sp.	MK131240		*G. ellipsoideum*
3539	*Ganoderma* sp.	MK131242		*G. ellipsoideum*
3540	*G. adspersum*	MK345425		*G. ellipsoideum*
3541	*G. gibbosum*	MK345435		*G. ellipsoideum*
3542	*G. gibbosum*	MK404342		*G. ellipsoideum*
3543	*G. adspersum*	MN396652		*G. ellipsoideum*
3544	*G. adspersum*	MN396653		*G. ellipsoideum*
3545	*G. adspersum*	MN396654		*G. ellipsoideum*
3546	*G. adspersum*	MN396655		*G. ellipsoideum*
3547	*G. gibbosum*	MN523324		*G. ellipsoideum*
3548	*G. gibbosum*	MN523325		*G. ellipsoideum*
3549	*G. australe*	MN689631		*G. ellipsoideum*
3550	*G. australe*	MN747808		*G. ellipsoideum*
3551	*G. gibbosum*	MT252597		*G. ellipsoideum*
3552	uncultured soil fungus	UDB0755339	SH1723040.08FU	*G. ellipsoideum*
3553	uncultured soil fungus	UDB0763169	SH1723040.08FU	*G. ellipsoideum*
3554	uncultured soil fungus	UDB0763546	SH1723040.08FU	*G. ellipsoideum*
3555	uncultured soil fungus	UDB0769802	SH1723040.08FU	*G. ellipsoideum*
3556	uncultured soil fungus	UDB0777839	SH1723040.08FU	*G. ellipsoideum*
3557	*G. australe*	MN696209		*G. ellipsoideum*
3558	*Ganoderma* sp.	JN596333		*G. ellipsoideum*
3559	*G. tornatum*	KM220585	SH1677215.08FU	*G. ellipsoideum*
3560	*Ganoderma* sp.	KX421866	SH1730466.08FU	*G. ellipsoideum*
3561	*G. australe*	MF680426	SH1723186.08FU	*G. ellipsoideum*
3562	*G. gibbosum*	MG231524	SH1730463.08FU	*G. ellipsoideum*
3563	*G. gibbosum*	MK268932	SH2768137.08FU	*G. ellipsoideum*

3564	*G. gibbosum*	MK404343	SH2767220.08FU	*G. ellipsoideum*
3565	*G. tornatum*	MN490057	SH2766087.08FU	*G. ellipsoideum*
3566	*G. australe*	EU239383	SH1723178.08FU	*Ganoderma* sp. E3
3567	*G. australe*	EU239390	SH1723176.08FU	*Ganoderma* sp. E3
3568	*G. australe*	FR821768	SH1723270.08FU	*Ganoderma* sp. E3
3569	*G. australe*	KJ654549	SH1723116.08FU	*Ganoderma* sp. E3
3570	*G. australe*	KJ654550	SH1723116.08FU	*Ganoderma* sp. E3
3571	*G. australe*	KJ654551	SH1723116.08FU	*Ganoderma* sp. E3
3572	uncultured soil fungus	UDB0755590	SH1723270.08FU	*Ganoderma* sp. E3
3573	*G. australe*	AF255142		*Ganoderma* sp. E4
3574	*G. australe*	AF255143	SH1677218.08FU	*Ganoderma* sp. E4
3575	*G. australe*	AF255144	SH1677211.08FU	*Ganoderma* sp. E4
3576	*G. australe*	AF255145	SH1677211.08FU	*Ganoderma* sp. E4
3577	*G. australe*	EU239389	SH1677213.08FU	*Ganoderma* sp. E4
3578	*G. australe*	KJ654379		*Ganoderma* sp. E4
3579	*G. australe*	KJ654544	SH1677211.08FU	*Ganoderma* sp. E4
3580	*G. australe*	KJ654545	SH1677211.08FU	*Ganoderma* sp. E4
3581	*G. australe*	KJ654546	SH1677211.08FU	*Ganoderma* sp. E4
3582	*G. australe*	KJ654547	SH1677211.08FU	*Ganoderma* sp. E4
3583	*G. australe*	KJ654548	SH1677214.08FU	*Ganoderma* sp. E4
3584	*G. australe*	KR093031	SH1677212.08FU	*Ganoderma* sp. E4
3585	*G. tornatum*	MN490058	SH1677211.08FU	*Ganoderma* sp. E4
3586	*G. knysnamense*	MH571681	SH2766456.08FU	*G. knysnamense*
3587	*G. knysnamense*	MH571682	SH2766456.08FU	*G. knysnamense*
3588	*G. knysnamense*	MH571683	SH2766456.08FU	*G. knysnamense*
3589	*G. knysnamense*	MH571684	SH2766456.08FU	*G. knysnamense*
3590	*G. mutabile*	JN383977	SH1723136.08FU	*G. mutabile*
3591	*G. mutabile*	MG231527	SH1723136.08FU	*G. mutabile*
3592	*G. cupreolaccatum*	MH856316	SH2759768.08FU	*G. cupreolaccatum*
3593	*G. pfeifferi*	MG279164	SH1723073.08FU	*G. pfeifferi*
3594	*G. pfeifferi*	MG279165	SH1723073.08FU	*G. pfeifferi*
3595	*G. pfeifferi*	MK415258		*G. pfeifferi*
3596	*G. pfeifferi*	MK415259		*G. pfeifferi*
3597	*G. pfeifferi*	MK415263		*G. pfeifferi*
3598	*G. pfeifferi*	MK415290		*G. pfeifferi*
3599	*G. pfeifferi*	MK415295		*G. pfeifferi*
3600	*G. pfeifferi*	MK415296		*G. pfeifferi*
3601	*G. pfeifferi*	MK415310		*G. pfeifferi*
3602	*G. pfeifferi*	AM906059	SH1723073.08FU	*G. pfeifferi*
3603	*G. pfeifferi*	AY884181	SH1723073.08FU	*G. pfeifferi*
3604	*G. pfeifferi*	AY884185	SH1723073.08FU	*G. pfeifferi*
3605	*G. pfeifferi*	JN008874	SH1723073.08FU	*G. pfeifferi*
3606	*G. pfeifferi*	JN222420	SH1723073.08FU	*G. pfeifferi*
3607	*G. pfeifferi*	KF605659	SH1723073.08FU	*G. pfeifferi*
3608	*G. pfeifferi*	KF605660	SH1723073.08FU	*G. pfeifferi*

3609	*G. pfeifferi*	MG706232	SH1723073.08FU	*G. pfeifferi*
3610	*G. chocoense*	MH890527/NR_163763	SH2764230.08FU	*G. chocoense*
3611	*G. podocarpense*	MF796661	SH1723234.08FU	*G. podocarpense*
3612	uncultured soil fungus	UDB0759513	SH1723234.08FU	*G. podocarpense*
3613	*G. lobatum*	AF169989/90 & AH008103		*Ganoderma* sp. E5
3614	*G. lobatum*	AF169991/2 & AH008104		*Ganoderma* sp. E5
3615	*G. tornatum*	AF169993/4 & AH008105		*Ganoderma* sp. E5
3616	*Ganoderma* sp.	AF255184		*Ganoderma* sp. E5
3617	*Ganoderma* sp.	AF255185		*Ganoderma* sp. E5
3618	*Ganoderma* sp.	AF255186		*Ganoderma* sp. E5
3619	*Ganoderma* sp.	AF255187	SH1678465.08FU	*Ganoderma* sp. E5
3620	uncultured soil fungus	UDB0768776	SH1678465.08FU	*Ganoderma* sp. E5
3621	*G.* cf. *australe*	AF255188	SH1723070.08FU	Ganoderma sp. E6
3622	*G.* cf. *philippii*	AF255189	SH1723070.08FU	Ganoderma sp. E6
3623	*Ganoderma* sp.	AF255190	SH1723070.08FU	Ganoderma sp. E6
3624	*G. applanatum*	AF255191/2		Ganoderma sp. E6
3625	*G. australe* IG 2	AF255193/4		Ganoderma sp. E6
3626	*G. australe* IG 2	AF255195	SH1723070.08FU	Ganoderma sp. E6
3627	*G. australe*	AY993918/9		Ganoderma sp. E6
3628	*G. applanatum*	EU232190	SH1723070.08FU	Ganoderma sp. E6
3629	*G. australe*	JN643731	SH1723070.08FU	Ganoderma sp. E6
3630	*G. australe*	JX195198	SH1723070.08FU	Ganoderma sp. E6
3631	*G. australe*	KT318590	SH1723070.08FU	Ganoderma sp. E6
3632	*Ganoderma* sp.	KT965497	SH1723070.08FU	Ganoderma sp. E6
3633	*Ganoderma* sp.	KU219992	SH1723070.08FU	Ganoderma sp. E6
3634	*Ganoderma* sp.	KU219993	SH1723070.08FU	Ganoderma sp. E6
3635	*G. australe*	LT716076	SH1723070.08FU	Ganoderma sp. E6
3636	*G. australe*	MH106871	SH1723070.08FU	Ganoderma sp. E6
3637	*G. australe*	MH106872	SH1723070.08FU	Ganoderma sp. E6
3638	*G. australe*	MK182313	SH1723070.08FU	Ganoderma sp. E6
3639	*G. australe*	MK345428	SH1723070.08FU	Ganoderma sp. E6
3640	*Ganoderma* sp.	MK605938	SH1723070.08FU	Ganoderma sp. E6
3641	*Ganoderma* sp.	MK605943	SH1723070.08FU	Ganoderma sp. E6
3642	*Ganoderma* sp.	MK605945	SH1723070.08FU	Ganoderma sp. E6
3643	*Ganoderma* sp.	MK774700	SH1723070.08FU	Ganoderma sp. E6
3644	*G. australe*	MK968731	SH1723070.08FU	Ganoderma sp. E6
3645	*G. australe*	MN396656		Ganoderma sp. E6
3646	*G. australe*	MN396657		Ganoderma sp. E6
3647	*G. australe*	MN396658		Ganoderma sp. E6
3648	*G. australe*	MN396659	SH1723070.08FU	Ganoderma sp. E6
3649	*G. australe*	MN396660		Ganoderma sp. E6
3650	*G. australe*	MN396661		Ganoderma sp. E6
3651	*G. australe*	MN398335		Ganoderma sp. E6
3652	*G. australe*	MN398336	SH1723070.08FU	Ganoderma sp. E6
3653	uncultured soil fungus	UDB0755383	SH2770217.08FU	Ganoderma sp. E6

3654	uncultured *Ganoderma*	KX831667		Ganoderma sp. E6
3655	*G. australe*	MK268929	SH1723070.08FU	Ganoderma sp. E6
3656	*G. australe cplx*	AF255183	SH1723220.08FU	Ganoderma sp. aff. E6
3657	*G. australe*	AJ627590/1		*G. australe*
3658	*G. australe*	AJ627592/3		*G. australe*
3659	*G. australe*	AY884180	SH1723052.08FU	*G. australe*
3660	*G. australe*	KF605661		*G. australe*
3661	*G. australe*	KF605662	SH1723052.08FU	*G. australe*
3662	*G. australe*	KF605663	SH1723052.08FU	*G. australe*
3663	*G. australe*	KF605664		*G. australe*
3664	*G. australe*	KF605665	SH1723052.08FU	*G. australe*
3665	*G. australe*	KU569531	SH1723052.08FU	*G. australe*
3666	*G. australe*	KU569532	SH1723052.08FU	*G. australe*
3667	*G. australe*	KU569533	SH1723052.08FU	*G. australe*
3668	*G. australe*	KU569534	SH1723052.08FU	*G. australe*
3669	*G. australe*	KU569536	SH1723052.08FU	*G. australe*
3670	*G. australe*	KU569538	SH1723052.08FU	*G. australe*
3671	*G. australe*	KU569539	SH1723052.08FU	*G. australe*
3672	*G. australe*	KU569540	SH1723052.08FU	*G. australe*
3673	*G. australe*	KU569541	SH1723052.08FU	*G. australe*
3674	*G. australe*	KU569545	SH1723052.08FU	*G. australe*
3675	*G. australe*	MF436675	SH1723052.08FU	*G. australe*
3676	*G. australe*	MF436676	SH1723052.08FU	*G. australe*
3677	*G. australe*	MH571685	SH1723052.08FU	*G. australe*
3678	*G. australe*	MH571686	SH1723052.08FU	*G. australe*
3679	*G. australe*	MH571687	SH1723052.08FU	*G. australe*
3680	*G. australe*	MH571688	SH1723052.08FU	*G. australe*
3681	*G. australe*	MK322289	SH1723052.08FU	*G. australe*
3682	*G. australe*	MK826012	SH1723052.08FU	*G. australe*
3683	*G. tornatum*	AF169975/6 & AH008096		*G. australe*
3684	*G. lipsiense*	AF169977/8 & AH008097		*G. australe*
3685	*G. tornatum*	AF169979/80 & AH008098		*G. australe*
3686	*Ganoderma* sp.	AF169981/2 & AH008099		*G. australe*
3687	*G. lobatum*	AF169983/4 & AH008100		*G. australe*
3688	*G. lobatum*	AF169987/8 & AH008102		*G. australe*
3689	*Ganoderma* sp.	AF169995/6 & AH008106		*G. australe*
3690	*G. applanatum* cplx	AF255148	SH1723052.08FU	*G. australe*
3691	*G. applanatum* cplx	AF255149	SH1723052.08FU	*G. australe*
3692	*G. lucidum*	AF255150	SH1723052.08FU	*G. australe*
3693	*G. australe* cplx	AF255151/2		*G. australe*
3694	*G. australe* cplx	AF255153/4		*G. australe*
3695	*G. australe* cplx	AF255155/6		*G. australe*
3696	*G. australe* cplx	AF255157		*G. australe*
3697	*G. australe* cplx	AF255158	SH1723052.08FU	*G. australe*
3698	*G. australe* cplx	AF255159	SH1723052.08FU	*G. australe*

3699	*G. australe* cplx	AF255160	SH1723239.08FU	*G. australe*
3700	*G. australe* cplx	AF255161/2		*G. australe*
3701	*Ganoderma* sp.	AF255163		*G. australe*
3702	*G. australe* cplx	AF255164/5		*G. australe*
3703	*G. australe* cplx	AF255166/7		*G. australe*
3704	*G. australe* cplx	AF255168/9		*G. australe*
3705	*G. australe* cplx	AF255170/1		*G. australe*
3706	*G. australe* cplx	AF255172/3		*G. australe*
3707	*G. australe* cplx	AF255174/5		*G. australe*
3708	*Ganoderma* sp.	AF255176		*G. australe*
3709	*Ganoderma* sp.	AF255177	SH1723052.08FU	*G. australe*
3710	*Ganoderma* sp.	AF255178/9		*G. australe*
3711	*Ganoderma* sp.	AF255180	SH1723052.08FU	*G. australe*
3712	*Ganoderma* sp.	AF255181/2		*G. australe*
3713	*G. adspersum*	AJ006685	SH1723052.08FU	*G. australe*
3714	*G. applanatum*	AJ608709	SH1723052.08FU	*G. australe*
3715	*Ganoderma* sp.	AM269768	SH1723052.08FU	*G. australe*
3716	*Ganoderma* sp.	AM269769	SH1723052.08FU	*G. australe*
3717	*Ganoderma* sp.	HM583824	SH1723052.08FU	*G. australe*
3718	*G. annulare*	JQ520160	SH1723052.08FU	*G. australe*
3719	*G. brownii*	MG279159		*G. australe*
3720	fungal sp.	MH242613	SH1723052.08FU	*G. australe*
3721	*Ganoderma* sp.	MK788298	SH1723052.08FU	*G. australe*
3722	*G. annulare*	MK826006	SH1723052.08FU	*G. australe*
3723	*G. brownii*	MK883702	SH1723052.08FU	*G. australe*
3724	*Ganoderma* sp.	MK934577		*G. australe*
3725	uncultured soil fungus	UDB0759941	SH1723052.08FU	*G. australe*
3726	uncultured soil fungus	UDB0762229	SH1723052.08FU	*G. australe*
3727	uncultured soil fungus	UDB0766747	SH2772714.08FU	*G. australe*
3728	uncultured soil fungus	UDB0767741	SH1723052.08FU	*G. australe*
3729	uncultured soil fungus	UDB0767760	SH1723052.08FU	*G. australe*
3730	uncultured soil fungus	UDB0767764	SH1723239.08FU	*G. australe*
3731	uncultured soil fungus	UDB0768220	SH1723052.08FU	*G. australe*
3732	uncultured soil fungus	UDB0768715	SH1723052.08FU	*G. australe*
3733	uncultured *Ganoderma*	KU712532		*G. australe*
3734	*G. australe*	MT076093		*G. australe*
3735	uncultured soil fungus	UDB0766717	SH1723052.08FU	*G. australe*
3736	uncultured soil fungus	UDB0766748	SH1723052.08FU	*G. australe*
3737	*G. applanatum* cplx	AF255099	SH1723077.08FU	*Ganoderma* sp. E7
3738	*G. applanatum* cplx	AF255100	SH1723077.08FU	*Ganoderma* sp. E7
3739	*Ganoderma* sp.	KF605653	SH1723077.08FU	*Ganoderma* sp. E7
3740	*Ganoderma* sp.	KF605654		*Ganoderma* sp. E7
3741	*Ganoderma* sp.	KF605655	SH1723077.08FU	*Ganoderma* sp. E7
3742	*Ganoderma* sp.	KF605656	SH1723077.08FU	*Ganoderma* sp. E7
3743	*Ganoderma* sp.	KF605657	SH1723077.08FU	*Ganoderma* sp. E7

3744	*Ganoderma* sp.	KF605658	SH1723077.08FU	*Ganoderma* sp. E7
3745	uncultured fungus	KF800469	SH1723077.08FU	*Ganoderma* sp. E7
3746	*Ganoderma* sp.	MN749647		*Ganoderma* sp. E7
3747	*Ganoderma* sp.	MN749648		*Ganoderma* sp. E7
3748	*G. lobatum*	MN906144		*Ganoderma* sp. E7
3749	*G. lobatum*	MN906145		*Ganoderma* sp. E7
3750	*Ganoderma* sp.	MN964001		*Ganoderma* sp. E7
3751	*Ganoderma* sp.	MN964002		*Ganoderma* sp. E7
3752	*G. lobatum*	X78740/X78761		*Ganoderma* sp. E7
3753	*G. applanatum* cplx	AF255098	SH1723305.08FU	*Ganoderma* sp. E7
3754	*G. australe* cplx	AF255102/3		G. aff. *adspersum*
3755	*G. australe* cplx	AF255104	SH1723117.08FU	G. aff. *adspersum*
3756	*G. applanatum*	JQ520161	SH1723117.08FU	G. aff. *adspersum*
3757	*G. adspersum*	KY364251		G. aff. *adspersum*
3758	*G. adspersum*	KY364252		G. aff. *adspersum*
3759	*G. adspersum*	KY364253		G. aff. *adspersum*
3760	*G. adspersum*	KY364254	SH1723117.08FU	G. aff. *adspersum*
3761	uncultured soil fungus	UDB0765962	SH1723117.08FU	G. aff. *adspersum*
3762	*G. applanatum*	MN080319		G. aff. *adspersum*
3763	*G. applanatum*	KR673486	SH1723208.08FU	G. aff. *adspersum*
3764	*G. applanatum*	KR673681	SH1723294.08FU	G. aff. *adspersum*
3765	*Ganoderma* sp.	AF255101		*G. adspersum*
3766	*Ganoderma* sp.	AF455510		*G. adspersum*
3767	*G. adspersum*	AM269767		*G. adspersum*
3768	*G. adspersum*	AM269770		*G. adspersum*
3769	*G. adspersum*	AM269771		*G. adspersum*
3770	*G. adspersum*	AM906054		*G. adspersum*
3771	*G. adspersum*	AM906055		*G. adspersum*
3772	*G. adspersum*	AM906056		*G. adspersum*
3773	*G. adspersum*	AM906057		*G. adspersum*
3774	basidiomycete sp.	AY605704		*G. adspersum*
3775	basidiomycete sp.	AY605708		*G. adspersum*
3776	*G. australe*	AY884182		*G. adspersum*
3777	*G. australe*	AY884183		*G. adspersum*
3778	*G. australe*	AY884184		*G. adspersum*
3779	*Ganoderma* sp.	EF060008		*G. adspersum*
3780	*G. adspersum*	EF060009		*G. adspersum*
3781	*G. adspersum*	EF060010		*G. adspersum*
3782	*G. adspersum*	EF060011		*G. adspersum*
3783	*Ganoderma* sp.	EF060012		*G. adspersum*
3784	*G. adspersum*	EU162053		*G. adspersum*
3785	*G. adspersum*	FJ655447		*G. adspersum*
3786	*G. adspersum*	FJ655449		*G. adspersum*
3787	*G. adspersum*	FJ655451		*G. adspersum*
3788	*G. adspersum*	FJ655452		*G. adspersum*

3789	*G. adspersum*	FJ655453		*G. adspersum*
3790	*G. australe*	FJ655458		*G. adspersum*
3791	*G. australe*	FJ655459		*G. adspersum*
3792	*G. australe*	FJ655460		*G. adspersum*
3793	*G. australe*	FJ655461		*G. adspersum*
3794	*G. australe*	FJ655462		*G. adspersum*
3795	*G. australe*	FJ655463		*G. adspersum*
3796	*G. australe*	FJ655464		*G. adspersum*
3797	*G. australe*	FJ655465		*G. adspersum*
3798	uncultured fungus	FJ820612		*G. adspersum*
3799	*G. australe*	FR686555		*G. adspersum*
3800	*G. applanatum*	GU731554		*G. adspersum*
3801	*G. adspersum*	JN176879		*G. adspersum*
3802	*G. adspersum*	JN176883		*G. adspersum*
3803	*G. adspersum*	JN176884		*G. adspersum*
3804	*G. adspersum*	JN176886		*G. adspersum*
3805	*G. adspersum*	JN176887		*G. adspersum*
3806	*G. adspersum*	JN176888		*G. adspersum*
3807	*G. adspersum*	JN176889		*G. adspersum*
3808	*G. adspersum*	JN176890		*G. adspersum*
3809	*G. adspersum*	JN176891		*G. adspersum*
3810	*G. adspersum*	JN176892		*G. adspersum*
3811	*G. adspersum*	JN176893		*G. adspersum*
3812	*G. adspersum*	JN176894		*G. adspersum*
3813	*G. adspersum*	JN176898		*G. adspersum*
3814	*G. adspersum*	JN176901		*G. adspersum*
3815	*G. adspersum*	JN176902		*G. adspersum*
3816	*G. adspersum*	JN176903		*G. adspersum*
3817	*G. adspersum*	JN176904		*G. adspersum*
3818	*G. adspersum*	JN176905	SH1723216.08FU	*G. adspersum*
3819	*G. adspersum*	JN176906		*G. adspersum*
3820	*G. adspersum*	JN176907	SH1723244.08FU	*G. adspersum*
3821	*G. adspersum*	JN176908		*G. adspersum*
3822	*G. adspersum*	JN222416	SH1723258.08FU	*G. adspersum*
3823	*G. adspersum*	JN222417		*G. adspersum*
3824	*G. adspersum*	JN222418		*G. adspersum*
3825	*G. adspersum*	JN588579		*G. adspersum*
3826	*G. adspersum*	JN588580		*G. adspersum*
3827	*G. adspersum*	JN588581		*G. adspersum*
3828	*G. adspersum*	JN588582		*G. adspersum*
3829	*G. adspersum*	JN588583		*G. adspersum*
3830	*G. adspersum*	JN588584		*G. adspersum*
3831	*G. adspersum*	JN588585		*G. adspersum*
3832	*G. adspersum*	JN588586		*G. adspersum*
3833	*G. adspersum*	KF605650		*G. adspersum*

3834	*G. adspersum*	KF605651	*G. adspersum*
3835	*G. adspersum*	KF605652	*G. adspersum*
3836	*G. adspersum*	KF975893	*G. adspersum*
3837	*G. adspersum*	KP941436	*G. adspersum*
3838	*G. adspersum*	KP941437	*G. adspersum*
3839	*G. adspersum*	KP941438	*G. adspersum*
3840	*G. adspersum*	KP941439	*G. adspersum*
3841	*G. adspersum*	KP941440	*G. adspersum*
3842	*G. adspersum*	KP941441	*G. adspersum*
3843	*G. adspersum*	KP941442	*G. adspersum*
3844	uncultured *Ganoderma*	KP974213	*G. adspersum*
3845	*Ganoderma* sp.	KT343298	*G. adspersum*
3846	*G. adspersum*	KT343300	*G. adspersum*
3847	*Ganoderma* sp.	KT343302	*G. adspersum*
3848	*G. adspersum*	KT343305	*G. adspersum*
3849	*G. adspersum*	KT343306	*G. adspersum*
3850	*G. adspersum*	KT343311	*G. adspersum*
3851	*G. adspersum*	KT343312	*G. adspersum*
3852	*G. adspersum*	KT343313	*G. adspersum*
3853	*G. adspersum*	KT343315	*G. adspersum*
3854	*G. applanatum*	KX449505	*G. adspersum*
3855	*G. adspersum*	KY977553	*G. adspersum*
3856	*G. adspersum*	MG066632	*G. adspersum*
3857	*G. adspersum*	MG279152	*G. adspersum*
3858	*G. adspersum*	MG279153	*G. adspersum*
3859	*G. adspersum*	MG279154	*G. adspersum*
3860	*G. adspersum*	MG706203	*G. adspersum*
3861	*G. adspersum*	MG706204	*G. adspersum*
3862	*G. adspersum*	MG706205	*G. adspersum*
3863	*G. adspersum*	MG706206	*G. adspersum*
3864	*G. adspersum*	MG706207	*G. adspersum*
3865	*G. adspersum*	MG706208	*G. adspersum*
3866	*G. adspersum*	MG706209	*G. adspersum*
3867	*G. adspersum*	MG706210	*G. adspersum*
3868	*G. applanatum*	MG706211	*G. adspersum*
3869	*G. applanatum*	MG706212	*G. adspersum*
3870	*G. australe*	MG835864	*G. adspersum*
3871	*Ganoderma* sp.	MK018627	*G. adspersum*
3872	*G. adspersum*	MK050589	*G. adspersum*
3873	*G. adspersum*	MK050589	*G. adspersum*
3874	*Ganoderma* sp.	MK050590	*G. adspersum*
3875	*G. adspersum*	MK050591	*G. adspersum*
3876	*G. adspersum*	MK050592	*G. adspersum*
3877	*Ganoderma* sp.	MK050593	*G. adspersum*
3878	*G. adspersum*	MK050594	*G. adspersum*

3879	*G. adspersum*	MK415250	*G. adspersum*
3880	*G. adspersum*	MK415256	*G. adspersum*
3881	*G. adspersum*	MK415257	*G. adspersum*
3882	*G. adspersum*	MK415262	*G. adspersum*
3883	*G. adspersum*	MK415265	*G. adspersum*
3884	*G. adspersum*	MK415268	*G. adspersum*
3885	*G. adspersum*	MK415280	*G. adspersum*
3886	*G. adspersum*	MK415288	*G. adspersum*
3887	*G. adspersum*	MK415289	*G. adspersum*
3888	*G. adspersum*	MK415291	*G. adspersum*
3889	*G. adspersum*	MK415292	*G. adspersum*
3890	*G. adspersum*	MK415293	*G. adspersum*
3891	*G. adspersum*	MK415294	*G. adspersum*
3892	*G. adspersum*	MK415300	*G. adspersum*
3893	*G. adspersum*	MK415301	*G. adspersum*
3894	*G. adspersum*	MK415302	*G. adspersum*
3895	*G. adspersum*	MK415303	*G. adspersum*
3896	*G. adspersum*	MK415306	*G. adspersum*
3897	*G. adspersum*	MK415307	*G. adspersum*
3898	*G. adspersum*	MK415315	*G. adspersum*
3899	*G. adspersum*	MK422152	*G. adspersum*
3900	*G. adspersum*	MN121020	*G. adspersum*
3901	*G. adspersum*	MN218201	*G. adspersum*
3902	*G. adspersum*	MN218202	*G. adspersum*
3903	*G. adspersum*	MN218203	*G. adspersum*
3904	*G. adspersum*	MN945139	*G. adspersum*
3905	*G. adspersum*	MT138615	*G. adspersum*
3906	*G. adspersum*	X78742/X78763	*G. adspersum*
3907	*G. adspersum*	MG835862	*G. adspersum*
3908	*Ganoderma* sp.	MN412653	*G. adspersum*
