# Supplementary material for: A global meta-analysis of ITS rDNA sequences from material belonging to the genus Ganoderma (Basidiomycota, Polyporales) including new data from selected taxa
Source: MycoKeys. 2020 Dec 1;75:71–143. doi: 10.3897/mycokeys.75.59872 (PMC7723883; doi:10.3897/mycokeys.75.59872)
Supplement: Supplementary material 2 — Figure S1 [file mycokeys-75-071-s002.pdf]

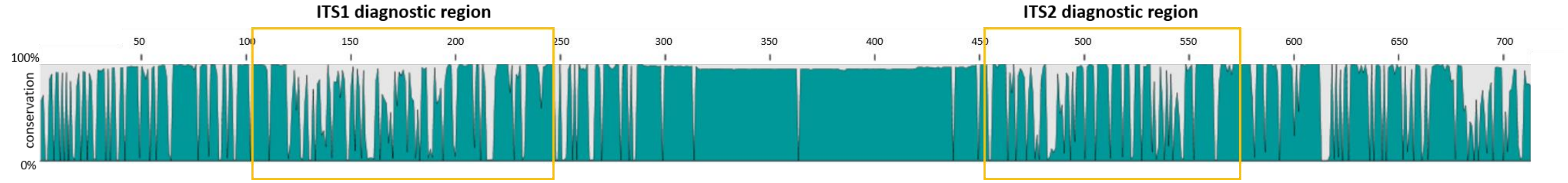

ITS1

	Position in main dataset alignment	103 113 123 133 143 153 163 173 183 193 203 213 223 233 243
	**Position in ITS1 region**	1 10 20 30 40 50 60 70 80 90 100 110 120 130 140
1	***G. oregonense* (27)**	tgcacttactgtggg--tttc-ag--a---tc-TGT-GA-AGCG--TG-CT-c-----ct-tgc-g-gggcttcgtg-aagc---gcgtctgt--gcctgcgt-tt-at----cacaaactc-yat--aaagtattagaatg
2	***G. tsugae* (54)**	...............--....-..--.-e-..-TGT-GA-AGCG--TG-CT-.-----..-...-.-..........-....---........--........-..-..----.........-y..--..............
3	***G. carnosum* (26)**	...............--....-..--.---..-TGT-GA-AGCG--TG-CT-.-----..-...-.-..........-....---........--........-..-..----.........-t..--..............
4	*G.* aff. *carnosum* (4)	...............--....-..--.---..-AGT-GA-AGC.--..-..-.-----..-...-.-..........-....---........--........-..-..----.........-t..--..............
5	***G. lucidum* (152)**	...............--....-..--.---.-..T-GA-AGCG--TN-CC-Ɏ-----Ɏ.-...-.-..........-....---........--........-..-..----.........-...--..............
6	***G. leucocontextum – G. weixiensis* (33)**	...............--....-..--.---..-.C-GA-AGCG--TG-C.-.-----..-...-.-..........-....---........--........-..-..----.........-t..--..............
7	*G. austroafricanum* (2)	...............--c...-..--.-g..--..-..-....--g-..-.-----t.-.a.-t-.a....-..a-GAGC---GTA....--........-..-..----.........-c..--..............
8	***G. hoehnelianum* (14)**	...............--CTTC-AG--T-Cg..--..-..-....--g-..-.-----t.-aa.-t-.a....-..a-g...---........--a.......-..-..----.........-t..--..............
9	***G. weberianum* (11)**	...............--....-.a--.-g..--..-r.-....--r.t..-.-----t.-.m.-y-.a....-..a-g...-g-........--........-..-..----.........-...--.......y......
10	***G. sichuanense* (19)**	...............--....-.a--.-g..--..-a.-....--r.t..-.-----t.-ya.-c-.a....-..a-g...-g-........--........-..-..----.........-...--.......y......
11	***G. carocalcareus* (13)**	...............--....-.A--A-CGTC--GT-KA-AGCG--GG-C.-.-----t.-.a.-t-.a....-..a-g...-g-.y......--........-..-..----.........ŧt..--..............
12	***Ganoderma* sp. A1 (17)**	.....ŧy........--....-.a--.-g..--..-..-..CG--GGTCT-------TT-Ta.-t-.a....-..a-g...-g-........--........-..-..----...m....-t..--..............
13	*G.* aff. *weberianum* (5)	...............--....-.a--.-k..--..-..-....--GGTCT-C-----TT-TAC-T-.a..y.-..a-g...-g-........--........-..-..----.........-c..--..............
14	***G. mexicanum* (16)**	...............--c...-..--.-sg..--.y-..-....--gG-CT-C-----TT-TAC-T-GAGCC.-..a-g...ar-.t......--........-..-..----.......y.-c..--..............
15	***G. parvulum* (22)**	...............--c...-..--.-sg..--.y-..-....--gG-CT-C-----TT-TAC-T-GAGCC.-..a-g...-r-.t......--........-..-..----.......y.-c..--..............
16	*Ganoderma* sp. A2 (2)	...........c...--c...-..--.-g.t--..-g.-....--g-..-.-----t.-.a.-.-.a....-..a-g.GCAGCGTGT....--.....t..-..-..----.........-t..--..............
17	***G. resinaceum* (126)**	...............--..c.-..--.-g.t--..g̶.-....--g-..-.-----t.-.a.-.-r....-..r-AAGCGGCG..c...--........-..-..----.........-t..--..............
18	***Ganoderma* sp. A3 (12)**	...............--..cy-..--.-g.t--..g.-....--g-..-.-----..-.at-.-.a....-..G-GATCGGCGT.c...--........-..-..----.........-t..--..............
19	*G.* aff. *sessile* (4)	...............--c...-..--.-g..--..-..-....--g-..-.-----t.-.a.-.-.a....-..A-GAGC---GTGC....--........-..-..----.........-t..--..............
20	*G.* aff. *polychromum* (8)	...............--c...-..--.-g..--..-..-....--g-..-.-----t.-.a.-.-.a....-..a-g...---........--.....GT-TT-AC---CACAAACAC-T.--..............
21	***G. polychromum* (19)**	...............--c...-..--.-g..--..-..-...r--g-..-.-----t.-.a.-.-.a....-..a-g...---........--........-..-..----.....ACACCTAT--..............
22	***G. sessile* (227)**	...............--c..s-..--.-g..--..-..-.r..--g-..-.-----t.-.a.-.-.a....-..r-g...---........--........-..-.C----CACAAACTC-TR.--..............
23	*G. concinnum* (2)	...............--c...-..--.tcg..--..-..-...A--GG-CT-C-----CTTTa.-c-...y..-.yrr...s---..k....--........-..-..----.........-t..--.......c......
24	***G. tuberculosum* (36)**	...............--c...-.G--ATTGTC--G.-r.-....--g-..-.------..-.a.-c-.........-.....-g-.t......--........-..-.c----t.......a-ta.ŧ-.......c......
25	*Ganoderma* sp. A4 (2)	...............--c...-..--.---..--..-..-.a..--g.-.c-.-----t.-ca.-c-.......-..G-AATC---GT....--........-..-..----.......c.-t..--..............
26	***G. wiiroense* (13)**	.....c.........--c...-.GGCATTATŧ--..c-.g-....--c.-t..-.-----..-ca.-t-..a...-....g....agt.ŧ.....ŧ--....a...-..-..----.........-t..--..a....c......
27	*G. flexipes* (7)	...ACACACTGTr..--c...-..--.---.y--..-..-..yr--g-..-.-----t.-.a.-r-.....c-........----.tg̶...y--........-..-..----.......c.-a..--.......c......
28	*Ganoderma* sp. A5 (7)	...............--c...-..--.---..--..-..-....--g-..-.-----t.-.a.-c-.....TGCGGAAGc---.t.....c--........-c.-..----.......c.-t..--.......cr.....
29	***G. philippii* (102)**	...............--c...-..--.---y.--..-..-....--g-..-.-----tT-TGC-T-GGGcty-.c.-.....---.t......--........-..-..----t.......-t..--.......c......
30	***G. lingzhi* (436)**	..............s--c..C-AG--A---TT--GC-..-g..a--c.-..-.-----t.-.a.-c-......-.c.-g...---rta....--........-..-..----.........-t..--......ac......
31	***G. curtisii* (141)**	...............--c...-..--.---..--..c-..-g..a--gk-..-.-----t.-.a.-c-....T-GCG-GAGC---GCAT....--........-..-..----.........-t..--r....ac......
32	***G. ravenelii* (12)**	...............--c...-.s--.---..--..c-..-g..a--s.-..-.-----t.-.a.-c-......-.c.-GAGT---GCAT....--.....r.-..-..----.........-t..--......ac......
33	***G. multiplicatum* (15)**	...............--c...-..--m---.y--..-..-.a..--g.-CC-C-----TT-TAT-c-..g̶..c-.c.-g...---.t......--........-..-..----.........-t..--.......c......
34	***G. destructans – G. dunense* (43)**	...............--c...-..--.---TC--GT-AA-AA..--g.t.c-.-----t.-.a.-c-....y.-.c.-g...---.t......--........-..-..----.........-t..--.......c......
35	*G. mizoramense* (3)	...............--c...-..--.---TC--GT-AA-AA..--g.t.c-.-----t.-.a.-c-......-.c.-g...---.t......--........-..-..----.........-t..--.......c......
36	***G. steyaertanum* (39)**	...............--c...-..--.---TC--VT-AA-AA..--g.t.c-s-----t.-.a.-c-.....y-.c.-g...---.y......--........-..-..----.........-t..--.......c....y.
37	***G. martinicense* (48)**	...............--c...-..--.---TC--GT-AA-AA..cgg.t.c-.-----t.-.a.-c-......-.c.gg...---.t......--........-..-..----y........-t..--.......c......
38	***G. multipileum* (130)**	...............--c...-..--.---TC--GT-AA-AA..--g.t.c-.-----t.-.a.-c-......-.c.-g...---.t......--........-..-..----.........-t..--.......c......
39	***Ganoderma* sp. A6 (15)**	.............ss.--c...-..--c---..--..-a.-...a--g.g.c-.-----t.-ca.-c-....TCCCA-GGA.---.t......--........-..-..----.........-t..--.......c.cm...
40	***G. tropicum* (31)**	...............--c...-.s--.---..--..-a.-...a--g.g.c-.-----t.-cAC-C-GGGCTTTGCA-gga.---.t......--........-..-..----.........-t..--.......c......
41	*Ganoderma* sp. A7 (3)	...............--c...-AG--A---TG--GT-a.-.s.a--g.g.c-y-----t.-cc.-c-......tsca-ggas---.t......--s.y.....-..-..----.c.......-t..--.......c......
42	*G. shanxiense* (2)	...............--....-..--g---..--..c-..-....--g-..-.-----tG-TAC-G-G-GC..-...-...---.ca....--........-..-..----.........-c..--..............
43	*Ganoderma* sp. B1 (4)	...............--.a..-..--.---..--..-..-....--g-..-.-----t.-TAATTAG.....-...-...t---.t..y...--....a...-..-..----.........-t..a-.......c......

44	*Ganoderma* sp. B2 (3)	...............--CATT-AG--A---_.--..-..-....--g.-..-.-----t.t.a.c.-.a..c.-...-....---.ta.....--....a...-..-..----.........-c..--.......c......
45	**_G. applanatum_ (158)**	...............--.a..-..--.---..--..-..-...G--TG-CT-YT----T.-.a.y.-.a....-...-....---.t.....--.........-..-..----.......a.-t..--........c......
46	**_G. neojaponicum_ (10)**	...............--..AT-GG--A---TC--GC-G.-r...--.r-..-s-----t.--------.ty.g.--c--r...---....y..-~~g~~.........-..-..----.........-tt.a-.......c......
47	*Ganoderma* sp. C1 (2)	...........k..k--..w.-g.--.---.y--.~~g~~-..-.a..--g.-CTTYC----TT--------.kc.gkttat-....---.....c..-g........-..-..----.........-tt.g-.......g......
48	*G. aridicola* (7)	...............--..a.-g.--.---..--.c-..-....--g.-CTTCC----TT--------.yc.g.ttat-....---.....c..-g........-..-..----.........-tt.g-..w....g......
49	*Ganoderma* sp. C2 (3)	...............--..a.-g.--.---..--.c-..-....--g.-CTTCC----TT--------.tc.g.ttaw-.mss---s...cs..-g........-..-..----..m......-tt.g-..r....~~g~~r..r..
50	**_G. enigmaticum_ – _G. thailandicum_ (10)**	...............--.ga.-r.--.---..--.c-a.-....--g.-CTTC-----TT--------GTC.g.-tat-....---..a....-g........-..-..----.........-tt.g-......c.......
51	**_G. casuarinicola_ (56)**	...........k...--.ga.-g.--.---..--.c-a.-....--gG-CT-C-----TT--------GTc.g.-tat-....---.ya....-g........-..-.y----.........-tt.g-......c.......
52	**_G. mbrekobenum_ (31)**	...............--TTWC-AG--A----SS-GT-.w-...r--r.~~g~~..-yy----t.--a.rrg.a....~~c~~...-....---.t......--r........-..-.y----y.........-tt.~~a~~-r..............
53	**_G. nasalanense_ (15)**	...............--y.a.-g.--.---..--.c-.g-..y.--g.-..-.-----t.-...-.-.a...c-.c.-...y---..t.....--....CGT-TT--T----C......a.-n..--........c......
54	**_G. sinense_ (66)**	...............--..a.-g.--r---ct--..-.g-....--g.-.t-c------t--~~g~~c-G-GAGCTC-GT.-....---.........--........t..-..----t......a.-t..--.....c........
55	*G. cupreum* (8)	...............--y.a.-g.--.---..--..-.g-....--g.-..-.-----t.-...-.-.a...c-.CG-AAGC---GCC.....--.........t..-..----a......a.-tt.--...............
56	*G. orbiforme* (5)	...............--..a.-g.--.---..--..-.g-....--g.-..-.-----t.-...-.-..........-...-....---..t.....--.........t..-..----a......a.-tTT--AAAGTATA.....
57	*G. subfornicatum* (9)	...............--..a.-g.--.---..--.T-GG-ACCG--GG-C.-.-----t.-...-.-......-...-....---..t.....--.........t..-..----a......a.-tt.--.......c......
58	**_G. mastoporum_ (122)**	...............--..a.-g.--.---y--..-.g-..y.--g.-..-.-----t.-y..-.-.......-...-....---..t.y...--..m.....t..-.d----a..r...a.-tt.a-.......k......
59	**_G. angustisporum_ (15)**	...............--.ygt-g.--.---.tgcGT-GT-AA---------------------------------AA..---.y...c..--........c..-.c----.......a.-t..a-........c......
60	*Ganoderma* sp. D1 (2)	...............--..at-g.--.---.tgcGC-GT-AA---------------------------------AA..---.....c..--........c..-.c----.......a.-tg.a-........c......
61	**_G. zonatum_ (84)**	...............--..at-g.--.---.tg...-.g-...T--CG-CT-CGCtc-t.-..a-c-.a.t..-.c.-....---.....k..--.........t..-.c----.......a.-t..t-.......c......
62	*Ganoderma* sp. D2 (2)	...............--..at-g.--.---....GT-GG-AACG--AG-..-.-----t.-..a-c-.a.w..-...-....---ay......--.........t..-.c----.......a.-t.c--...............
63	**_G. ryvardenii_ (16)**	...............--..at-g.--.---TCGTGC-Gr-....--g.-..-.-----t.-..a-c-.a.t..-.c.-....---.........--.........t..-.c----.......a.-t.c--...............
64	**_G. boninense_ (61)**	.......y......--..at-..--.---yyg...-.g-....--a.-..-.~~gtt~~yGTT-TGA-C-RAGTTy-.y.-....---..........--....y..t..~~t~~..----.......a.-t..--...............
65	***Ganoderma* sp. D3 (12)**	...............--..at-..--.---.GGCGT-GGT.a..--a.-..-.-----t.-.aa-c-.a....-.y.-....---.y.....--a.......t..-.c----.......a.-t..--...............
66	**_G. williamsianum_ (42)**	...............-CTT-C-AG--G---TC--..-..-r...--a.-.c-.k----t.-~~tag~~-c-.....c-.c.-g...---..a.....--.........-..-..----.......c.-t..--......ayc.....
67	***Ganoderma* sp. E1 (23)**	..............GTTTTAC-G.--g---..--..-..-mat.--g.-..-.g----t.-.attc-......-..t-g...---..act...t~~t~~.......-..-..----.........-t..--...............
68	***Ganoderma* sp. E2 (37)**	..............GTTTTAC-G.--g---..--..-..-.atk--g.-..-.g----t.-cattc-....y-..t-g...---..act...t-........-..-..----.........-..--...............
69	**_G._ aff. _gibbosum_ (46)**	...............-t..a.-g.--g---..--.T-GA-AACG--GG-CT-CG----t.-.attc-......-..t-g...---..act...t-........-..-..----.........-t..--.......c.t....
70	*G. eickeri* (4)	...............-t..a.-g.--g---..--.T-GA-AACG--GG-CT-CG----t.-.attt-......-..t-g...---..act...t-........-..-..----.........-t..--.......c......
71	**_G. gibbosum_ (107)**	...............-t..a.-g.--g---..--.T-GA-AASG--GG-CT-YG----t.-yattc-......-..t-g...---..act...y-t.......-..-.y----.........-t..--r......y......
72	**_G. ellipsoideum_ (73)**	...............-t..a.-g.--g---..--.T-GA-AASG--GG-CT-CG----t.-yrttc-......-..t-g...---..act...y-k.......-..-..----.........-tr.--.......c......
73	*Ganoderma* sp. E3 (7)	...............-t..a.-g.--g---..--.T-GA-ARCG--GG-CT-YG----t.-.rttc-......-..y-g...---..act...t-........-..-..----.........-t..--.......c......
74	***Ganoderma* sp. E4 (13)**	...............-t..a.-g.--g---..-RT-TA-AACG--g.-..-.g----t.-.attc-......-..t-g...---..act...t-........-..-..caca.......a.-t.y--..............
75	*G. knysnamense* (4)	...............-t..g.-g.--g---.C--GC-GA-AACG--a.-..-.g----t.-ca.tc-......-..c-g...---..act...t-........-..-..----.........-t..--..............
76	*G. mutabile* (2)	...............-t..a.-g.--g---.C--GC-GA-AATG--g.-..-.g----t.-.attc-......-..t-g...---..act...t-.........-.c-..----.........-tg.--..............
77	*G. cupreolaccatum* (1)	.C......-.......t..a--g.--g--g..--.c-..-.a..--GG-CC-CG----TT-Tattc-......-..t-g...---..act...t-........-..-..----.........-t..--..............
78	**_G. pfeifferi_ (17)**	...............-t..r.-g.--g--~~t~~..--.c-..-.a..--GG-CC-CG----TT-Tattc-......-..t-g...---..act...t-........-..-..----.........-t..--..............
79	*G. chocoense* (1)	...............--..a.-..--.---..--..-..-....--g.-TC-CT----TT--G-Tt-.....c-.....c-...-....---..ac....--...c....-..-..----.........-t....c....c......
80	*G. podocarpense* (2)	...............--..a.-..--.---..--..-..-....--g.-..-CT----TT--G-CC-.....c-.c.-g...---..ac....--.........-..-..----.........-t..--.c.....c......
81	*Ganoderma* sp. E5 (8)	...............--..a.-..--.---..--..-..-.a..--g.-..-.t----TT--G--CTGAG..c-.ca-g...---..ac....--...c....-..-..----.........-t..--..............
82	***Ganoderma* sp. E6 (35)**	...............--..a.-..--.---..--.c-..-.a..--g.-..-.t----tc--A--CYGAGCYY-GC.-g..y---..ac....--...c....-..-..----.......c.-t..--.......c......
83	**_G. australe_ (76)**	...............-t..a.-s.--g---..--.C-GA-AACG--KG-CT-CG----t.-.attc-......-...-g...---..act...t-........-..-..----.........-c..~~t~~-..............
84	***Ganoderma* sp. E7 (16)**	...............--..a.-..--g--a..--.c-..-.a..--g.-.w-.t----t.--a--cg.....c-~~g~~cr-g...---...ct...--...c....-..-..----.....CCC-CAT--G............
85	*G.* aff. *adspersum* (9)	...............-t..a.g..------..--.c-g.-.a.G--GG-CC-CG----TT-Cattc-.....c-..t-g...---..rct...t-........-..-..----.........tt..--.........r....
86	**_G. adspersum_ (142)**	...............-t..a.r..------..--.c-r.-.a.A--GG-CC-CG----TT-Cattc-......-...-g...---..rct...t-........-..-..----.........-t..--.........r....

ITS2

<table>
<tr><td></td><td>Position in main dataset alignment</td><td>452 462 472 482 492 502 512 522 532 542 552 562 572</td></tr>
<tr><td></td><td>Position in ITS2 region</td><td>1 10 20 30 40 50 60 70 80 90 100 110 120</td></tr>
<tr><td></td><td></td><td>| | | | | | | | | | | | |</td></tr>
<tr><td>1</td><td>G. oregonense (27)</td><td>aa--tcttcaa-cct-acaa-gCC---TTT-----GCG-GG-T-TTGT-AGGC-TTGGAC-ttgga-ggc--ttg-tcggc-cct-t-t-gtc--ggtc-ggctcctct--taaatgcattagct</td></tr>
<tr><td>2</td><td>G. tsugae (54)</td><td>w.--.......-...-....-.CC---TTT-----GCG-GG-T-WTGY-RGGC-TTGGAC-.....-...--...-.....-...-.-.-...--....-.........--..............</td></tr>
<tr><td>3</td><td>G. carnosum (26)</td><td>..--.......-...-...A-GCC----TT-----GC--..-.-....-....-......-.....-...--...-.....-...-y-~~t~~-~~gtc~~--....-.........--..............</td></tr>
<tr><td>4</td><td>G. aff. carnosum (4)</td><td>..--.......-...-.s..-...---...-----..G-GG-T-TTGT-AGGC-TTGGAT-.....-...--...-.....-...-.-.-...--....-.........--..............</td></tr>
<tr><td>5</td><td>G. lucidum (152)</td><td>..--.......-...-....-...---...-----..--..-.-....-....-......-.....-...--...-.....-.bb-y-.-...--....-.........--..............</td></tr>
<tr><td>6</td><td>G. leucocontextum – G. weixiensis (33)</td><td>..--.......-...-....-..t---...-----..--..-.-....-....-......-.....-...--...-.....-...-c-.-~~g~~.--....-.........--..............</td></tr>
<tr><td>7</td><td>G. austroafricanum (2)</td><td>..--.......-...-...g-ACC---TTT-----GT.-..-.-....-....-......-.....-...--...-.....--.g-.g.-t..--....-.........--..............</td></tr>
<tr><td>8</td><td>G. hoehnelianum (14)</td><td>..--.......-...-...g-a..---CTT-----GTG-GG-TT...-....-......-.....-...--...-yc...--.g-.g.-t..--....-.........--..............</td></tr>
<tr><td>9</td><td>G. weberianum (11)</td><td>..--.......-...-..rg-a..---...-----...-..-.t....-....-......-.....-...--...-....y--.g-.g.-t..--....-.........--..............</td></tr>
<tr><td>10</td><td>G. sichuanense (19)</td><td>..--.......-...-...g-a..---...-----...-..~~t~~.t....-....-......-.....-...--...-.....--.g-.g.-t..--....-.........--..............</td></tr>
<tr><td>11</td><td>G. carocalcareus (13)</td><td>..--.......-...-...g-r..---...-----...-..-.t....-.r..-......-.....-...--...-.....--.g-.g.-t..--....-.........--..............</td></tr>
</table>

12	**_Ganoderma_ sp. A1 (17)**	..--.......-...-..gg-a..---...-----...-..-.t....-....-......-.....-...--...-.....--CG-TCT-TTC--....g.........--..............
13	_G._ aff. _weberianum_ (5)	..--.......-...-...g-a..---...-----.~~e~~.-..-.-~~t~~...-....-......-.....-...--...-.....--.g-.g.-t.y--....~~g~~.........--..............
14	**_G. mexicanum_ (16)**	..--.......-...-...g-a..---...-----...-..-.-....-....-......-.....-...--...-.....--.g-.g.-t.C--GGCC-GGCTCCTCT--TAAATGC.......
15	**_G. parvulum_ (22)**	..--.......-...-...g-a..---...-----...-..-.-~~t~~...-....-......-.....-...--...-.....--.g-.g.-t.C--GGCC-GGCTCCTCT--TAAATGC.......
16	_Ganoderma_ sp. A2 (2)	..--.......-...-...g-a..---...-----...-..-.-....-....-......-.....-..C-TTTG-TCGGC--CG-Tg.-t.c--...t-.........--..............
17	**_G. resinaceum_ (126)**	..--.......-...-...g-a..---...-----...-..-.-....-....-......~~t~~.....-...--...-.....--.r-.g.-t.y--....-.........--..............
18	**_Ganoderma_ sp. A3 (12)**	..--.......-...-ACAG-ATC---Ttt-----...-..-.-....-....-......~~t~~.....~~a~~..y--.k.-y....--.g-kg.-d..--....-.........--..............
19	_G._ aff. _sessile_ (4)	..--.......-...-...g-a..---...-----...-..-.-....-....-......t.....-...--...-.....--.g-.g.-t.t--....-.........--..............
20	_G._ aff. _polychromum_ (8)	..--.......-...-...g-a..---...-----...-..-.-....-....-......-.....-...--...-.....--.GGTGT-TTC--....-.........--..............
21	**_G. polychromum_ (19)**	..--.......-...-...g-a..---...-----...-..-.-....-....-......-.....-...--...-.....--.g-.g.-t..--....-.........--..............
22	**_G. sessile_ (227)**	..--.......-CTT-ACAA-A..---...-----...-..-.-....-....-......-.....-...--...-.....--.r-.g.-t..--..yc-.........--..............
23	_G. concinnum_ (2)	..--.......-...-....-GCT---TGT-----GT--G.-.-....-....-......-.....-...--...-.....--y.-.-...--....-.........--c.............
24	**_G. tuberculosum_ (36)**	..--y......-...-g...-..t---tat-----.t--..-.-....-....-......-.....-...-t...-....C--CA-T-G-CCC--....-.........--c.............
25	_Ganoderma_ sp. A4 (2)	..--.......-...-..g.-..t---...-----.t--..-.-....-....-......-TTGGA-GGC--TTG-CT...--.g-.-.c.t--....-.........--..............
26	**_G. wiiroense_ (13)**	..--.......-...-..g.-..t---...-----.t--..-~~t~~....-....-~~t~~......-.....-...--...-.....--.g-.-.c.t--....-...TTCTCT--TA............
27	_G. flexipes_ (7)	..--.......-...-..g.-..t---...-----.t--..-.-.y..-....-......-.....-...--...-.....--.G-T-T-RCT--G...-.........--c.............
28	_Ganoderma_ sp. A5 (7)	..--.......-...-....-..t---...-----..--..-.-....-....-......-CTGGA-GGC--TTG-Ct...--.g-.-.c.t--....-.........--c.............
29	**_G. philippii_ (102)**	..--.......-...-....-.CT---TTT-----GT--GG-YTTT..-....-......-.....-...--...-.....--.r-.-.c..--....-.........--..............
30	**_G. lingzhi_ (436)**	..--..y....~~e~~...-....-..t---...-----.t--..-.-....-....-......-.....-...--...-.CGGC--CG-T-T-AT.--....-.........--..............
31	**_G. curtisii_ (141)**	m.--.......-...-....-..t---...-----.t--..-.-...y-....-......-.....-...--...-.CGGC--CG-T-T-ATk--....-.........--y.............
32	**_G. ravenelii_ (12)**	..--.......-...-....-..t---...-----.t--..-h-...y-....-......-.....-...--...-.CGGC--CG-T-T-ATt--....-.........--..............
33	**_G. multiplicatum_ (15)**	..--.......-...-....-..t---...-----..--..-.-....-....-......-.....-...--...-.....--.g-.-.c.t--....-.........--..............
34	**_G. destructans_ – _G. dunense_ (43)**	..--.......-...-....-..t---...-----.t--..-.-....-....-......-.....-...--...-.....--.g-.-.c.t--....-.........--..............
35	_G. mizoramense_ (3)	..--.......-...-....-..t---...-----.t--..-.-c...-....-......-.....-...-t...-....T--CG-C-T-CTT--GGCT-.........--..............
36	**_G. steyaertanum_ (39)**	..--.......-...-....-..t---...-----.y--..-.-y...-....-......-.....-...-t...-.....--.g-C-T-CTT--GGCC-r........--..............
37	**_G. martinicense_ (48)**	..--.......-...-g...-..t---...----t.t--..-.-....-....-......-.....-...--...-.y...--CA-T-T-CTT--G...-.........--..............
38	**_G. multipileum_ (130)**	..--.......-...-GCAA-GCT---TTT-----Gt--..-.-....-....-......-.....-...--...-.....--.g-.-.s.y--....-.........--..............
39	**_Ganoderma_ sp. A6 (15)**	..--.......-...-........---...-----..--..-.t....-....-......-.....-...--...-.....--.g-.-.c.t--....-.CTCCTCTCT.............
40	**_G. tropicum_ (31)**	..--.......-...-....-r..---...-----..--..-.t....-....-......-.....-...--...-.....--.g-.-.c.t--....-.........--..............
41	_Ganoderma_ sp. A7 (3)	..--..a...m-...-m...-..T-TCTTT----A.--..-.t....-....-......-.....-...--...-.....--.g-.-.c.t--....-.........--..............
42	_G. shanxiense_ (2)	..--y......-...-..AA-GCT----TT-----GT--A.----------..-....-......-.....-...--...-.t...--.g-.-.a.t--....-a........--....cra.......
43	_Ganoderma_ sp. B1 (4)	..--.......-...-....-..t---...-----.t--AG-C-TTGT-C...-......-.....-...--...-.....--t.-.-.a.t--....-....y...--..............
44	_Ganoderma_ sp. B2 (3)	..--.......-...-....-..t---...-----.C--GG-C-CTGC-....-......-.....-...--...-.....--.-.-.-a.tt-....-.........--..............
45	**_G. applanatum_ (158)**	..--.......-...-aTAA-GCT---TTK-----GT--..-.-....-....-......-.....-...--...-.....--.-.-.g-a.y--....-.........--..............
46	**_G. neojaponicum_ (10)**	..--.......~~a~~...-g...-..t-t-...---~~tt~~.t--..-y-.k.y-....-......-....A-GGTGTTTG-....-.-..-.tw-aat--....-.........--c.............
47	_Ganoderma_ sp. C1 (2)	..--.......-.t.-g...-..t-c-...-----....a..-....g-....-......-.....g..--...-.....--CT-TTT-AATG-GG..-......-..--..............
48	_G. aridicola_ (7)	..--.......-.t.-g...-..t-c-...-----...ga.-.~~t~~....-....-......-.....g..--...-.....--CT-TTT-AATG-GG..-.........--..............
49	_Ganoderma_ sp. C2 (3)	..--.......-.t.-g...-..t-c-...-----...ga.-.t....-....-......-.....g..--...-.....--CT-TTT-AATG-GG..-.........--..............
50	**_G. enigmaticum_ – _G. thailandicum_ (10)**	..--.......-.TT-GCAA-CCt-c-...-----...ga.-....-....-......-.....g..--...-.y...--.-.-.-aay--....-.........--..............
51	**_G. casuarinicola_ (56)**	..--.......-.TT-GCAA-CCt-c-...-----...gak-....-....-......-.....g..--...-.y...--.-.-.-aat--....-.........--...........s..
52	**_G. mbrekobenum_ (31)**	..--.......-...-g...-..t-t--..----aatc-r.-.-....-....-.....t-....A-GGCTATT.-.....--.-.-.-a.y--....-.........--....y.y.......
53	**_G. nasalanense_ (15)**	..--.......-.t.-....-...-t-.g.----aat.-..-.-....-....-......-.....-...--...-....T--CT-T-TAATA--....-.........--..............
54	**_G. sinense_ (66)**	..--.......-...-....-.gt-c-...--GTAAAG-GC-.-..t.-....-......-.....-...--...-....t--.-.-.-a.a--...y-.........--..............
55	_G. cupreum_ (8)	..--.......~~a~~.-....-.t-t-...----aat.-..-.-TTGC-GGGC-.....-.....-...--....-.... t--.-.-.-a.t--....-.........--c.............
56	_G. orbiforme_ (5)	..--.......-CCT-ACAA-GCT-T-TTT---AAAt.-.t-.-....-....-......-.....-...--...-....t--.-.-.-a.t--....-.........--c...c.........
57	_G. subfornicatum_ (9)	..--.......-TCT-ACAA-GCT-T-TT.---.....-..-.-....-....-......-.....-...--...-....c--.-.-.-a.t--....-.........--c...c.........
58	**_G. mastoporum_ (122)**	..--.......-...-....-..T-T-TTT----ARYG-RK-T-TTGT-AGG.-......-.....-...--...-....y--..-.-k-ayt--....-.........--c.....r.......
59	**_G. angustisporum_ (15)**	..--.......-...-....-..t-t-...~~a~~aataATG-GC-T-WGT.-....-......-.....-...--...-....t--.-.-.-a.t--....-.........--y...c.........
60	_Ganoderma_ sp. D1 (2)	..--.......-...-....-..T-T-TTT-----GTG-GC-T-TT..-....-......-.....-...--...-.....--y.-.-.a..--....-.........--c...c.........
61	**_G. zonatum_ (84)**	..TCTCTTCA.-...-...g-t.t-c-...-----...-.t-.t....-....-......-.....-...--...-....t--.-.-.-act--.a..-.........--c.............
62	_Ganoderma_ sp. D2 (2)	..--.......-...-....-TGT-C-TTT-----GCG-.t-.t....-....-......-.....-...--...-....t--.-.-.-act--.a..-.........--c.............
63	**_G. ryvardenii_ (16)**	..--.......-...-....-t.t-c-...-----.t-..-.t....-....-......-.....-...--...-....t--CT-TTA-ACT--....-.........--c.............
64	**_G. boninense_ (61)**	..--.......-...-...A-TCT-C-TTT-----GY.-.t-.t....-....-......-.....-...--...-....t--..-.t.-a.t--....-........y--c.....y.......
65	**_Ganoderma_ sp. D3 (12)**	..--.......-...-...A-TCT-C-TTT-----GC.-.t-.t....-....-......-.....-...--...-....t--..-.t.-a.t--....-.........--c.............
66	**_G. williamsianum_ (42)**	..--.......-...-....-..t-t-CTT-AATYG---A.-.-....d....-.....y-.....-...--y..-y...t.--..---...m--....-.........--..............
67	**_Ganoderma_ sp. E1 (23)**	..--.......-t...ATRA-GCT-T-CT.-aatt.-.-a.-.-.......-..n.-.....y-.....-...--...-....t.--..-.-.aca--....-.........--..............
68	**_Ganoderma_ sp. E2 (37)**	..--.......-t..-....-..t-t-c.T--A-TGKG-AG-.-....-....-......-.....-...--...-....t.--..-.-.aca--....-.........--c.............
69	**_G._ aff. _gibbosum_ (46)**	..--.......-t..-....-a.t-t-c..--a-t.-.-a.-.-....-....-......-.....-...--...-....TC-CCT-T-TACA--....-.........--..............
70	_G. eickeri_ (4)	..--.......-t..-....-a.t-t-c..--a-t.-.-a.-.-....-....-......-.....-...--...-....TC-CTT-T-TACA--....-.......y.--..............
71	**_G. gibbosum_ (107)**	..--.......-t..-....-a.t-t-y..--a-t.-.r.-k-....-r...-.y....-.....-...--...-....TC-CYT-T-TACR~~a~~-....-.........--..............
72	**_G. ellipsoideum_ (73)**	..--.......-t..-....-a.t-t-...--a-y.-..-c-....-....-......-.....-...--...-....TC-CCT-T-TACAa-....-.........--.........r...
73	_Ganoderma_ sp. E3 (7)	.~~-~~--.......-.y.-r...-..t~~t~~t~~t~~c..--att.aa-..-c~~t~~....-....-.....t-.....-..--...-TCGGACTW~~e~~.-a-.a.r--....-.........--..............

74	***Ganoderma* sp. E4 (13)**	..--.......-.y.-....-..ttttc..--aty.aa-..-c-....-....-.....t-.....-...--...-TCGGACTT-.-at.a.acg....-.........--....w........
75	*G. knysnamense* (4)	..--.......-...-....-..t-t-...-----.-y-..-c-....-....-......-.....-...--...~~g~~....-.--..-.-.GTA--GGTC-GGCTCCTCT--TAAAC........
76	*G. mutabile* (2)	..--.......-...-....-..t-t-...-----.-t-..-.-.c..-....t......-.....-...--...-....-.--..-.-...A--GGTC-GGCTCCTCT--TAAATGT......
77	*G. cupreolaccatum* (1)	..--.......-...-....-..t-t-...-----.-t-..-.-.c..-....-......-.....-...--...-...G-C--CT-T-TGTC--....-.........--......t.......
78	***G. pfeifferi* (17)**	..--.......-...-....-..t-t-...-----.-t-..-.-.c..-....-......-.....-...--...-...G-C--CT-T-TGTC--r...-.........--.............
79	*G. chocoense* (1)	..--.......-...-....-..t-t-...-----.-t-..-.-....-....-......-.....-...--...-....-.--..-.-.a.t--....-.........--.............
80	*G. podocarpense* (2)	..--.......-...-....-.CT-T-TTT----TG-T-..-.-....-....-......-.....-...--...-....-.--..-.-.a.t--....-.........--.............
81	*Ganoderma* sp. E5 (8)	..--.......-...-....-..t-c-...---tTG-T-GG-C-TC..-....-.....t-.....-...--...-.t..-.--..-.-.a.t--....-.........--.............
82	***Ganoderma* sp. E6 (35)**	..--y......-.t.-rc..-..T-C-TTT-----GCGGGG-.-....-....-......-.....-...--...-....-.--..-.-kaat--....-.........--....y........
83	***G. australe* (76)**	..--.......-.t.-....-..T-C-TTT-----GCGGGG-.-....-....-......-.....-...--...-....-.--..-.-.aay--....-.........--.............
84	***Ganoderma* sp. E7 (16)**	..--.......-.t.-acg.-..t-t-c..-----...a..-.-....-....-......-.....-...--...-...G-T--CT-T-TACA--....-.........--.............
85	*G.* aff. *adspersum* (9)	..--.......-.t.-acg.-.CT-T-CTT-----GCGG..-.-....-....-......-.....-...--...-....-.--..-.-.aaa--....-.........--.............
86	***G. adspersum* (142)**	..--.......-.t.-acg.-..t-t-c..-----...AGG-T-TTGT-AGGG-......-.....-...--...-....-.--..-.-.ara--....-.........--.............

Supplementary Figure S1. Hypervariable regions of potential diagnostic value in ITS1 and ITS2 spacers for the *Ganoderma* taxa appearing in Table 1, Supplementary Table S2 and Fig. 3. Taxa represented by ≥ 10 sequence entries appear in bold typeface (numbers in parentheses denote number of entries corresponding to each taxon). Selected bases or sequence fragments of potential diagnostic value are presented for each spacer in capital underlined characters. In the alignment performed, the hypervariable fragment in ITS1 begins with –TGCAC– and ends with –GAATG– while in ITS2 begins with –AATCT– and ends with –TAGCT–. Numbering of base positions was according to (a) the alignment of sequences included in the main dataset (DS) used for the purposes of this study and (b) the position from the start of ITS1 or ITS2 diagnostic regions as these appear on the upper part of the graph.
