# Supplementary material for: A global meta-analysis of ITS rDNA sequences from material belonging to the genus Ganoderma (Basidiomycota, Polyporales) including new data from selected taxa
Source: MycoKeys. 2020 Dec 1;75:71–143. doi: 10.3897/mycokeys.75.59872 (PMC7723883; doi:10.3897/mycokeys.75.59872)
Supplement: Supplementary material 3 — Figure S2a [file mycokeys-75-071-s003.pdf]

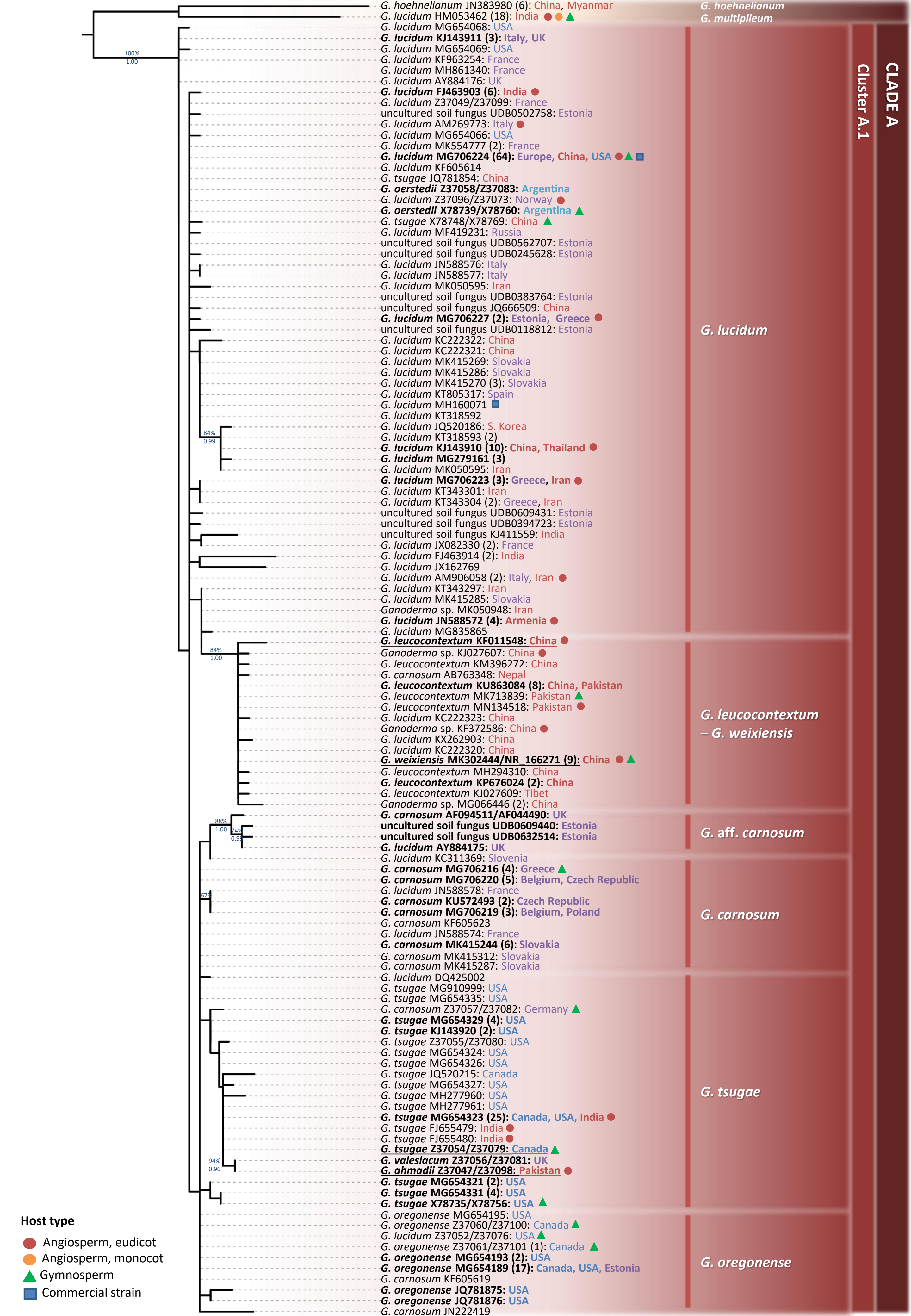

G. hoehnelianum JN383980 (6): China, Myanmar
G. lucidum HM053462 (18): India
G. lucidum MG654068: USA
G. lucidum KJ143911 (3): Italy, UK
G. lucidum MG654069: USA
G. lucidum KF963254: France
G. lucidum MH861340: France
G. lucidum AY884176: UK
G. lucidum FJ463903 (6): India
G. lucidum Z37049/Z37099: France
uncultured soil fungus UDB0502758: Estonia
G. lucidum AM269773: Italy
G. lucidum MG654066: USA
G. lucidum MK554777 (2): France
G. lucidum MG706224 (64): Europe, China, USA
G. lucidum KF605614
G. tsugae JQ781854: China
G. oerstedii Z37058/Z37083: Argentina
G. lucidum Z37096/Z37073: Norway
G. oerstedii X78739/X78760: Argentina
G. tsugae X78748/X78769: China
G. lucidum MF419231: Russia
uncultured soil fungus UDB0562707: Estonia
uncultured soil fungus UDB0245628: Estonia
G. lucidum JN588576: Italy
G. lucidum JN588577: Italy
G. lucidum MK050595: Iran
uncultured soil fungus UDB0383764: Estonia
uncultured soil fungus JQ666509: China
G. lucidum MG706227 (2): Estonia, Greece
uncultured soil fungus UDB0118812: Estonia
G. lucidum KC222322: China
G. lucidum KC222321: China
G. lucidum MK415269: Slovakia
G. lucidum MK415286: Slovakia
G. lucidum MK415270 (3): Slovakia
G. lucidum KT805317: Spain
G. lucidum MH160071
G. lucidum KT318592
G. lucidum JQ520186: S. Korea
G. lucidum KT318593 (2)
G. lucidum KJ143910 (10): China, Thailand
G. lucidum MG279161 (3)
G. lucidum MK050595: Iran
G. lucidum MG706223 (3): Greece, Iran
G. lucidum KT343301: Iran
G. lucidum KT343304 (2): Greece, Iran
uncultured soil fungus UDB0609431: Estonia
uncultured soil fungus UDB0394723: Estonia
uncultured soil fungus KJ411559: India
G. lucidum JX082330 (2): France
G. lucidum FJ463914 (2): India
G. lucidum JX162769
G. lucidum AM906058 (2): Italy, Iran
G. lucidum KT343297: Iran
G. lucidum MK415285: Slovakia
Ganoderma sp. MK050948: Iran
G. lucidum JN588572 (4): Armenia
G. lucidum MG835865
G. leucocontextum KF011548: China
Ganoderma sp. KJ027607: China
G. leucocontextum KM396272: China
G. carnosum AB763348: Nepal
G. leucocontextum KU863084 (8): China, Pakistan
G. leucocontextum MK713839: Pakistan
G. leucocontextum MN134518: Pakistan
G. lucidum KC222323: China
Ganoderma sp. KF372586: China
G. lucidum KX262903: China
G. lucidum KC222320: China
G. weixiensis MK302444/NR_166271 (9): China
G. leucocontextum MH294310: China
G. leucocontextum KP676024 (2): China
G. leucocontextum KJ027609: Tibet
Ganoderma sp. MG066446 (2): China
G. carnosum AF094511/AF044490: UK
uncultured soil fungus UDB0609440: Estonia
uncultured soil fungus UDB0632514: Estonia
G. lucidum AY884175: UK
G. lucidum KC311369: Slovenia
G. carnosum MG706216 (4): Greece
G. carnosum MG706220 (5): Belgium, Czech Republic
G. lucidum JN588578: France
G. carnosum KU572493 (2): Czech Republic
G. carnosum MG706219 (3): Belgium, Poland
G. carnosum KF605623
G. lucidum JN588574: France
G. carnosum MK415244 (6): Slovakia
G. carnosum MK415312: Slovakia
G. carnosum MK415287: Slovakia
G. lucidum DQ425002
G. tsugae MG910999: USA
G. tsugae MG654335: USA
G. carnosum Z37057/Z37082: Germany
G. tsugae MG654329 (4): USA
G. tsugae KJ143920 (2): USA
G. tsugae Z37055/Z37080: USA
G. tsugae MG654324: USA
G. tsugae MG654326: USA
G. tsugae JQ520215: Canada
G. tsugae MG654327: USA
G. tsugae MH277960: USA
G. tsugae MH277961: USA
G. tsugae MG654323 (25): Canada, USA, India
G. tsugae FJ655479: India
G. tsugae FJ655480: India
G. tsugae Z37054/Z37079: Canada
G. valesiacum Z37056/Z37081: UK
G. ahmadii Z37047/Z37098: Pakistan
G. tsugae MG654321 (2): USA
G. tsugae MG654331 (4): USA
G. tsugae X78735/X78756: USA
G. oregonense MG654195: USA
G. oregonense Z37060/Z37100: Canada
G. lucidum Z37052/Z37076: USA
G. oregonense Z37061/Z37101 (1): Canada
G. oregonense MG654193 (2): USA
G. oregonense MG654189 (17): Canada, USA, Estonia
G. carnosum KF605619
G. oregonense JQ781875: USA
G. oregonense JQ781876: USA
G. carnosum JN222419
100%
1.00
84%
0.99
84%
1.00
88%
1.00
74%
0.95
67%
94%
0.96
G. hoehnelianum
G. multipileum
G. lucidum
G. leucocontextum – G. weixiensis
G. aff. carnosum
G. carnosum
G. tsugae
G. oregonense
Cluster A.1
CLADE A
Host type
Angiosperm, eudicot
Angiosperm, monocot
Gymnosperm
Commercial strain

Supplementary Figure S2a. Phylogenetic reconstruction of the genus *Ganoderma* inferred from ML analysis based on ITS sequence data (pDS1a; Table 2) for Clade A, Cluster A.1. ML bootstrap values (BS) ≥ 65% and Bayesian posterior probabilities (BPP) ≥ 0.95 are shown. Sequences names on the left appear as initially labelled, and are followed by the respective GenBank/ENA/DDBJ or UNITE accession no., while the total number of identical entries corresponding to a particular sequence is placed in parentheses, followed by the type of host plant (legend for the colored shapes is found at the lower left side of tree) and geographic origin of the respective material (the latter appears in different font color depending on the continent of provenance; see also Tables 1 and S2). Species names on the right correspond to those inferred in this study evaluated in conjunction with literature data. Sequences included in the respective Figure (Fig. 4) of the manuscript appear in bold typeface, while underlined sequences are those originating from type material. Scale bar: 0.01 nucleotide substitutions per site.
