# Supplementary material for: A global meta-analysis of ITS rDNA sequences from material belonging to the genus Ganoderma (Basidiomycota, Polyporales) including new data from selected taxa
Source: MycoKeys. 2020 Dec 1;75:71–143. doi: 10.3897/mycokeys.75.59872 (PMC7723883; doi:10.3897/mycokeys.75.59872)
Supplement: Supplementary material 4 — Figure S2b [file mycokeys-75-071-s004.pdf]

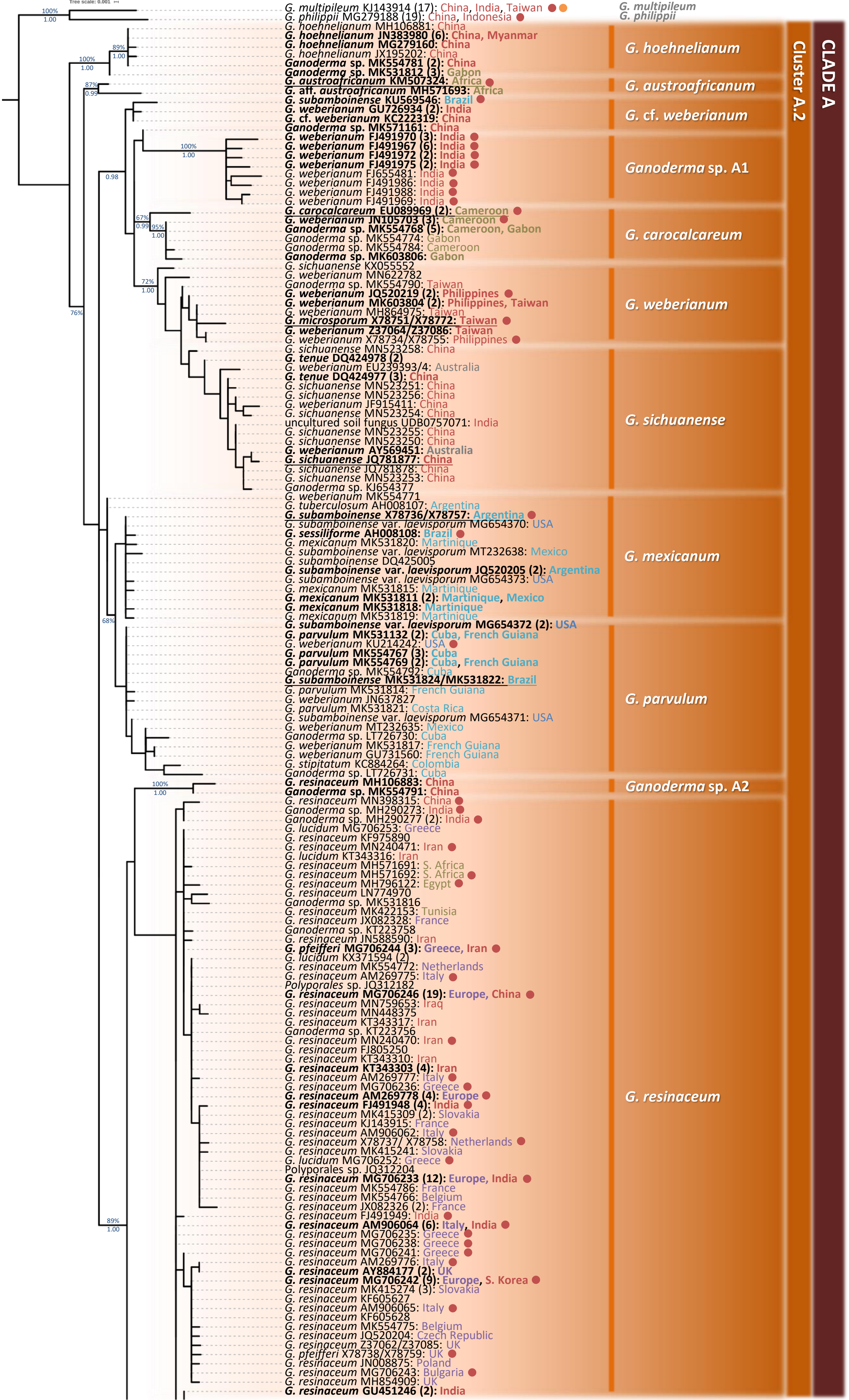

Tree scale: 0.001
G. multipileum KJ143914 (17): China, India, Taiwan
G. philippii MG279188 (19): China, Indonesia
G. hoehnelianum MH106881: China
G. hoehnelianum JN383980 (6): China, Myanmar
G. hoehnelianum MG279160: China
G. hoehnelianum JX195202: China
Ganoderma sp. MK554781 (2): China
Ganoderma sp. MK531812 (3): Gabon
G. austroafricanum KM507324: Africa
G. aff. austroafricanum MH571693: Africa
G. subamboinense KU569546: Brazil
G. weberianum GU726934 (2): India
G. cf. weberianum KC222319: China
Ganoderma sp. MK571161: China
G. weberianum FJ491970 (3): India
G. weberianum FJ491967 (6): India
G. weberianum FJ491972 (2): India
G. weberianum FJ491975 (2): India
G. weberianum FJ655481: India
G. weberianum FJ491986: India
G. weberianum FJ491988: India
G. weberianum FJ491969: India
G. carocalcareum EU089969 (2): Cameroon
G. weberianum JN105703 (3): Cameroon
Ganoderma sp. MK554768 (5): Cameroon, Gabon
Ganoderma sp. MK554774: Gabon
Ganoderma sp. MK554784: Cameroon
Ganoderma sp. MK603806: Gabon
G. sichuanense KX055552
G. weberianum MN622782
Ganoderma sp. MK554790: Taiwan
G. weberianum JQ520219 (2): Philippines
G. weberianum MK603804 (2): Philippines, Taiwan
G. weberianum MH864975: Taiwan
G. microsporum X78751/X78772: Taiwan
G. weberianum Z37064/Z37086: Taiwan
G. weberianum X78734/X78755: Philippines
G. sichuanense MN523258: China
G. tenue DQ424978 (2)
G. weberianum EU239393/4: Australia
G. tenue DQ424977 (3): China
G. sichuanense MN523251: China
G. sichuanense MN523256: China
G. weberianum JF915411: China
G. sichuanense MN523254: China
uncultured soil fungus UDB0757071: India
G. sichuanense MN523255: China
G. sichuanense MN523250: China
G. weberianum AY569451: Australia
G. sichuanense JQ781877: China
G. sichuanense JQ781878: China
G. sichuanense MN523253: China
Ganoderma sp. KJ654377
G. weberianum MK554771
G. tuberculosum AH008107: Argentina
G. subamboinense X78736/X78757: Argentina
G. subamboinense var. laevisporum MG654370: USA
G. sessiliforme AH008108: Brazil
G. mexicanum MK531820: Martinique
G. subamboinense var. laevisporum MT232638: Mexico
G. subamboinense DQ425005
G. subamboinense var. laevisporum JQ520205 (2): Argentina
G. subamboinense var. laevisporum MG654373: USA
G. mexicanum MK531815: Martinique
G. mexicanum MK531811 (2): Martinique, Mexico
G. mexicanum MK531818: Martinique
G. mexicanum MK531819: Martinique
G. subamboinense var. laevisporum MG654372 (2): USA
G. parvulum MK531132 (2): Cuba, French Guiana
G. weberianum KU214242: USA
G. parvulum MK554767 (3): Cuba
G. parvulum MK554769 (2): Cuba, French Guiana
Ganoderma sp. MK554792: Cuba
G. subamboinense MK531824/MK531822: Brazil
G. parvulum MK531814: French Guiana
G. weberianum JN637827
G. parvulum MK531821: Costa Rica
G. subamboinense var. laevisporum MG654371: USA
G. weberianum MT232635: Mexico
Ganoderma sp. LT726730: Cuba
G. weberianum MK531817: French Guiana
G. weberianum GU731560: French Guiana
G. stipitatum KC884264: Colombia
Ganoderma sp. LT726731: Cuba
G. resinaceum MH106883: China
Ganoderma sp. MK554791: China
G. resinaceum MN398315: China
Ganoderma sp. MH290273: India
Ganoderma sp. MH290277 (2): India
G. lucidum MG706253: Greece
G. resinaceum KF975890
G. resinaceum MN240471: Iran
G. lucidum KT343316: Iran
G. resinaceum MH571691: S. Africa
G. resinaceum MH571692: S. Africa
G. resinaceum MH796122: Egypt
G. resinaceum LN774970
Ganoderma sp. MK531816
G. resinaceum MK422153: Tunisia
G. resinaceum JX082328: France
Ganoderma sp. KT223758
G. resinaceum JN588590: Iran
G. pfeifferi MG706244 (3): Greece, Iran
G. lucidum KX371594 (2)
G. resinaceum MK554772: Netherlands
G. resinaceum AM269775: Italy
Polyporales sp. JQ312182
G. resinaceum MG706246 (19): Europe, China
G. resinaceum MN759653: Iraq
G. resinaceum MN448375
G. resinaceum KT343317: Iran
Ganoderma sp. KT223756
G. resinaceum MN240470: Iran
G. resinaceum FJ805250
G. resinaceum KT343310: Iran
G. resinaceum KT343303 (4): Iran
G. resinaceum AM269777: Italy
G. resinaceum MG706236: Greece
G. resinaceum AM269778 (4): Europe
G. resinaceum FJ491948 (4): India
G. resinaceum MK415309 (2): Slovakia
G. resinaceum KJ143915: France
G. resinaceum AM906062: Italy
G. resinaceum X78737/ X78758: Netherlands
G. resinaceum MK415241: Slovakia
G. lucidum MG706252: Greece
Polyporales sp. JQ312204
G. resinaceum MG706233 (12): Europe, India
G. resinaceum MK554786: France
G. resinaceum MK554766: Belgium
G. resinaceum JX082326 (2): France
G. resinaceum FJ491949: India
G. resinaceum AM906064 (6): Italy, India
G. resinaceum MG706235: Greece
G. resinaceum MG706238: Greece
G. resinaceum MG706241: Greece
G. resinaceum AM269776: Italy
G. resinaceum AY884177 (2): UK
G. resinaceum MG706242 (9): Europe, S. Korea
G. resinaceum MK415274 (3): Slovakia
G. resinaceum KF605627
G. resinaceum AM906065: Italy
G. resinaceum KF605628
G. resinaceum MK554775: Belgium
G. resinaceum JQ520204: Czech Republic
G. resinaceum Z37062/Z37085: UK
G. pfeifferi X78738/X78759: UK
G. resinaceum JN008875: Poland
G. resinaceum MG706243: Bulgaria
G. resinaceum MH854909: UK
G. resinaceum GU451246 (2): India
G. multipileum
G. philippii
G. hoehnelianum
G. austroafricanum
G. cf. weberianum
Ganoderma sp. A1
G. carocalcareum
G. weberianum
G. sichuanense
G. mexicanum
G. parvulum
Ganoderma sp. A2
G. resinaceum
Cluster A.2
CLADE A

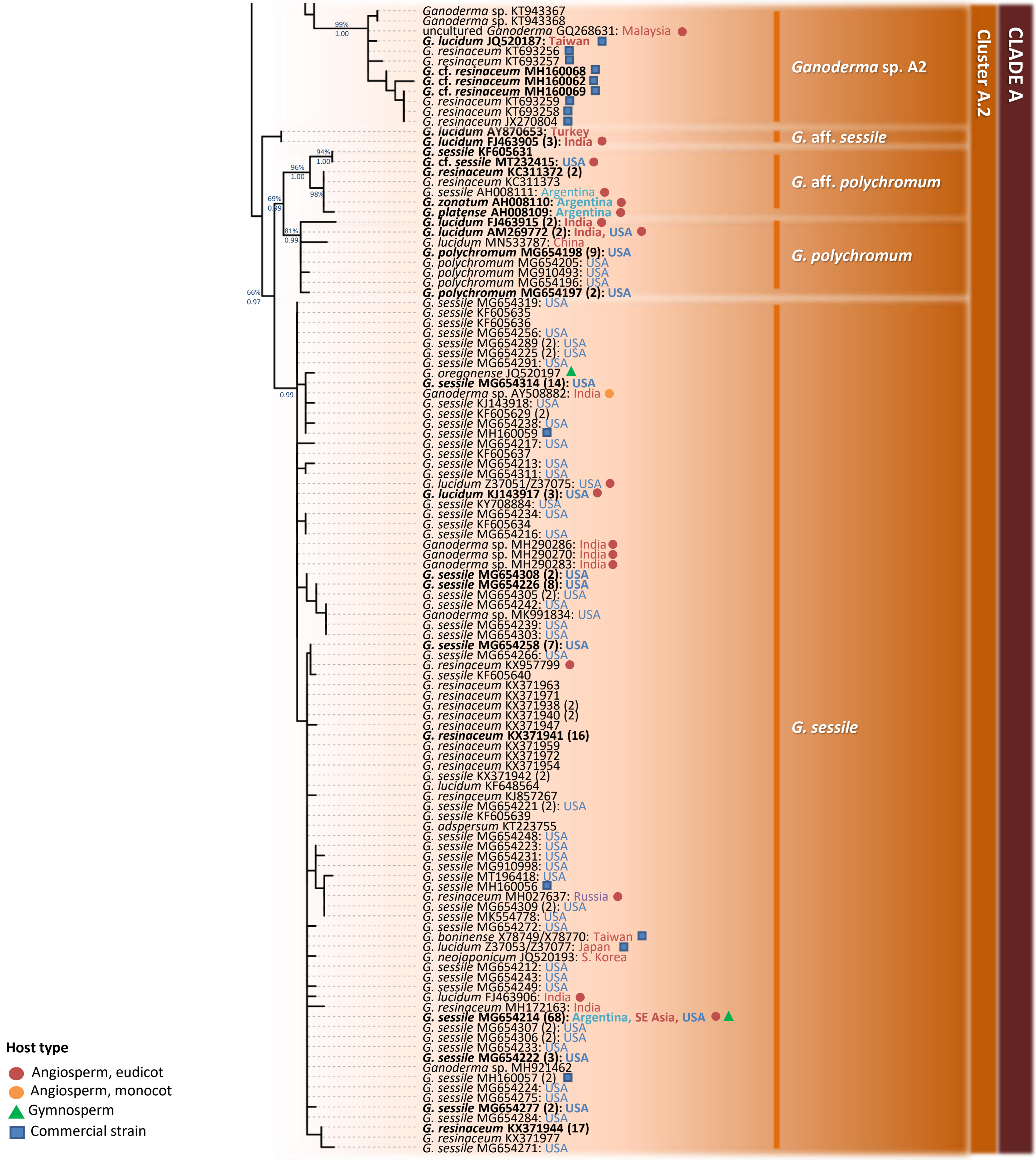

Supplementary Figure S2b. Phylogenetic reconstruction of the genus *Ganoderma* inferred from ML analysis based on ITS sequence data (pDS1b; Table 2) for Clade A, Cluster A.2. ML bootstrap values (BS) ≥ 65% and Bayesian posterior probabilities (BPP) ≥ 0.95 are shown. Sequences names on the left appear as initially labelled, and are followed by the respective GenBank/ENA/DDBJ or UNITE accession no., while the total number of identical entries corresponding to a particular sequence is placed in parentheses, followed by the type of host plant (legend for the colored shapes is found at the lower left side of tree) and geographic origin of the respective material (the latter appears in different font color depending on the continent of provenance; see also Tables 1 and S2). Species names on the right correspond to those inferred in this study evaluated in conjunction with literature data. Sequences included in the respective Figure (Fig. 4) of the manuscript appear in bold typeface, while underlined sequences are those originating from type material. Scale bar: 0.01 nucleotide substitutions per site.
