# Supplementary material for: A global meta-analysis of ITS rDNA sequences from material belonging to the genus Ganoderma (Basidiomycota, Polyporales) including new data from selected taxa
Source: MycoKeys. 2020 Dec 1;75:71–143. doi: 10.3897/mycokeys.75.59872 (PMC7723883; doi:10.3897/mycokeys.75.59872)
Supplement: Supplementary material 5 — Figure S2c [file mycokeys-75-071-s005.pdf]

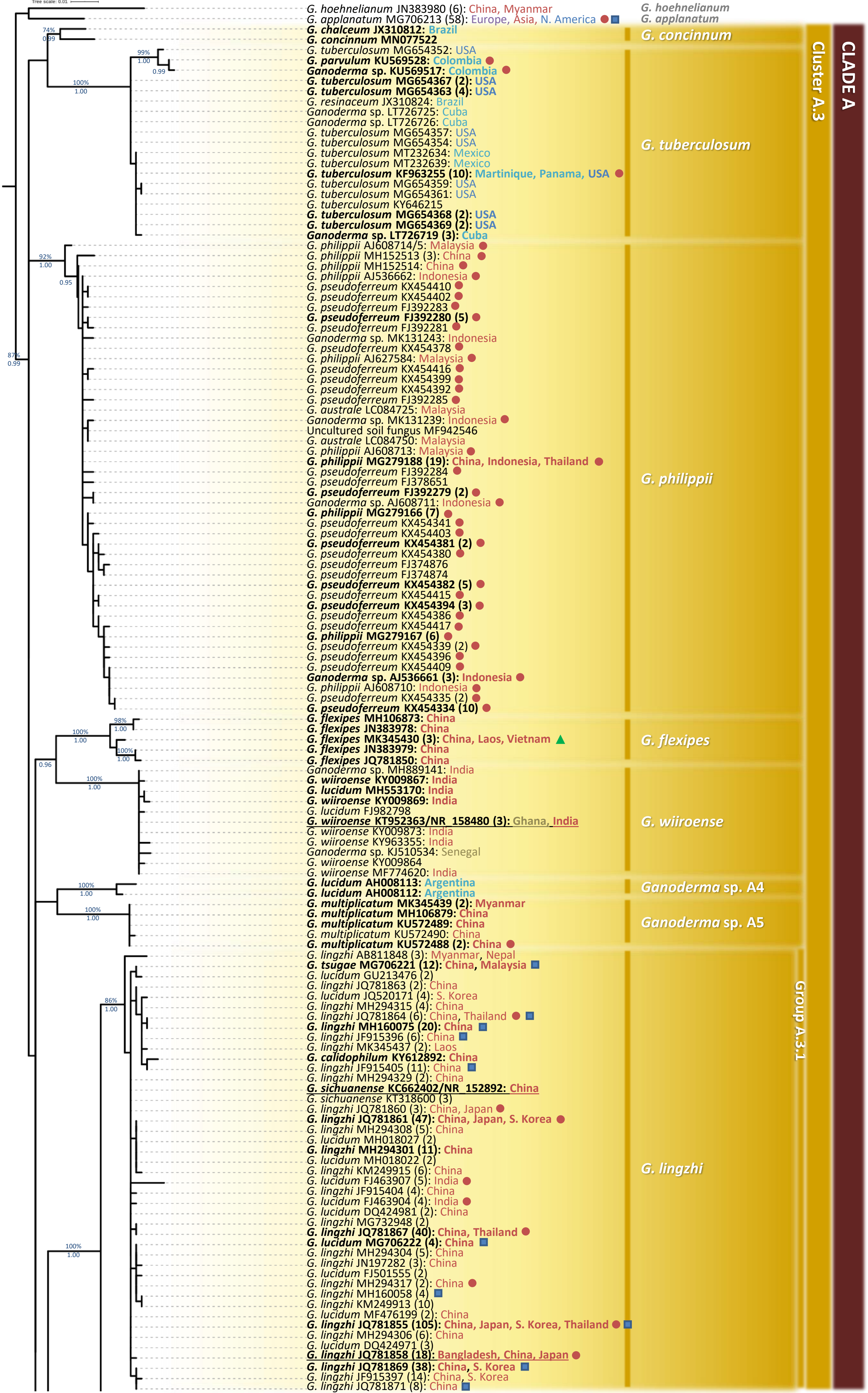
Tree scale: 0.01
G. hoehnelianum JN383980 (6): China, Myanmar
G. applanatum MG706213 (58): Europe, Asia, N. America
G. chalceum JX310812: Brazil
G. concinnum MN077522
G. tuberculosum MG654352: USA
G. parvulum KU569528: Colombia
Ganoderma sp. KU569517: Colombia
G. tuberculosum MG654367 (2): USA
G. tuberculosum MG654363 (4): USA
G. resinaceum JX310824: Brazil
Ganoderma sp. LT726725: Cuba
Ganoderma sp. LT726726: Cuba
G. tuberculosum MG654357: USA
G. tuberculosum MG654354: USA
G. tuberculosum MT232634: Mexico
G. tuberculosum MT232639: Mexico
G. tuberculosum KF963255 (10): Martinique, Panama, USA
G. tuberculosum MG654359: USA
G. tuberculosum MG654361: USA
G. tuberculosum KY646215
G. tuberculosum MG654368 (2): USA
G. tuberculosum MG654369 (2): USA
Ganoderma sp. LT726719 (3): Cuba
G. philippii AJ608714/5: Malaysia
G. philippii MH152513 (3): China
G. philippii MH152514: China
G. philippii AJ536662: Indonesia
G. pseudoferreum KX454410
G. pseudoferreum KX454402
G. pseudoferreum FJ392283
G. pseudoferreum FJ392280 (5)
G. pseudoferreum FJ392281
Ganoderma sp. MK131243: Indonesia
G. pseudoferreum KX454378
G. philippii AJ627584: Malaysia
G. pseudoferreum KX454416
G. pseudoferreum KX454399
G. pseudoferreum KX454392
G. pseudoferreum FJ392285
G. australe LC084725: Malaysia
Ganoderma sp. MK131239: Indonesia
Uncultured soil fungus MF942546
G. australe LC084750: Malaysia
G. philippii AJ608713: Malaysia
G. philippii MG279188 (19): China, Indonesia, Thailand
G. pseudoferreum FJ392284
G. pseudoferreum FJ378651
G. pseudoferreum FJ392279 (2)
Ganoderma sp. AJ608711: Indonesia
G. philippii MG279166 (7)
G. pseudoferreum KX454341
G. pseudoferreum KX454403
G. pseudoferreum KX454381 (2)
G. pseudoferreum KX454380
G. pseudoferreum FJ374876
G. pseudoferreum FJ374874
G. pseudoferreum KX454382 (5)
G. pseudoferreum KX454415
G. pseudoferreum KX454394 (3)
G. pseudoferreum KX454386
G. pseudoferreum KX454417
G. philippii MG279167 (6)
G. pseudoferreum KX454339 (2)
G. pseudoferreum KX454396
G. pseudoferreum KX454409
Ganoderma sp. AJ536661 (3): Indonesia
G. philippii AJ608710: Indonesia
G. pseudoferreum KX454335 (2)
G. pseudoferreum KX454334 (10)
G. flexipes MH106873: China
G. flexipes JN383978: China
G. flexipes MK345430 (3): China, Laos, Vietnam
G. flexipes JN383979: China
G. flexipes JQ781850: China
Ganoderma sp. MH889141: India
G. wiiroense KY009867: India
G. lucidum MH553170: India
G. wiiroense KY009869: India
G. lucidum FJ982798
G. wiiroense KT952363/NR_158480 (3): Ghana, India
G. wiiroense KY009873: India
G. wiiroense KY963355: India
Ganoderma sp. KJ510534: Senegal
G. wiiroense KY009864
G. wiiroense MF774620: India
G. lucidum AH008113: Argentina
G. lucidum AH008112: Argentina
G. multiplicatum MK345439 (2): Myanmar
G. multiplicatum MH106879: China
G. multiplicatum KU572489: China
G. multiplicatum KU572490: China
G. multiplicatum KU572488 (2): China
G. lingzhi AB811848 (3): Myanmar, Nepal
G. tsugae MG706221 (12): China, Malaysia
G. lucidum GU213476 (2)
G. lingzhi JQ781863 (2): China
G. lucidum JQ520171 (4): S. Korea
G. lingzhi MH294315 (4): China
G. lingzhi JQ781864 (6): China, Thailand
G. lingzhi MH160075 (20): China
G. lingzhi JF915396 (6): China
G. lingzhi MK345437 (2): Laos
G. calidophilum KY612892: China
G. lingzhi JF915405 (11): China
G. lingzhi MH294329 (2): China
G. sichuanense KC662402/NR_152892: China
G. sichuanense KT318600 (3)
G. lingzhi JQ781860 (3): China, Japan
G. lingzhi JQ781861 (47): China, Japan, S. Korea
G. lingzhi MH294308 (5): China
G. lucidum MH018027 (2)
G. lingzhi MH294301 (11): China
G. lucidum MH018022 (2)
G. lingzhi KM249915 (6): China
G. lucidum FJ463907 (5): India
G. lingzhi JF915404 (4): China
G. lucidum FJ463904 (4): India
G. lucidum DQ424981 (2): China
G. lingzhi MG732948 (2)
G. lingzhi JQ781867 (40): China, Thailand
G. lucidum MG706222 (4): China
G. lingzhi MH294304 (5): China
G. lingzhi JN197282 (3): China
G. lucidum FJ501555 (2)
G. lingzhi MH294317 (2): China
G. lingzhi MH160058 (4)
G. lingzhi KM249913 (10)
G. lucidum MF476199 (2): China
G. lingzhi JQ781855 (105): China, Japan, S. Korea, Thailand
G. lingzhi MH294306 (6): China
G. lucidum DQ424971 (3)
G. lingzhi JQ781858 (18): Bangladesh, China, Japan
G. lingzhi JQ781869 (38): China, S. Korea
G. lingzhi JF915397 (14): China, S. Korea
G. lingzhi JQ781871 (8): China
G. hoehnelianum
G. applanatum
G. concinnum
G. tuberculosum
G. philippii
G. flexipes
G. wiiroense
Ganoderma sp. A4
Ganoderma sp. A5
G. lingzhi
Group A.3.1
Cluster A.3
CLADE A
74% 0.99
99% 1.00
0.99
100% 1.00
92% 1.00
0.95
87% 0.99
98% 1.00
100% 1.00
100% 1.00
0.96
100% 1.00
100% 1.00
100% 1.00
86% 1.00
100% 1.00

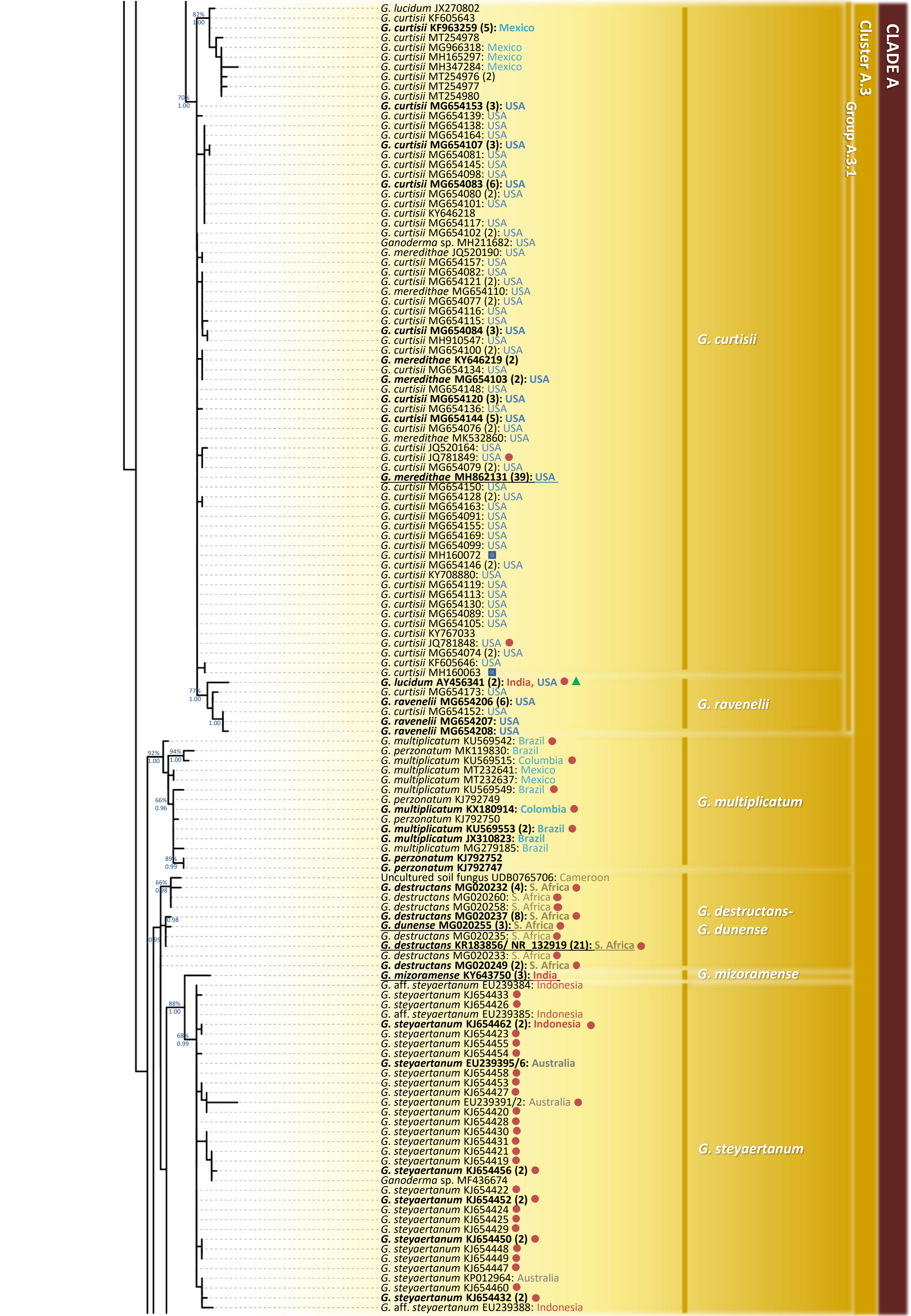
CLADE A
Cluster A.3
Group A.3.1
G. lucidum JX270802
G. curtisii KF605643
G. curtisii KF963259 (5): Mexico
G. curtisii MT254978
G. curtisii MG966318: Mexico
G. curtisii MH165297: Mexico
G. curtisii MH347284: Mexico
G. curtisii MT254976 (2)
G. curtisii MT254977
G. curtisii MT254980
G. curtisii MG654153 (3): USA
G. curtisii MG654139: USA
G. curtisii MG654138: USA
G. curtisii MG654164: USA
G. curtisii MG654107 (3): USA
G. curtisii MG654081: USA
G. curtisii MG654145: USA
G. curtisii MG654098: USA
G. curtisii MG654083 (6): USA
G. curtisii MG654080 (2): USA
G. curtisii MG654101: USA
G. curtisii KY646218
G. curtisii MG654117: USA
G. curtisii MG654102 (2): USA
Ganoderma sp. MH211682: USA
G. meredithae JQ520190: USA
G. curtisii MG654157: USA
G. curtisii MG654082: USA
G. curtisii MG654121 (2): USA
G. meredithae MG654110: USA
G. curtisii MG654077 (2): USA
G. curtisii MG654116: USA
G. curtisii MG654115: USA
G. curtisii MG654084 (3): USA
G. curtisii MH910547: USA
G. curtisii MG654100 (2): USA
G. meredithae KY646219 (2)
G. curtisii MG654134: USA
G. meredithae MG654103 (2): USA
G. curtisii MG654148: USA
G. curtisii MG654120 (3): USA
G. curtisii MG654136: USA
G. curtisii MG654144 (5): USA
G. curtisii MG654076 (2): USA
G. meredithae MK532860: USA
G. curtisii JQ520164: USA
G. curtisii JQ781849: USA
G. curtisii MG654079 (2): USA
G. meredithae MH862131 (39): USA
G. curtisii MG654150: USA
G. curtisii MG654128 (2): USA
G. curtisii MG654163: USA
G. curtisii MG654091: USA
G. curtisii MG654155: USA
G. curtisii MG654169: USA
G. curtisii MG654099: USA
G. curtisii MH160072
G. curtisii MG654146 (2): USA
G. curtisii KY708880: USA
G. curtisii MG654119: USA
G. curtisii MG654113: USA
G. curtisii MG654130: USA
G. curtisii MG654089: USA
G. curtisii MG654105: USA
G. curtisii KY767033
G. curtisii JQ781848: USA
G. curtisii MG654074 (2): USA
G. curtisii KF605646: USA
G. curtisii MH160063
G. curtisii
G. lucidum AY456341 (2): India, USA
G. curtisii MG654173: USA
G. ravenelii MG654206 (6): USA
G. curtisii MG654152: USA
G. ravenelii MG654207: USA
G. ravenelii MG654208: USA
G. ravenelii
G. multiplicatum KU569542: Brazil
G. perzonatum MK119830: Brazil
G. multiplicatum KU569515: Columbia
G. multiplicatum MT232641: Mexico
G. multiplicatum MT232637: Mexico
G. multiplicatum KU569549: Brazil
G. perzonatum KJ792749
G. multiplicatum KX180914: Colombia
G. perzonatum KJ792750
G. multiplicatum KU569553 (2): Brazil
G. multiplicatum JX310823: Brazil
G. multiplicatum MG279185: Brazil
G. perzonatum KJ792752
G. perzonatum KJ792747
G. multiplicatum
Uncultured soil fungus UDB0765706: Cameroon
G. destructans MG020232 (4): S. Africa
G. destructans MG020260: S. Africa
G. destructans MG020258: S. Africa
G. destructans MG020237 (8): S. Africa
G. dunense MG020255 (3): S. Africa
G. destructans MG020235: S. Africa
G. destructans KR183856/ NR_132919 (21): S. Africa
G. destructans MG020233: S. Africa
G. destructans MG020249 (2): S. Africa
G. destructans-
G. dunense
G. mizoramense KY643750 (3): India
G. mizoramense
G. aff. steyaertanum EU239384: Indonesia
G. steyaertanum KJ654433
G. steyaertanum KJ654426
G. aff. steyaertanum EU239385: Indonesia
G. steyaertanum KJ654462 (2): Indonesia
G. steyaertanum KJ654423
G. steyaertanum KJ654455
G. steyaertanum KJ654454
G. steyaertanum EU239395/6: Australia
G. steyaertanum KJ654458
G. steyaertanum KJ654453
G. steyaertanum KJ654427
G. steyaertanum EU239391/2: Australia
G. steyaertanum KJ654420
G. steyaertanum KJ654428
G. steyaertanum KJ654430
G. steyaertanum KJ654431
G. steyaertanum KJ654421
G. steyaertanum KJ654419
G. steyaertanum KJ654456 (2)
Ganoderma sp. MF436674
G. steyaertanum KJ654422
G. steyaertanum KJ654452 (2)
G. steyaertanum KJ654424
G. steyaertanum KJ654425
G. steyaertanum KJ654429
G. steyaertanum KJ654450 (2)
G. steyaertanum KJ654448
G. steyaertanum KJ654449
G. steyaertanum KJ654447
G. steyaertanum KP012964: Australia
G. steyaertanum KJ654460
G. steyaertanum KJ654432 (2)
G. aff. steyaertanum EU239388: Indonesia
G. steyaertanum

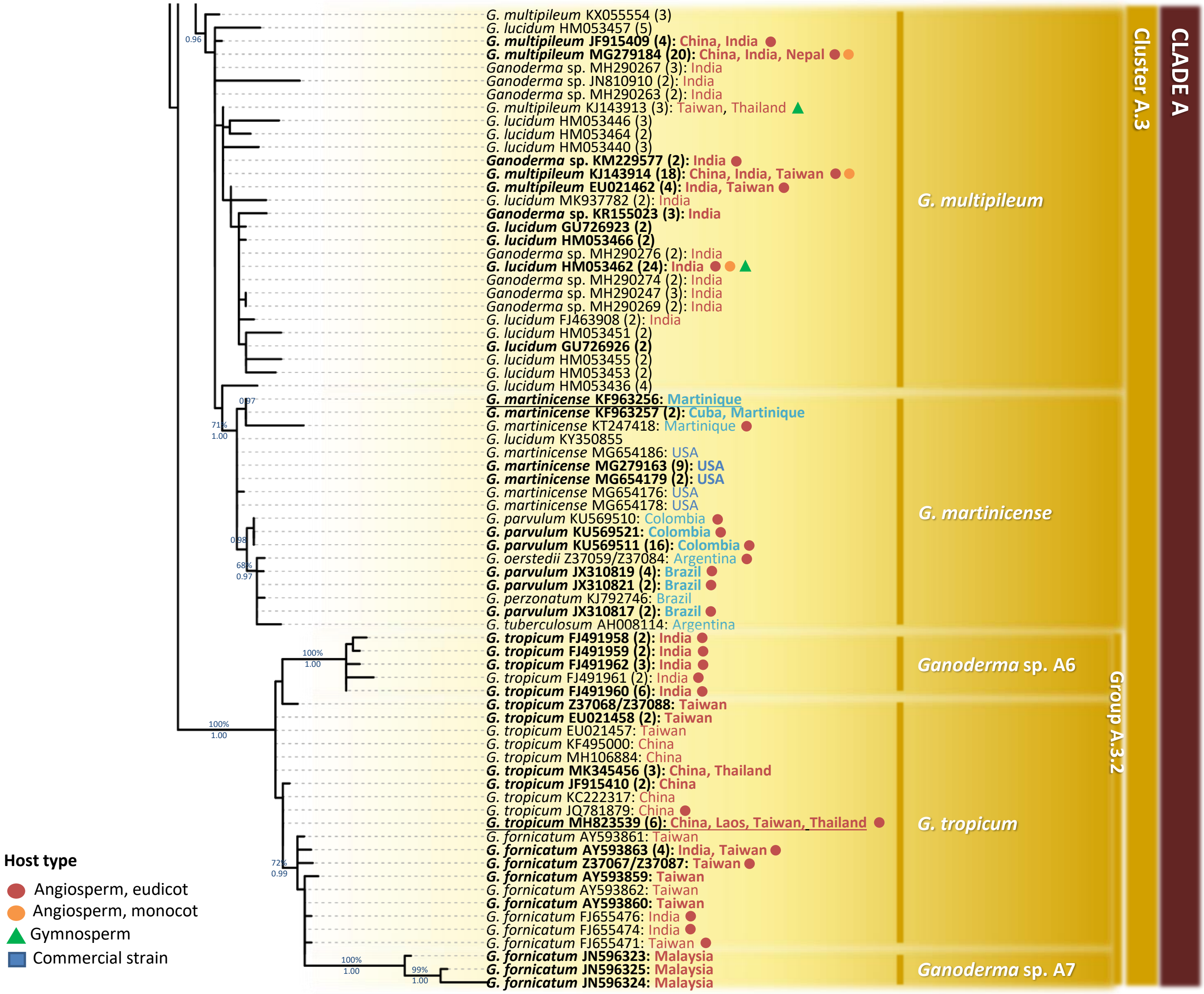

Supplementary Figure S2c. Phylogenetic reconstruction of the genus *Ganoderma* inferred from ML analysis based on ITS sequence data (pDS1c; Table 2) for Clade A, Cluster A.3. ML bootstrap values (BS) ≥ 65% and Bayesian posterior probabilities (BPP) ≥ 0.95 are shown. Sequences names on the left appear as initially labelled, and are followed by the respective GenBank/ENA/DDBJ or UNITE accession no., while the total number of identical entries corresponding to a particular sequence is placed in parentheses, followed by the type of host plant (legend for the colored shapes is found at the lower left side of tree) and geographic origin of the respective material (the latter appears in different font color depending on the continent of provenance; see also Tables 1 and S2). Species names on the right correspond to those inferred in this study evaluated in conjunction with literature data. Sequences included in the respective Figure (Fig. 5) of the manuscript appear in bold typeface, while underlined sequences are those originating from type material. Scale bar: 0.01 nucleotide substitutions per site.
