# Supplementary material for: A global meta-analysis of ITS rDNA sequences from material belonging to the genus Ganoderma (Basidiomycota, Polyporales) including new data from selected taxa
Source: MycoKeys. 2020 Dec 1;75:71–143. doi: 10.3897/mycokeys.75.59872 (PMC7723883; doi:10.3897/mycokeys.75.59872)
Supplement: Supplementary material 6 — Figure S2d [file mycokeys-75-071-s006.pdf]

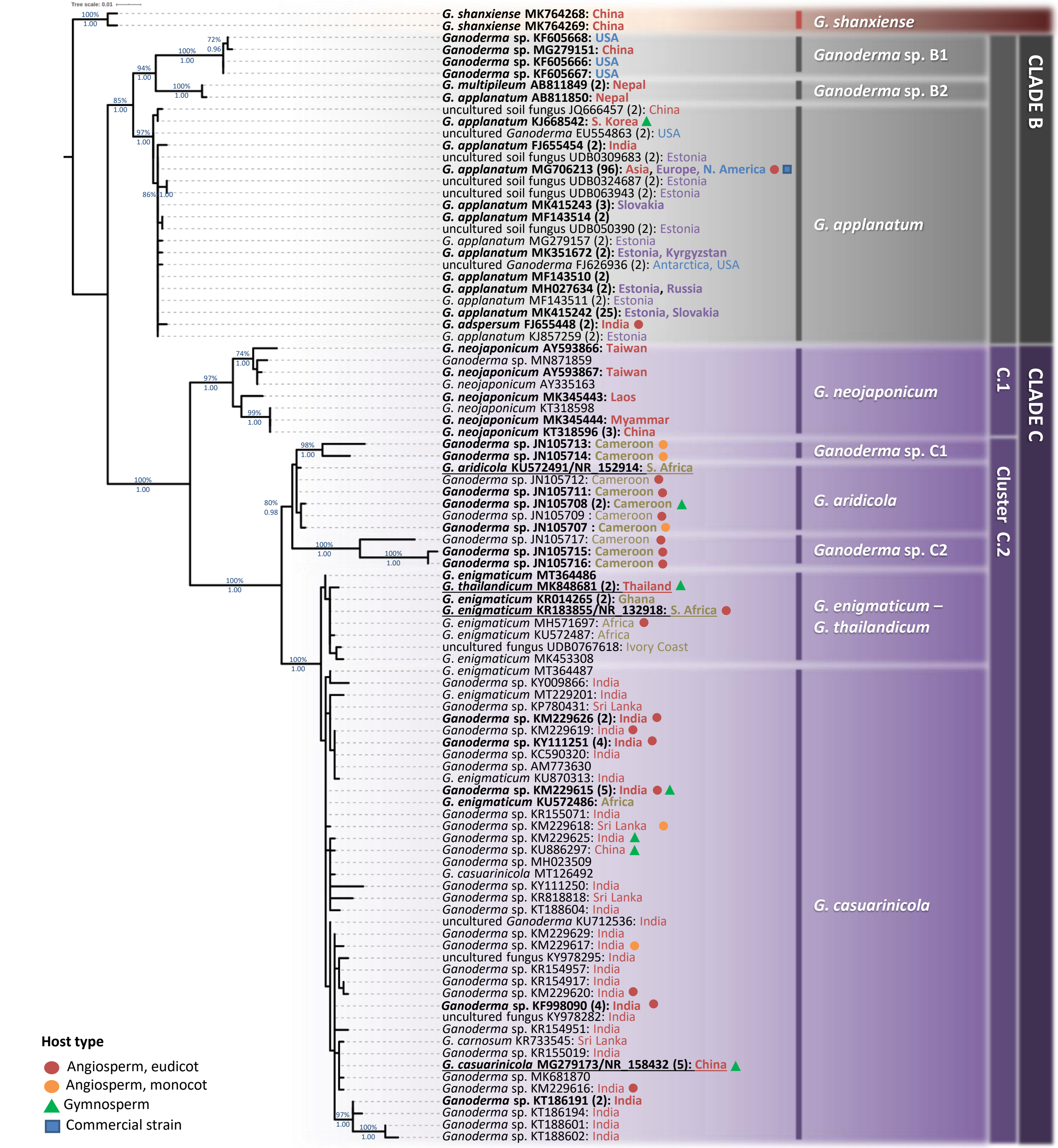

Supplementary Figure S2d. Phylogenetic reconstruction of the genus *Ganoderma* inferred from ML analysis based on ITS sequence data (pDS2 & pDS3; Table 2) for Clades B & C. ML bootstrap values (BS) ≥ 65% and Bayesian posterior probabilities (BPP) ≥ 0.95 are shown. Sequences names on the left appear as initially labelled, and are followed by the respective GenBank/ENA/DDBJ or UNITE accession no., while the total number of identical entries corresponding to a particular sequence is placed in parentheses, followed by the type of host plant (legend for the colored shapes is found at the lower left side of tree) and geographic origin of the respective material (the latter appears in different font color depending on the continent of provenance; see also Tables 1 and S2). Species names on the right correspond to those inferred in this study evaluated in conjunction with literature data. Sequences included in the respective Figure (Fig. 6) of the manuscript appear in bold typeface, while underlined sequences are those originating from type material. Scale bar: 0.01 nucleotide substitutions per site.
