# Supplementary material for: A global meta-analysis of ITS rDNA sequences from material belonging to the genus Ganoderma (Basidiomycota, Polyporales) including new data from selected taxa
Source: MycoKeys. 2020 Dec 1;75:71–143. doi: 10.3897/mycokeys.75.59872 (PMC7723883; doi:10.3897/mycokeys.75.59872)
Supplement: Supplementary material 7 — Figure S2e [file mycokeys-75-071-s007.pdf]

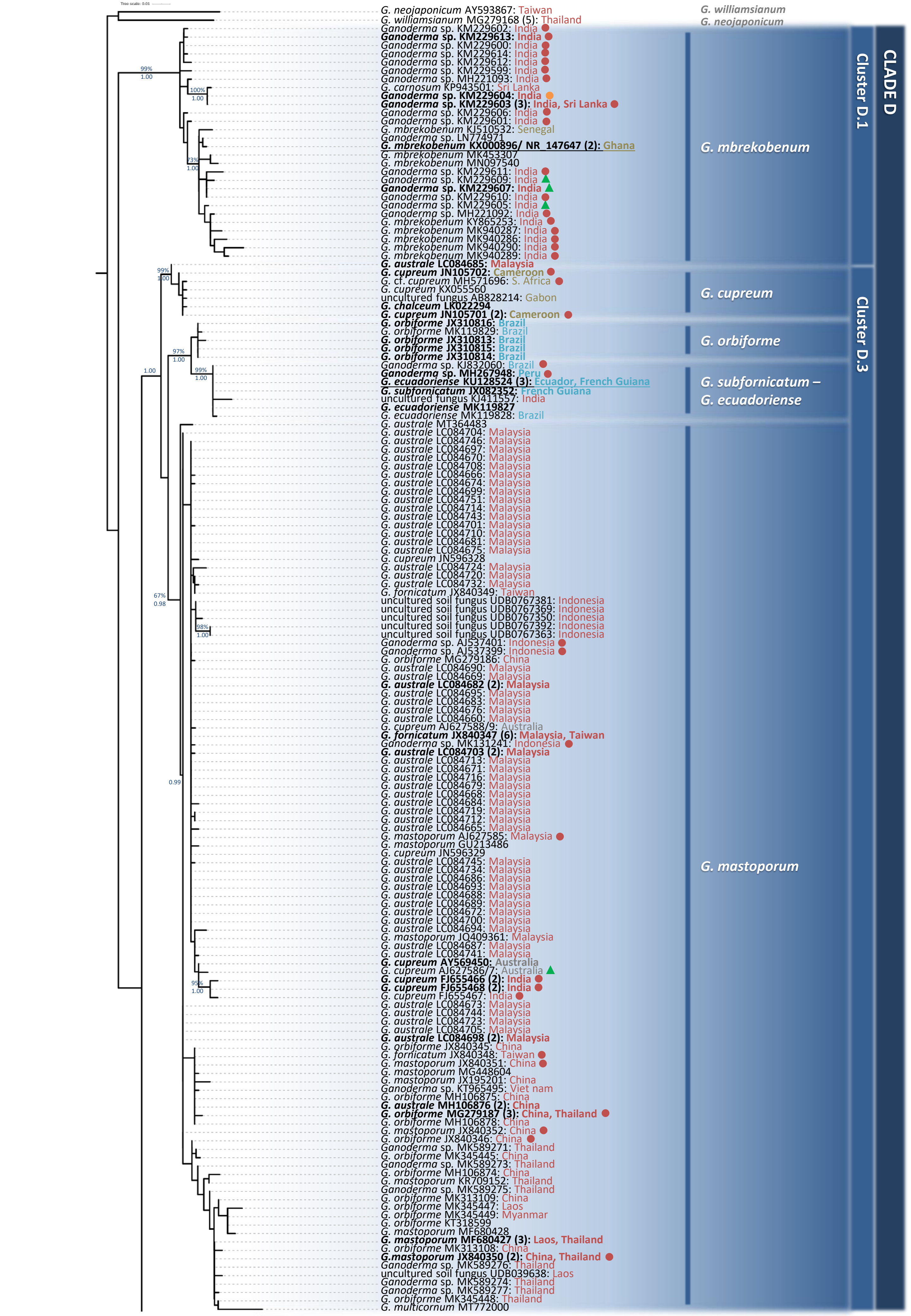
Tree scale: 0.01
G. neojaponicum AY593867: Taiwan
G. williamsianum MG279168 (5): Thailand
G. williamsianum
G. neojaponicum
Ganoderma sp. KM229602: India
Ganoderma sp. KM229613: India
Ganoderma sp. KM229600: India
Ganoderma sp. KM229614: India
Ganoderma sp. KM229612: India
Ganoderma sp. KM229599: India
Ganoderma sp. MH221093: India
G. carnosum KP943501: Sri Lanka
Ganoderma sp. KM229604: India
Ganoderma sp. KM229603 (3): India, Sri Lanka
Ganoderma sp. KM229606: India
Ganoderma sp. KM229601: India
G. mbrekobenum KJ510532: Senegal
Ganoderma sp. LN774971
G. mbrekobenum KX000896/ NR_147647 (2): Ghana
G. mbrekobenum MK453307
G. mbrekobenum MN097540
Ganoderma sp. KM229611: India
Ganoderma sp. KM229609: India
Ganoderma sp. KM229607: India
Ganoderma sp. KM229610: India
Ganoderma sp. KM229605: India
Ganoderma sp. MH221092: India
G. mbrekobenum KY865253: India
G. mbrekobenum MK940287: India
G. mbrekobenum MK940286: India
G. mbrekobenum MK940290: India
G. mbrekobenum MK940289: India
G. australe LC084685: Malaysia
G. cupreum JN105702: Cameroon
G. cf. cupreum MH571696: S. Africa
G. cupreum KX055560
uncultured fungus AB828214: Gabon
G. chalceum LK022294
G. cupreum JN105701 (2): Cameroon
G. orbiforme JX310816: Brazil
G. orbiforme MK119829: Brazil
G. orbiforme JX310813: Brazil
G. orbiforme JX310815: Brazil
G. orbiforme JX310814: Brazil
Ganoderma sp. KJ832060: Brazil
Ganoderma sp. MH267948: Peru
G. ecuadoriense KU128524 (3): Ecuador, French Guiana
G. subfornicatum JX082352: French Guiana
uncultured fungus KJ411557: India
G. ecuadoriense MK119827
G. ecuadoriense MK119828: Brazil
G. australe MT364483
G. australe LC084704: Malaysia
G. australe LC084746: Malaysia
G. australe LC084697: Malaysia
G. australe LC084670: Malaysia
G. australe LC084708: Malaysia
G. australe LC084666: Malaysia
G. australe LC084674: Malaysia
G. australe LC084699: Malaysia
G. australe LC084751: Malaysia
G. australe LC084714: Malaysia
G. australe LC084743: Malaysia
G. australe LC084701: Malaysia
G. australe LC084710: Malaysia
G. australe LC084681: Malaysia
G. australe LC084675: Malaysia
G. cupreum JN596328
G. australe LC084724: Malaysia
G. australe LC084720: Malaysia
G. australe LC084732: Malaysia
G. fornicatum JX840349: Taiwan
uncultured soil fungus UDB0767381: Indonesia
uncultured soil fungus UDB0767369: Indonesia
uncultured soil fungus UDB0767350: Indonesia
uncultured soil fungus UDB0767392: Indonesia
uncultured soil fungus UDB0767363: Indonesia
Ganoderma sp. AJ537401: Indonesia
Ganoderma sp. AJ537399: Indonesia
G. orbiforme MG279186: China
G. australe LC084690: Malaysia
G. australe LC084669: Malaysia
G. australe LC084682 (2): Malaysia
G. australe LC084695: Malaysia
G. australe LC084683: Malaysia
G. australe LC084676: Malaysia
G. australe LC084660: Malaysia
G. cupreum AJ627588/9: Australia
G. fornicatum JX840347 (6): Malaysia, Taiwan
Ganoderma sp. MK131241: Indonesia
G. australe LC084703 (2): Malaysia
G. australe LC084713: Malaysia
G. australe LC084671: Malaysia
G. australe LC084716: Malaysia
G. australe LC084679: Malaysia
G. australe LC084668: Malaysia
G. australe LC084684: Malaysia
G. australe LC084719: Malaysia
G. australe LC084712: Malaysia
G. australe LC084665: Malaysia
G. mastoporum AJ627585: Malaysia
G. mastoporum GU213486
G. cupreum JN596329
G. australe LC084745: Malaysia
G. australe LC084734: Malaysia
G. australe LC084686: Malaysia
G. australe LC084693: Malaysia
G. australe LC084688: Malaysia
G. australe LC084689: Malaysia
G. australe LC084672: Malaysia
G. australe LC084700: Malaysia
G. australe LC084694: Malaysia
G. mastoporum JQ409361: Malaysia
G. australe LC084687: Malaysia
G. australe LC084741: Malaysia
G. cupreum AY569450: Australia
G. cupreum AJ627586/7: Australia
G. cupreum FJ655466 (2): India
G. cupreum FJ655468 (2): India
G. cupreum FJ655467: India
G. australe LC084673: Malaysia
G. australe LC084744: Malaysia
G. australe LC084723: Malaysia
G. australe LC084705: Malaysia
G. australe LC084698 (2): Malaysia
G. orbiforme JX840345: China
G. fornicatum JX840348: Taiwan
G. mastoporum JX840351: China
G. mastoporum MG448604
G. mastoporum JX195201: China
Ganoderma sp. KT965495: Viet nam
G. orbiforme MH106875: China
G. australe MH106876 (2): China
G. orbiforme MG279187 (3): China, Thailand
G. orbiforme MH106878: China
G. mastoporum JX840352: China
G. orbiforme JX840346: China
Ganoderma sp. MK589271: Thailand
G. orbiforme MK345445: China
Ganoderma sp. MK589273: Thailand
G. orbiforme MH106874: China
G. mastoporum KR709152: Thailand
Ganoderma sp. MK589275: Thailand
G. orbiforme MK313109: China
G. orbiforme MK345447: Laos
G. orbiforme MK345449: Myanmar
G. orbiforme KT318599
G. mastoporum MF680428
G. mastoporum MF680427 (3): Laos, Thailand
G. orbiforme MK313108: China
G.mastoporum JX840350 (2): China, Thailand
Ganoderma sp. MK589276: Thailand
uncultured soil fungus UDB039638: Laos
Ganoderma sp. MK589274: Thailand
Ganoderma sp. MK589277: Thailand
G. orbiforme MK345448: Thailand
G. multicornum MT772000
99% 1.00
100% 1.00
73% 1.00
99% 1.00
97% 1.00
1.00
99% 1.00
67% 0.98
98% 1.00
0.99
95% 1.00
G. mbrekobenum
G. cupreum
G. orbiforme
G. subfornicatum – G. ecuadoriense
G. mastoporum
Cluster D.1
Cluster D.3
CLADE D

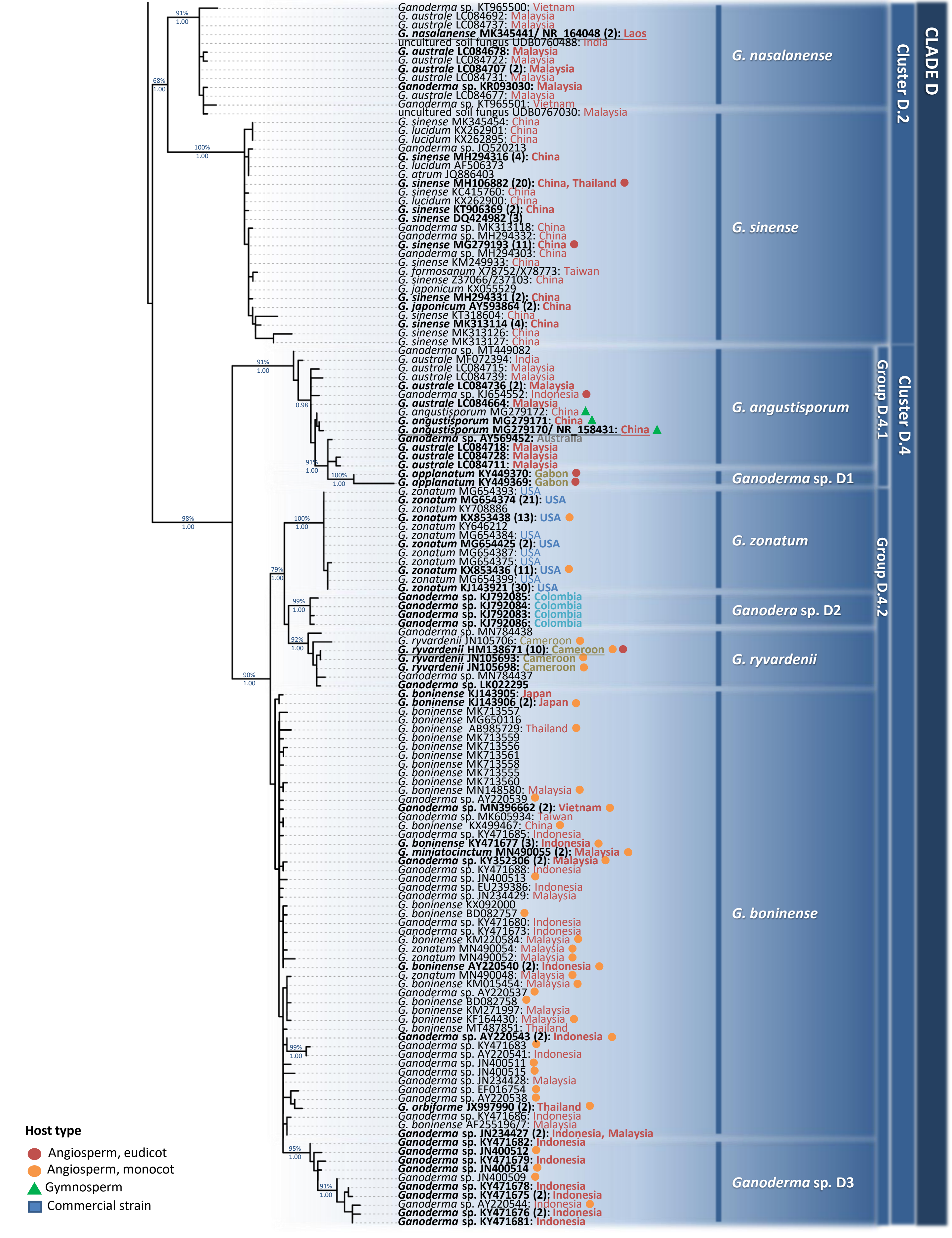

Supplementary Figure S2e. Phylogenetic reconstruction of the genus *Ganoderma* inferred from ML analysis based on ITS sequence data (pDS4; Table 2) for Clade D. ML bootstrap values (BS) ≥ 65% and Bayesian posterior probabilities (BPP) ≥ 0.95 are shown. Sequences names on the left appear as initially labelled, and are followed by the respective GenBank/ENA/DDBJ or UNITE accession no., while the total number of identical entries corresponding to a particular sequence is placed in parentheses, followed by the type of host plant (legend for the colored shapes is found at the lower left side of tree) and geographic origin of the respective material (the latter appears in different font color depending on the continent of provenance; see also Tables 1 and S2). Species names on the right correspond to those inferred in this study evaluated in conjunction with literature data. Sequences included in the respective Figure (Fig. 6) of the manuscript appear in bold typeface, while underlined sequences are those originating from type material. Scale bar: 0.01 nucleotide substitutions per site.
