# Supplementary material for: A global meta-analysis of ITS rDNA sequences from material belonging to the genus Ganoderma (Basidiomycota, Polyporales) including new data from selected taxa
Source: MycoKeys. 2020 Dec 1;75:71–143. doi: 10.3897/mycokeys.75.59872 (PMC7723883; doi:10.3897/mycokeys.75.59872)
Supplement: Supplementary material 8 — Figure S2f [file mycokeys-75-071-s008.pdf]

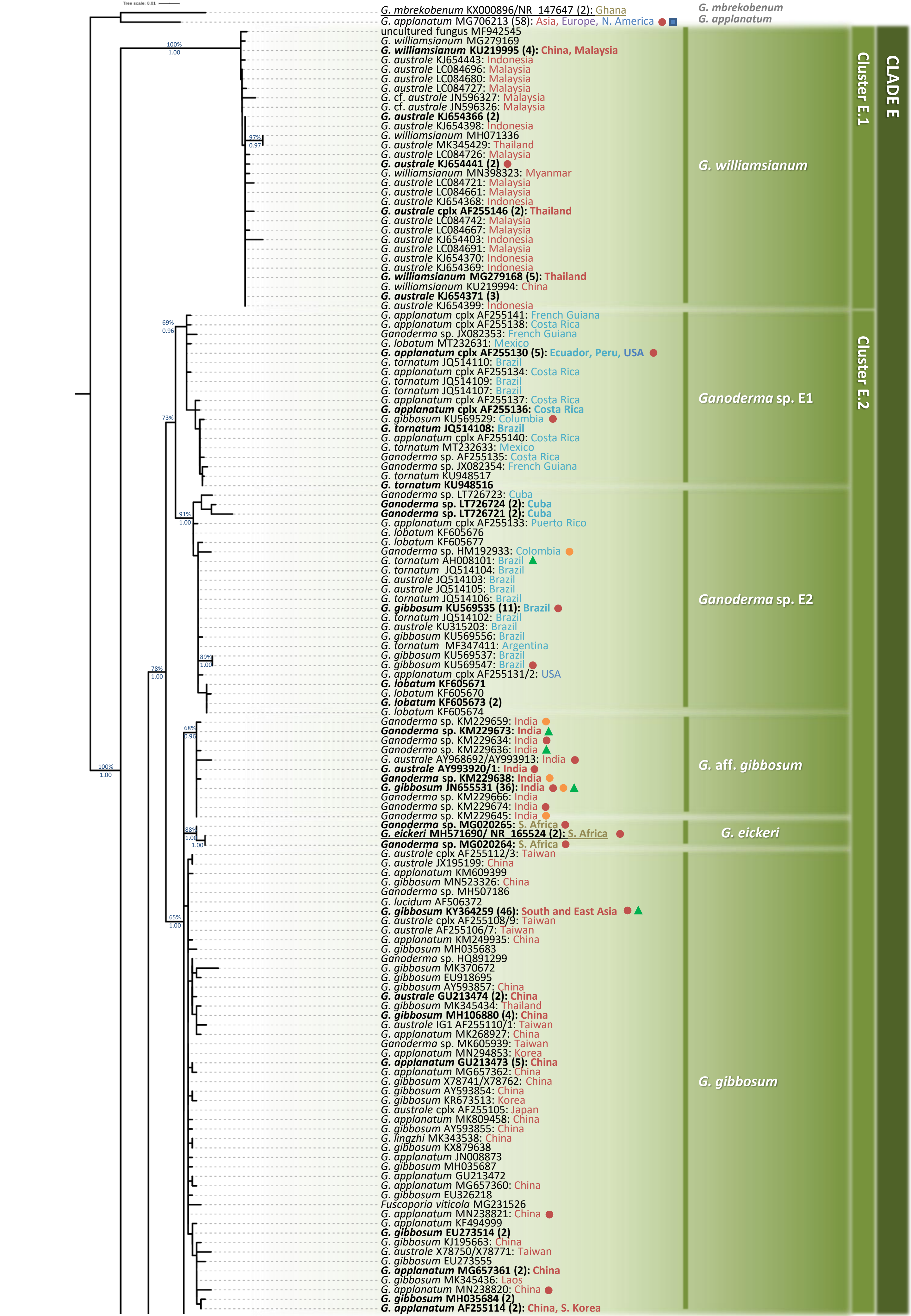
Tree scale: 0.01
G. mbrekobenum KX000896/NR_147647 (2): Ghana
G. applanatum MG706213 (58): Asia, Europe, N. America
uncultured fungus MF942545
G. williamsianum MG279169
G. williamsianum KU219995 (4): China, Malaysia
G. australe KJ654443: Indonesia
G. australe LC084696: Malaysia
G. australe LC084680: Malaysia
G. australe LC084727: Malaysia
G. cf. australe JN596327: Malaysia
G. cf. australe JN596326: Malaysia
G. australe KJ654366 (2)
G. australe KJ654398: Indonesia
G. williamsianum MH071336
G. australe MK345429: Thailand
G. australe LC084726: Malaysia
G. australe KJ654441 (2)
G. williamsianum MN398323: Myanmar
G. australe LC084721: Malaysia
G. australe LC084661: Malaysia
G. australe KJ654368: Indonesia
G. australe cplx AF255146 (2): Thailand
G. australe LC084742: Malaysia
G. australe LC084667: Malaysia
G. australe KJ654403: Indonesia
G. australe LC084691: Malaysia
G. australe KJ654370: Indonesia
G. australe KJ654369: Indonesia
G. williamsianum MG279168 (5): Thailand
G. williamsianum KU219994: China
G. australe KJ654371 (3)
G. australe KJ654399: Indonesia
G. applanatum cplx AF255141: French Guiana
G. applanatum cplx AF255138: Costa Rica
Ganoderma sp. JX082353: French Guiana
G. lobatum MT232631: Mexico
G. applanatum cplx AF255130 (5): Ecuador, Peru, USA
G. tornatum JQ514110: Brazil
G. applanatum cplx AF255134: Costa Rica
G. tornatum JQ514109: Brazil
G. tornatum JQ514107: Brazil
G. applanatum cplx AF255137: Costa Rica
G. applanatum cplx AF255136: Costa Rica
G. gibbosum KU569529: Columbia
G. tornatum JQ514108: Brazil
G. applanatum cplx AF255140: Costa Rica
G. tornatum MT232633: Mexico
Ganoderma sp. AF255135: Costa Rica
Ganoderma sp. JX082354: French Guiana
G. tornatum KU948517
G. tornatum KU948516
Ganoderma sp. LT726723: Cuba
Ganoderma sp. LT726724 (2): Cuba
Ganoderma sp. LT726721 (2): Cuba
G. applanatum cplx AF255133: Puerto Rico
G. lobatum KF605676
G. lobatum KF605677
Ganoderma sp. HM192933: Colombia
G. tornatum AH008101: Brazil
G. tornatum JQ514104: Brazil
G. australe JQ514103: Brazil
G. australe JQ514105: Brazil
G. tornatum JQ514106: Brazil
G. gibbosum KU569535 (11): Brazil
G. tornatum JQ514102: Brazil
G. australe KU315203: Brazil
G. gibbosum KU569556: Brazil
G. tornatum MF347411: Argentina
G. gibbosum KU569537: Brazil
G. gibbosum KU569547: Brazil
G. applanatum cplx AF255131/2: USA
G. lobatum KF605671
G. lobatum KF605670
G. lobatum KF605673 (2)
G. lobatum KF605674
Ganoderma sp. KM229659: India
Ganoderma sp. KM229673: India
Ganoderma sp. KM229634: India
Ganoderma sp. KM229636: India
G. australe AY968692/AY993913: India
G. australe AY993920/1: India
Ganoderma sp. KM229638: India
G. gibbosum JN655531 (36): India
Ganoderma sp. KM229666: India
Ganoderma sp. KM229674: India
Ganoderma sp. KM229645: India
Ganoderma sp. MG020265: S. Africa
G. eickeri MH571690/ NR_165524 (2): S. Africa
Ganoderma sp. MG020264: S. Africa
G. australe cplx AF255112/3: Taiwan
G. australe JX195199: China
G. applanatum KM609399
G. gibbosum MN523326: China
Ganoderma sp. MH507186
G. lucidum AF506372
G. gibbosum KY364259 (46): South and East Asia
G. australe cplx AF255108/9: Taiwan
G. australe AF255106/7: Taiwan
G. applanatum KM249935: China
G. gibbosum MH035683
Ganoderma sp. HQ891299
G. gibbosum MK370672
G. gibbosum EU918695
G. gibbosum AY593857: China
G. australe GU213474 (2): China
G. gibbosum MK345434: Thailand
G. gibbosum MH106880 (4): China
G. australe IG1 AF255110/1: Taiwan
G. applanatum MK268927: China
Ganoderma sp. MK605939: Taiwan
G. applanatum MN294853: Korea
G. applanatum GU213473 (5): China
G. applanatum MG657362: China
G. gibbosum X78741/X78762: China
G. gibbosum AY593854: China
G. gibbosum KR673513: Korea
G. australe cplx AF255105: Japan
G. applanatum MK809458: China
G. gibbosum AY593855: China
G. lingzhi MK343538: China
G. gibbosum KX879638
G. applanatum JN008873
G. gibbosum MH035687
G. applanatum GU213472
G. applanatum MG657360: China
G. gibbosum EU326218
Fuscoporia viticola MG231526
G. applanatum MN238821: China
G. applanatum KF494999
G. gibbosum EU273514 (2)
G. gibbosum KJ195663: China
G. australe X78750/X78771: Taiwan
G. gibbosum EU273555
G. applanatum MG657361 (2): China
G. gibbosum MK345436: Laos
G. applanatum MN238820: China
G. gibbosum MH035684 (2)
G. applanatum AF255114 (2): China, S. Korea
G. mbrekobenum
G. applanatum
G. williamsianum
Ganoderma sp. E1
Ganoderma sp. E2
G. aff. gibbosum
G. eickeri
G. gibbosum
Cluster E.1
Cluster E.2
CLADE E
100%
1.00
97%
0.97
69%
0.96
73%
91%
1.00
89%
1.00
78%
1.00
68%
0.96
88%
1.00
1.00
65%
1.00
100%
1.00

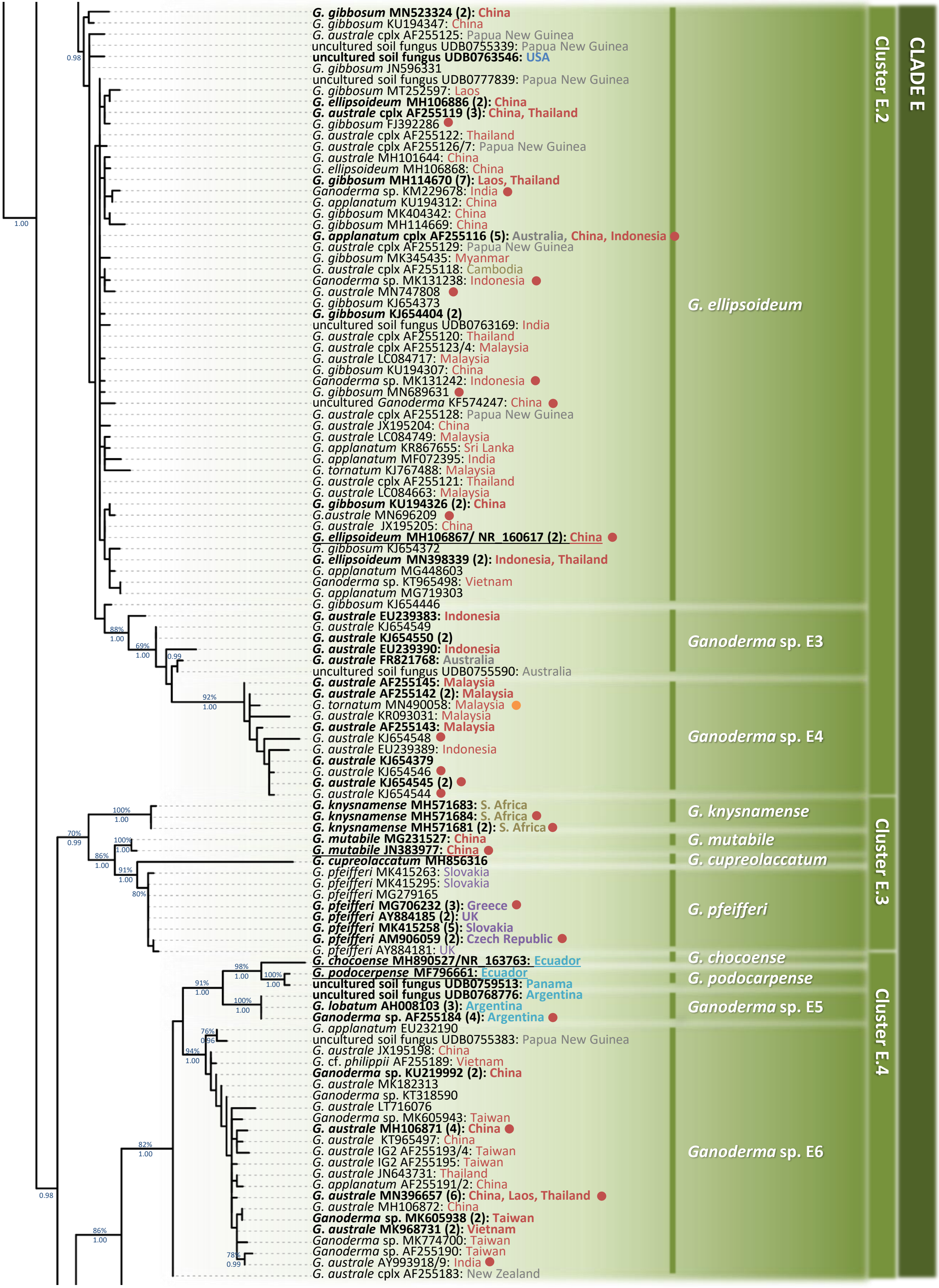

G. gibbosum MN523324 (2): China
G. gibbosum KU194347: China
G. australe cplx AF255125: Papua New Guinea
uncultured soil fungus UDB0755339: Papua New Guinea
uncultured soil fungus UDB0763546: USA
G. gibbosum JN596331
uncultured soil fungus UDB0777839: Papua New Guinea
G. gibbosum MT252597: Laos
G. ellipsoideum MH106886 (2): China
G. australe cplx AF255119 (3): China, Thailand
G. gibbosum FJ392286
G. australe cplx AF255122: Thailand
G. australe cplx AF255126/7: Papua New Guinea
G. australe MH101644: China
G. ellipsoideum MH106868: China
G. gibbosum MH114670 (7): Laos, Thailand
Ganoderma sp. KM229678: India
G. applanatum KU194312: China
G. gibbosum MK404342: China
G. gibbosum MH114669: China
G. applanatum cplx AF255116 (5): Australia, China, Indonesia
G. australe cplx AF255129: Papua New Guinea
G. gibbosum MK345435: Myanmar
G. australe cplx AF255118: Cambodia
Ganoderma sp. MK131238: Indonesia
G. australe MN747808
G. gibbosum KJ654373
G. gibbosum KJ654404 (2)
uncultured soil fungus UDB0763169: India
G. australe cplx AF255120: Thailand
G. australe cplx AF255123/4: Malaysia
G. australe LC084717: Malaysia
G. gibbosum KU194307: China
Ganoderma sp. MK131242: Indonesia
G. gibbosum MN689631
uncultured Ganoderma KF574247: China
G. australe cplx AF255128: Papua New Guinea
G. australe JX195204: China
G. australe LC084749: Malaysia
G. applanatum KR867655: Sri Lanka
G. applanatum MF072395: India
G. tornatum KJ767488: Malaysia
G. australe cplx AF255121: Thailand
G. australe LC084663: Malaysia
G. gibbosum KU194326 (2): China
G.australe MN696209
G. australe JX195205: China
G. ellipsoideum MH106867/ NR_160617 (2): China
G. gibbosum KJ654372
G. ellipsoideum MN398339 (2): Indonesia, Thailand
G. applanatum MG448603
Ganoderma sp. KT965498: Vietnam
G. applanatum MG719303
G. gibbosum KJ654446
G. australe EU239383: Indonesia
G. australe KJ654549
G. australe KJ654550 (2)
G. australe EU239390: Indonesia
G. australe FR821768: Australia
uncultured soil fungus UDB0755590: Australia
G. australe AF255145: Malaysia
G. australe AF255142 (2): Malaysia
G. tornatum MN490058: Malaysia
G. australe KR093031: Malaysia
G. australe AF255143: Malaysia
G. australe KJ654548
G. australe EU239389: Indonesia
G. australe KJ654379
G. australe KJ654546
G. australe KJ654545 (2)
G. australe KJ654544
G. knysnamense MH571683: S. Africa
G. knysnamense MH571684: S. Africa
G. knysnamense MH571681 (2): S. Africa
G. mutabile MG231527: China
G. mutabile JN383977: China
G. cupreolaccatum MH856316
G. pfeifferi MK415263: Slovakia
G. pfeifferi MK415295: Slovakia
G. pfeifferi MG279165
G. pfeifferi MG706232 (3): Greece
G. pfeifferi AY884185 (2): UK
G. pfeifferi MK415258 (5): Slovakia
G. pfeifferi AM906059 (2): Czech Republic
G. pfeifferi AY884181: UK
G. chocoense MH890527/NR_163763: Ecuador
G. podocerpense MF796661: Ecuador
uncultured soil fungus UDB0759513: Panama
uncultured soil fungus UDB0768776: Argentina
G. lobatum AH008103 (3): Argentina
Ganoderma sp. AF255184 (4): Argentina
G. applanatum EU232190
uncultured soil fungus UDB0755383: Papua New Guinea
G. australe JX195198: China
G. cf. philippii AF255189: Vietnam
Ganoderma sp. KU219992 (2): China
G. australe MK182313
Ganoderma sp. KT318590
G. australe LT716076
Ganoderma sp. MK605943: Taiwan
G. australe MH106871 (4): China
G. australe KT965497: China
G. australe IG2 AF255193/4: Taiwan
G. australe IG2 AF255195: Taiwan
G. australe JN643731: Thailand
G. applanatum AF255191/2: China
G. australe MN396657 (6): China, Laos, Thailand
G. australe MH106872: China
Ganoderma sp. MK605938 (2): Taiwan
G. australe MK968731 (2): Vietnam
Ganoderma sp. MK774700: Taiwan
Ganoderma sp. AF255190: Taiwan
G. australe AY993918/9: India
G. australe cplx AF255183: New Zealand
G. ellipsoideum
Ganoderma sp. E3
Ganoderma sp. E4
G. knysnamense
G. mutabile
G. cupreolaccatum
G. pfeifferi
G. chocoense
G. podocarpense
Ganoderma sp. E5
Ganoderma sp. E6
Cluster E.2
Cluster E.3
Cluster E.4
CLADE E
0.98
1.00
88%
1.00
69%
1.00
0.99
92%
1.00
100%
1.00
70%
0.99
100%
1.00
86%
1.00
91%
1.00
80%
98%
1.00
100%
1.00
91%
1.00
100%
1.00
76%
0.96
94%
1.00
82%
1.00
0.98
86%
1.00
78%
0.99

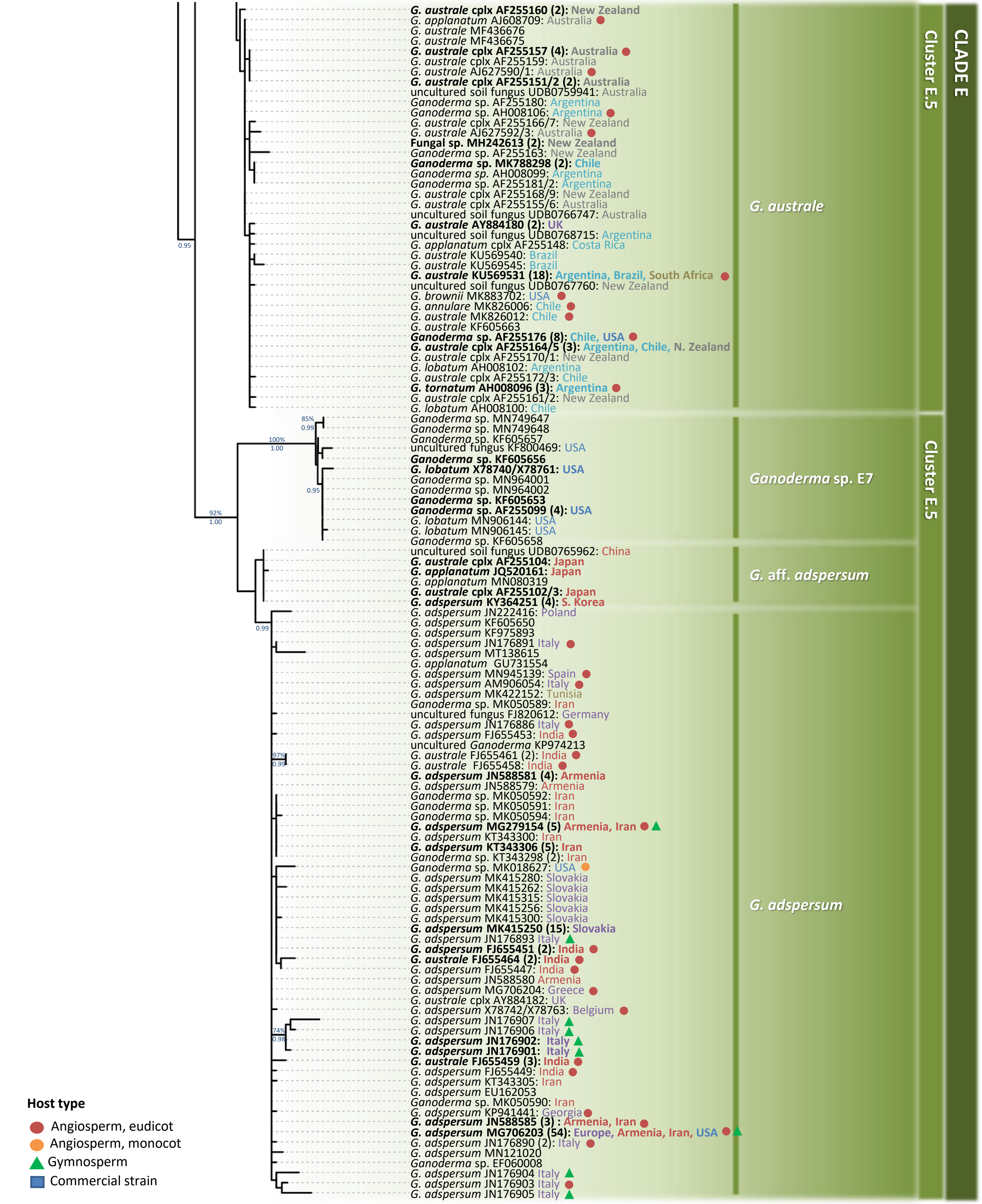

Supplementary Figure S2f. Phylogenetic reconstruction of the genus *Ganoderma* inferred from ML analysis based on ITS sequence data (pDS5; Table 2) for Clade E. ML bootstrap values (BS) ≥ 65% and Bayesian posterior probabilities (BPP) ≥ 0.95 are shown. Sequences names on the left appear as initially labelled, and are followed by the respective GenBank/ENA/DDBJ or UNITE accession no., while the total number of identical entries corresponding to a particular sequence is placed in parentheses, followed by the type of host plant (legend for the colored shapes is found at the lower left side of tree) and geographic origin of the respective material (the latter appears in different font color depending on the continent of provenance; see also Tables 1 and S2). Species names on the right correspond to those inferred in this study evaluated in conjunction with literature data. Sequences included in the respective Figure (Fig. 7) of the manuscript appear in bold typeface, while underlined sequences are those originating from type material. Scale bar: 0.01 nucleotide substitutions per site.
